# Cobalt-Catalyzed Halogen-Retentive C–H Activation and Its Application in Medicinal Chemistry

**DOI:** 10.1021/jacsau.6c00425

**Published:** 2026-08-22

**Authors:** Yongke Lei, Xiyu Luo, Lutz Ackermann, Jiayu Mo

**Affiliations:** † Guangxi Key Laboratory of Bioactive Molecules Research and Evaluation, School of Pharmacy, 74626 Guangxi Medical University, Nanning 530021, China; ‡ Wöhler Research Institute for Sustainable Chemistry (WISCh), 9375 Georg-August-Universität Göttingen, Göttingen 37077, Germany

**Keywords:** C–H activation, haloalkyne, bromoalkyne, Benzo[*c*]phenanthridinones

## Abstract

The development of C–C bond-forming strategies that retain the halogen atom represents an attractive yet challenging endeavor in synthetic chemistry, offering unique opportunities for downstream diversification. Herein, we report the halogen-retentive C–H annulation of haloalkynes, particularly bromoalkynes, enabled by an earth-abundant cobalt catalyst. The retained bromine atom serves as a versatile linchpin for subsequent C–H activation reactions, providing a general synthetic route for potential bioactive molecules and identifying a new compound with *anti*-cancer activity.

## Graphical abstract

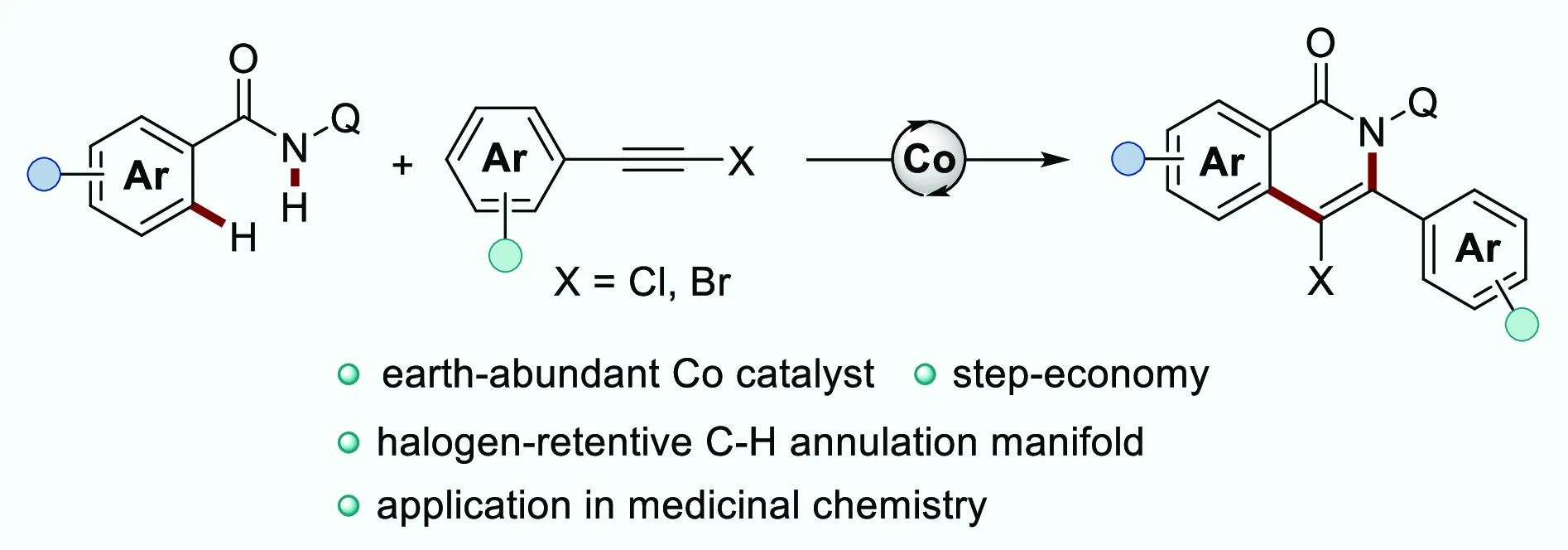

## Introduction

The assembly of carbon–carbon bonds represents a central endeavor in organic synthesis, and the development of novel, efficient C–C bond-forming approaches that enable the rapid construction of molecular complexity from simple precursors remains of paramount importance.^1^ Among the diverse classes of substrates employed to this end, organic halides occupy a privileged position.^2–4^ However, conventional strategies typically necessitate the sacrifice of the halogen atom as a leaving group, thereby compromising atom economy and, more critically, precluding its exploitation as a functional handle for downstream diversification.^3,5–9^ Consequently, the development of C–C bond-forming processes that retain the halogen atom within the product,^10–18^ especially bromo atom,^19–29^ has attracted considerable attention, as such transformations offer unique opportunities to expand both structural and functional space.

In parallel, C–H bond functionalization has emerged as a cornerstone of sustainable synthesis, embodying the principles of atom and step economy by enabling the direct conversion of simple, unactivated feedstocks into architecturally intricate products without recourse to prefunctionalized intermediates.^30,31^ The merger of this strategy with halogenated alkynes offers a particularly platform: it permits the rapid assembly of halogenated frameworks in a single operation, while the embedded halogenated moiety serves as a versatile linchpin for subsequent elaborations. Despite these conceptual advantages, halogen-retentive C–H bond activation remains underdeveloped, with the reported examples largely restricted to noble catalysts such as palladium^32^ and gold,^33,34^ which suffer from high cost, toxicity, and narrow scope. In contrast, earth-abundant 3d transition metals,^35–40^ particularly cobalt,^41–45^ will offer an attractive alternative by virtue of their low cost, low toxicity, and demonstrated capacity for chemo- and regioselective C–H functionalization. In 2005, the Daugulis group pioneered the use of 8-aminoquinoline (Q) as a bidentate auxiliary to enable directed C–H activation.^46^ This foundational strategy was subsequently extended to cobalt catalysis in 2014, when the same group developed C–H annulation with alkynes.^43^ Recently, Sundararaju and co-workers disclosed the presence of bromine-retentive 1,2-annulation in cobalt-catalyzed debrominative annulations.^47,48^ Despite these important advances, 3d-metal-catalyzed halogen-retentive C–H activation remains largely unexplored, primarily because the inherent chemoselectivity challenge, distinguishing between debrominated alkynylation^49–51^ and debrominated annulation,^47,48^ presents a significant obstacle to implementation.

We hypothesized that a suitable cobalt-catalyzed system, in combination with the 8-aminoquinoline (Q) directing group, a privileged scaffold prevalent in *anti*-cancer active molecules,^52–56^ could enhance the chemoselective C–H annulation of haloalkynes, especially bromoalkynes. This transformation would deliver bromoalkene-containing polycycles directly, with the retained bromine atom enabling modular diversification through subsequent C–H activation reactions. Specifically, we envisioned this platform as an effective strategy for integrating two antitumor scaffolds, quinoline (Q) and benzo­[*c*]­phenanthridinone (BPO), thereby enabling the assembly of hybrid molecular architectures inspired by natural products endowed with significant antitumor activity.^57–62^ Herein, we report the realization of this strategy, which establishes cobalt-catalyzed directed C–H activation as an enabling manifold for the rapid, programmable assembly of halogenated polycyclic frameworks, specifically 4-halogenated isoquinolones, from easily available bromoalkyne precursors. Notable features of this approach include: (i) good atom economy; (ii) operation with an earth-abundant and less toxic cobalt catalyst; (iii) broad functional group compatibility; (iv) access to novel compounds with potential bioactivity; and (v) identification of a novel compound with *anti*-cancer activity (Scheme 1).

**Scheme 1:**
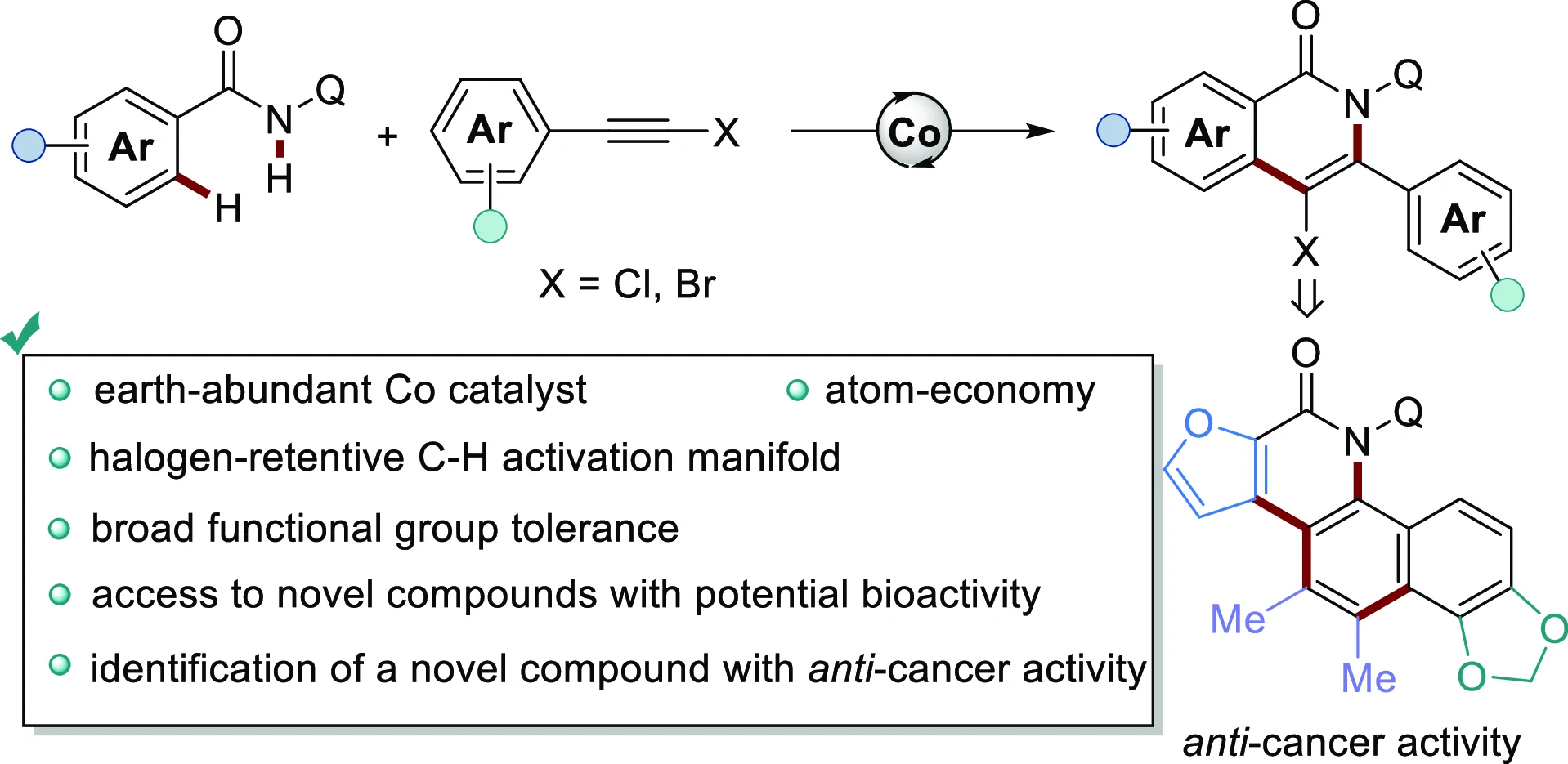
Co-Catalyzed Halogen-Retentive C–H Annulation

## Results and Discussion

We selected readily available cobalt­(II) acetate as the catalyst in combination with bis­(ethoxycarbonyl)-2,2′-bipyridine as the ligand, using sodium chlorate, copper­(II), and air as the oxidation environment. With this combination, preliminary optimization identified 2,2,2-trifluoroethanol (TFE) as the optimal solvent (Table 1, entries 1–3). The electronic properties of the bidentate nitrogen-based ligand exerted a slight influence on this transformation (Table 1, entries 4–6). Both the use of a monodentate ligand and the complete absence of ligand resulted in low efficiency (Table 1, entries 7–9).^48,63^ Cobalt­(II) bromide exhibited less efficiency toward cobalt­(II) acetate (Table 1, entry 10).^64^ Notably, ambient air proved optimal, whereas pure oxygen accelerated undesired β-Br elimination side reaction (Table 1, entries 11; Table S13 and Supporting Information Section 5), and nitrogen atmosphere nearly shut down the reaction (Table 1, entries 12). Other oxidants, such as silver nitrate and *p*-benzoquinone (PBQ), proved less effective than sodium chlorate (Table 1, entries 13 and 14). These observations indicate that sodium chlorate and copper­(II) function as the terminal oxidants. Meanwhile, air is likely required to mediate the oxidation of cobalt­(II) to cobalt­(III), a step for enabling the C–H activation process. Prolonged reaction times did not lead to debromination, indicating the target product was stable under identical conditions (Table 1, entry 15).

**Table 1:**
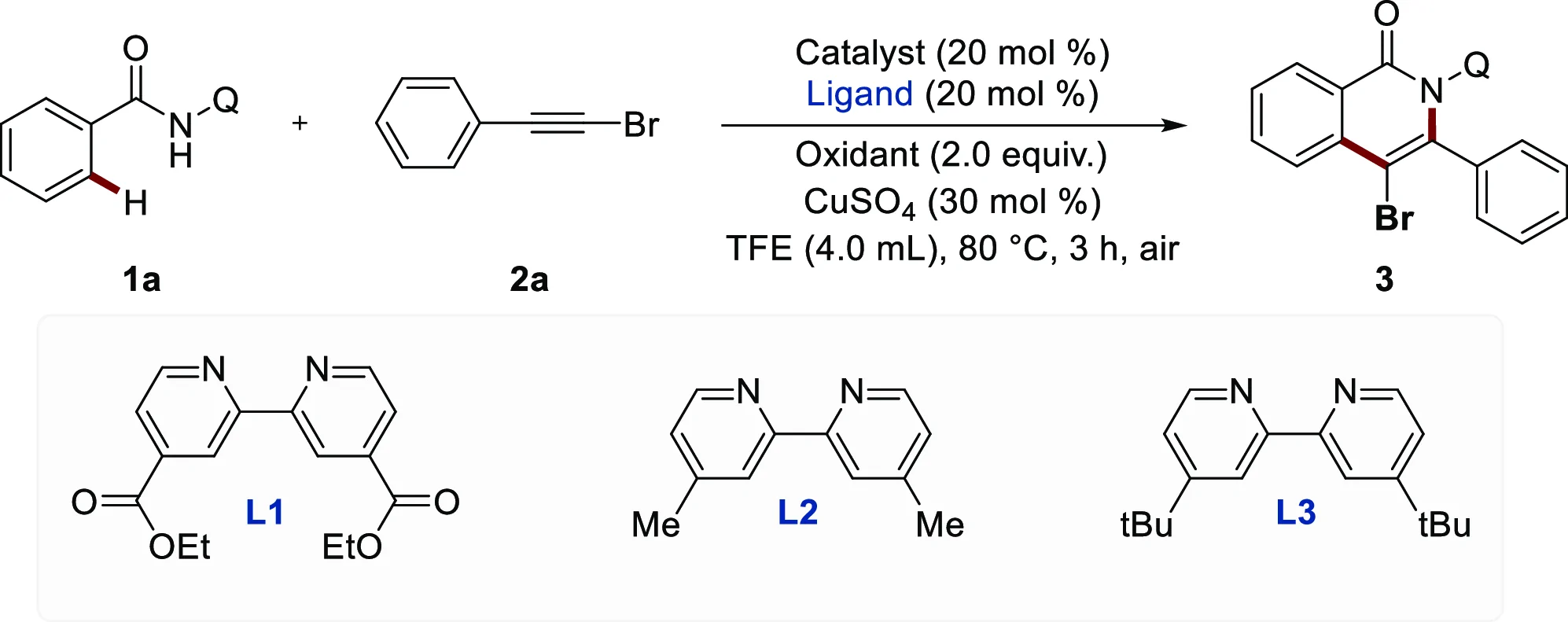
Optimization of the Reaction Conditions^a^

entry	catalyst	oxidant	ligand^a^	solvent	yield
1	Co(OAc)_2_	NaClO_3_	L1	EtOH	trace
2	Co(OAc)_2_	NaClO_3_	L1	HFIP	40
3	Co(OAc)_2_	NaClO_3_	L1	TFE	63
4	Co(OAc)_2_	NaClO_3_	L2	TFE	58
5	Co(OAc)_2_	NaClO_3_	L3	TFE	50
6	Co(OAc)_2_	NaClO_3_	2,2′-bipyridine	TFE	57
7	Co(OAc)_2_	NaClO_3_	pyridine	TFE	17
8	Co(OAc)_2_	NaClO_3_	DMAP	TFE	33
9	Co(OAc)_2_	NaClO_3_	-	TFE	30
10	CoBr_2_	NaClO_3_	L1	TFE	45
11^b^	Co(OAc)_2_	NaClO_3_	L1	TFE	40
12^c^	Co(OAc)_2_	NaClO_3_	L1	TFE	trace
13	Co(OAc)_2_	AgNO_3_	L1	TFE	30
14	Co(OAc)_2_	PBQ	L1	TFE	15
15^d^	Co(OAc)_2_	NaClO_3_	L1	TFE	63

a Reaction conditions: **1a** (0.30 mmol), **2a** (0.45 mmol), catalyst (20 mol %), ligand (20 mol %), oxidant (2.0 equiv), CuSO_4_ (30 mol %), TFE (4.0 mL), 80 °C, 3 h, under air, Isolated yields.

b Under O_2_ atmosphere.

c Under N_2_ atmosphere.

d 10 h. DMAP = 4-dimethylaminopyridine.

With the optimized reaction conditions for C–H/N–H cyclization in hand, we evaluated the substrate scope to delineate the application potential of this transformation (Scheme 2). Initially, various substituents on the phenyl ring of 8-AQ benzamides were examined (Scheme 2A). A range of electron-donating (**4**, **6**–**9**) and electron-withdrawing (**5**, **10**–**19**) groups were well tolerated on either the directing group or the benzamide fragment, affording the corresponding products in 25%–67% yield. Notably, the catalytic system exhibited compatibility with catalyst-poisoning groups (**8** and **9**), strongly electron-withdrawing groups (**14**–**16**), oxidation-sensitive groups (**8** and **19**), bioactivity relevant groups (**10**, **14**, **16**–**18** and **20**), as well as pharmaceutically relevant heterocycles (**22**–**24**). With respect to haloalkynes, chloroalkyne **25** proved equally competent to bromoalkynes (Scheme 2B), under the optimized conditions, affording the product in 70% isolated yield. For bromoalkynes, electron-donating substituents (**26**–**29**) on the phenyl ring exerted negligible effects on the reaction efficiency, whereas electron-withdrawing groups (**30**–**38**) slightly enhanced the reactivity, delivering the corresponding products in moderate yields. Bromoalkynes bearing sensitive functional groups, including cyano (**34**), nitro (**35**), acyl (**36**, **37**), and aldehyde (**38**) moieties, proceeded smoothly to give the products in moderate yields. Furthermore, this strategy was applied to bromoalkynes bearing diverse structures, including benzo-fused (**39**) frameworks, heterocycles (**40**–**42**), and alkyl groups (**43**–**44**). The linear aliphatic bromoalkyne delivered the desired product **43** in 65% yield, retaining identical regioselectivity as aryl bromoalkyne counterparts. In contrast, sterically hindered cyclic alkyl bromoalkyne mainly afforded product **44′** with reverse regioselectivity. These results suggest that the observed regioselectivity is likely governed by the steric bulk of the bromoalkyne substituent.^65,66^

**Scheme 2:**
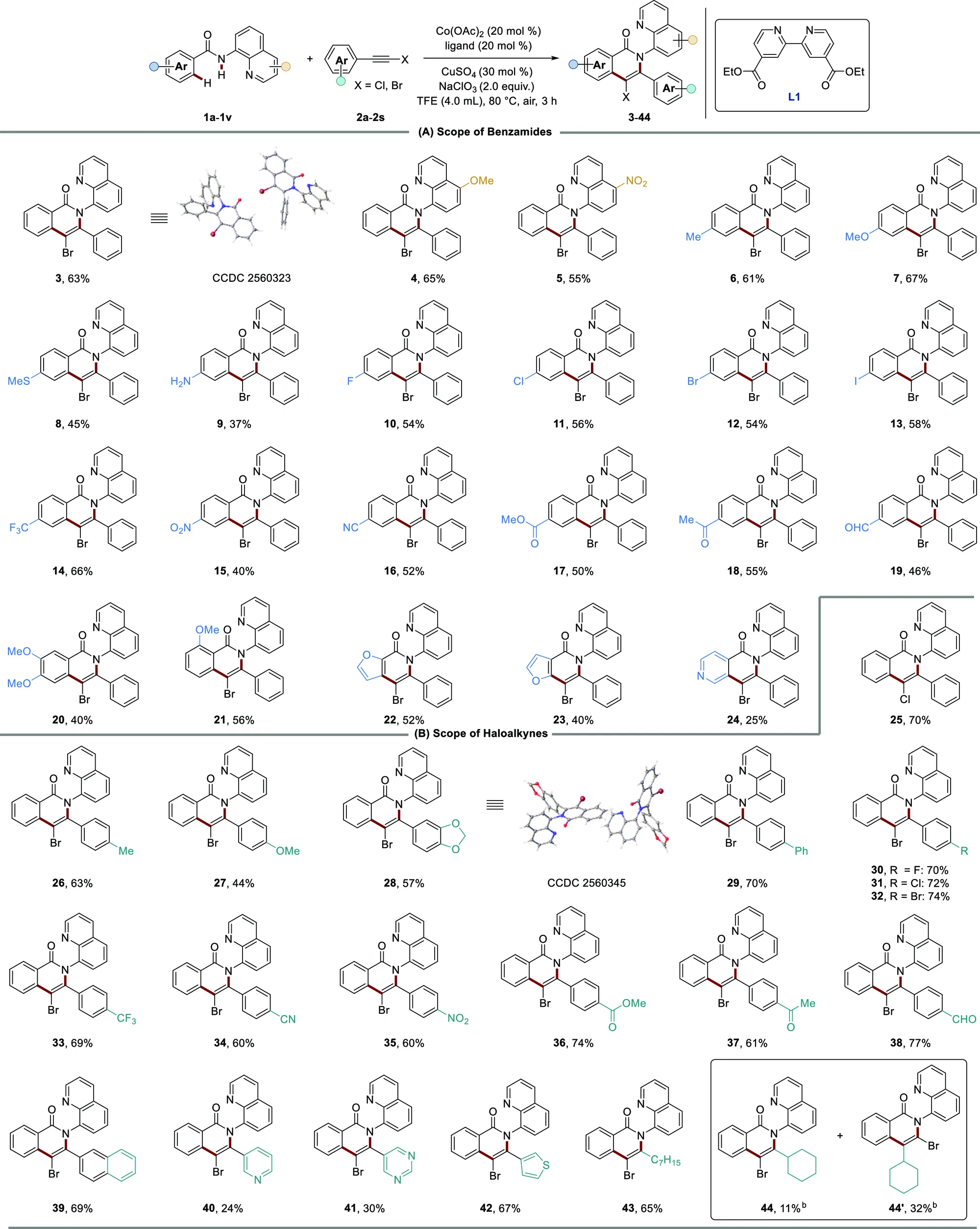
Substrate Scope^a^,^b^

a Reaction conditions: **1a**-**1v** (0.30 mmol), **2a**-**2s** (0.45 mmol), Co­(OAc)_2_ (20 mol %), **L1** (20 mol %), CuSO_4_ (30 mol %), NaClO_3_ (2.0 equiv), TFE (4.0 mL), 80 °C, 3 h, under air, isolated yield.
b The yield was determined by ^1^H-NMR analysis of the isolated isomer mixture.

To evaluate the general applicability of this strategy in medicinal chemistry, we designed a series of molecular fragments guided by SAR principles for the assembly of potential antitumor molecules containing the BPO alkaloid scaffold (Figure 1A). The C–H/N–H annulation with bromoalkynes successfully enabled the assembly of brominated intermediates **45**–**48** in moderate efficiency (Figure 1B). Subsequent direct adaptation^67^ or minimal modification^68^ of established palladium-catalyzed conditions provided efficient access to tetracyclic scaffolds **49**–**54**. Notably, this platform accommodates both the installation of alkaloid-privileged substituents (**50**–**53**) and the execution of common medicinal chemistry maneuvers including bioisosteric replacement (**49**) and scaffold morphing (**54**). Preliminary biological evaluation revealed that compound **49** exhibits antitumor potency comparable to the reference compound camptothecin, further demonstrating the potential of this synthetic strategy in medicinal chemistry (Figure 1C). Thus, this synthetic manifold provides an access to the assembly of novel compounds with potential *anti*-cancer activity, thereby facilitating the construction of focused compound libraries.

**Figure 1:**
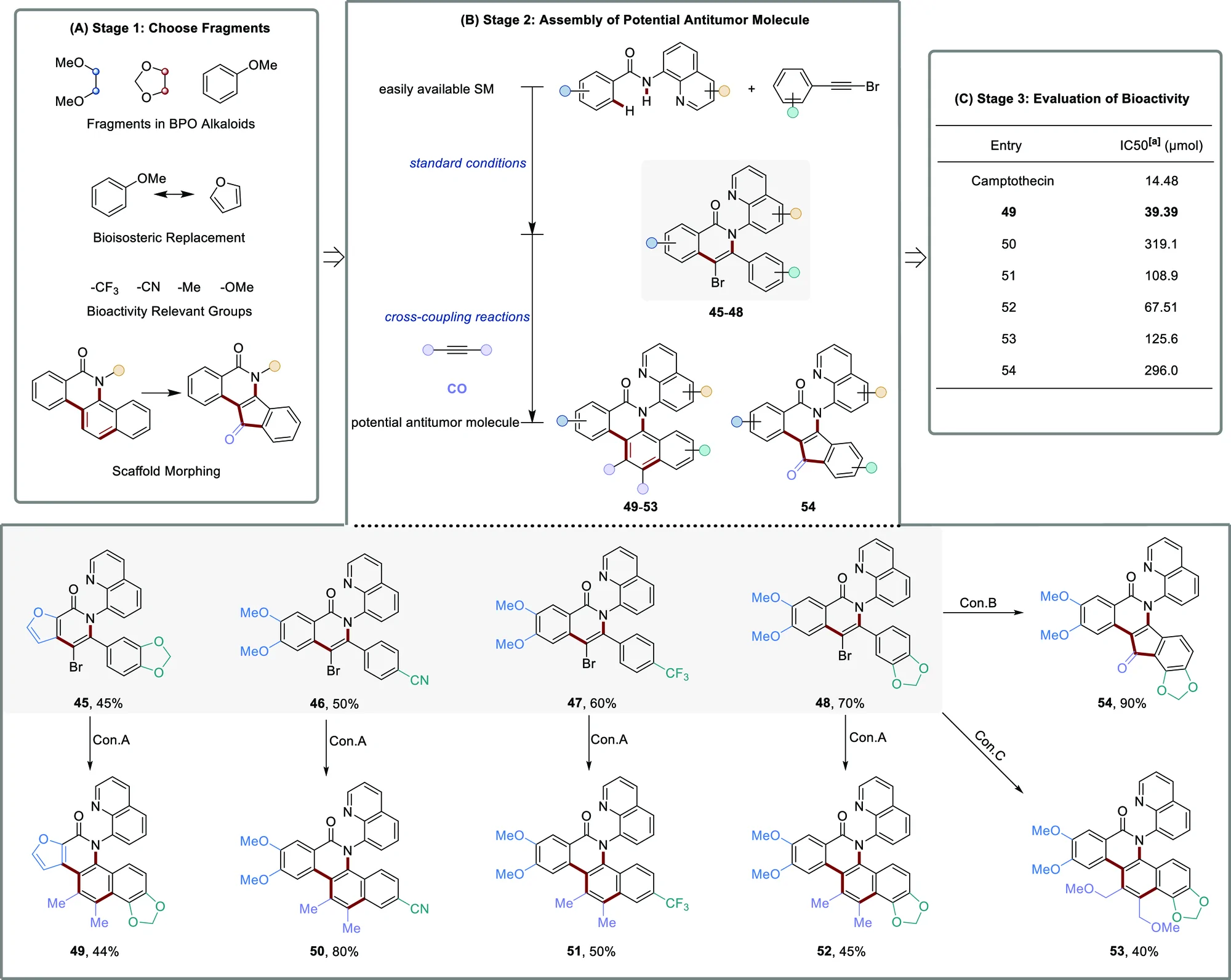
Synthetic Applications. Con. (A): **45**-**48** (0.20 mmol), 2-butyne (0.80 mmol), Pd­(PPh_3_)_4_ (5.0 mol %), Cs_2_CO_3_ (3.0 equiv) and DMF (4.0 mL), N_2_, 5 h, 110 °C. Con. (B): **48** (0.20 mmol), Pd­(PCy_3_)­Cl_2_ (5.0 mol %), CsOPiv (2.0 equiv) and DMF (4.0 mL), CO, 16 h, 110 °C. Con. (C): **48** (0.20 mmol), 1,4-dimethoxy-2-butyne (4.0 equiv), Pd­(PPh_3_)_4_ (5.0 mol %), Cs_2_CO_3_ (3.0 equiv) and DMF (4.0 mL), N_2_, 5 h, 110 °C. ^(a)^The compound concentration resulting in 50% inhibition of Huh-7 cell viability (CCK-8 assay).

To gain deeper mechanistic insights, a series of control experiments were conducted. Competition experiments with benzamides **1d** and **1l** revealed that electron-donating benzamides exhibited kinetically superior reactivity (Scheme 3A). This finding is consistent with a base-assisted intramolecular electrophilic substitution (BIES) mechanism.^69,70^ Kinetic isotope effect (KIE) studies indicated that C–H bond cleavage is neither the rate-determining step nor the first irreversible step of the reaction (Scheme 3B).

**Scheme 3:**
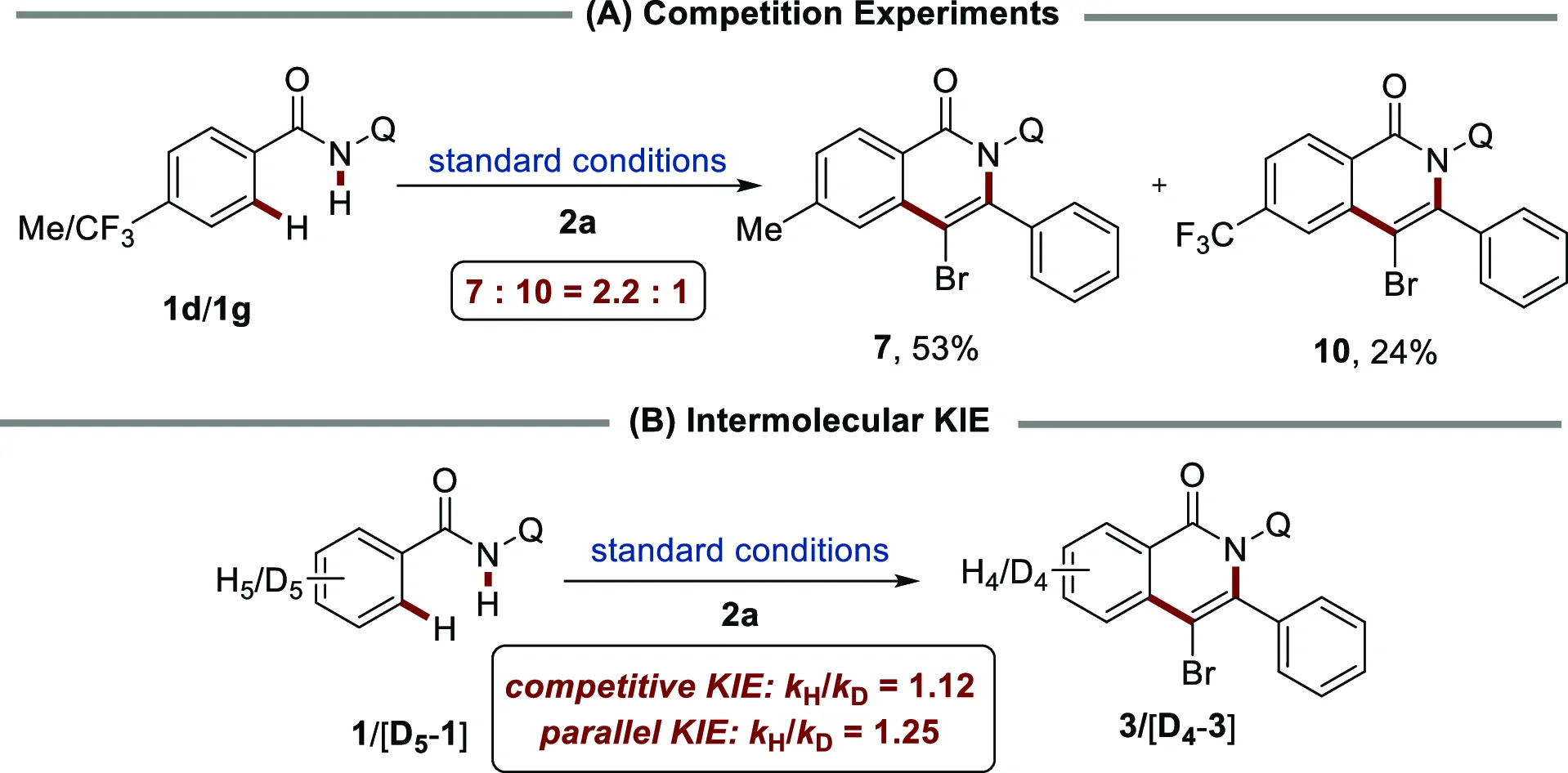
Mechanistic Studies

Based on mechanistic experiments and previous mechanistic insights into cobalt-catalyzed C–H activation,^65,66,69–71^ a plausible reaction mechanism is proposed as follows. Substrate **1a** initially coordinates to the cobalt center via ligand exchange, generating **Int-I**. The cobalt of **Int-I** is then oxidized to provide **Int-II**, which undergoes C–H activation to deliver **Int-III**. Coordination of alkyne **2a** to **Int-III** subsequently affords **Int-IV**. Depending on the steric bulk of the bromoalkyne substituent, **Int-IV** undergoes either 2,1-migratory insertion with a small substituent on the alkyne, leading to **Int-V**, while 1,2-migratory insertion would occur with a bulky alkyne. Subsequent reductive elimination delivers product **3** with concomitant generation of **Int-VI**. Finally, oxidation of **Int-VI** regenerates the active catalyst **Int-I**, completing the catalytic cycle (Scheme 4).

**Scheme 4:**
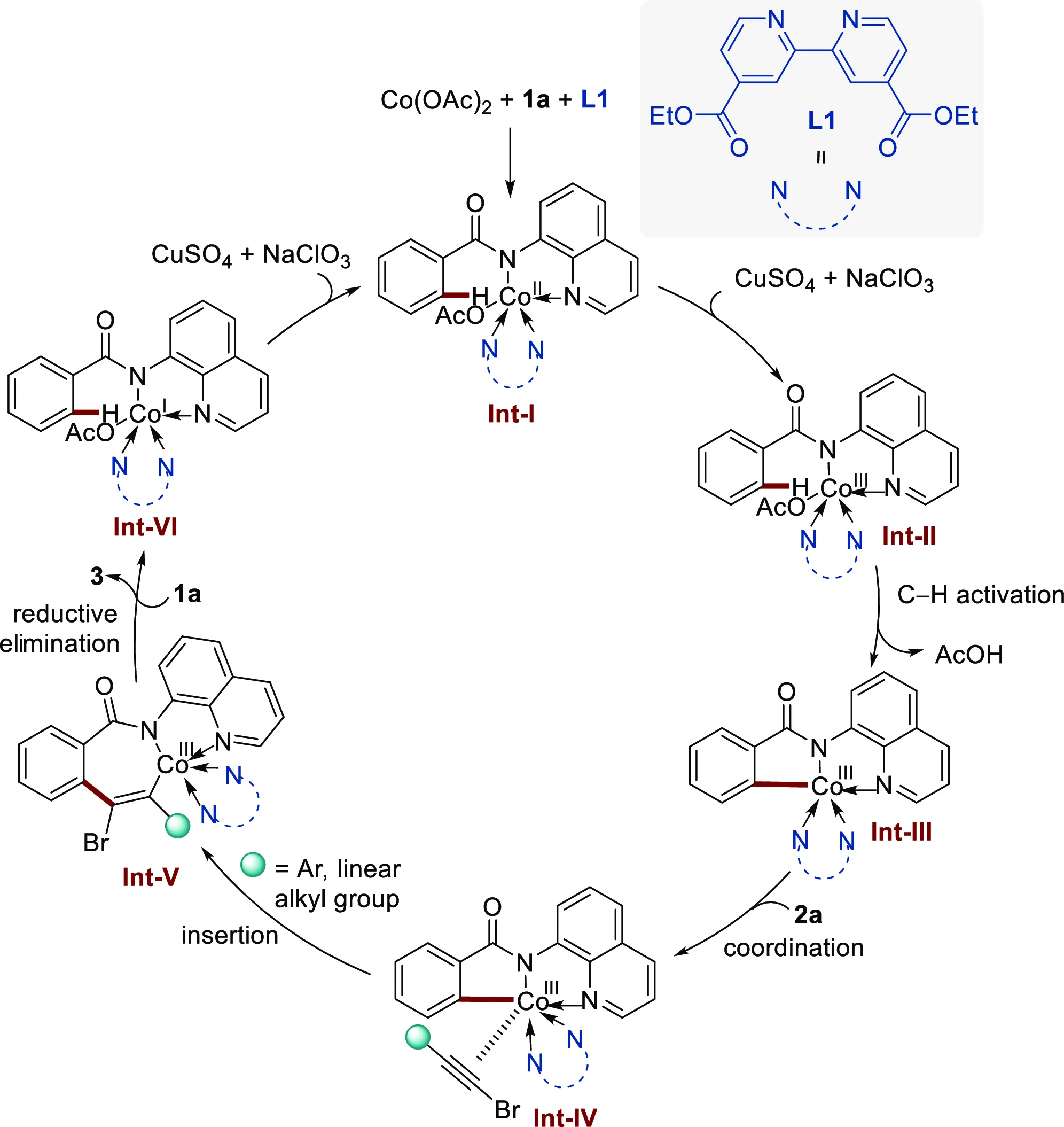
Proposed Mechanism

## Conclusion

In summary, we have achieved cobalt-catalyzed halogen-retentive C–H annulation, establishing an earth-abundant metal platform for C–C bond formation with retained halogen functionality. The strategy’s chemoselectivity—overcoming competing debromination pathways—stems from the synergistic combination of cobalt catalytic system and the 8-aminoquinoline directing group, enabling direct access to bromoalkene-containing polycycles from unactivated feedstocks. The retained bromine atom serves as a strategic linchpin for divergent postannulation modifications, expanding the structural space accessible from a common intermediate. The merging of two anticancer molecular scaffolds and the discovery of a novel compound with *anti*-cancer activity illustrating the practical value of this strategy in medicinal chemistry.

## Supplementary Material



## Data Availability

The data underlying this study are available in the published article and its Supporting Information.

## Notes

Experimental details and characterization data, including ^1^H, ^13^C, ^19^F NMR, 2D NMR spectra and HRMS (PDF) (PDF)
