# Supplementary material for: Cobalt-Catalyzed Halogen-Retentive C–H Activation and Its Application in Medicinal Chemistry
Source: JACS Au. 2026 Aug 22;6(9):4922–8. doi: 10.1021/jacsau.6c00425 (PMC13625552; doi:10.1021/jacsau.6c00425)
Supplement: Supplementary file 1 [file au6c00425_si_001.pdf]

Supporting Information

Cobalt-Catalyzed Halogen-Retentive C–H Activation and Its Application in Medicinal Chemistry

Yongke Lei,[a‡] Xiyu Luo,[a‡] Lutz Ackermann,[b*] and Jiayu Mo[a*]

[a]Guangxi Key Laboratory of Bioactive Molecules Research and Evaluation,

School of Pharmacy, Guangxi Medical University,

Shuangyong Road 22, 530021 Nanning, P. R. China

E-mail: Jiayumo@gxmu.edu.cn

[b]Wöhler Research Institute for Sustainable Chemistry (WISCh),
Georg-August-Universität Göttingen
Tammannstrasse 2, 37077 Göttingen, Germany
Fax: +49/ 551-39-6777

E-mail: Lutz.Ackermann@chemie.uni-goettingen.de

Table of Contents

1. General Remarks....................2

2. Synthesis of Substrates....................2

3. General Procedures....................4

4. Optimization of Reaction Conditions....................6

5. Origin of Debromination....................12

6. HUH-7 Cell Experiments....................13

7. Mechanistic Research....................14

8. Mass Balance Analysis....................17

9. Characterization Data....................18

10. Reaction with TIPS-Substituted Bromoalkyne....................50

11. References....................50

12. NMR Spectra....................50

1. General Remarks

Unless otherwise noted, catalytic reactions were carried out in pre-dried glassware (10 mL). All reagents and solvents were purchased from commercial suppliers (Adamas-beta, Bidepharm, General Reagent) and used without further purification unless mentioned otherwise. Thin-layer chromatography (TLC) was performed on 200 ± 50 μm glass-backed silica gel plates purchased from Shanghai Titan Technology Co., Ltd., China. Column chromatography was carried out on silica gel (200-300 mesh) purchased from Yantai Xinnuo Chemical Co., Ltd., China.

NMR spectra were recorded on Bruker Avance NEO 600 instrument. Chemical shifts (δ) are given in ppm relative to the solvent residual peak, multiplicity (s = singlet, d = doublet, t = triplet, q = quartet, m = multiplet, bs = broad singlet), and coupling constants (J) are in Hertz (Hz). High revolution mass spectra (HRMS) were obtained on an Agilent 6545 Q-TOF.

The materials and instruments used for the CCK-8 cytotoxicity assay were as follows. Micropipettes were from Thermo Fisher Scientific. A NU-425-400S biological safety cabinet (NUAIRE) and a NU-5800 CO_2 incubator (NUAIRE) were employed. Cell imaging was performed using an MF53-N inverted fluorescence microscope (Mshot). Centrifugation steps utilized a Legend Micro 17R refrigerated microcentrifuge (Thermo Fisher Scientific). A BK-3D constant temperature water bath (Shanghai Benteng Instrument Co., Ltd.) and an SPECTRAMAX iD3 multi-mode microplate reader (Molecular Devices) were also used. Key reagents included DMEM (Vivacell; C3113), fetal bovine serum (FBS; Qisai Biology; QS-H295A), Penicillin- Streptomycin Solution (100×; Qisai Biology; QS-H295A), 1× phosphate-buffered saline (PBS; Hyclone; SH30256), and the Cell Counting Kit-8 (CCK-8; Beyotime; C0038). The human hepatocellular carcinoma cell line HUH-7 (catalog numbers QS-H018, respectively) were used in this study.

2. Synthesis of Substrates

The amides **1b**[1], **1c**[2] were synthesized according to literature procedures. Unless otherwise noted, other amide compounds were synthesized by the following methods.

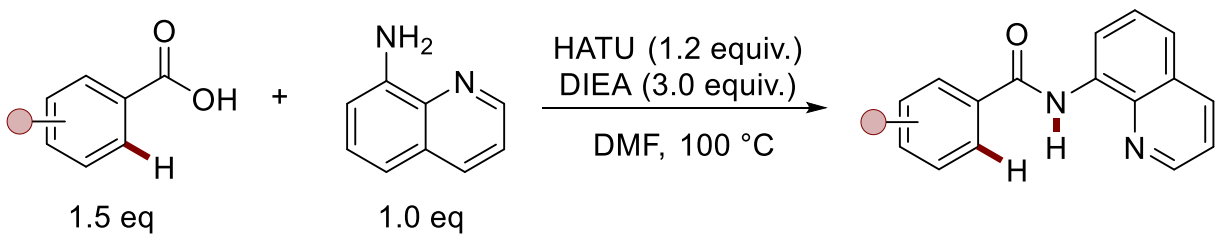

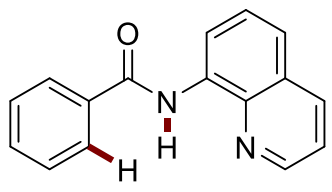

1a

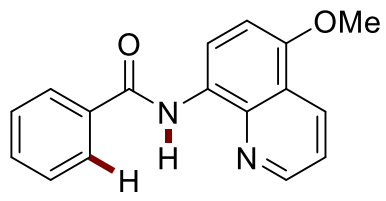

1b

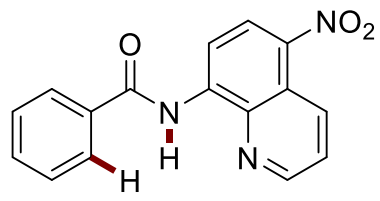

1c

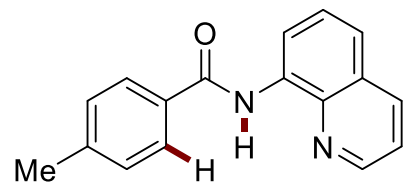

1d

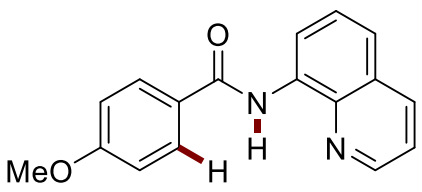

1e

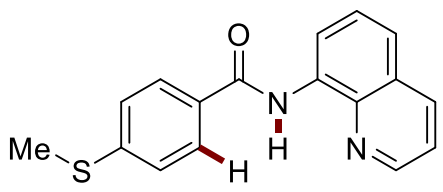

1f

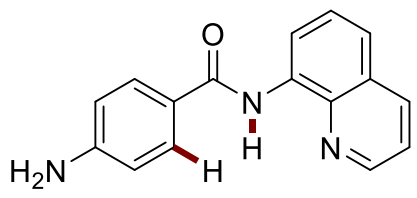

1g

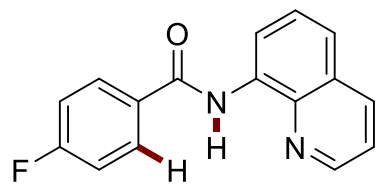

1h

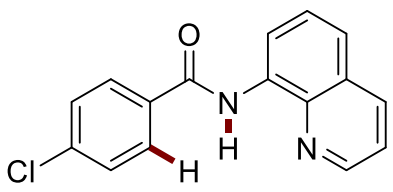

1i

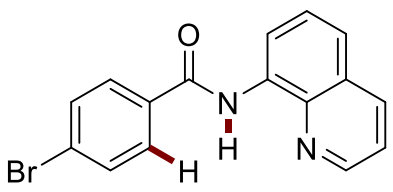

1j

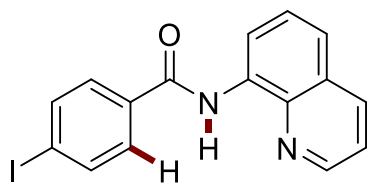

1k

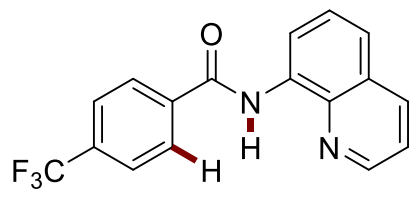

1l

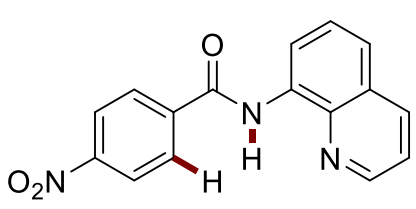

1m

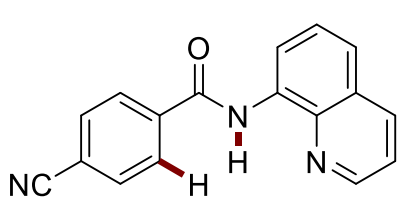

1n

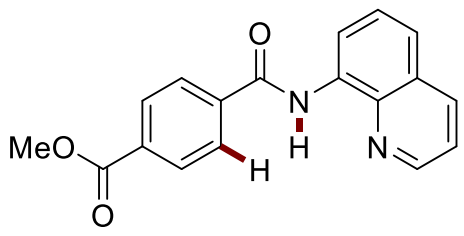

1o

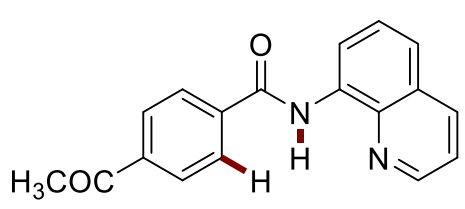

1p

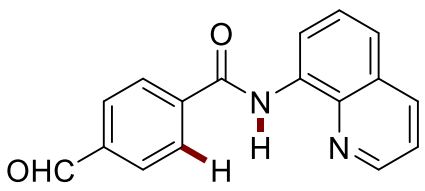

1q

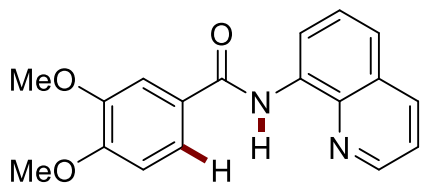

1r

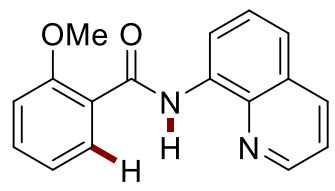

1s

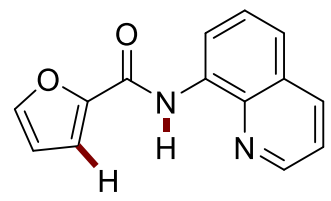

1t

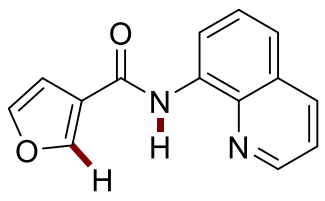

1u

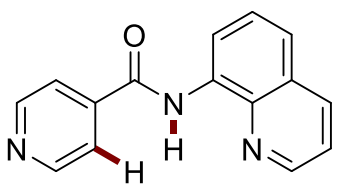

1v

Alkynes were synthesized according to literature procedures[3]. The characterization data were consistent with those reported.

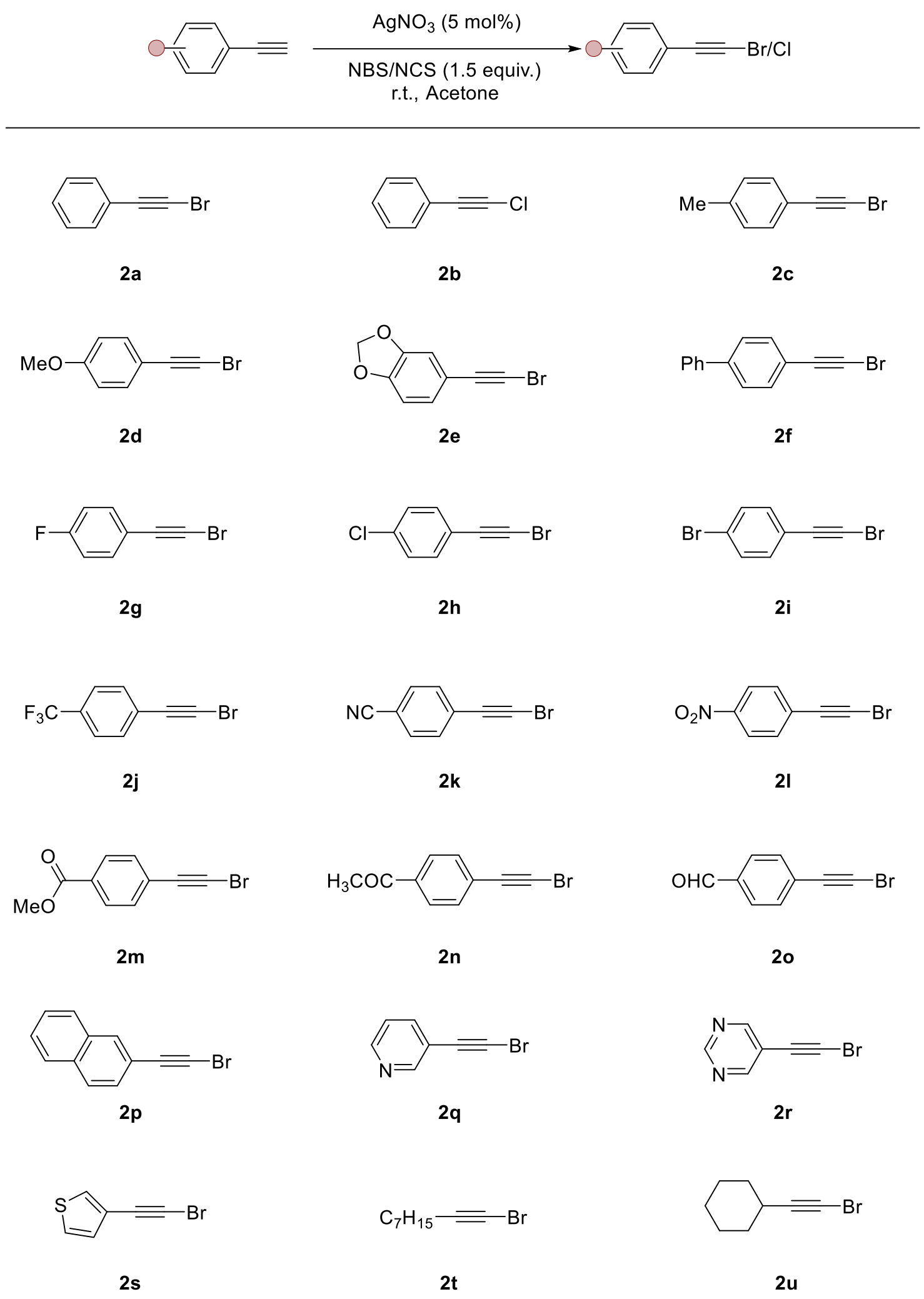

3. General Procedures

3.1. Procedure A

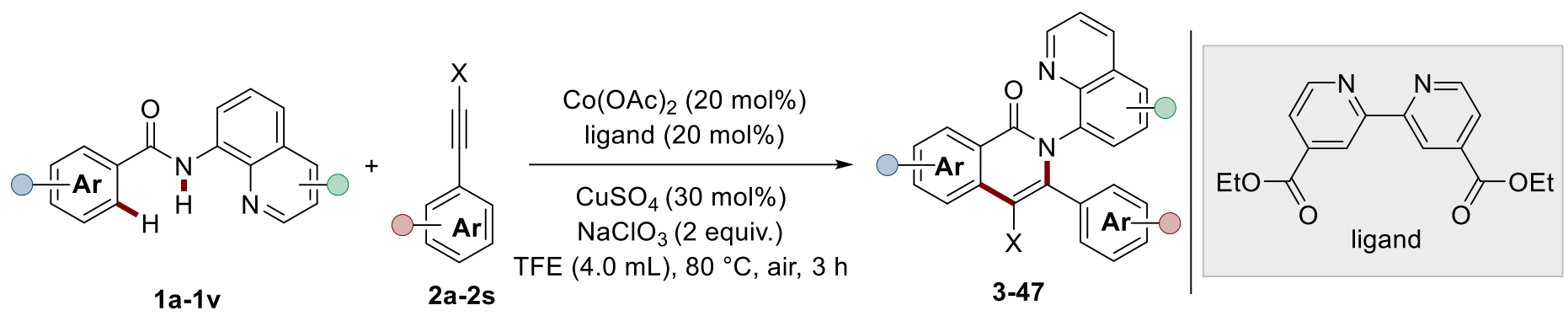

A 10 mL schlenk tube equipped with a magnetic stir bar was charged with *N*-(quinolin-8-yl)benzamide **1a**–**1v** (0.30 mmol), alkyne **2a**–**2s** (0.45 mmol, 1.5 equiv), $Co(OAc)_2$ (0.06 mmol, 20 mol%), $CuSO_4$ (0.09 mmol, 30 mol%), $NaClO_3$ (0.60 mmol, 2.0 equiv.), ligand (0.60 mmol, 20 mol%) and TFE (4.0 mL). The mixture was stirred for 3 hours under air at 80 °C. After completion of the reaction, the solvent was then removed under vacuum and the residue was purified by column chromatography on silica gel to afford the corresponding products **3**–**46**.

3.2. Procedure B[3]

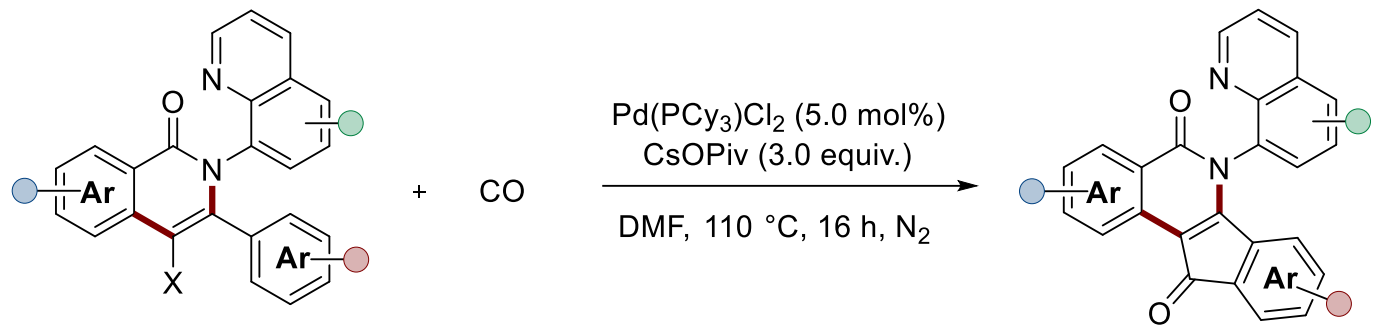

Under CO atmosphere, a 10 mL schlenk tube equipped with a magnetic stir bar was charged with compound **46** (0.20 mmol), $Pd(PCy_3)Cl_2$ (0.01 mmol), CsOPiv (0.40 mmol) and DMF (4.0 mL). The mixture was stirred for 16 hours at 110 °C. The resulting mixture was diluted with EA (20 mL) and Washed with saturated sodium chloride solution (3 × 60 mL). The combined organic phase was dried over Na_2SO_4 and filtered. The filtrate was concentrated under reduced pressure at 50 °C. The residue was purified by chromatography on silica gel to afford the corresponding products **52**.

3.3. Procedure C

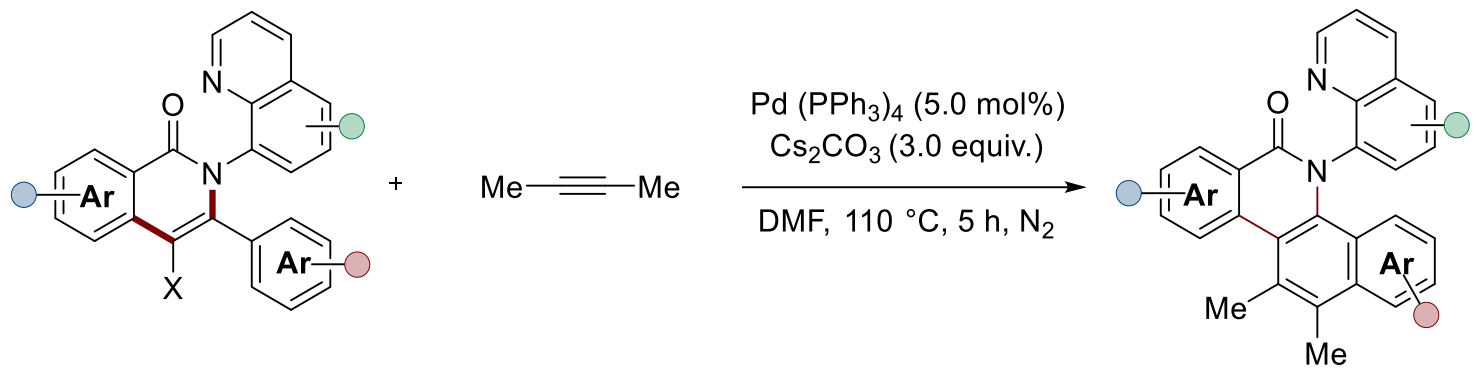

Under N_2 atmosphere, a 10 mL schlenk tube equipped with a magnetic stir bar was charged with compound **43**–**46** (0.20 mmol), 2-butyne (0.80 mmol), $Pd(PPh_3)_4$ (0.01 mmol), Cs_2CO_3 (0.60 mmol) and DMF (4.0 mL). The mixture was stirred for 5 hours at 110 °C. The resulting solution was diluted with EA (20 mL) and washed with saturated sodium chloride solution (3 × 60 mL). The combined organic phase was dried over Na_2SO_4 and filtered. The filtrate was concentrated under reduced pressure at 50 °C. The residue was purified by chromatography on silica gel to afford the corresponding products **47**–**50.**

3.4. Procedure D

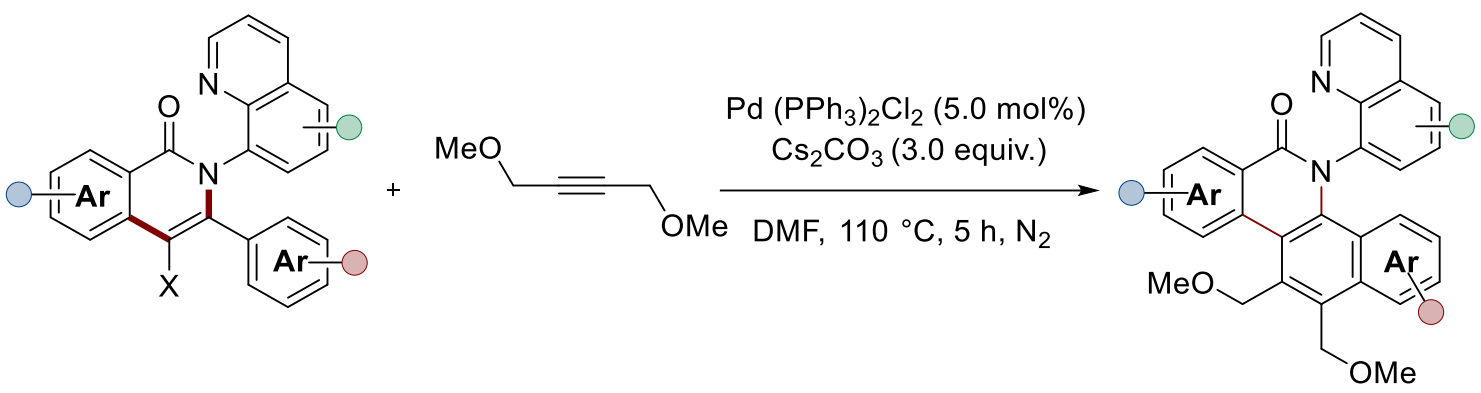

Under N_2 atmosphere, a 10 mL Schlenk tube equipped with a magnetic stir bar was charged with compound **46** (0.20 mmol), 1,4-dimethoxy-2-butyne (0.80 mmol), $PdCl_2(PPh_3)_2$ (0.01 mmol), Cs_2CO_3 (0.60 mmol) and DMF (4.0 mL). The mixture was stirred for 5 hours at 110 °C. The resulting mixture was diluted with EA (20 mL) and washed with saturated sodium chloride solution (3 × 60 mL). The combined organic phase was dried over Na_2SO_4 and filtered. The filtrate was concentrated under reduced pressure at 50 °C. The residue was purified by chromatography on silica gel to afford the corresponding product **51**.

4. Optimization of Reaction Conditions

4.1. Optimization of Solvent

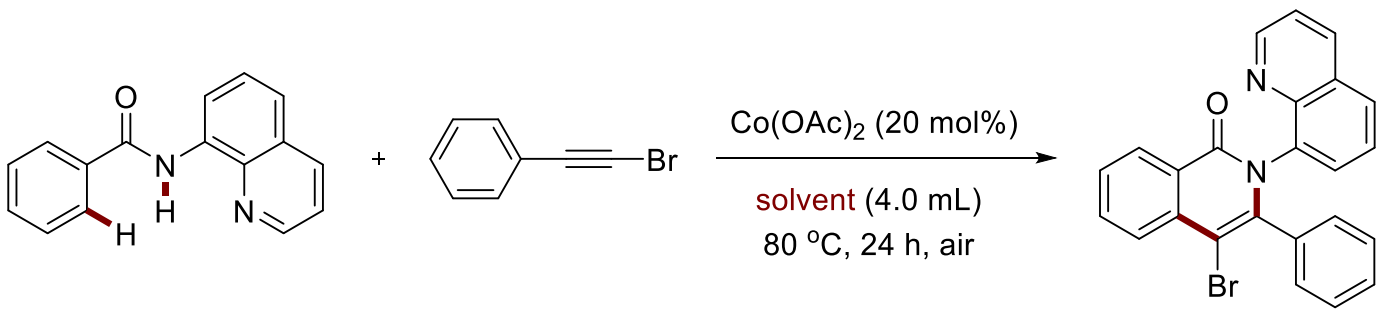

1a 2a 3

Entry	Solvent	Yield (%)
**1**	**TFE**	**10**
2	Chlorobenzene	trace
3	DMAc	trace
4	*tert*-Pentanol	3
5	1,4-Dioxane	trace
6	Toluene	5
7	EtOH	trace
	HFIP	8

Table S1. Reaction conditions: **1a** (0.30 mmol), **2a** (0.45 mmol), $Co(OAc)_2$ (0.06 mmol), solvent (4.0 mL), 80 °C, 24 h, under air. The yield was determined by LC-MS analysis.

4.2. Screening of Catalyst

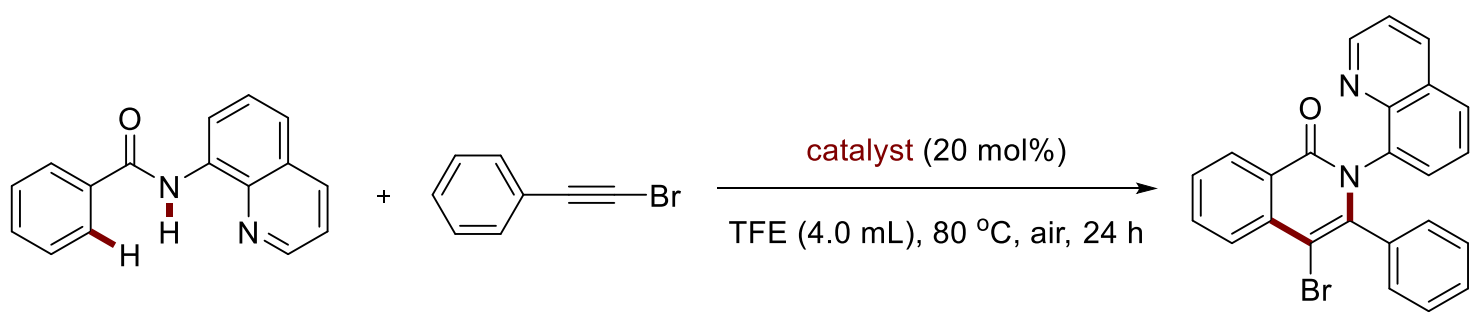

1a 2a 3

Entry	Catalyst	Yield (%)
**1**	**$Co(OAc)_2$**	**10**
2	$Cp^*Co(CO)I_2$	10
3	$Co_2(CO)_8$	15
4	$Co(acac)_2$	NR
5	$CoBr_2$	15
6	$Ni(OAc)_2$	NR

Table S2. Reaction conditions: **1a** (0.30 mmol), **2a** (0.45 mmol), catalyst (0.06 mmol), TFE (4.0 mL), 80 °C, 24 h, under air. The yield was determined by LC-MS analysis.

4.3. Screening of Base

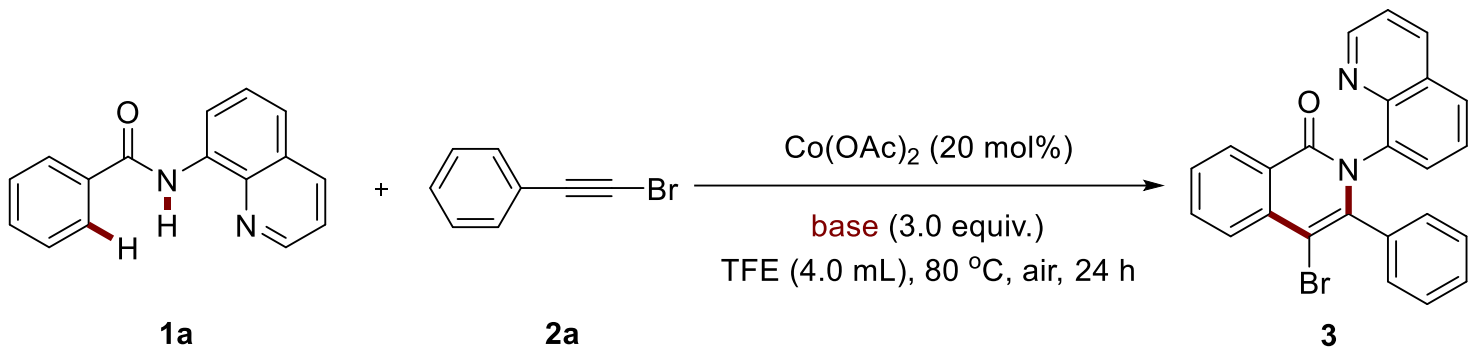

Entry	Base	Yield (%)
1	Na_2CO_3	13
2	EtONa	NR
3	EtMgBr	NR
4	Pyridine	5
5	Triethylamine	trace
6	$[CH_3(CH_2)_3]_4NClO_4$	3
7	CF_3CH_2ONa	5
8	NaOPiv	15[a]

Table S3. [a] Substantial pivalate-substituted product (bromine displaced) was produced. Reaction conditions: **1a** (0.30 mmol), **2a** (0.45 mmol), $Co(OAc)_2$ (0.06 mmol), base (0.90 mmol), TFE (4.0 mL), 80 °C, 24 h, under air. The yield was determined by LC-MS analysis.

4.4. Screening of Oxidant

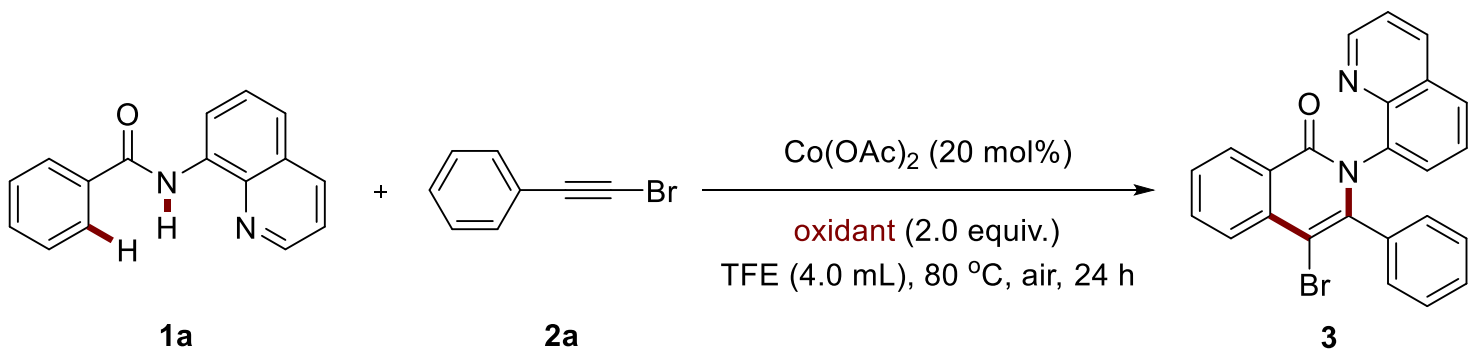

Entry	Oxidant	Yield (%)
1	$AgNO_3$	21
2	$Mn(OAc)_2$	7
3	*m*-CPBA	NR
4	Benzoquinone	7
5	H_2O_2	trace
**6**	**$NaClO_3$**	**20**
7	(Diacetoxyiodo)benzene	trace

8	C(+)/Pt(-), (4 mA), TBAF, (16 h)	NR
9	Na_2[Eosin Y], 450nm, 10w, (0.4 equiv.)	trace
10	O_2	trace

Table S4. Reaction conditions: **1a** (0.30 mmol), **2a** (0.45 mmol), $Co(OAc)_2$ (0.06 mmol), oxidant (0.60 mmol), TFE (4.0 mL), 80 °C, 24 h, under air. The yield was determined by LC-MS analysis.

4.5. Screening of Ligand

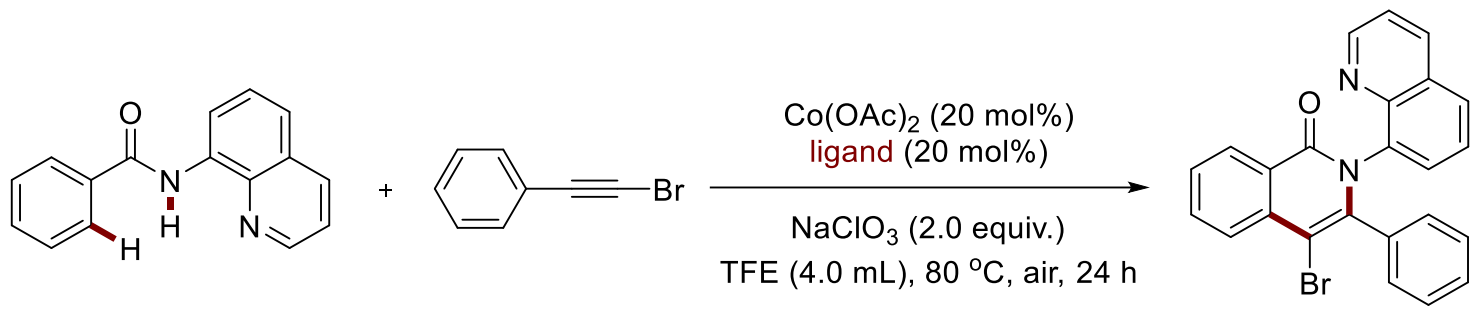

1a **2a** **3**

Entry	Ligand	Yield (%)
1	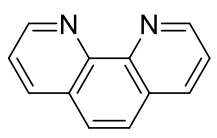	15
2	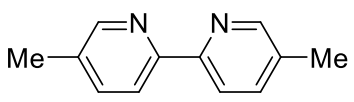	20
3	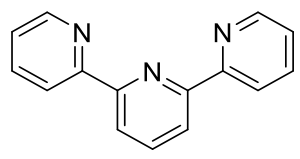	NR
4	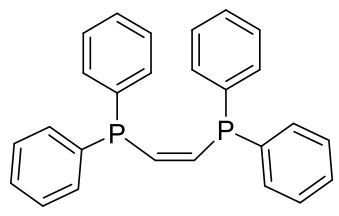	NR
5	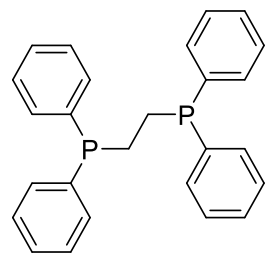	NR
6	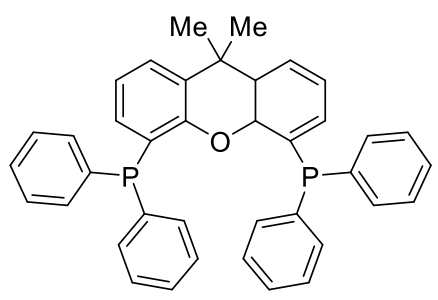	NR

Table S5. Reaction conditions: **1a** (0.30 mmol), **2a** (0.45 mmol), $Co(OAc)_2$ (0.06 mmol), ligand (0.06 mmol), TFE (4.0 mL), 80 °C, 24 h, under air. The yield was determined by LC-MS analysis.

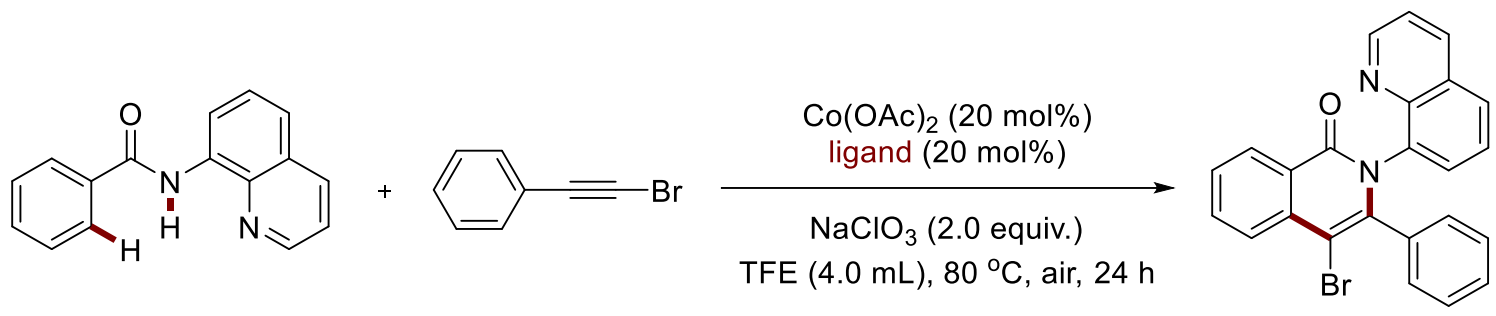

1a 2a 3

Entry	Ligand	Yield (%)
7	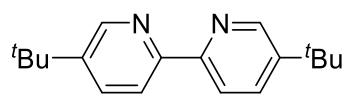	35
8	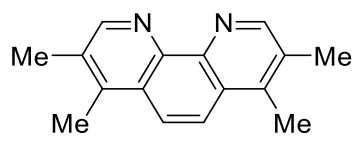	30
9	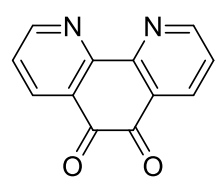	40
**10**	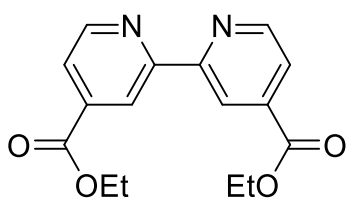	**50**
11	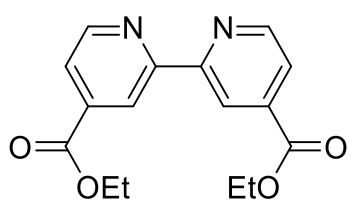 (40 mol%)	43

Table S6. Reaction conditions: **1a** (0.30 mmol), **2a** (0.45 mmol), $Co(OAc)_2$ (0.06 mmol), ligand (0.06 mmol), TFE (4.0 mL), $NaClO_3$, 80 °C, 24 h, under air. The yield was determined by isolated yields.

4.6. Screening of Additive

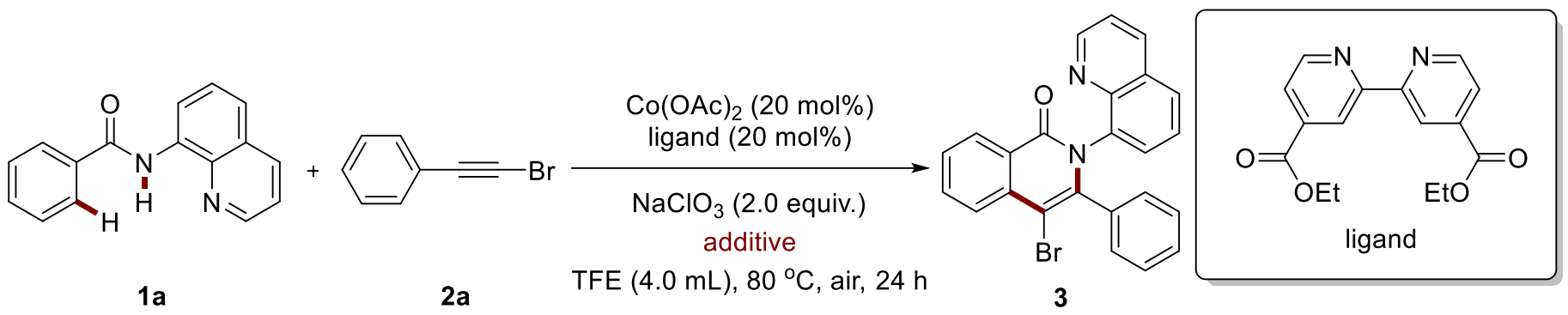

1a 2a 3

Entry	Additive	Yield (%)
1	Na_2CO_3 (3.0 eq)	NR
2	$CuSO_4$ (20 mol%)	55
3	$CuSO_4$ (20 mol%) without $Co(OAc)_2$	NR
4	CH_3COOH (2.0 eq) without ligand	30

Table S7. Reaction conditions: **1a** (0.30 mmol), **2a** (0.45 mmol), $Co(OAc)_2$ (0.06 mmol), ligand (0.06 mmol), $NaClO_3$ (0.60 mmol), TFE (4.0 mL), 80 °C, 24 h, under air. The yield was determined by isolated yields.

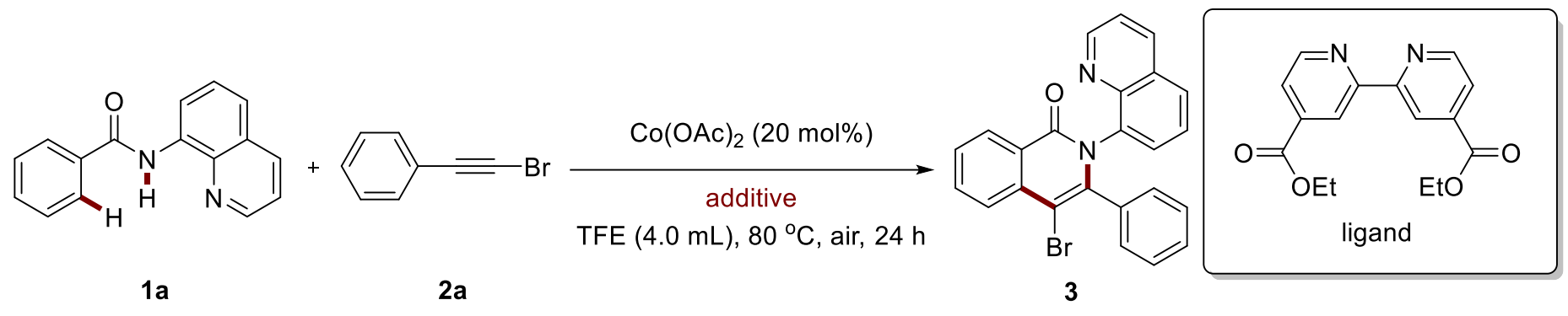

Entry	Additive[a]	Yield (%)
1	$CuSO_4$ (3.0), ligand (0.2), $NaClO_3$ (1.0)	35
2	$CuSO_4$ (1.0), ligand (0.2), $NaClO_3$ (2.0)	60
3	$CuSO_4$ (2.0), ligand (0.2), $NaClO_3$ (2.0)	55
**4**	**$CuSO_4$ (0.3), ligand (0.2), $NaClO_3$ (2.0)**	**63**
5	$CuSO_4$ (0.5), ligand (0.2), $NaClO_3$ (2.0)	60
6	$CuSO_4$ (0.3), ligand (0.5), $NaClO_3$ (2.0)	40

Table S8. [a]: All reagents were used in equivalents (equiv.). Reaction conditions: **1a** (0.30 mmol), **2a** (0.45 mmol), $Co(OAc)_2$ (0.06 mmol), TFE (4.0 mL), 80 °C, 24 h, under air. The yield was determined by isolated yields.

4.7. Screening of Co-oxidant

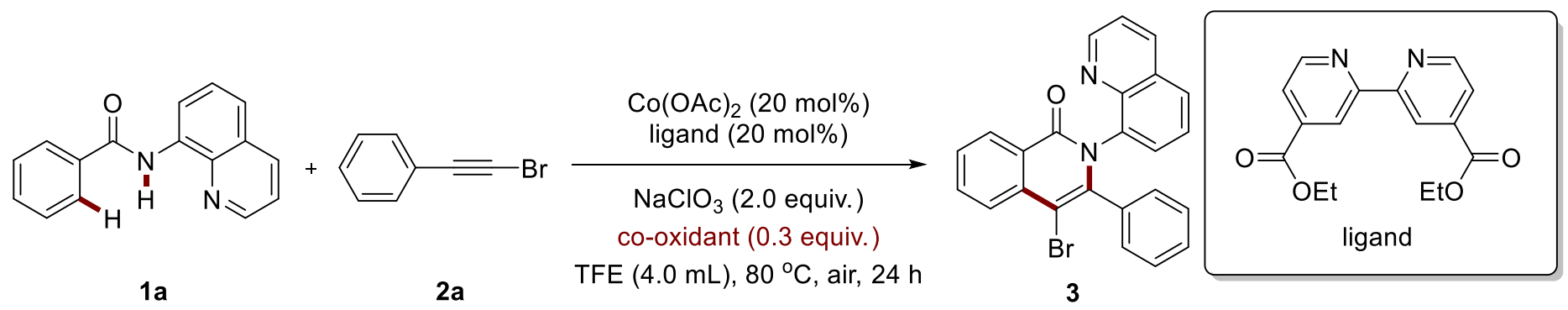

Entry	Co-oxidant	Yield (%)
1	$Cu(OAc)_2$	30
2	$Cu(acac)_2$	40
3	$CuBr_2$	40
4	$Cu(CF_3SO_3)_2$	30
**5**	**$CuSO_4$**	**63**

Table S9. Reaction conditions: **1a** (0.30 mmol), **2a** (0.45 mmol), $Co(OAc)_2$ (0.06 mmol), ligand (0.06 mmol), co-oxidant (0.09 mmol), $NaClO_3$ (0.60 mmol), TFE (4.0 mL), 80 °C, 24 h, under air. The yield was determined by isolated yields.

4.8. Screening of Time

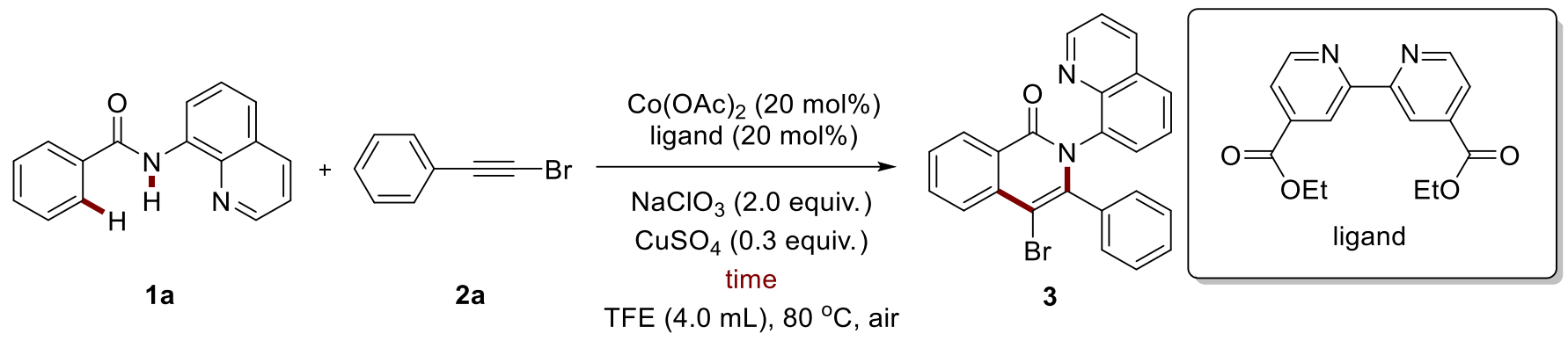

Entry	Time (h)	Yield (%)
1	10	63
2	5	63
**3**	**3**	**63**
4	1	20

Table S10. Reaction conditions: **1a** (0.30 mmol), **2a** (0.45 mmol), $Co(OAc)_2$ (0.06 mmol), ligand (0.06 mmol), $CuSO_4$ (0.09 mmol), $NaClO_3$ (0.60 mmol), TFE (4.0 mL), 80 °C, time (h), under air. The yield was determined by isolated yields.

4.9. Screening of Atmosphere

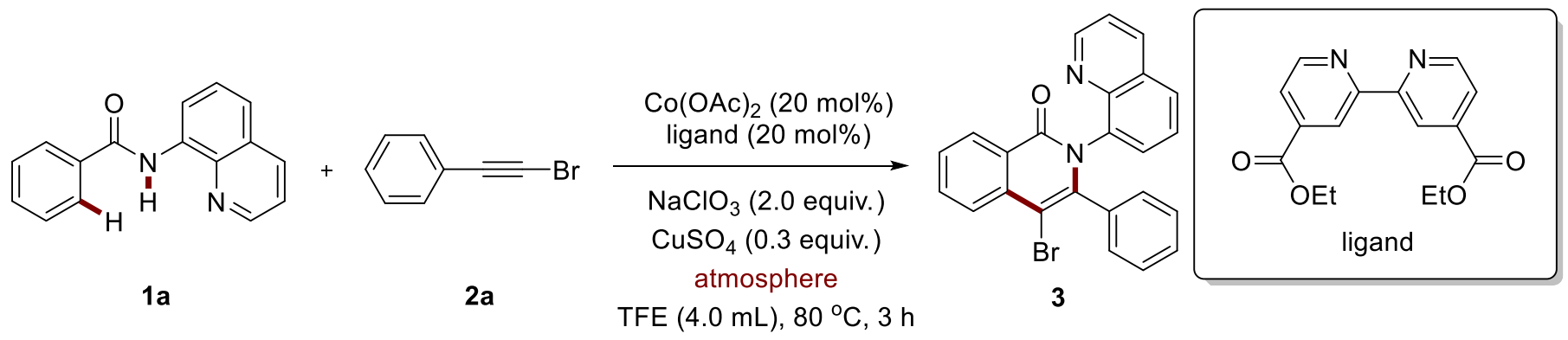

Entry	Atmosphere	Yield (%)
1	N_2	Trace
2	O_2	40
**3**	Air	**63**

Table S11. Reaction conditions: **1a** (0.30 mmol), **2a** (0.45 mmol), $Co(OAc)_2$ (0.06 mmol), ligand (0.06 mmol), $CuSO_4$ (0.09 mmol), $NaClO_3$ (0.60 mmol), TFE (4.0 mL), 80 °C, 3 h, atmosphere. The yield was determined by isolated yields.

4.10. Screening of Temperature

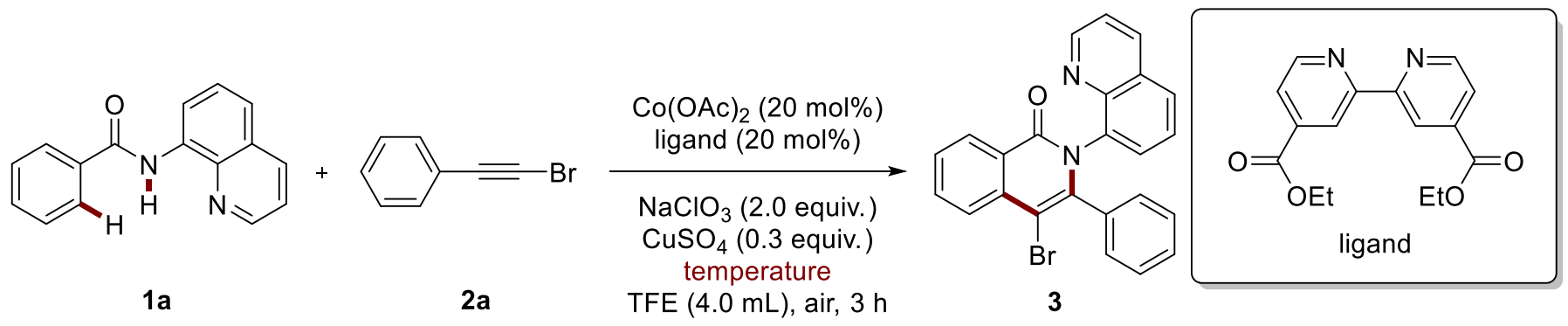

Entry	Temperature	Yield (%)
1	60	40
**2**	**80**	**63**
3	100	63

Table S12. Reaction conditions: **1a** (0.30 mmol), **2a** (0.45 mmol), $Co(OAc)_2$ (0.06 mmol), ligand (0.06 mmol), $CuSO_4$ (0.09 mmol), $NaClO_3$ (0.60 mmol), TFE (4.0 mL), temperature °C, 3 h, under air. The yield was determined by isolated yields.

4.11. The role of Oxygen

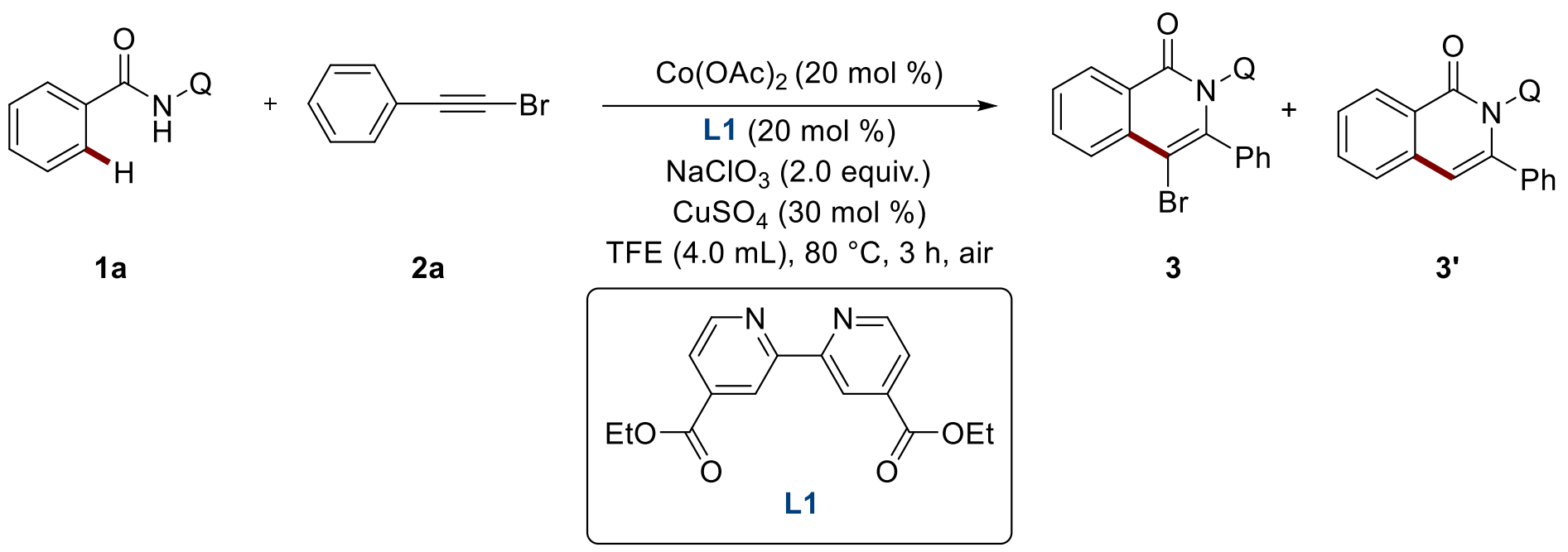

Entry	Variation	Yield of 3 (3') (%)
1	none	63 (25)
2	used O_2 instead of air	40 (42)
3	used O_2 instead of air and $NaClO_3$	8 (10)
4	used N_2 instead of air	trace (trace)

Table S13. Reaction conditions: **1a** (0.30 mmol), **2a** (0.45 mmol), $Co(OAc)_2$ (0.06 mmol), ligand (0.06 mmol), $CuSO_4$ (0.09 mmol), $NaClO_3$ (if required, 0.60 mmol), TFE (4.0 mL), temperature °C, 3 h, under air. The yield was determined by isolated yields.

The control experiments confirming that an appropriate amount of O_2 are essential for oxidizing Co(II) to the catalytically active Co(III) species required for C–H activation and $NaClO_3$ may play the role as terminal oxidant to regenerate the active Co(II) species.

5. Origin of Debromination

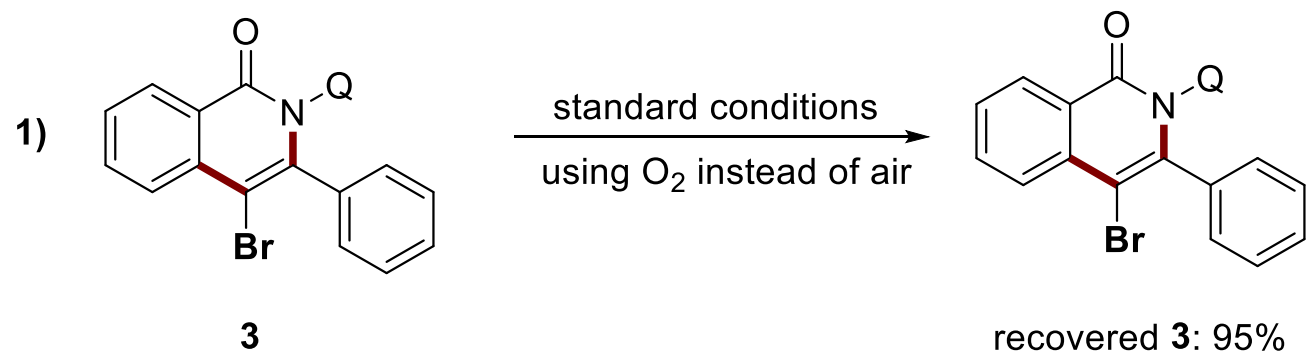

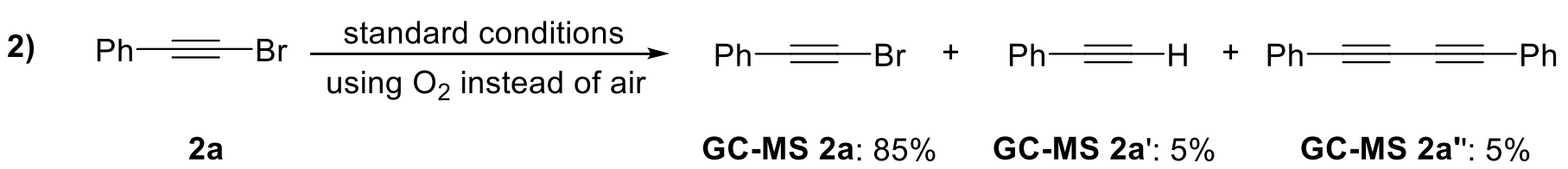

Control experiments further showed that both the product and the bromoalkyne are stable under O_2, excluding simple oxidative decomposition. Thus, the debromination observed under aerobic conditions is attributed to β-bromide elimination during the catalytic cycle, while the debrominated byproduct likely arises from cyclization of the alkynylation intermediate.[4, 5]

6. HUH-7 Cell Experiments

6.1. Cell Culture

1. Retrieve the cryovial from liquid nitrogen and immediately place it in a 37°C water bath. Gently swirl the vial until the cryopreservation medium is fully thawed.
2. Transfer the cell suspension into a centrifuge tube containing 5 mL of pre-warmed culture medium. Centrifuge at 1000 rpm for 5 min at room temperature to pellet the cells, then carefully discard the supernatant.
3. Resuspend the cell pellet in complete medium supplemented with 10% fetal bovine serum (FBS). Seed the cells into a culture dish, mix gently by pipetting, and incubate at 37°C in a humidified atmosphere with 5% CO_2.
4. Harvest cells in the logarithmic growth phase that exhibit robust viability, and seed them into a 96-well plate at a density of 4×10^3 cells per well. Incubate the plate overnight at 37°C with 5% CO_2. (Add 100 μL of sterile PBS to the perimeter wells to minimize evaporation edge effects.)

6.2. Material Treatment

1. The samples were sterilized by high-temperature and high-pressure steam autoclaving. Following sterilization, the samples were prepared at concentrations of 100 μM, 50 μM, 25 μM, 5 μM, and 1 μM.
2. A 96-well plate seeded with cells the previous day was used. The samples at various concentrations were added to the wells for co-culture with the cells.
3. One time point was set for the experimental group: 24 hours.

The experiment consisted of three groups: Experimental Group (cells + culture medium + drug), Control Group (cells + culture medium, no drug), Blank Control Group (no cells, culture medium only, no drug).

The plate was then placed back into the cell culture incubator and incubated for the designated time period before subsequent analysis.

6.3. CCK-8 Assay

After incubating the 96-well plate for the designated period, 10 μL of CCK-8 reagent was added to each well under light-protected conditions. The plate was then incubated for 3 hours, after which the absorbance at 450 nm was measured using a microplate reader. Cell viability was calculated based on the obtained absorbance values.

6.4. Analysis of Experimental Results

The absorbance values for each group were entered into Excel, and the relative viability was calculated using the following formula: Relative Cell Viability (%) = (ODexperimental − ODbackground) / (Mean ODcontrol − ODbackground) × 100.

In this formula, the background absorbance (OD_{background}) refers to the optical density of the culture medium alone, measured from wells containing only medium (no cells). The results are shown in Table S13.

Compound ID	Structure	IC$_{50}$ against Huh-7 cells[a] (Camptothecin) (μmol)
49	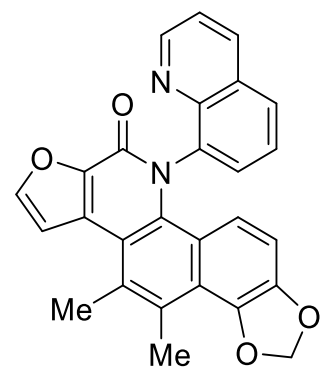	39.39 (14.48)
50	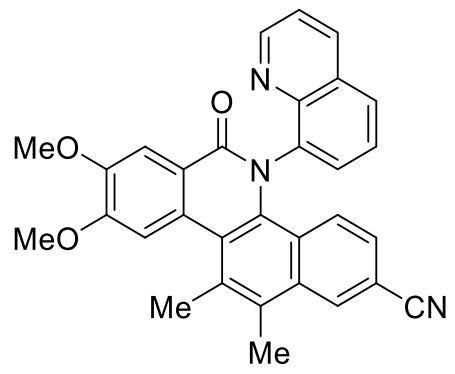	319.1 (14.48)
51	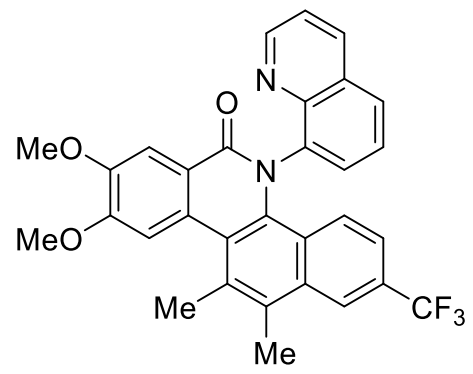	108.9 (14.48)
52	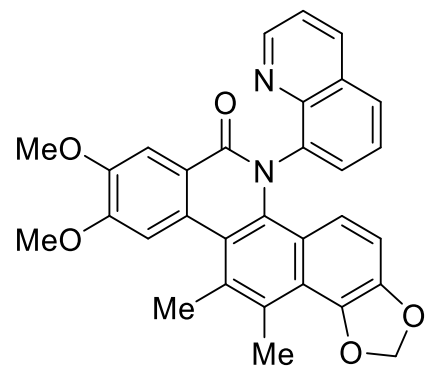	67.51 (14.48)
53	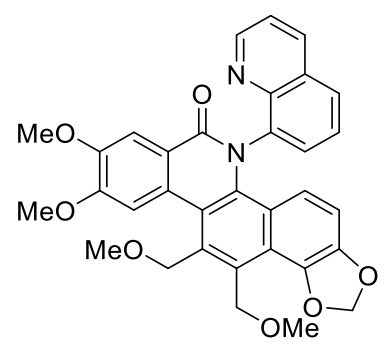	125.6 (14.48)
54	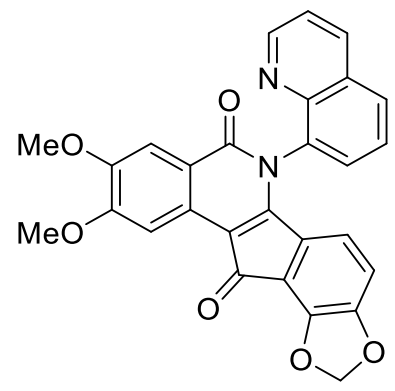	296 (14.48)

Table S14. [a] The half-maximal inhibitory concentration for cell viability (IC$_{50}$)

7. Mechanistic Research

7.1 Competition Experiments

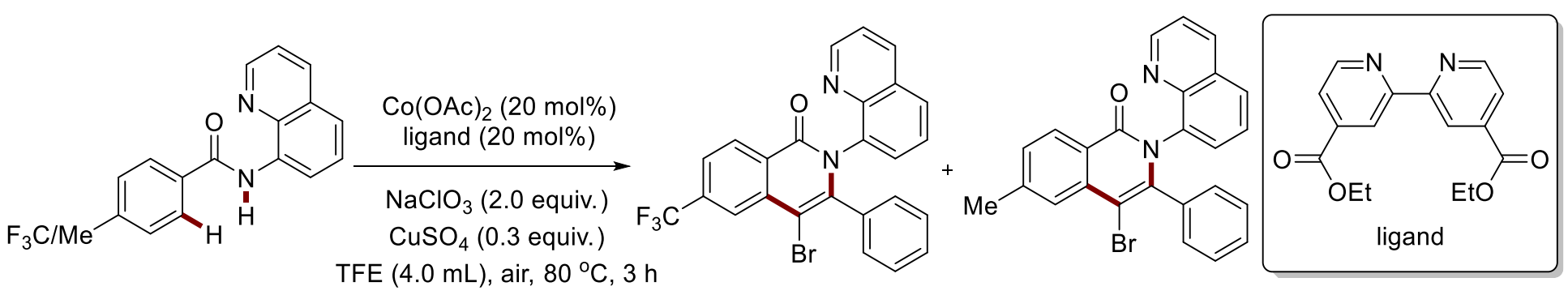

1d/1l **14** (24% Yield) **6** (53% Yield)

A 10 mL schlenk tube equipped with a magnetic stir bar was charged with **1d** (0.30 mmol) and **1l** (0.30 mmol), alkyne **2a** (0.45 mmol, 1.5 equiv), Co(OAc)$_2$ (0.06 mmol,

20 mol%), $CuSO_4$ (0.09 mmol, 30 mol%), $NaClO_3$ (0.60 mmol, 2.0 equiv.), ligand (0.60 mmol, 20 mol%) and TFE (4.0 mL). The mixture was reacted for 3 hours under air at 80 °C. After completion of the reaction, the solvent was then removed under vacuum and the residue was purified by column chromatography on silica gel to afford the corresponding products **14** (35,7 mg, 24%) and **6** (70.2 mg, 53%). The ratio between **14** and **6** was 1.00/2.21.

7.2.1 Kinetic Isotope Effect (KIE) Experiments

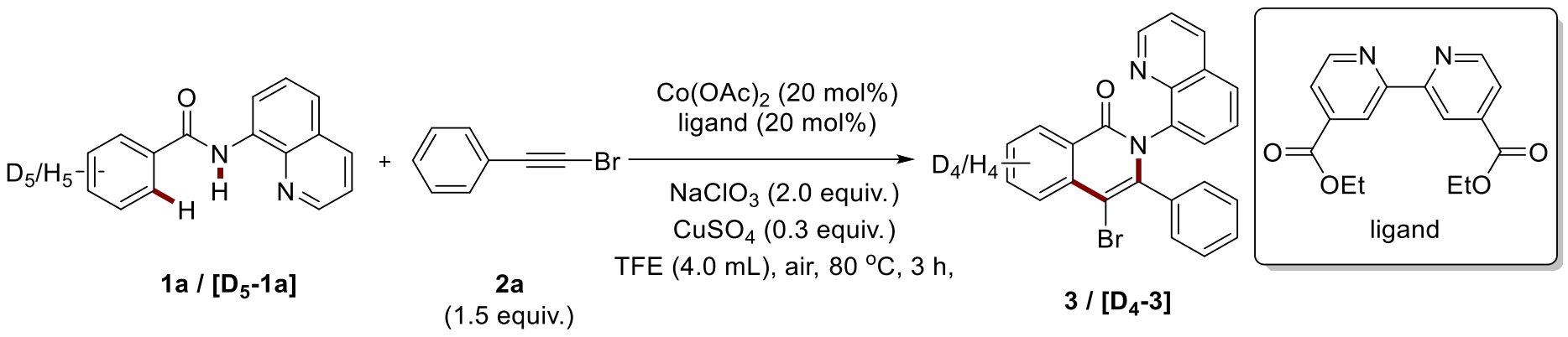

The model reaction using substrates **1a** (0.30 mmol) or substrates **[D$_5$]-1a** (0.30 mmol) were carried out to determine the KIE by comparison of the initial rates. The yields of the products were determined by ^{1}H NMR measurement using anisole (0.10 mmol) as the internal standard.

Table S15

Time/min	20	30	40	50	60	90
**3**/%	5.00	7.67	13.33	14.00	17.00	27.00

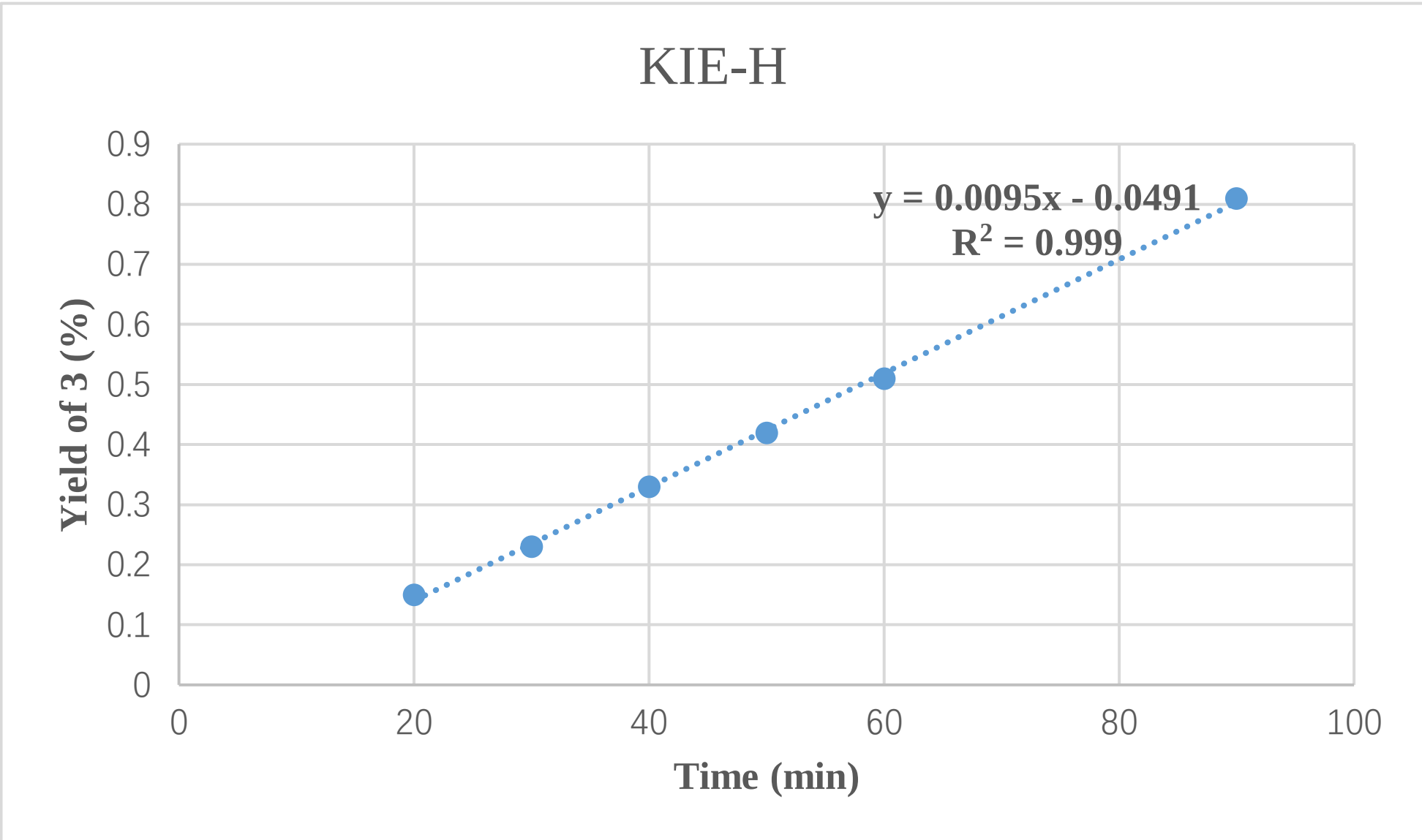

Figure S1. Linear function fit for the reaction rate of **3**.

Table S16

Time/min	20	30	40	50	60	90
[D$_4$]-**3**/%	4.33	7.00	9.33	12.00	14.67	22.00

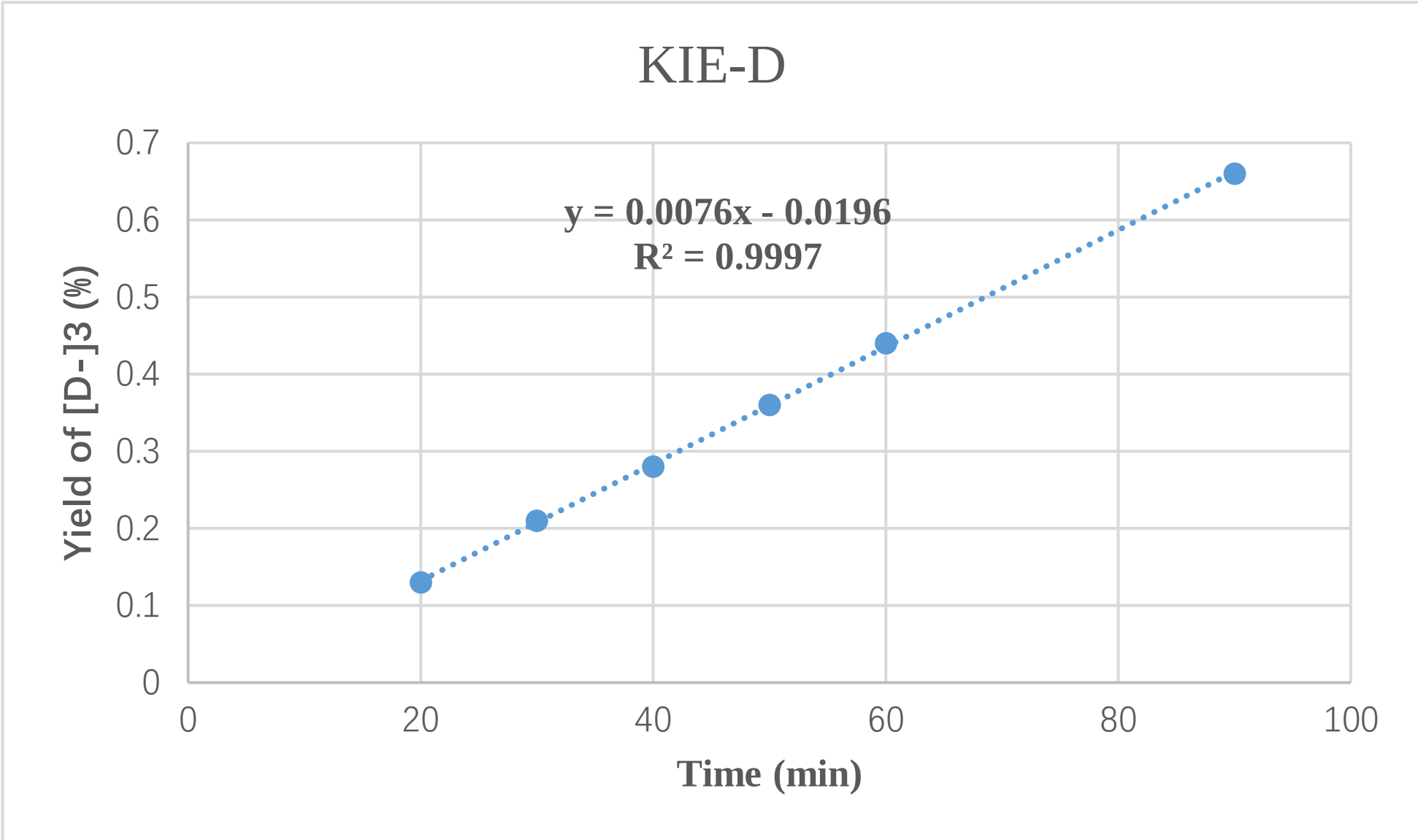

Figure S2. Linear function fit for the reaction rate of **[D$_4$]-3**.

The intermolecular isotope effect value is determined to be 1.25.
$k_H/k_D = (0.0095)/(0.0076) = 1.25$

7.2.2 Competitive KIE Experiment

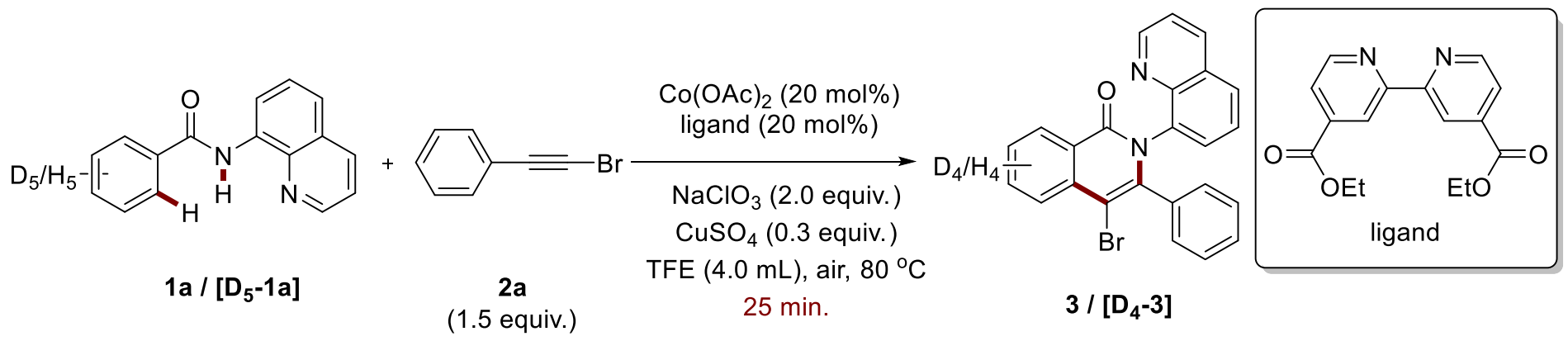

The Model reactions were performed with a mixture of substrate **1a** (0.15 mmol) and deuterated substrate [D$_5$]-**1a** (0.15 mmol). The kinetic isotope effect (KIE) was determined based on the product ratio of the two corresponding products, which was quantified via HPLC-MS analysis. All measurements were conducted in triplicate to afford the averaged value.

	Run 1	***Run 2***	***Run 3***
	k_H/k_{D1}	k_H/k_{D2}	k_H/k_{D3}
	1.14	1.10	1.12
Average	1.12		

The competitive KIE value was determined to be 1.12.

8. Mass Balance Analysis

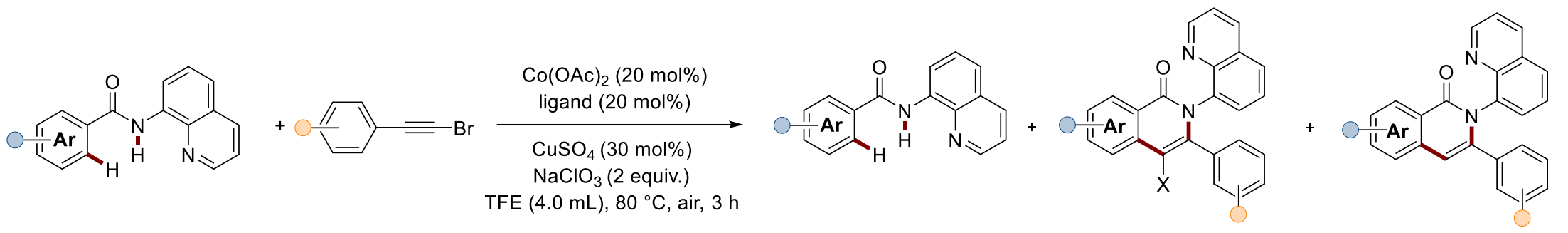

Benzamide	Bromoalkyne	Recovered Benzamide (%)	Product (%)	Debrominated Product
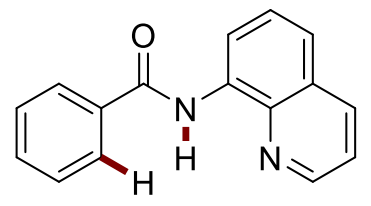 **1a**	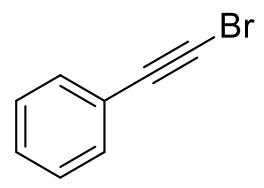 **2a**	-	63%	25%
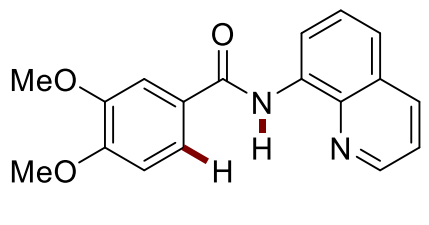 **1r**	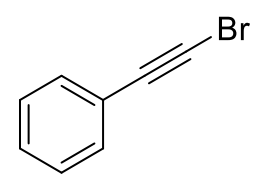 **2a**	-	40%	43%
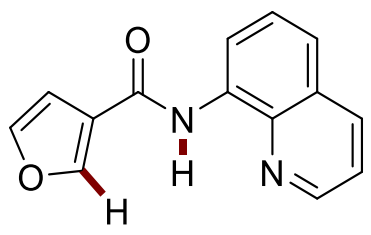 **1u**	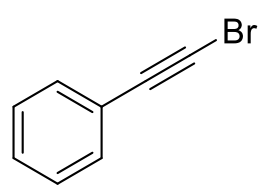 **2a**	-	40%	35%
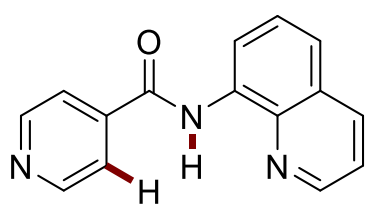 **1v**	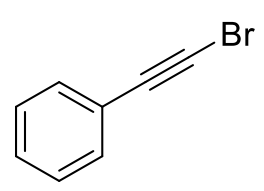 **2a**	17%	25%	28%
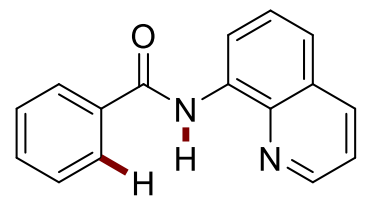 **1a**	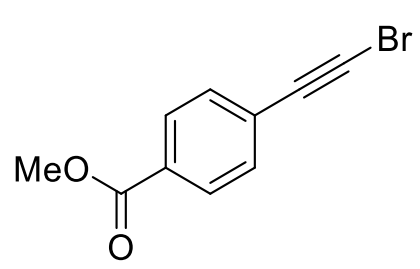 **2m**	-	74%	10%
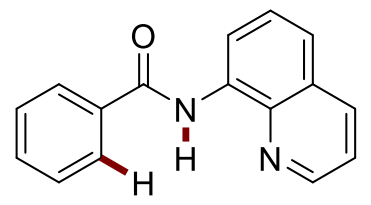 **1a**	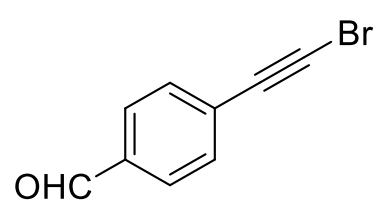 **2o**	-	77%	10%

The major by-product of the reaction is debrominative annulation product and a range of minor unknow by-product account for the loss of mass balance.

9. Characterization Data

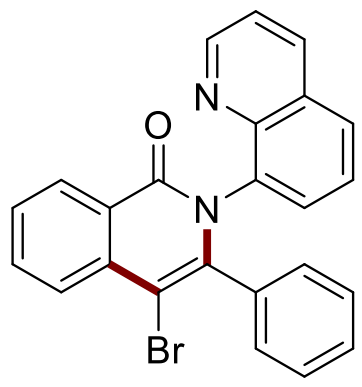

4-Bromo-3-phenyl-2-(quinolin-8-yl)isoquinolin-1(2*H*)-one (3)
Prepared according to the general procedure **A** using **1a** (74.4 mg, 0.30 mmol) and **2a** (81.0 mg, 0.45 mmol), purification by column chromatography on silica gel (petroleum ether/EtOAc/DCM = 2:1:1) yielded **3** (80.7 mg, 63%) as a colorless solid.

^{1}H NMR (600 MHz, $CDCl_3$): δ = 8.90 (d, J = 3.6 Hz, 1H), 8.53 (d, J = 7.9 Hz, 1H), 8.12 (d, J = 8.2 Hz, 1H), 8.06 (d, J = 8.2, 1H), 7.83 (t, J = 7.7 Hz, 1H), 7.67 (d, J = 8.2 Hz, 1H), 7.60 (t, J = 7.6 Hz, 1H), 7.47 (d, J = 7.3, 1H), 7.40–7.35 (m, 2H), 7.24 (d, J = 7.6 Hz, 1H), 7.16 (t, J = 7.6 Hz, 1H), 7.01 (t, J = 7.5, 1H), 6.91 (d, J = 7.8 Hz, 1H), 6.77 (t, J = 7.6 Hz, 1H).

^{13}C NMR (150 MHz, $CDCl_3$): δ = 162.4, 151.0, 144.5, 143.0, 137.5, 136.4, 136.2, 136.1, 133.6, 130.9, 130.1, 129.0, 129.0, 128.9, 128.8, 128.5, 127.8, 127.5, 127.3, 126.9, 126.0, 125.8, 121.8, 102.3.

HR-MS (ESI): *m/z* calcd for $C_{24}H_{15}BrN_2O$ $[M+Na]^+$ 449.0261, 451.0240, found 449.0260, 451.0239.

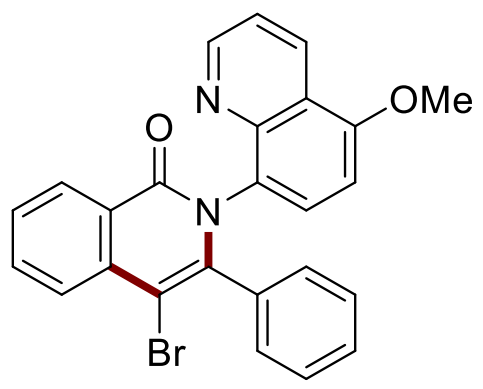

4-Bromo-2-(5-methoxyquinolin-8-yl)-3-phenylisoquinolin-1(2*H*)-one (4)
Prepared according to the general procedure **A** using **1b** (84.0 mg, 0.30 mmol) and **2a** (81.0 mg, 0.45 mmol), purification by column chromatography on silica gel (petroleum ether/EtOAc/DCM = 1:1:1) yielded **4** (89.1 mg, 65%) as a colorless solid.

^{1}H NMR (600 MHz, $CDCl_3$): δ = 8.87 (dd, J = 4.1, 1.8 Hz, 1H), 8.52 (dd, J = 8.0, 1.4 Hz, 1H), 8.45 (dd, J = 8.4, 1.8 Hz, 1H), 8.11 (d, J = 8.3 Hz, 1H), 7.81 (ddd, J = 8.3, 7.2, 1.4 Hz, 1H), 7.59 (t, J = 7.6 Hz, 1H), 7.38–7.32 (m, 2H), 7.24 (d, J = 7.9 Hz, 1H), 7.18 (t, J = 7.5 Hz, 1H), 7.04 (t, J = 7.4 Hz, 1H), 6.95 (d, J = 7.6 Hz, 1H), 6.80 (t, J = 7.6 Hz, 1H), 6.66 (d, J = 8.2 Hz, 1H), 3.92 (s, 3H).

^{13}C NMR (150 MHz, $CDCl_3$): δ = 162.7, 155.4, 151.1, 144.8, 143.5, 136.5, 133.5, 131.0, 130.8, 130.1, 130.0, 128.9, 128.9, 128.4, 127.7, 127.5, 127.4, 126.8, 126.0, 121.1, 120.8, 103.4, 102.1, 55.9.

HR-MS (ESI): *m/z* calcd for $C_{25}H_{17}BrN_2O_2$ $[M+Na]^+$ 479.0366, 481.0346, found 479.0364, 481.0346.

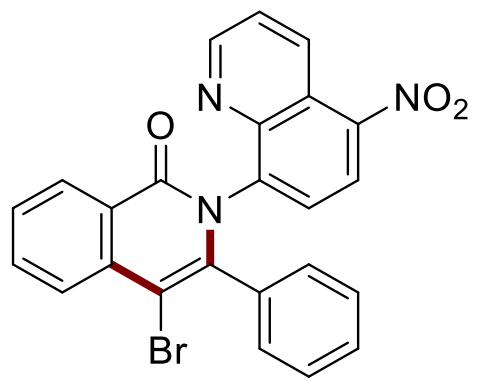

4-Bromo-2-(5-nitroquinolin-8-yl)-3-phenylisoquinolin-1(2*H*)-one (5)
Prepared according to the general procedure **A** using **1c** (88.0 mg, 0.30 mmol) and **2a** (81.0 mg, 0.45 mmol), purification by column chromatography on silica gel (petroleum ether/EtOAc/DCM = 1:1:1) yielded **5** (77.9 mg, 55%) as a pale yellow solid.

^{1}H NMR (600 MHz, $CDCl_3$): δ = 8.99 (dd, *J* = 4.1, 1.6 Hz, 1H), 8.92 (dd, *J* = 8.8, 1.6 Hz, 1H), 8.49 (dd, *J* = 8.0, 1.4 Hz, 1H), 8.22 (d, *J* = 8.2 Hz, 1H), 8.14 (d, *J* = 7.9 Hz, 1H), 7.87 (ddd, *J* = 8.2, 7.2, 1.4 Hz, 1H), 7.68–7.61 (m, 2H), 7.59 (d, *J* = 8.2 Hz, 1H), 7.25 (d, *J* = 6.8 Hz, 1H), 7.21 (td, *J* = 7.6, 1.3 Hz, 1H), 7.08 (t, *J* = 7.4 Hz, 1H), 6.87 (d, *J* = 7.9 Hz, 1H), 6.82 (td, *J* = 7.6, 1.3 Hz, 1H).

^{13}C NMR (150 MHz, $CDCl_3$): δ = 162.2, 152.0, 145.3, 144.5, 143.6, 141.9, 136.3, 135.5, 134.0, 132.3, 130.0, 129.5, 129.1, 129.0, 128.7, 128.2, 128.0, 127.7, 127.1, 125.7, 124.5, 124.1, 121.9, 102.9.

HR-MS (ESI): *m/z* calcd for $C_{24}H_{14}BrN_3O_3$ $[M+Na]^+$ 494.0111, 496.0091, found 494.0109, 496.0092.

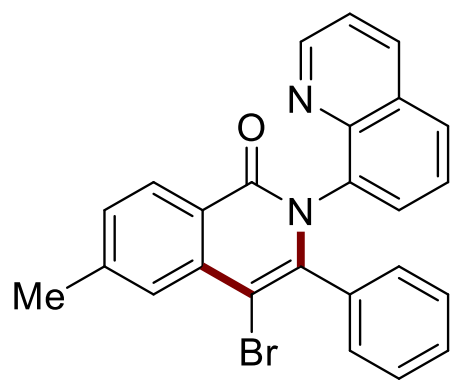

4-Bromo-6-methyl-3-phenyl-2-(quinolin-8-yl)isoquinolin-1(2*H*)-one (6)
Prepared according to the general procedure **A** using **1d** (78.6 mg, 0.30 mmol) and **2a** (81.0 mg, 0.45 mmol), purification by column chromatography on silica gel (petroleum ether/EtOAc/DCM = 3:1:1) yielded **6** (80.7 mg, 61%) as a colorless solid.

^{1}H NMR (600 MHz, $CDCl_3$): δ = 8.89 (dd, *J* = 4.2, 1.7 Hz, 1H), 8.41 (d, *J* = 8.2 Hz, 1H), 8.05 (dd, *J* = 8.3, 1.7 Hz, 1H), 7.90 (s, 1H), 7.66 (dd, *J* = 8.2, 1.4 Hz, 1H), 7.46 (dd, *J* = 7.3, 1.4 Hz, 1H), 7.41 (dd, *J* = 8.2, 1.6 Hz, 1H), 7.40–7.34 (m, 2H), 7.24 (d, *J* = 7.8 Hz, 1H), 7.15 (t, *J* = 7.6 Hz, 1H), 7.01 (t, *J* = 7.5 Hz, 1H), 6.90 (d, *J* = 7.8 Hz, 1H), 6.76 (t, *J* = 7.6 Hz, 1H), 2.59 (s, 3H).

^{13}C NMR (150 MHz, $CDCl_3$): δ = 162.4, 151.0, 144.5, 144.4, 142.9, 137.6, 136.4, 136.3,

136.2, 130.9, 130.1, 129.3, 129.0, 128.9, 128.8, 128.4, 127.5, 127.3, 126.7, 125.8, 123.8, 121.7, 102.2, 22.3.

HR-MS (ESI): *m/z* calcd for $C_{25}H_{17}BrN_2O$ $[M+H]^+$ 441.0597, 443.0577, found 441.0598, 443.0579.

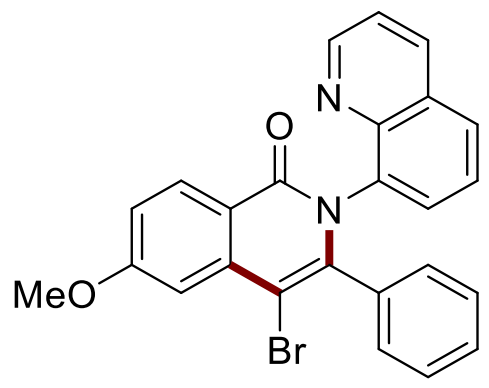

4-Bromo-6-methoxy-3-phenyl-2-(quinolin-8-yl)isoquinolin-1(2*H*)-one (7)
Prepared according to the general procedure **A** using **1e** (83.4 mg, 0.30 mmol) and **2a** (81.0 mg, 0.45 mmol), purification by column chromatography on silica gel (petroleum ether/EtOAc/DCM = 2:1:1) yielded **7** (91.9 mg, 67%) as a colorless solid.

^{1}H NMR (600 MHz, $CDCl_3$): δ = 8.90 (dd, J = 4.2, 1.7 Hz, 1H), 8.43 (d, J = 8.3 Hz, 1H), 8.05 (dd, J = 8.3, 1.7 Hz, 1H), 7.66 (dd, J = 8.3, 2.5 Hz, 1H), 7.49 (d, J = 2.5 Hz, 1H), 7.46 (dd, J = 7.2, 1.4 Hz, 1H), 7.39–7.35 (m, 2H), 7.23 (d, J = 7.8 Hz, 1H), 7.18–7.12 (m, 2H), 7.01 (tt, J = 7.5, 1.4 Hz, 1H), 6.90 (dt, J = 7.8, 1.5 Hz, 1H), 6.76 (td, J = 7.6, 1.3 Hz, 1H), 4.00 (s, 3H).

^{13}C NMR (150 MHz, $CDCl_3$): δ = 164.0, 162.1, 151.0, 144.5, 143.7, 138.6, 137.6, 136.3, 136.1, 131.1, 131.0, 130.0, 129.0, 128.9, 128.8, 128.5, 127.5, 127.3, 125.8, 121.7, 119.7, 116.9, 108.5, 102.0, 55.8.

HR-MS (ESI): *m/z* calcd for $C_{25}H_{17}BrN_2O_2$ $[M+H]^+$ 457.0546, 459.0526, found 457.0548, 459.0526.

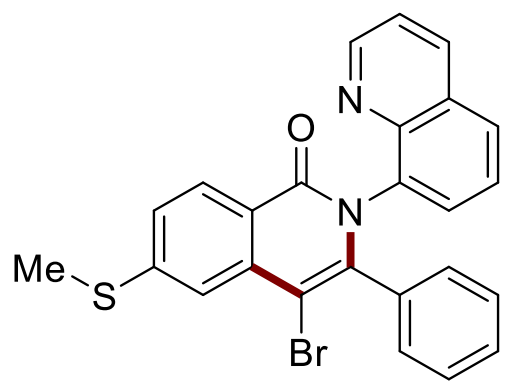

4-Bromo-6-(methylthio)-3-phenyl-2-(quinolin-8-yl)isoquinolin-1(2*H*)-one (8)
Prepared according to the general procedure **A** using **1f** (88.2 mg, 0.30 mmol) and **2a** (81.0 mg, 0.45 mmol), purification by column chromatography on silica gel (petroleum ether/EtOAc/DCM = 1:1:1) yielded **8** (63.9 mg, 45%) as a pale yellow solid.

^{1}H NMR (600 MHz, $CDCl_3$): δ = 8.90 (d, J = 4.1 Hz, 1H), 8.38 (d, J = 8.5 Hz, 1H), 8.05 (d, J = 8.2 Hz, 1H), 7.86 (s, 1H), 7.67 (d, J = 8.2 Hz, 1H), 7.46 (d, J = 7.2 Hz, 1H), 7.42 (d, J = 8.5 Hz, 1H), 7.40–7.34 (m, 2H), 7.24 (d, J = 7.6 Hz, 1H), 7.16 (t, J = 7.6 Hz, 1H), 7.01 (t, J = 7.4 Hz, 1H), 6.90 (d, J = 7.4 Hz, 1H), 6.77 (t, J = 7.6 Hz, 1H), 2.64 (s, 3H).

^{13}C NMR (150 MHz, $CDCl_3$): δ = 162.2, 151.0, 146.7, 144.4, 143.8, 137.5, 136.8, 136.2, 131.0, 130.0, 129.1, 129.0, 128.9, 128.8, 128.5, 127.6, 127.3, 125.8, 125.3, 122.7, 121.9, 121.8, 101.7, 15.1.

HR-MS (ESI): *m/z* calcd for $C_{25}H_{17}BrN_2OS$ $[M+H]^+$ 473.0318, 475.0298, found 473.0319, 475.0299.

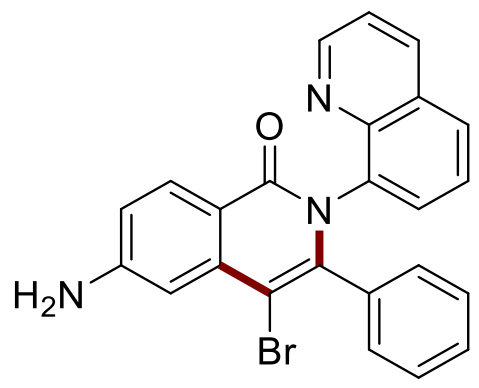

6-Amino-4-bromo-3-phenyl-2-(quinolin-8-yl)isoquinolin-1(2*H*)-one (9)
Prepared according to the general procedure **A** using **1g** (78.9 mg, 0.30 mmol) and **2a** (81.0 mg, 0.45 mmol), purification by column chromatography on silica gel (petroleum ether/EtOAc/DCM = 1:2:1) yielded **9** (49.1 mg, 37%) as a colorless solid.

^{1}H NMR (600 MHz, $CDCl_3$): δ = 8.91 (d, *J* = 3.8 Hz, 1H), 8.29 (d, *J* = 8.6 Hz, 1H), 8.07 (d, *J* = 7.1 Hz, 1H), 7.66 (d, *J* = 8.1 Hz, 1H), 7.46 (d, *J* = 7.3 Hz, 1H), 7.41–7.34 (m, 2H), 7.22 (d, *J* = 7.7 Hz, 1H), 7.20 (d, *J* = 2.2 Hz, 1H), 7.14 (t, *J* = 7.6 Hz, 1H), 6.99 (t, *J* = 7.6 Hz, 1H), 6.92 (d, *J* = 7.8 Hz, 1H), 6.85 (dd, *J* = 8.6, 2.2 Hz, 1H), 6.75 (t, *J* = 7.7 Hz, 1H).

^{13}C NMR (150 MHz, $CDCl_3$): δ = 162.2, 151.6, 150.8, 143.2, 138.4, 137.6, 136.5, 131.3, 131.0, 130.1, 128.9, 128.9, 128.4, 127.5, 127.3, 125.9, 121.7, 117.4, 116.2, 109.2, 102.0.

HR-MS (ESI): *m/z* calcd for $C_{24}H_{16}BrN_3O$ $[M+H]^+$ 442.0550, 444.0530, found 442.0551, 444.0530.

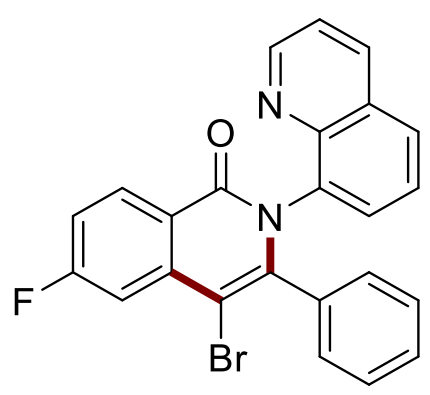

5-Bromo-6-fluoro-3-phenyl-2-(quinolin-8-yl)isoquinolin-1(2*H*)-one (10)
Prepared according to the general procedure **A** using **1h** (79.8 mg, 0.30 mmol) and **2a** (81.0 mg, 0.45 mmol), purification by column chromatography on silica gel (petroleum ether/EtOAc/DCM = 3:1:1) yielded **10** (72.1 mg, 54%) as a colorless solid.

^{1}H NMR (600 MHz, $CDCl_3$): δ = 8.90 (dd, *J* = 4.1, 1.7 Hz, 1H), 8.53 (dd, *J* = 8.8, 5.8 Hz, 1H), 8.06 (dd, *J* = 8.3, 1.7 Hz, 1H), 7.77 (dd, *J* = 10.3, 2.5 Hz, 1H), 7.68 (d, *J* = 8.2 Hz, 1H), 7.46 (d, *J* = 7.2 Hz, 1H), 7.43–7.33 (m, 2H), 7.28 (td, *J* = 8.5, 2.5 Hz, 1H), 7.23 (d, *J* = 7.6 Hz, 1H), 7.16 (t, *J* = 7.5 Hz, 1H), 7.02 (t, *J* = 7.4 Hz, 1H), 6.89 (d, *J* =

7.5 Hz, 1H), 6.77 (t, J = 7.6 Hz, 1H).

^{13}C NMR (150 MHz, $CDCl_3$): δ = 166.3 (d, $^1J_{C-F}$ = 253.3 Hz), 161.8, 151.1, 144.5, 144.3, 139.2, 139.1, 137.3, 136.2, 135.9, 132.3 (d, $^3J_{C-F}$ = 10.1 Hz), 130.9, 129.9, 129.2, 128.9, 128.7 (d, $^3J_{C-F}$ = 10.5 Hz), 127.6, 127.4, 125.9, 122.5 (d, $^4J_{C-F}$ = 1.6 Hz), 121.9, 116.3 (d, $^2J_{C-F}$ = 23.4 Hz), 112.6 (d, $^2J_{C-F}$ = 24.9 Hz), 101.2 (d, J = 3.1 Hz).

^{19}F NMR (565 MHz, $CDCl_3$): δ = -104.41.

HR-MS (ESI): m/z calcd for $C_{24}H_{14}BrFN_2O$ $[M+H]^+$ 445.0346, 447.0326, found 445.0346, 447.0325.

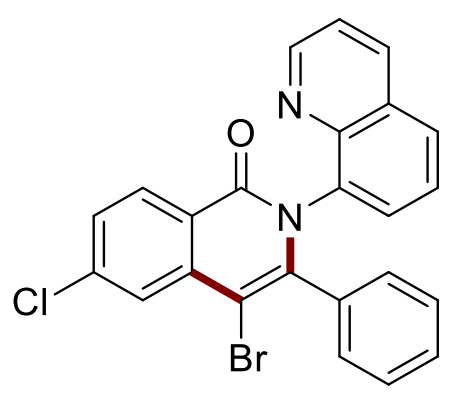

4-Bromo-6-chloro-3-phenyl-2-(quinolin-8-yl)isoquinolin-1(2*H*)-one (11)
Prepared according to the general procedure **A** using **1i** (84.6 mg, 0.30 mmol) and **2a** (81.0 mg, 0.45 mmol), purification by column chromatography on silica gel (petroleum ether/EtOAc/DCM = 3:1:1) yielded **11** (77.6 mg, 56%) as a colorless solid.

^{1}H NMR (600 MHz, $CDCl_3$): δ = 8.89 (dd, J = 4.2, 1.7 Hz, 1H), 8.45 (d, J = 8.5 Hz, 1H), 8.11 (d, J = 2.0 Hz, 1H), 8.06 (dd, J = 8.3, 1.7 Hz, 1H), 7.68 (dd, J = 8.2, 1.4 Hz, 1H), 7.53 (dd, J = 8.5, 2.0 Hz, 1H), 7.46 (dd, J = 7.3, 1.4 Hz, 1H), 7.41–7.35 (m, 2H), 7.23 (d, J = 7.9 Hz, 1H), 7.16 (t, J = 7.5 Hz, 1H), 7.02 (t, J = 7.5 Hz, 1H), 6.88 (d, J = 7.7 Hz, 1H), 6.77 (t, J = 8.0 Hz, 1H).

^{13}C NMR (150 MHz, $CDCl_3$): δ = 161.8, 151.1, 144.4, 144.3, 140.4, 137.8, 137.2, 136.2, 135.9, 130.8, 130.7, 129.9, 129.2, 128.8, 128.7, 128.7, 128.3, 127.6, 127.4, 126.5, 125.8, 124.3, 121.8, 100.9.

HR-MS (ESI): m/z calcd for $C_{24}H_{14}BrClN_2O$ $[M+H]^+$ 461.0051, 463.0031, found 461.0053, 463.0031.

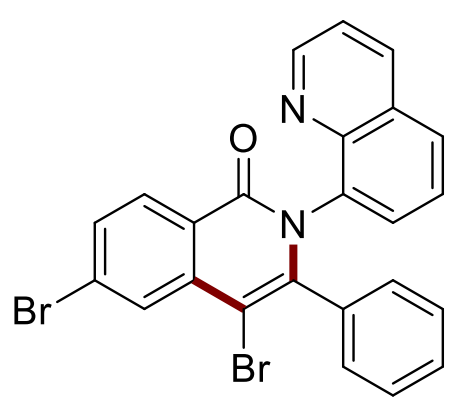

4,6-Dibromo-3-phenyl-2-(quinolin-8-yl)isoquinolin-1(2*H*)-one (12)
Prepared according to the general procedure **A** using **1j** (97.8 mg, 0.30 mmol) and **2a** (81.0 mg, 0.45 mmol), purification by column chromatography on silica gel (petroleum ether/EtOAc/DCM = 3:1:1) yielded **12** (82.0 mg, 54%) as a pale yellow solid.

^{1}H NMR (600 MHz, $CDCl_3$): δ = 8.87 (d, J = 4.3, 1.6 Hz, 1H), 8.36 (d, J = 8.5 Hz, 1H), 8.30 (s, 1H), 8.03 (d, J = 7.8 Hz, 1H), 7.70–7.64 (m, 2H), 7.46 (d, J = 7.0 Hz, 1H), 7.39–7.34 (m, 2H), 7.22 (d, J = 7.7 Hz, 1H), 7.15 (t, J = 7.6 Hz, 1H), 7.01 (t, J = 7.5 Hz, 1H), 6.91 (d, J = 7.7 Hz, 1H), 6.77 (t, J = 7.6 Hz, 1H).

^{13}C NMR (150 MHz, $CDCl_3$): δ = 162.0, 151.0, 144.7, 144.6, 138.1, 137.6, 136.1, 136.1, 131.1, 130.9, 130.7, 130.0, 129.7, 129.1, 129.1, 129.1, 129.0, 128.7, 127.6, 127.3, 125.8, 125.0, 121.8, 100.5.

HR-MS (ESI): *m/z* calcd for $C_{24}H_{14}Br_2N_2O$ $[M+H]^+$ 504.9546, 506.9526, found 504.9547, 506.9526.

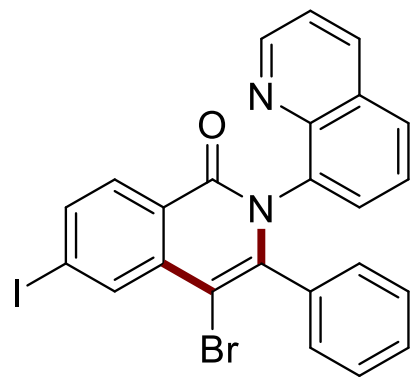

4-Bromo-6-iodo-3-phenyl-2-(quinolin-8-yl)isoquinolin-1(2*H*)-one (13)

Prepared according to the general procedure **A** using **1k** (112.2 mg, 0.30 mmol) and **2a** (81.0 mg, 0.45 mmol), purification by column chromatography on silica gel (petroleum ether/EtOAc/DCM = 3:1:1) yielded **13** (96.2 mg, 58%) as a pale yellow solid.

^{1}H NMR (600 MHz, $CDCl_3$): δ = 8.89 (dd, J = 4.3, 1.8 Hz, 1H), 8.50 (s, 1H), 8.18 (d, J = 8.4 Hz, 1H), 8.06 (d, J = 8.7 Hz, 1H), 7.90 (d, J = 8.4 Hz, 1H), 7.68 (d, J = 8.0 Hz, 1H), 7.45 (d, J = 7.0 Hz, 1H), 7.41–7.35 (m, 2H), 7.22 (d, J = 7.7 Hz, 1H), 7.16 (t, J = 7.6 Hz, 1H), 7.02 (t, J = 7.5 Hz, 1H), 6.87 (d, J = 7.7 Hz, 1H), 6.76 (t, J = 7.6 Hz, 1H).

^{13}C NMR (150 MHz, $CDCl_3$): δ = 162.2, 151.0, 144.5, 137.9, 137.5, 136.9, 136.2, 136.1, 135.9, 131.0, 130.3, 130.0, 129.1, 129.0, 128.7, 127.6, 127.4, 125.8, 125.5, 121.8, 101.6, 100.3.

HR-MS (ESI): *m/z* calcd for $C_{24}H_{14}BrIN_2O$ $[M+H]^+$ 552.9407, 554.9387, found 552.9408, 554.9386.

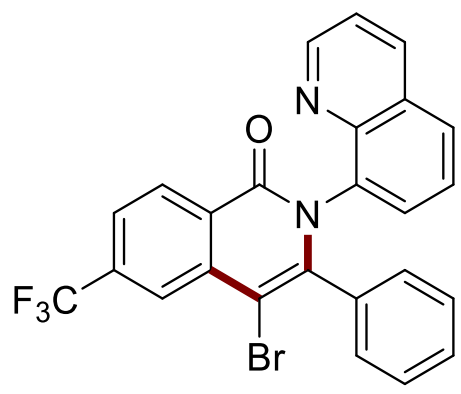

4-Bromo-3-phenyl-2-(quinolin-8-yl)-6-(trifluoromethyl)isoquinolin-1(2*H*)-one (14)

Prepared according to the general procedure **A** using **1l** (94.8 mg, 0.30 mmol) and **2a** (81.0 mg, 0.45 mmol), purification by column chromatography on silica gel (petroleum ether/EtOAc/DCM = 3:1:1) yielded **14** (98.0 mg, 66%) as a colorless solid.

^{1}H NMR (600 MHz, $CDCl_3$): δ = 8.89 (dd, J = 4.2, 1.7 Hz, 1H), 8.64 (d, J = 8.3 Hz, 1H), 8.40 (s, 1H), 8.07 (dd, J = 8.3, 1.7 Hz, 1H), 7.80 (dd, J = 8.3, 1.7 Hz, 1H), 7.69 (dd, J = 8.3, 1.4 Hz, 1H), 7.47 (dd, J = 7.3, 1.4 Hz, 1H), 7.43–7.35 (m, 2H), 7.24 (dt, J = 8.1, 1.6 Hz, 1H), 7.18 (td, J = 7.6, 1.3 Hz, 1H), 7.04 (tt, J = 7.5, 1.3 Hz, 1H), 6.89 (d, J = 7.9 Hz, 1H), 6.78 (td, J = 7.7, 1.3 Hz, 1H).

^{13}C NMR (150 MHz, $CDCl_3$): δ = 161.6, 151.1, 144.6, 144.2, 137.1, 136.8, 136.2, 135.7, 135.2 (q, ^{2}J = 32.6 Hz), 130.7, 130.0, 129.9, 129.3, 128.9, 128.8, 128.7, 128.2, 127.7, 127.4, 125.8, 124.5 (q, ^{3}J = 4.2 Hz), 123.8 (q, ^{1}J = 273.3 Hz), 123.8 (q, ^{3}J = 3.4 Hz), 121.9, 101.5.

^{19}F NMR (565 MHz, $CDCl_3$): δ = -62.90.

HR-MS (ESI): *m/z* calcd for $C_{25}H_{14}BrF_3N_2O$ $[M+H]^+$ 495.0314, 497.0294, found 495.0316, 497.0294.

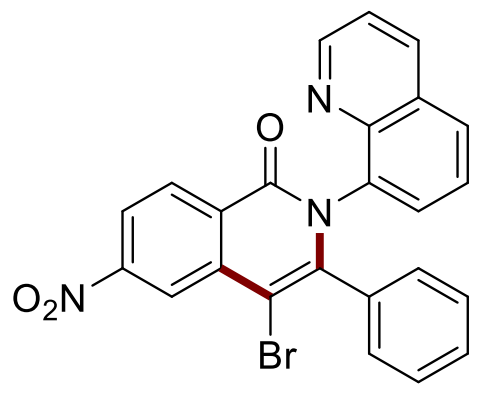

4-Bromo-6-nitro-3-phenyl-2-(quinolin-8-yl)isoquinolin-1(2*H*)-one (15)
Prepared according to the general procedure **A** using **1m** (87.9 mg, 0.30 mmol) and **2a** (81.0 mg, 0.45 mmol), purification by column chromatography on silica gel (petroleum ether/EtOAc/DCM = 1:1:1) yielded **15** (56.6 mg, 40%) as a pale yellow solid.

^{1}H NMR (400 MHz, d_6-DMSO): δ = 8.21 (dd, J = 4.3, 1.7 Hz, 1H), 8.12 (d, J = 2.3 Hz, 1H), 7.88 (d, J = 8.6 Hz, 1H), 7.73 (d, J = 8.6 Hz, 1H), 7.63 (d, J = 8.2 Hz, 1H), 7.19 (d, J = 8.2 Hz, 1H), 7.09 (d, J = 7.3 Hz, 1H), 6.85 (dd, J = 8.3, 4.3 Hz, 1H), 6.80 (t, J = 7.8 Hz, 1H), 6.73 (d, J = 7.7 Hz, 1H), 6.53 (t, J = 7.6 Hz, 1H), 6.38 (t, J = 7.6 Hz, 1H), 6.13 (dt, J = 13.8, 7.7 Hz, 2H).

^{13}C NMR (101 MHz, d_6-DMSO): δ = 159.8, 150.7, 150.7, 145.7, 143.2, 136.4, 136.1, 136.0, 134.9, 130.8, 130.4, 129.5, 128.9, 128.7, 128.5, 128.0, 127.7, 127.4, 126.8, 125.4, 121.7, 121.3, 121.3, 99.1.

HR-MS (ESI): *m/z* calcd for $C_{24}H_{14}BrN_3O_3$ $[M+H]^+$ 472.0291, 474.0271, found 472.0291, 474.0273.

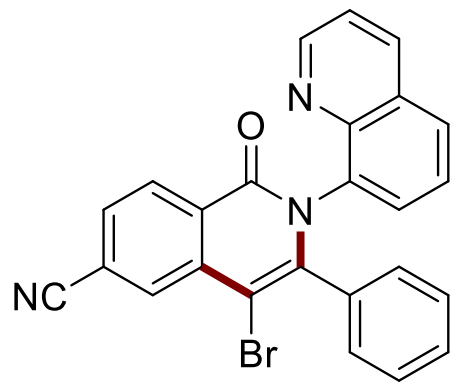

4-Bromo-1-oxo-3-phenyl-2-(quinolin-8-yl)-1,2-dihydroisoquinoline-6-carbonitrile (16)

Prepared according to the general procedure **A** using **1n** (81.9 mg, 0.30 mmol) and **2a** (81.0 mg, 0.45 mmol), purification by column chromatography on silica gel (petroleum ether/EtOAc/DCM = 2:1:1) yielded **16** (70.5 mg, 52%) as a colorless solid.

^{1}H NMR (600 MHz, $CDCl_3$): δ = 8.88 (d, J = 4.2 Hz, 1H), 8.60 (d, J = 8.2 Hz, 1H), 8.46 (s, 1H), 8.08 (d, J = 8.3 Hz, 1H), 7.78 (d, J = 8.2 Hz, 1H), 7.70 (d, J = 8.2 Hz, 1H), 7.46 (d, J = 7.2 Hz, 1H), 7.42–7.36 (m, 2H), 7.23 (d, J = 7.6 Hz, 1H), 7.18 (t, J = 7.5 Hz, 1H), 7.04 (t, J = 7.5 Hz, 1H), 6.87 (d, J = 7.8 Hz, 1H), 6.78 (t, J = 7.6 Hz, 1H).

^{13}C NMR (150 MHz, $CDCl_3$): δ = 161.3, 151.2, 145.3, 144.1, 136.9, 136.2, 135.5, 131.8, 130.6, 130.0, 129.8, 129.6, 129.4, 128.9, 128.9, 128.7, 128.4, 127.7, 127.5, 125.9, 122.0, 118.3, 117.2, 100.6.

HR-MS (ESI): *m/z* calcd for $C_{25}H_{14}BrN_3O$ $[M+Na]^+$ 474.0212, 476.0192, found 474.0214, 476.0194.

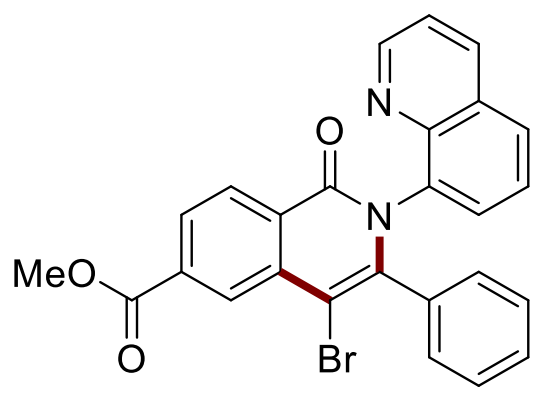

Methyl-4-bromo-1-oxo-3-phenyl-2-(quinolin-8-yl)-1,2-dihydroisoquinoline-6-carboxylate (17)

Prepared according to the general procedure **A** using **1o** (91.8 mg, 0.30 mmol) and **2a** (81.0 mg, 0.45 mmol), purification by column chromatography on silica gel (petroleum ether/EtOAc/DCM = 2:1:1) yielded **17** (72.8 mg, 50%) as a colorless solid.

^{1}H NMR (600 MHz, $CDCl_3$): δ = 8.91 (d, J = 4.0 Hz, 1H), 8.79 (d, J = 1.6 Hz, 1H), 8.57 (d, J = 8.2 Hz, 1H), 8.20 (dd, J = 8.2, 1.6 Hz, 1H), 8.10 (d, J = 8.1 Hz, 1H), 7.70 (d, J = 8.3 Hz, 1H), 7.48 (dd, J = 7.4, 1.3 Hz, 1H), 7.44–7.36 (m, 2H), 7.24 (d, J = 7.9 Hz, 1H), 7.17 (t, J = 7.6 Hz, 1H), 7.03 (t, J = 7.5 Hz, 1H), 6.93 (d, J = 7.6 Hz, 1H), 6.78 (t, J = 7.6 Hz, 1H), 4.03 (s, 3H).

^{13}C NMR (150 MHz, $CDCl_3$): δ = 166.5, 161.9, 150.9, 143.8, 137.0, 136.5, 135.9, 134.6, 131.1, 130.0, 129.3, 129.2, 128.9, 128.8, 128.8, 128.7, 127.9, 127.6, 127.5, 126.1, 121.9, 102.3, 52.8.

HR-MS (ESI): *m/z* calcd for $C_{26}H_{17}BrN_2O_3$ $[M+H]^+$ 485.0495, 487.0475, found 485.0496, 487.0476.

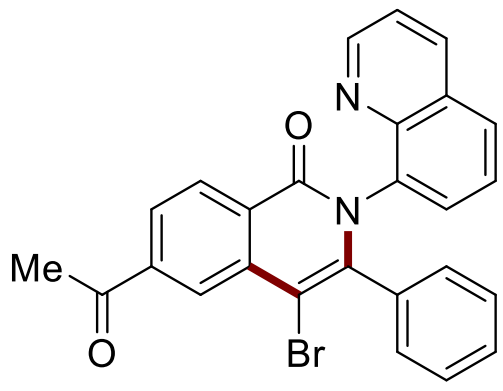

6-Acetyl-4-bromo-3-phenyl-2-(quinolin-8-yl)isoquinolin-1(2*H*)-one (18)
Prepared according to the general procedure **A** using **1p** (87.0 mg, 0.30 mmol) and **2a** (81.0 mg, 0.45 mmol), purification by column chromatography on silica gel (petroleum ether/EtOAc/DCM = 2:1:1) yielded **18** (77.4 mg, 55%) as a colorless solid.

^{1}H NMR (600 MHz, $CDCl_3$): δ = 8.89 (dd, J = 4.2, 1.6 Hz, 1H), 8.67 (d, J = 1.6 Hz, 1H), 8.59 (d, J = 8.3 Hz, 1H), 8.11 (dd, J = 8.3, 1.6 Hz, 1H), 8.07 (dd, J = 8.3, 1.6 Hz, 1H), 7.69 (dd, J = 8.2, 1.4 Hz, 1H), 7.47 (dd, J = 7.3, 1.4 Hz, 1H), 7.43–7.34 (m, 2H), 7.25 (d, J = 7.9 Hz, 1H), 7.17 (t, J = 7.8 Hz, 1H), 7.03 (t, J = 7.5 Hz, 1H), 6.90 (d, J = 8.2 Hz, 1H), 6.78 (td, J = 7.6, 1.3 Hz, 1H), 2.80 (s, 3H).

^{13}C NMR (150 MHz, $CDCl_3$): δ = 197.9, 161.8, 151.1, 144.2, 144.0, 140.8, 137.2, 136.6, 136.3, 135.8, 130.8, 130.0, 129.6, 129.2, 128.9, 128.8, 128.7, 127.6, 127.6, 127.4, 126.4, 125.9, 121.9, 102.2, 27.3.

HR-MS (ESI): *m/z* calcd for $C_{26}H_{17}BrN_2O_2$ $[M+H]^+$ 469.0546, 471.0526, found 469.0547, 471.0526.

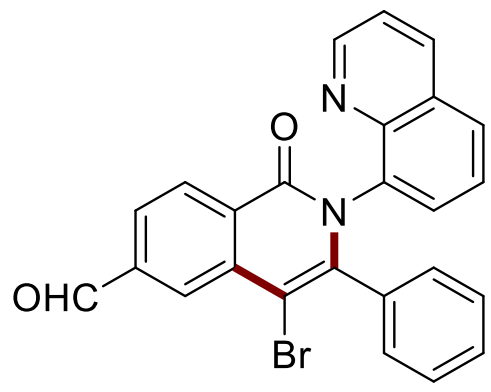

4-Bromo-1-oxo-3-phenyl-2-(quinolin-8-yl)-1,2-dihydroisoquinoline-6-carbaldehyde (19)
Prepared according to the general procedure **A** using **1q** (82.8 mg, 0.30 mmol) and **2a** (81.0 mg, 0.45 mmol), purification by column chromatography on silica gel (petroleum ether/EtOAc/DCM = 1:1:1) yielded **19** (62.8 mg, 46%) as a colorless solid.

^{1}H NMR (600 MHz, $CDCl_3$): δ = 10.28 (s, 1H), 8.89 (dd, J = 4.2, 1.7 Hz, 1H), 8.65 (d, J = 8.1 Hz, 1H), 8.60 (d, J = 1.5 Hz, 1H), 8.11–8.03 (m, 2H), 7.70 (dd, J = 8.3, 1.4 Hz, 1H), 7.48 (dd, J = 7.3, 1.4 Hz, 1H), 7.45–7.37 (m, 2H), 7.25 (d, J = 7.8 Hz, 1H), 7.18 (td, J = 7.6, 1.3 Hz, 1H), 7.04 (tt, J = 7.5, 1.3 Hz, 1H), 6.90 (dt, J = 7.5, 1.5 Hz, 1H), 6.78 (td, J = 7.6, 1.3 Hz, 1H).

^{13}C NMR (150 MHz, $CDCl_3$): δ = 192.0, 161.8, 151.1, 144.4, 144.2, 139.7, 137.1, 137.1, 136.2, 135.7, 130.7, 130.4, 130.0, 129.9, 129.6, 129.3, 128.9, 128.8, 128.8, 127.7, 127.4, 126.3, 125.9, 121.9, 101.8.

HR-MS (ESI): *m/z* calcd for $C_{25}H_{15}BrN_2O_2$ $[M+H]^+$ 455.0390, 457.0370, found 455.0390, 457.0371.

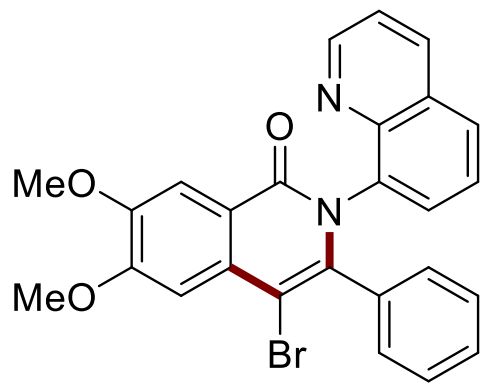

4-Bromo-6,7-dimethoxy-3-phenyl-2-(quinolin-8-yl)isoquinolin-1(2*H*)-one (20)
Prepared according to the general procedure **A** using **1r** (92.4 mg, 0.30 mmol) and **2a** (81.0 mg, 0.45 mmol), purification by column chromatography on silica gel (petroleum ether/EtOAc/DCM = 1:1:1) yielded **20** (58.4 mg, 40%) as a colorless solid.

^{1}H NMR (600 MHz, $CDCl_3$): δ = 8.90 (dd, *J* = 4.3, 1.7 Hz, 1H), 8.06 (dd, *J* = 8.3, 1.7 Hz, 1H), 7.92 (s, 1H), 7.66 (dd, *J* = 8.2, 1.4 Hz, 1H), 7.49 (s, 1H), 7.44 (dd, *J* = 7.3, 1.3 Hz, 1H), 7.40–7.33 (m, 2H), 7.25 (d, *J* = 7.1 Hz, 1H), 7.16 (t, *J* = 7.5 Hz, 1H), 7.01 (t, *J* = 7.3 Hz, 1H), 6.88 (d, *J* = 7.7 Hz, 1H), 6.76 (t, *J* = 7.6 Hz, 1H), 4.09 (s, 3H), 4.01 (s, 3H).

^{13}C NMR (150 MHz, $CDCl_3$): δ = 161.7, 154.3, 151.1, 149.8, 144.6, 141.5, 137.8, 136.3, 136.1, 131.9, 130.8, 130.3, 129.0, 128.9, 128.8, 128.4, 127.5, 127.3, 125.8, 121.7, 119.6, 108.8, 107.6, 101.9, 56.4, 56.4.

HR-MS (ESI): *m/z* calcd for $C_{26}H_{19}BrN_2O_3$ $[M+H]^+$ 487.0652, 489.0632, found 487.0653, 489.0633.

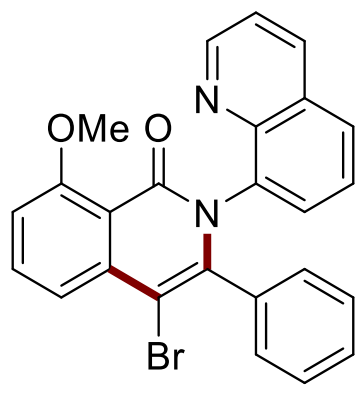

4-Bromo-8-methoxy-3-phenyl-2-(quinolin-8-yl)isoquinolin-1(2*H*)-one (21)
Prepared according to the general procedure **A** using **1s** (83.4 mg, 0.30 mmol) and **2a** (81.0 mg, 0.45 mmol), purification by column chromatography on silica gel (petroleum ether/EtOAc/DCM = 1:1:1) yielded **21** (76.8 mg, 56%) as a colorless solid.

^{1}H NMR (600 MHz, $CDCl_3$): δ = 8.89 (dd, *J* = 4.2, 1.8 Hz, 1H), 8.03 (d, *J* = 8.2 Hz, 1H), 7.74 (d, *J* = 8.0 Hz, 1H), 7.71 (t, *J* = 7.8 Hz, 1H), 7.63 (d, *J* = 8.1 Hz, 1H), 7.44 (d, *J* = 7.1 Hz, 1H), 7.38–7.32 (m, 2H), 7.22 (d, *J* = 7.6 Hz, 1H), 7.14 (t, *J* = 7.5 Hz, 1H), 7.03 (d, *J* = 8.0 Hz, 1H), 6.99 (t, *J* = 7.5 Hz, 1H), 6.94 (d, *J* = 7.6 Hz, 1H), 6.76 (t, *J* =

7.6 Hz, 1H), 3.95 (s, 3H).

^{13}C NMR (150 MHz, $CDCl_3$): δ = 161.7, 160.8, 150.9, 143.9, 139.2, 137.9, 136.5, 136.1, 134.1, 131.2, 129.9, 128.8, 128.8, 128.7, 128.4, 127.5, 127.2, 125.8, 121.6, 119.1, 115.4, 109.5, 101.6, 56.4.

HR-MS (ESI): *m/z* calcd for $C_{25}H_{17}BrN_2O_2$ $[M+H]^+$ 457.0546, 459.0526, found 457.0546, 459.0525.

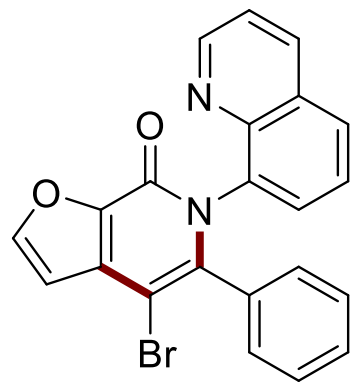

5-Bromo-5-phenyl-6-(quinolin-8-yl)furo[2,3-*c*]pyridin-7(6*H*)-one (22)
Prepared according to the general procedure **A** using **1t** (71.4 mg, 0.30 mmol) and **2a** (81.0 mg, 0.45 mmol), purification by column chromatography on silica gel (petroleum ether/EtOAc/DCM = 1:2:1) yielded **22** (65.1 mg, 52%) as a colorless solid.

^{1}H NMR (600 MHz, $CDCl_3$): δ = 8.90 (dd, *J* = 4.2, 1.6 Hz, 1H), 8.05 (dd, *J* = 8.4, 1.7 Hz, 1H), 7.86 (d, *J* = 2.0 Hz, 1H), 7.68 (dd, *J* = 8.2, 1.4 Hz, 1H), 7.46 (dd, *J* = 7.3, 1.4 Hz, 1H), 7.40–7.35 (m, 2H), 7.17 (d, *J* = 7.8 Hz, 1H), 7.13 (t, *J* = 7.5 Hz, 1H), 7.01 (tt, *J* = 7.5, 1.4 Hz, 1H), 6.90–6.83 (m, 2H), 6.76 (t, *J* = 7.6 Hz, 1H).

^{13}C NMR (150 MHz, $CDCl_3$): δ = 153.4, 151.1, 148.6, 144.3, 143.4, 142.1, 136.8, 136.2, 135.2, 135.0, 131.1, 130.2, 129.3, 129.1, 128.9, 128.6, 127.6, 127.3, 125.8, 121.8, 108.8, 94.6.

HR-MS (ESI): *m/z* calcd for $C_{22}H_{13}BrN_2O_2$ $[M+H]^+$ 417.0233, 419.0213, found 417.0235, 419.0213.

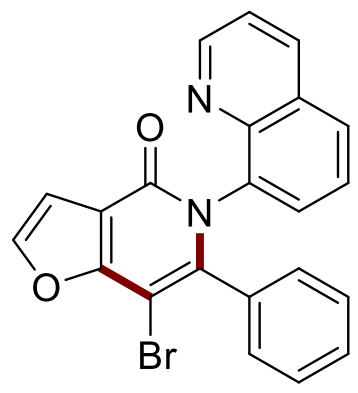

7-Bromo-6-phenyl-5-(quinolin-8-yl)furo[3,2-*c*]pyridin-4(5*H*)-one (23)
Prepared according to the general procedure **A** using **1u** (71.4 mg, 0.30 mmol) and **2a** (81.0 mg, 0.45 mmol), purification by column chromatography on silica gel (petroleum ether/EtOAc/DCM = 1:2:1) yielded **23** (50.0 mg, 40%) as a colorless solid.

^{1}H NMR (600 MHz, CDC_3): δ = 8.91 (dd, *J* = 4.3, 1.6 Hz, 1H), 8.06 (dd, *J* = 8.2, 1.6 Hz, 1H), 7.68 (dd, *J* = 8.2, 1.4 Hz, 1H), 7.65 (d, *J* = 2.1 Hz, 1H), 7.44 (dd, *J* = 7.4, 1.4 Hz, 1H), 7.41–7.35 (m, 2H), 7.19 (dt, *J* = 7.8, 1.6 Hz, 1H), 7.17–7.12 (m, 2H), 7.02 (t,

J = 7.3 Hz, 1H), 6.86 (d, J = 7.7 Hz, 1H), 6.77 (t, J = 7.6 Hz, 1H).

^{13}C NMR (150 MHz, $CDCl_3$): δ = 159.3, 157.4, 151.1, 144.9, 144.4, 144.0, 137.2, 136.2, 134.5, 131.0, 130.1, 129.2, 129.0, 128.8, 128.8, 127.6, 127.4, 125.8, 121.8, 115.7, 109.1, 89.6.

HR-MS (ESI): *m/z* calcd for $C_{22}H_{13}BrN_2O_2$ $[M+H]^+$ 417.0233, 419.0213, found 417.0234, 419.0213.

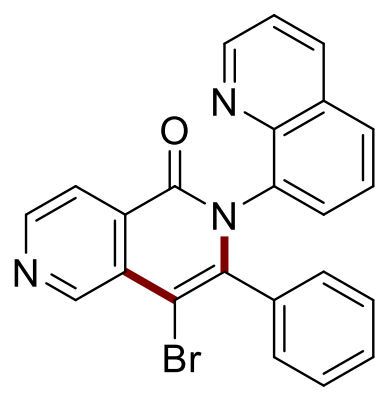

4-Bromo-3-phenyl-2-(quinolin-8-yl)-2,6-naphthyridin-1(2*H*)-one (24)
Prepared according to the general procedure **A** using **1v** (74.7 mg, 0.30 mmol) and **2a** (81.0 mg, 0.45 mmol), purification by column chromatography on silica gel (petroleum ether/EtOAc/DCM = 1:1:1) yielded **24** (32.1 mg, 25%) as a colorless solid.

^{1}H NMR (600 MHz, $CDCl_3$): δ = 9.51 (s, 1H), 8.88 (dd, J = 4.2, 1.7 Hz, 1H), 8.84 (s, 1H), 8.25 (d, J = 5.2 Hz, 1H), 8.07 (dd, J = 8.3, 1.7 Hz, 1H), 7.69 (dd, J = 8.2, 1.4 Hz, 1H), 7.45 (dd, J = 7.3, 1.4 Hz, 1H), 7.41–7.37 (m, 2H), 7.23 (dt, J = 7.7, 1.6 Hz, 1H), 7.18 (td, J = 7.6, 1.3 Hz, 1H), 7.04 (tt, J = 7.5, 1.3 Hz, 1H), 6.88 (d, J = 7.9 Hz, 1H), 6.78 (td, J = 7.6, 1.3 Hz, 1H).

^{13}C NMR (150 MHz, $CDCl_3$): δ = 161.3, 151.1, 150.3, 147.5, 144.8, 144.1, 136.9, 136.2, 135.2, 131.1, 130.6, 130.0, 129.4, 128.9, 128.9, 128.8, 127.7, 127.5, 125.8, 122.0, 120.2, 99.1.

HR-MS (ESI): *m/z* calcd for $C_{23}H_{14}BrN_3O$ $[M+Na]^+$ 450.0212, 452.0192, found 450.0212, 452.0193.

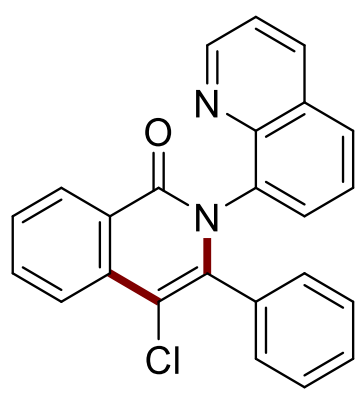

4-Chloro-3-phenyl-2-(quinolin-8-yl)isoquinolin-1(2*H*)-one (25)
Prepared according to the general procedure **A** using **1a** (74.4 mg, 0.30 mmol) and **2b** (61.7 mg, 0.45 mmol), purification by column chromatography on silica gel (petroleum ether/EtOAc/DCM = 2:1:1) yielded **25** (80.4 mg, 70%) as a colorless solid.

^{1}H NMR (600 MHz, $CDCl_3$): δ = 8.90 (dd, J = 4.2, 1.7 Hz, 1H), 8.54 (dd, J = 8.0, 1.4 Hz, 1H), 8.10 (dd, J = 8.2, 1.0 Hz, 1H), 8.06 (dd, J = 8.3, 1.7 Hz, 1H), 7.85 (ddd, J =

8.2, 7.2, 1.4 Hz, 1H), 7.68 (dd, J = 8.2, 1.4 Hz, 1H), 7.62 (t, J = 7.6, 1H), 7.47 (dd, J = 7.3, 1.4 Hz, 1H), 7.41–7.35 (m, 2H), 7.26 (d, J = 7.6, 1H), 7.16 (td, J = 7.6, 1.4 Hz, 1H), 7.02 (tt, J = 7.5, 1.3 Hz, 1H), 6.93 (dt, J = 7.8, 1.6 Hz, 1H), 6.78 (td, J = 7.7, 1.3 Hz, 1H).

^{13}C NMR (150 MHz, $CDCl_3$): δ = 162.2, 151.1, 144.5, 141.3, 137.3, 136.1, 135.6, 134.2, 133.4, 130.9, 130.2, 129.1, 129.0, 128.9, 128.9, 128.5, 127.8, 127.6, 127.4, 126.0, 125.8, 124.2, 121.8, 111.4.

HR-MS (ESI): *m/z* calcd for $C_{24}H_{15}ClN_2O$ $[M+Na]^+$ 405.0765, 407.0736, found 405.0765, 407.0735.

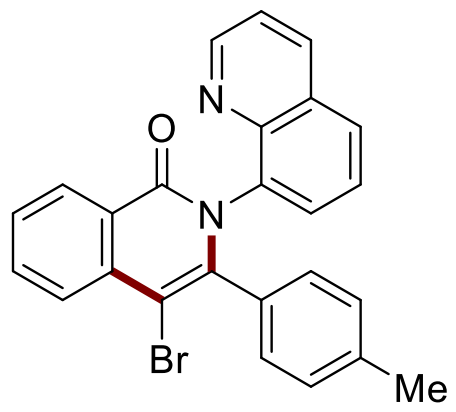

4-Bromo-2-(quinolin-8-yl)-3-(p-tolyl)isoquinolin-1(2*H*)-one (26)

Prepared according to the general procedure **A** using **1a** (74.4 mg, 0.30 mmol) and **2c** (87.5 mg, 0.45 mmol), purification by column chromatography on silica gel (petroleum ether/EtOAc/DCM = 3:1:1) yielded **26** (83.3 mg, 63%) as a colorless solid.

^{1}H NMR (600 MHz, $CDCl_3$): δ = 8.89 (d, J = 3.8 Hz, 1H), 8.52 (d, J = 7.9 Hz, 1H), 8.11 (d, J = 8.2 Hz, 1H), 8.06 (d, J = 8.3 Hz, 1H), 7.82 (t, J = 7.7 Hz, 1H), 7.68 (d, J = 8.0 Hz, 1H), 7.59 (t, J = 7.6 Hz, 1H), 7.45 (d, J = 7.3 Hz, 1H), 7.41–7.35 (m, 2H), 7.13 (d, J = 7.8, 1H), 6.96 (d, J = 7.8 Hz, 1H), 6.78 (d, J = 7.9, 1H), 6.57 (d, J = 7.9 Hz, 1H), 2.12 (s, 3H).

^{13}C NMR (150 MHz, $CDCl_3$): δ = 162.5, 151.0, 144.5, 143.1, 138.3, 137.7, 136.5, 136.1, 133.5, 133.3, 130.8, 130.0, 128.9, 128.9, 128.8, 128.7, 128.3, 128.0, 127.7, 126.9, 126.0, 125.9, 121.7, 102.5, 21.3.

HR-MS (ESI): *m/z* calcd for $C_{25}H_{17}BrN_2O$ $[M+Na]^+$ 463.0416, 465.0396, found 463.0416, 465.0396.

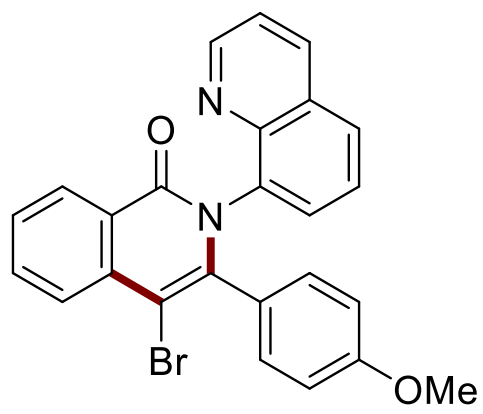

4-Bromo-3-(4-methoxyphenyl)-2-(quinolin-8-yl)isoquinolin-1(2*H*)-one (27)

Prepared according to the general procedure **A** using **1a** (74.4 mg, 0.30 mmol) and **2d** (94.5 mg, 0.45 mmol), purification by column chromatography on silica gel (petroleum ether/EtOAc/DCM = 1:1:1) yielded **27** (60.3 mg, 44%) as a colorless solid.

^{1}H NMR (600 MHz, $CDCl_3$): δ = 8.89 (dd, J = 4.1, 1.7 Hz, 1H), 8.51 (d, J = 7.9 Hz, 1H), 8.11 (d, J = 8.2 Hz, 1H), 8.07 (dd, J = 8.3, 1.7 Hz, 1H), 7.82 (t, J = 7.6 Hz, 1H), 7.69 (dd, J = 8.2, 1.4 Hz, 1H), 7.59 (t, J = 7.6 Hz, 1H), 7.46 (dd, J = 7.2, 1.4 Hz, 1H), 7.40 (t, J = 7.8 Hz, 1H), 7.37 (dd, J = 8.3, 4.1 Hz, 1H), 7.15 (dd, J = 8.5, 2.3 Hz, 1H), 6.83 (dd, J = 8.6, 2.2 Hz, 1H), 6.68 (dd, J = 8.5, 2.7 Hz, 1H), 6.29 (dd, J = 8.6, 2.7 Hz, 1H), 3.62 (s, 3H).

^{13}C NMR (150 MHz, $CDCl_3$): δ = 162.5, 159.3, 151.0, 144.5, 142.9, 137.7, 136.5, 136.2, 133.5, 131.4, 130.8, 130.3, 129.0, 128.9, 128.8, 128.7, 127.7, 126.9, 126.0, 125.9, 121.7, 112.9, 112.7, 102.9, 55.1.

HR-MS (ESI): *m/z* calcd for $C_{25}H_{17}BrN_2O_2$ $[M+Na]^+$ 479.0366, 481.0346, found 479.0364, 481.0347.

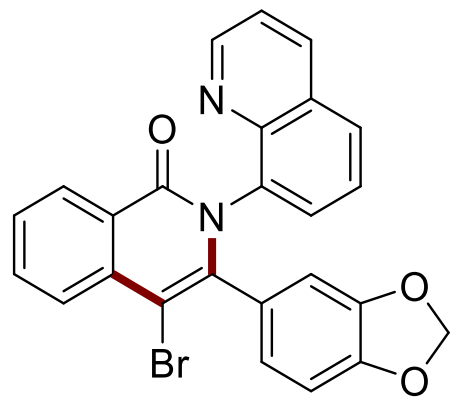

3-(Benzo[d][1,3]dioxol-5-yl)-4-bromo-2-(quinolin-8-yl)isoquinolin-1(2*H*)-one (28)
Prepared according to the general procedure **A** using **1a** (74.4 mg, 0.30 mmol) and **2e** (101.0 mg, 0.45 mmol), purification by column chromatography on silica gel (petroleum ether/EtOAc/DCM = 1:1:1) yielded **28** (80.5 mg, 57%) as a colorless solid.

^{1}H NMR (600 MHz, $CDCl_3$, compound exists as a mixture of rotamers): δ = 8.89 (td, J = 4.2, 1.6 Hz, 2H), 8.50 (d, J = 7.9 Hz, 2H), 8.12–8.08 (m, 4H), 7.82 (td, J = 7.7, 1.4 Hz, 2H), 7.75–7.71 (m, 2H), 7.59 (t, J = 7.6 Hz, 2H), 7.48–7.41 (m, 4H), 7.39 (dt, J = 7.9, 3.7 Hz, 2H), 6.75–6.67 (m, 2H), 6.60 (d, J = 8.0 Hz, 1H), 6.43–6.37 (m, 2H), 6.20 (d, J = 8.1 Hz, 1H), 5.87 (s, 1H), 5.84 (s, 1H), 5.76 (s, 1H), 5.67 (s, 1H).

^{13}C NMR (151 MHz, CDCl3): δ = 162.4, 151.2, 151.1, 147.5, 147.4, 146.9, 146.6, 144.6, 144.4, 142.4, 142.4, 137.6, 137.5, 136.4, 136.4, 136.2, 136.2, 133.6, 130.9, 130.6, 129.9, 129.7, 129.1, 129.1, 128.9, 128.9, 128.8, 127.8, 126.9, 126.9, 126.0, 126.0, 125.9, 124.3, 123.1, 121.9, 121.8, 110.5, 109.8, 107.6, 107.4, 102.9, 101.2, 101.0.

HR-MS (ESI): *m/z* calcd for $C_{25}H_{15}BrN_2O_3$ $[M+Na]^+$ 493.0158, 495.0138, found 493.0157, 495.0138.

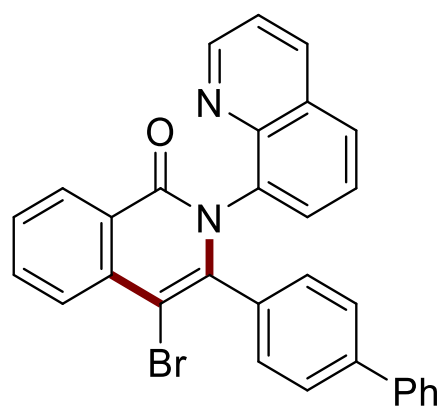

3-([1,1'-Biphenyl]-4-yl)-4-bromo-2-(quinolin-8-yl)isoquinolin-1(2*H*)-one (29)

Prepared according to the general procedure **A** using **1a** (74.4 mg, 0.30 mmol) and **2f** (115.2 mg, 0.45 mmol), purification by column chromatography on silica gel (petroleum ether/EtOAc/DCM = 2:1:1) yielded **29** (105.6 mg, 70%) as a colorless solid.

^{1}H NMR (600 MHz, $CDCl_3$): δ = 8.92 (d, J = 4.0 Hz, 1H), 8.54 (d, J = 7.9 Hz, 1H), 8.14 (d, J = 8.2 Hz, 1H), 8.05 (d, J = 8.2 Hz, 1H), 7.84 (t, J = 7.7 Hz, 1H), 7.67 (d, J = 8.2 Hz, 1H), 7.61 (t, J = 7.6 Hz, 1H), 7.50 (d, J = 7.2 Hz, 1H), 7.43–7.39 (m, 4H), 7.38 (d, J = 4.1 Hz, 1H), 7.35 (t, J = 8.1 Hz, 2H), 7.31 (t, J = 7.9 Hz, 1H), 7.29 (t, J = 7.5 Hz, 1H), 7.02 (d, J = 7.8 Hz, 1H), 6.98 (d, J = 8.2 Hz, 1H).

^{13}C NMR (150 MHz, $CDCl_3$): δ = 162.4, 151.1, 144.5, 142.8, 140.9, 140.1, 137.5, 136.5, 136.2, 135.1, 133.6, 130.9, 130.6, 129.4, 129.1, 128.9, 128.8, 128.8, 127.8, 127.7, 127.0, 126.9, 126.1, 126.0, 125.9, 125.8, 121.8, 102.4.

HR-MS (ESI): *m/z* calcd for $C_{30}H_{19}BrN_2O$ $[M+Na]^+$ 525.0573, 527.0553, found 525.0573, 527.0553.

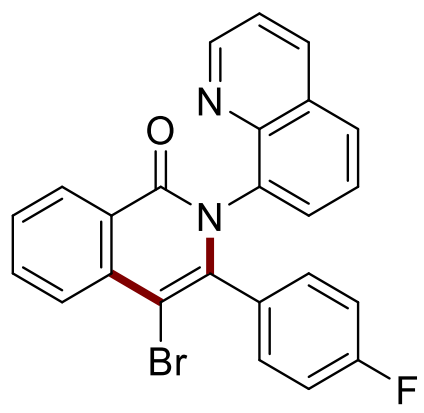

4-Bromo-3-(4-fluorophenyl)-2-(quinolin-8-yl)isoquinolin-1(2*H*)-one (30)

Prepared according to the general procedure **A** using **1a** (74.4 mg, 0.30 mmol) and **2g** (89.1 mg, 0.45 mmol), purification by column chromatography on silica gel (petroleum ether/EtOAc/DCM = 3:1:1) yielded **30** (93.5 mg, 70%) as a colorless solid.

^{1}H NMR (600 MHz, $CDCl_3$): δ = 8.88 (dd, J = 4.2, 1.7 Hz, 1H), 8.52 (d, J = 8.0 Hz, 1H), 8.11 (d, J = 8.2 Hz, 1H), 8.08 (dd, J = 8.3, 1.7 Hz, 1H), 7.83 (ddd, J = 8.4, 7.2, 1.4 Hz, 1H), 7.71 (dd, J = 8.2, 1.5 Hz, 1H), 7.61 (t, J = 7.6 Hz, 1H), 7.48 (d, J = 7.2 Hz, 1H), 7.41 (t, J = 7.7 Hz, 1H), 7.38 (dd, J = 8.3, 4.1 Hz, 1H), 7.21 (ddd, J = 8.2, 5.3, 2.3 Hz, 1H), 6.94 (ddd, J = 8.2, 5.4, 2.3 Hz, 1H), 6.84 (td, J = 8.6, 2.7 Hz, 1H), 6.47 (td, J = 8.6, 2.7 Hz, 1H).

^{13}C NMR (150 MHz, $CDCl_3$): δ = 162.4, 162.3 (d, $^1J_{C–F}$ = 248.9 Hz), 151.1, 144.3, 142.0, 137.4, 136.3, 136.2, 133.7, 132.2 (d, $^4J_{C–F}$ = 3.7 Hz), 132.0 (d, $^3J_{C–F}$ = 8.3 Hz), 131.1, 131.1 (d, $^3J_{C–F}$ = 8.3 Hz), 129.2, 128.9, 128.8, 128.0, 126.9, 126.1, 125.9, 121.9, 114.8 (d, $^2J_{C–F}$ = 22.0 Hz), 114.5 (d, $^2J_{C–F}$ = 21.6 Hz), 102.7.

^{19}F NMR (565 MHz, $CDCl_3$): δ = -111.91 (tq, J = 9.0, 5.3 Hz).

HR-MS (ESI): *m/z* calcd for $C_{24}H_{14}BrFN_2O$ $[M+Na]^+$ 467.0166, 469.0146, found 467.0166, 469.0147.

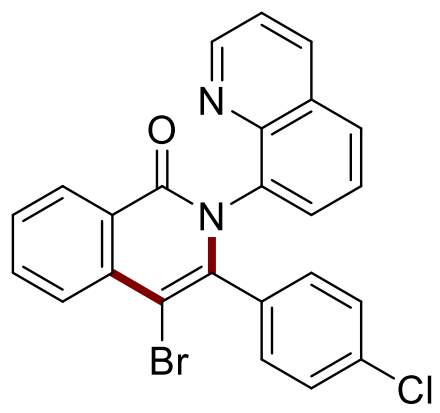

4-Bromo-3-(4-chlorophenyl)-2-(quinolin-8-yl)isoquinolin-1(2*H*)-one (31)

Prepared according to the general procedure **A** using **1a** (74.4 mg, 0.30 mmol) and **2h** (96.5 mg, 0.45 mmol), purification by column chromatography on silica gel (petroleum ether/EtOAc/DCM = 3:1:1) yielded **31** (99.8 mg, 72%) as a colorless solid.

^{1}H NMR (600 MHz, $CDCl_3$): δ = 8.88 (dd, J = 4.2, 1.7 Hz, 1H), 8.52 (d, J = 8.0 Hz, 1H), 8.11 (d, J = 8.2 Hz, 1H), 8.08 (dd, J = 8.3, 1.7 Hz, 1H), 7.83 (t, J = 7.7 Hz, 1H), 7.72 (d, J = 8.2 Hz, 1H), 7.61 (t, J = 7.6 Hz, 1H), 7.48 (dd, J = 7.2, 1.4 Hz, 1H), 7.41 (t, J = 7.8 Hz, 1H), 7.38 (dd, J = 8.3, 4.2 Hz, 1H), 7.17 (dd, J = 8.3, 2.1 Hz, 1H), 7.13 (dd, J = 8.3, 2.2 Hz, 1H), 6.89 (dd, J = 8.3, 2.1 Hz, 1H), 6.76 (dd, J = 8.3, 2.2 Hz, 1H).

^{13}C NMR (150 MHz, $CDCl_3$): δ = 162.3, 151.1, 144.4, 141.8, 137.3, 136.3, 136.3, 134.6, 134.5, 133.7, 131.5, 130.8, 130.5, 129.3, 129.0, 128.8, 128.0, 127.9, 127.7, 126.9, 126.1, 125.9, 121.9, 102.5.

HR-MS (ESI): *m/z* calcd for $C_{24}H_{14}BrClN_2O$ $[M+Na]^+$ 482.9870, 484.9850, found 482.9867, 484.9848.

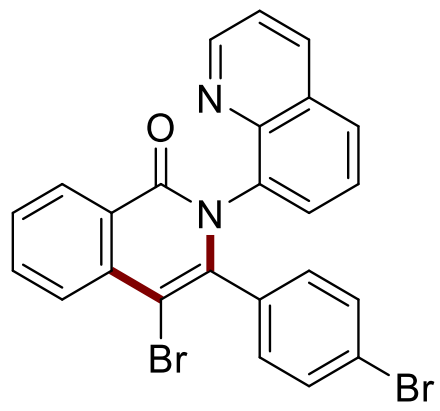

4-Bromo-3-(4-bromophenyl)-2-(quinolin-8-yl)isoquinolin-1(2*H*)-one (32)

Prepared according to the general procedure **A** using **1a** (74.4 mg, 0.30 mmol) and **2i** (116.0 mg, 0.45 mmol), purification by column chromatography on silica gel (petroleum ether/EtOAc/DCM = 3:1:1) yielded **32** (112.3 mg, 74%) as a pale yellow solid.

^{1}H NMR (600 MHz, $CDCl_3$): δ = 8.88 (dd, J = 4.1, 1.7 Hz, 1H), 8.51 (dd, J = 8.0, 1.5 Hz, 1H), 8.13–8.07 (m, 2H), 7.83 (ddd, J = 8.4, 7.2, 1.5 Hz, 1H), 7.73 (dd, J = 8.2, 1.6 Hz, 1H), 7.61 (t, J = 7.6 Hz, 1H), 7.52–7.45 (m, 1H), 7.42 (t, J = 7.8 Hz, 1H), 7.38 (dd, J = 8.3, 4.1 Hz, 1H), 7.29 (dd, J = 8.2, 2.1 Hz, 1H), 7.11 (dd, J = 8.3, 2.0 Hz, 1H), 6.92 (dd, J = 8.3, 1.9 Hz, 1H), 6.83 (dd, J = 8.2, 2.0 Hz, 1H).

^{13}C NMR (150 MHz, $CDCl_3$): δ = 162.3, 151.1, 144.4, 141.8, 137.3, 136.3, 136.3, 135.1, 133.7, 131.7, 130.9, 130.8, 130.8, 130.6, 129.4, 129.0, 128.9, 128.1, 126.9, 126.1, 125.9, 122.9, 121.9, 102.4.

HR-MS (ESI): *m/z* calcd for $C_{24}H_{14}Br_2N_2O$ $[M+Na]^+$ 526.9365, 528.9345, found 526.9364, 528.9344.

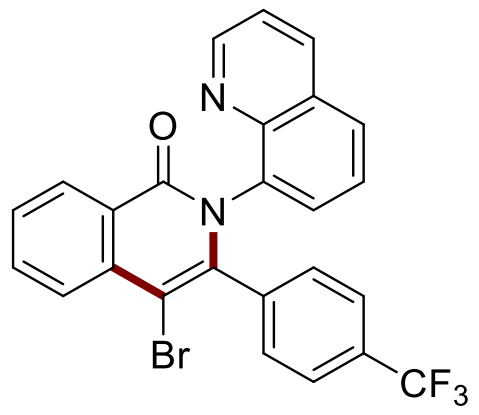

4-Bromo-2-(quinolin-8-yl)-3-(4-(trifluoromethyl)phenyl)isoquinolin-1(2*H*)-one (33)

Prepared according to the general procedure **A** using **1a** (74.4 mg, 0.30 mmol) and **2j** (111.6 mg, 0.45 mmol), purification by column chromatography on silica gel (petroleum ether/EtOAc/DCM = 3:1:1) yielded **33** (102.5 mg, 69%) as a colorless solid.

^{1}H NMR (600 MHz, $CDCl_3$): δ = 8.89 (d, J = 4.2 Hz, 1H), 8.53 (d, J = 7.9 Hz, 1H), 8.11 (d, J = 8.2 Hz, 1H), 8.08 (d, J = 8.0 Hz, 1H), 7.85 (t, J = 7.7 Hz, 1H), 7.71 (d, J = 8.2 Hz, 1H), 7.63 (t, J = 7.6 Hz, 1H), 7.48 (d, J = 7.2 Hz, 1H), 7.45–7.35 (m, 4H), 7.09 (d, J = 8.1 Hz, 1H), 7.05 (d, J = 8.1 Hz, 1H).

^{13}C NMR (150 MHz, $CDCl_3$): δ = 162.3, 151.2, 144.3, 141.5, 139.6, 137.1, 136.3, 136.2, 133.8, 130.8, 130.5 (q, $^2J_{C–F}$ = 32.7 Hz), 129.7, 129.5, 129.0, 128.9, 128.2, 126.9, 126.1, 125.9, 124.6 (q, $^3J_{C–F}$ = 3.8 Hz), 124.3 (q, $^3J_{C–F}$ = 3.6 Hz),123.7 (q, $^1J_{C–F}$ = 272.0 Hz), 122.8, 122.0, 102.3.

^{19}F NMR (565 MHz, CDCl3) δ -62.93.

HR-MS (ESI): *m/z* calcd for $C_{25}H_{14}BrF_3N_2O$ $[M+Na]^+$ 517.0134, 519.0114, found 517.0132, 519.0113.

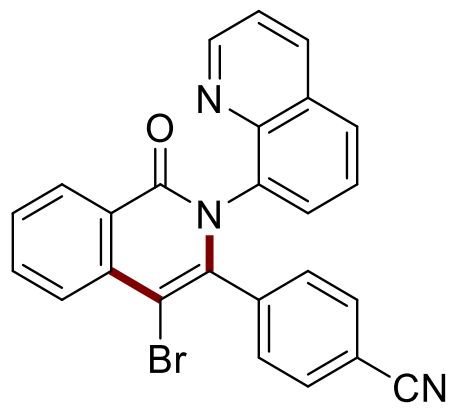

4-(4-Bromo-1-oxo-2-(quinolin-8-yl)-1,2-dihydroisoquinolin-3-yl)benzonitrile (34)

Prepared according to the general procedure **A** using **1a** (74.4 mg, 0.30 mmol) and **2k** (93.0 mg, 0.45 mmol), purification by column chromatography on silica gel (petroleum ether/EtOAc/DCM = 2:1:1) yielded **34** (81.4 mg, 60%) as a colorless solid.

^{1}H NMR (600 MHz, $CDCl_3$): δ = 8.88 (dd, J = 4.0, 1.7 Hz, 1H), 8.52 (d, J = 7.9 Hz, 1H), 8.13–8.06 (m, 2H), 7.85 (ddd, J = 8.5, 7.2, 1.5 Hz, 1H), 7.73 (dd, J = 8.2, 1.6 Hz, 1H), 7.64 (t, J = 7.6 Hz, 1H), 7.50 (d, J = 6.0 Hz, 1H), 7.45–7.38 (m, 3H), 7.35 (d, J = 8.0 Hz, 1H), 7.12 (d, J = 8.1 Hz, 1H), 7.08 (d, J = 8.1 Hz, 1H).

^{13}C NMR (150 MHz, $CDCl_3$): δ = 162.1, 151.2, 144.2, 140.9, 140.5, 136.9, 136.4, 136.0, 133.8, 131.4, 131.1, 131.0, 130.9, 130.2, 129.6, 129.0, 128.9, 128.4, 127.0, 126.2, 125.9, 122.1, 118.2, 112.4, 102.1.

HR-MS (ESI): *m/z* calcd for $C_{25}H_{14}BrN_3O$ $[M+Na]^+$ 474.0212, 476.0192, found 474.0213, 476.0191.

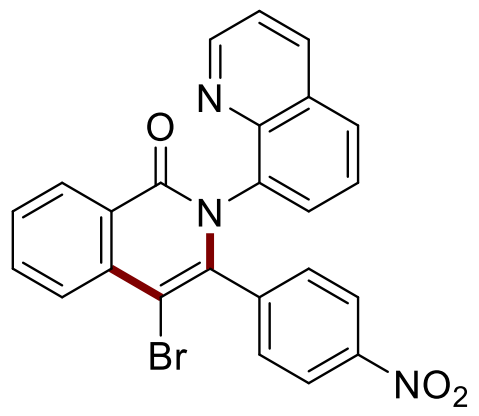

4-Bromo-3-(4-nitrophenyl)-2-(quinolin-8-yl)isoquinolin-1(2*H*)-one (35)

Prepared according to the general procedure **A** using **1a** (74.4 mg, 0.30 mmol) and **2l** (102.0 mg, 0.45 mmol), purification by column chromatography on silica gel (petroleum ether/EtOAc/DCM = 1:1:1) yielded **35** (85.0 mg, 60%) as a pale yellow solid.

^{1}H NMR (600 MHz, $CDCl_3$): δ = 8.90 (dd, *J* = 4.2, 1.7 Hz, 1H), 8.53 (d, *J* = 7.9 Hz, 1H), 8.11 (d, *J* = 8.2 Hz, 1H), 8.08 (dd, *J* = 8.3, 1.7 Hz, 1H), 8.01 (dd, *J* = 8.5, 2.4 Hz, 1H), 7.86 (ddd, *J* = 8.2, 7.9, 1.4 Hz, 1H), 7.72 (dd, *J* = 8.2, 1.4 Hz, 1H), 7.68–7.62 (m, 2H), 7.52 (dd, *J* = 7.3, 1.4 Hz, 1H), 7.45–7.39 (m, 3H), 7.21 (dd, *J* = 8.5, 1.9 Hz, 1H).

^{13}C NMR (150 MHz, $CDCl_3$): δ = 162.1, 151.3, 147.5, 144.2, 142.3, 140.6, 136.9, 136.4, 136.0, 133.9, 131.3, 130.9, 130.6, 129.7, 129.0, 128.9, 128.5, 127.0, 126.2, 126.0, 122.8, 122.6, 122.1, 102.2.

HR-MS (ESI): *m/z* calcd for $C_{24}H_{14}BrN_3O_3$ $[M+Na]^+$ 494.0111, 496.0091, found 494.0112, 496.0091.

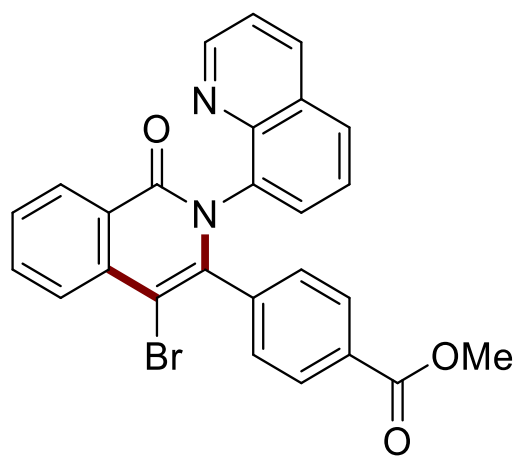

Methyl 4-(4-bromo-1-oxo-2-(quinolin-8-yl)-1,2-dihydroisoquinolin-3-yl)benzoate (36)

Prepared according to the general procedure **A** using **1a** (74.4 mg, 0.30 mmol) and **2m** (107.0 mg, 0.45 mmol), purification by column chromatography on silica gel (petroleum ether/EtOAc/DCM = 1:1:1) yielded **36** (107.7 mg, 74%) as a colorless solid.

^{1}H NMR (600 MHz, $CDCl_3$): δ = 8.89 (dd, *J* = 4.2, 1.7 Hz, 1H), 8.52 (dd, *J* = 8.0, 1.4

Hz, 1H), 8.11 (d, J = 8.2 Hz, 1H), 8.05 (dd, J = 8.2, 1.7 Hz, 1H), 7.86–7.81 (m, 2H), 7.68 (dd, J = 8.2, 1.4 Hz, 1H), 7.62 (t, J = 7.6 Hz, 1H), 7.48 (dd, J = 7.3, 1.4 Hz, 1H), 7.46 (dd, J = 8.2, 1.8 Hz, 1H), 7.40–7.36 (m, 2H), 7.33 (dd, J = 8.0, 1.8 Hz, 1H), 7.04 (dd, J = 8.1, 1.8 Hz, 1H), 3.80 (s, 3H).

^{13}C NMR (150 MHz, $CDCl_3$): δ = 166.5, 162.3, 151.2, 144.3, 141.9, 140.5, 137.2, 136.2, 136.2, 133.7, 130.8, 130.3, 130.0, 129.4, 129.3, 128.9, 128.9, 128.8, 128.6, 128.1, 126.9, 126.1, 125.9, 121.9, 52.3.

HR-MS (ESI): m/z calcd for $C_{26}H_{17}BrN_2O_3$ $[M+Na]^+$ 507.0315, 509.0295, found 507.0316, 509.0296.

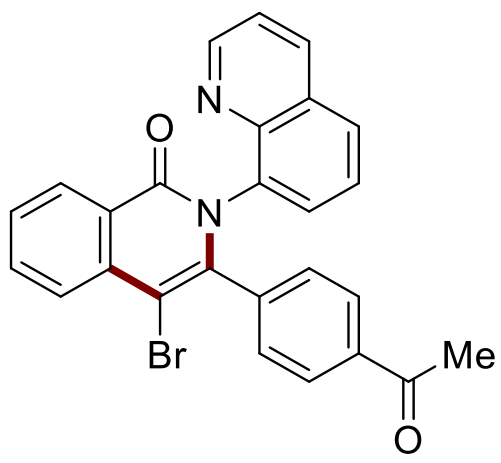

3-(4-Acetylphenyl)-4-bromo-2-(quinolin-8-yl)isoquinolin-1(2*H*)-one (37)
Prepared according to the general procedure **A** using **1a** (74.4 mg, 0.30 mmol) and **2n** (100.0 mg, 0.45 mmol), purification by column chromatography on silica gel (petroleum ether/EtOAc/DCM = 2:1:1) yielded **37** (85.8 mg, 61%) as a colorless solid.

^{1}H NMR (600 MHz, $CDCl_3$): δ = 8.89 (dd, J = 4.2, 1.7 Hz, 1H), 8.52 (d, J = 7.9 Hz, 1H), 8.11 (d, J = 8.2 Hz, 1H), 8.06 (dd, J = 8.3, 1.7 Hz, 1H), 7.84 (t, J = 7.7 Hz, 1H), 7.75 (dd, J = 8.0, 1.9 Hz, 1H), 7.68 (d, J = 8.2 Hz, 1H), 7.62 (t, J = 7.6 Hz, 1H), 7.49 (d, J = 7.3 Hz, 1H), 7.41–7.34 (m, 4H), 7.07 (dd, J = 8.1, 1.8 Hz, 1H), 2.43 (s, 3H).

^{13}C NMR (150 MHz, $CDCl_3$): δ = 197.5, 162.2, 151.2, 144.3, 141.8, 140.6, 137.2, 136.6, 136.3, 136.2, 133.7, 130.8, 130.5, 129.5, 129.4, 128.9, 128.9, 128.1, 127.6, 127.3, 126.9, 126.1, 125.9, 121.9, 102.1, 26.6.

HR-MS (ESI): m/z calcd for $C_{26}H_{17}BrN_2O_2$ $[M+Na]^+$ 491.0366, 493.0346, found 491.0365, 493.0348.

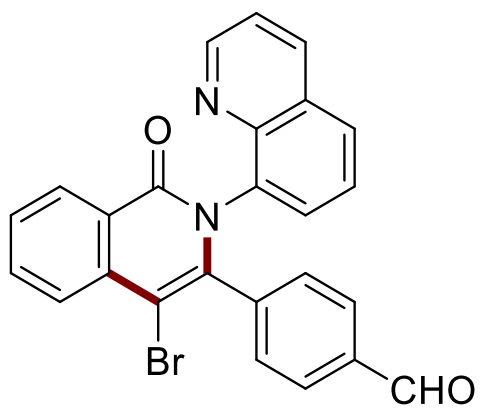

5-(4-Bromo-1-oxo-2-(quinolin-8-yl)-1,2-dihydroisoquinolin-3-yl)benzaldehyde (38)
Prepared according to the general procedure **A** using **1a** (74.4 mg, 0.30 mmol) and **2o** (93.6 mg, 0.45 mmol), purification by column chromatography on silica gel (petroleum ether/EtOAc/DCM = 1:1:1) yielded **38** (105.1 mg, 77%) as a colorless solid.

^{1}H NMR (600 MHz, $CDCl_3$): δ = 9.78 (s, 1H), 8.90 (dd, J = 3.2, 1.1 Hz, 1H), 8.53 (d, J = 7.9 Hz, 1H), 8.11 (d, J = 8.2 Hz, 1H), 8.06 (d, J = 8.2 Hz, 1H), 7.85 (t, J = 7.7 Hz, 1H), 7.71–7.65 (m, 2H), 7.63 (t, J = 7.6 Hz, 1H), 7.50 (d, J = 7.3 Hz, 1H), 7.43 (d, J = 7.9 Hz, 1H), 7.41–7.36 (m, 2H), 7.30 (dd, J = 8.0, 2.0 Hz, 1H), 7.16 (dd, J = 8.0, 1.7 Hz, 1H).

^{13}C NMR (150 MHz, $CDCl_3$): δ = 191.6, 162.2, 151.2, 144.3, 141.9, 141.6, 137.1, 136.3, 136.1, 135.8, 133.8, 131.0, 130.9, 130.0, 129.5, 129.0, 128.9, 128.8, 128.6, 128.2, 126.9, 126.1, 125.9, 122.0, 102.0.

HR-MS (ESI): *m/z* calcd for $C_{25}H_{15}BrN_2O_2$ $[M+Na]^+$ 477.0209, 479.0189, found 477.0208, 479.0191.

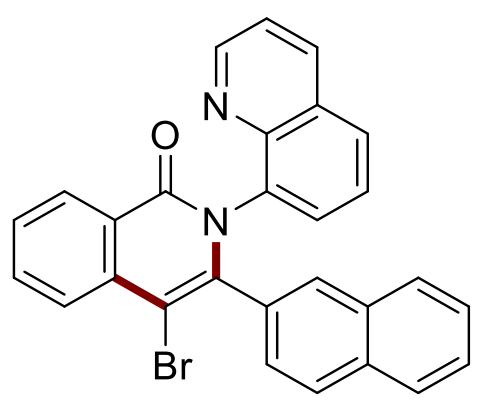

4-Bromo-3-(naphthalen-2-yl)-2-(quinolin-8-yl)isoquinolin-1(2*H*)-one (39)
Prepared according to the general procedure **A** using **1a** (74.4 mg, 0.30 mmol) and **2p** (103.5 mg, 0.45 mmol), purification by column chromatography on silica gel (petroleum ether/EtOAc/DCM = 2:1:1) yielded **39** (97.3 mg, 68%) as a colorless solid.

^{1}H NMR (600 MHz, $CDCl_3$, compound exists as a mixture of rotamers): δ = 8.97 (dd, J = 4.3, 1.6 Hz, 1H), 8.93 (dd, J = 4.2, 1.6 Hz, 1H), 8.55 (d, J = 8.0 Hz, 2H), 8.14 (d, J = 8.3, 4.5 Hz, 2H), 8.00 (dd, J = 8.4, 1.7 Hz, 1H), 7.94 (dd, J = 8.3, 1.7 Hz, 1H), 7.84 (t, J = 7.7 Hz, 2H), 7.77–7.73 (m, 2H), 7.66–7.60 (m, 4H), 7.57 (t, J = 8.7 Hz, 2H), 7.54 (d, J = 8.3 Hz, 1H), 7.52 (dd, J = 7.1, 3.3 Hz, 2H), 7.46 (s, 1H), 7.43 (t, J = 7.3 Hz, 1H), 7.39 (d, J = 7.0 Hz, 1H), 7.38–7.30 (m, 5H), 7.30–7.26 (m, 2H), 7.26–7.23 (m, 2H), 7.01 (dd, J = 8.5, 1.7 Hz, 1H).

^{13}C NMR (151 MHz, CDCl3): δ = 162.5, 162.4, 151.1, 151.0, 144.6, 144.5, 142.9, 137.6, 137.5, 136.5, 136.2, 133.8, 133.6, 133.4, 132.7, 132.7, 132.3, 132.1, 130.9, 130.5, 130.0, 129.2, 129.0, 128.9, 128.8, 128.2, 128.1, 127.9, 127.7, 127.6, 127.3, 127.3, 126.9, 126.9, 126.7, 126.6, 126.4, 126.3, 126.1, 126.1, 126.0, 125.8, 125.8, 121.8, 121.7, 102.6, 102.6.

HR-MS (ESI): *m/z* calcd for $C_{28}H_{17}BrN_2O$ $[M+Na]^+$ 499.0416, 501.0396, found 499.0414, 501.0397.

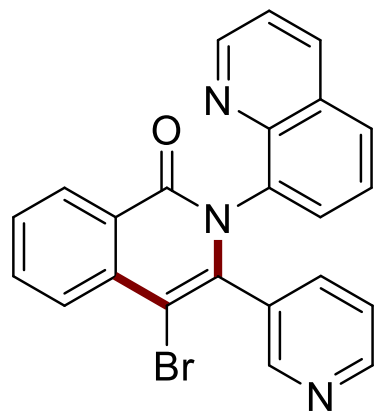

4-Bromo-3-(pyridin-3-yl)-2-(quinolin-8-yl)isoquinolin-1(2*H*)-one (40)
Prepared according to the general procedure **A** using **1a** (74.4 mg, 0.30 mmol) and **2q** (81.5 mg, 0.45 mmol), purification by column chromatography on silica gel (petroleum ether/EtOAc/DCM = 1:1:1) yielded **40** (30.8 mg, 24%) as a colorless solid.

^{1}H NMR (600 MHz, $CDCl_3$, compound exists as a mixture of rotamers): δ = 8.89 (td, J = 4.6, 1.6 Hz, 2H), 8.53 (d, J = 7.9 Hz, 2H), 8.50 (s, 1H), 8.29 (s, 1H), 8.27–8.18 (m, 2H), 8.11 (d, J = 8.2 Hz, 2H), 8.07 (dd, J = 8.3, 1.7 Hz, 1H), 8.04 (dd, J = 8.4, 1.7 Hz, 1H), 7.85 (t, J = 7.7 Hz, 2H), 7.71 (d, J = 8.6 Hz, 2H), 7.63 (t, J = 7.6 Hz, 2H), 7.58–7.49 (m, 3H), 7.43 (q, J = 8.0 Hz, 2H), 7.38 (td, J = 8.8, 4.2 Hz, 2H), 7.31 (d, J = 7.9 Hz, 1H), 7.04 (dd, J = 7.8, 4.9 Hz, 1H), 6.74 (dd, J = 7.9, 4.8 Hz, 1H).

^{13}C NMR (151 MHz, CDCl3): δ = 162.3, 162.2, 151.4, 151.2, 150.4, 149.9, 149.4, 149.3, 144.2, 144.0, 139.8, 139.7, 137.4, 137.1, 137.0, 136.5, 136.3, 136.2, 136.1, 133.8, 132.5, 132.2, 131.0, 130.7, 129.5, 129.4, 129.1, 129.0, 128.9, 128.3, 127.0, 127.0, 126.3, 126.2, 125.7, 122.2, 122.1, 122.0, 103.2.

HR-MS (ESI): *m/z* calcd for $C_{23}H_{14}BrN_3O$ $[M+Na]^+$ 450.0212, 452.0192, found 450.0210, 452.0191.

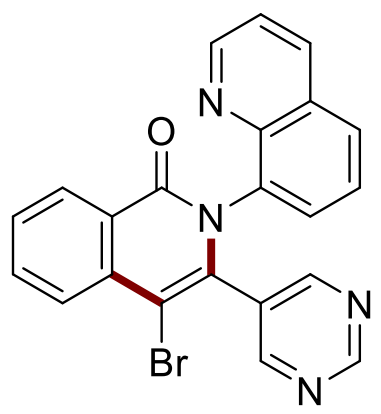

4-Bromo-3-(pyrimidin-5-yl)-2-(quinolin-8-yl)isoquinolin-1(2*H*)-one (41)
Prepared according to the general procedure **A** using **1a** (74.4 mg, 0.30 mmol) and **2r** (82.0 mg, 0.45 mmol), purification by column chromatography on silica gel (petroleum ether/EtOAc/DCM = 1:1:1) yielded **41** (38.6 mg, 30%) as a colorless solid.

^{1}H NMR (600 MHz, $CDCl_3$): δ = 8.88 (dd, J = 4.2, 1.7 Hz, 1H), 8.79 (s, 1H), 8.55 (d, J = 2.9 Hz, 1H), 8.53 (dd, J = 8.0, 1.4 Hz, 1H), 8.49 (d, J = 2.9 Hz, 1H), 8.11 (d, J = 8.2 Hz, 1H), 8.08 (dd, J = 8.3, 1.7 Hz, 1H), 7.87 (ddd, J = 8.4, 7.3, 1.4 Hz, 1H), 7.77 (dd, J = 8.1, 1.2 Hz, 1H), 7.69–7.60 (m, 2H), 7.51 (t, J = 7.8 Hz, 1H), 7.40 (dd, J = 8.3, 4.1 Hz, 1H).

^{13}C NMR (150 MHz, $CDCl_3$): δ = 162.1, 157.9, 157.0, 157.0, 151.5, 143.7, 136.7, 136.5, 136.4, 135.7, 133.9, 130.9, 130.8, 130.0, 129.2, 129.0, 128.8, 127.1, 126.4, 126.2, 122.3,

103.9.

HR-MS (ESI): *m/z* calcd for $C_{22}H_{13}BrN_4O$ $[M+Na]^+$ 451.0165, 453.0145, found 451.0164, 453.0145, 453.0145.

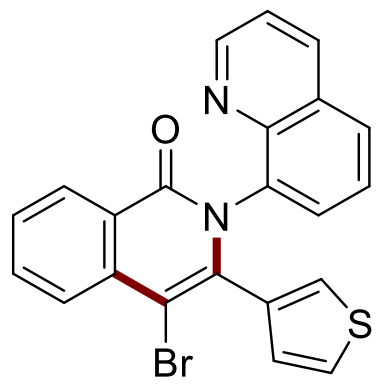

4-Bromo-2-(quinolin-8-yl)-3-(thiophen-3-yl)isoquinolin-1(2*H*)-one (42)
Prepared according to the general procedure **A** using **1a** (74.4 mg, 0.30 mmol) and **2s** (83.7 mg, 0.45 mmol), purification by column chromatography on silica gel (petroleum ether/EtOAc/DCM = 1:2:1) yielded **42** (87.0 mg, 67%) as a colorless solid.

^{1}H NMR (600 MHz, $CDCl_3$, compound exists as a mixture of rotamers): δ = 8.89 (d, *J* = 5.0 Hz, 2H), 8.51 (d, *J* = 7.9 Hz, 2H), 8.11 (d, *J* = 7.9 Hz, 4H), 7.82 (t, *J* = 7.7 Hz, 2H), 7.73 (d, *J* = 8.1 Hz, 2H), 7.60 (t, *J* = 7.6 Hz, 2H), 7.48 (d, *J* = 7.1 Hz, 2H), 7.43 (t, *J* = 7.7 Hz, 2H), 7.39 (dd, *J* = 8.4, 4.2 Hz, 2H), 7.18 (s, 1H), 7.03 (s, 1H), 6.95 (s, 1H), 6.77 (s, 1H), 6.72 (s, 1H), 6.44 (s, 1H).

^{13}C NMR (151 MHz, CDCl3): δ = 162.4, 151.1, 144.5, 138.6, 137.6, 136.3, 136.2, 135.5, 133.6, 130.6, 130.1, 129.1, 128.9, 128.8, 128.2, 127.9, 127.1, 126.9, 126.1, 126.0, 126.0, 124.5, 124.3, 121.8, 103.2.

HR-MS (ESI): *m/z* calcd for $C_{22}H_{13}BrN_2OS$ $[M+Na]^+$ 454.9824, 456.9804, found 454.9824, 456.9804.

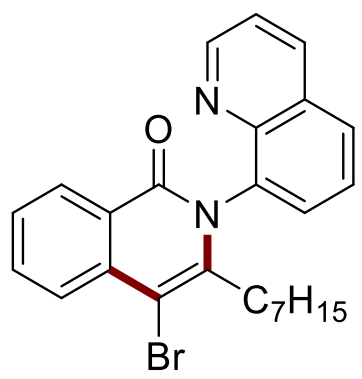

4-Bromo-3-heptyl-2-(quinolin-8-yl)isoquinolin-1(2*H*)-one (43)
Prepared according to the general procedure **A** using **1a** (74.4 mg, 0.30 mmol) and **2t** (91.0 mg, 0.45 mmol), purification by column chromatography on silica gel (petroleum ether/EtOAc/DCM = 2:1:1) yielded **43** (87.4 mg, 65%) as a colorless solid.

^{1}H NMR (600 MHz, $CDCl_3$): δ = 8.87 (dd, *J* = 4.1, 1.7 Hz, 1H), 8.40 (dd, *J* = 8.0, 1.4 Hz, 1H), 8.25 (dd, *J* = 8.3, 1.7 Hz, 1H), 8.04 (d, *J* = 8.3 Hz, 1H), 7.99 (dd, *J* = 7.6, 2.1 Hz, 1H), 7.76 (ddd, *J* = 8.4, 7.1, 1.4 Hz, 1H), 7.72–7.68 (m, 2H), 7.52–7.48 (m, 1H), 7.45 (dd, *J* = 8.3, 4.1 Hz, 1H), 2.73 (ddd, *J* = 13.9, 11.6, 5.1 Hz, 1H), 2.17 (ddd, *J* = 13.9, 11.4, 5.2 Hz, 1H), 1.65 (d, *J* = 44.4 Hz, 1H), 1.49–1.35 (m, 2H), 1.11 (h, *J* = 7.4 Hz, 2H), 1.01–0.83 (m, 5H), 0.78 (t, *J* = 7.3 Hz, 3H).

^{13}**C NMR** (150 MHz, $CDCl_3$): δ = 162.90, 151.63, 144.65, 143.43, 137.05, 136.68, 136.43, 133.42, 130.25, 129.64, 129.47, 128.61, 127.00, 126.28, 126.12, 125.20, 122.12, 101.87, 34.56, 31.47, 29.26, 28.34, 27.69, 22.58, 14.12.

HR-MS (ESI): *m/z* calcd for $C_{25}H_{25}BrN_2O$ $[M+H]^+$ 449.1224, 451.1203, found 449.1223, 451.1205.

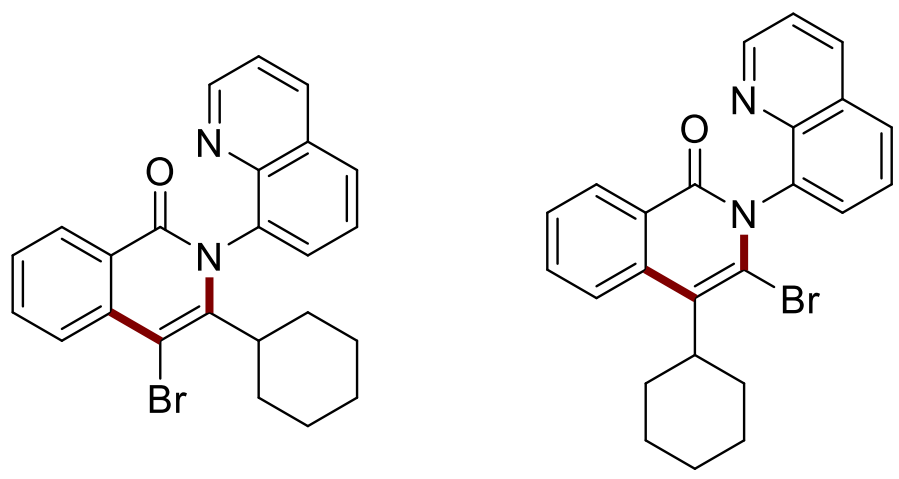

4-Bromo-3-cyclohexyl-2-(quinolin-8-yl)isoquinolin-1(2*H*)-one and **3-bromo-4-cyclohexyl-2-(quinolin-8-yl)isoquinolin-1(2H)-one (44 and 44')**

Prepared according to the general procedure **A** using **1a** (74.4 mg, 0.30 mmol) and **2u** (84.0 mg, 0.45 mmol), purification by column chromatography on silica gel (petroleum ether/EtOAc/DCM = 2:1:1) yielded **44** and **44'** as a mixture (56.0 mg, 43%，**44**/**44'** = 1:2.9).

^{1}**H NMR** (600 MHz, $CDCl_3$): δ = 8.87–8.86 (m, 3H), 8.86 (s, 1H), 8.48 (dd, *J* = 8.0, 1.6 Hz, 3H), 8.38 (dd, *J* = 7.9, 1.4 Hz, 1H), 8.27–8.25 (m, 1H), 8.24–8.22 (m, 6H), 8.15 (d, *J* = 8.4 Hz, 1H), 8.00–7.98 (m, 1H), 7.97 (dd, *J* = 7.7, 1.9 Hz, 3H), 7.76 (ddd, *J* = 8.5, 7.1, 1.4 Hz, 1H), 7.71 (d, *J* = 7.0 Hz, 1H), 7.70–7.65 (m, 9H), 7.61 (dd, *J* = 7.3, 1.4 Hz, 1H), 7.51 (s, 1H), 7.49 (td, *J* = 6.7, 3.4 Hz, 3H), 7.45 (dd, *J* = 7.5, 3.3 Hz, 1H), 7.43 (dd, *J* = 8.3, 4.2 Hz, 3H), 3.49 (d, *J* = 13.6 Hz, 3H), 2.54 (td, *J* = 12.6, 3.7 Hz, 1H), 2.51 –2.42 (m, 1H), 2.30–2.22 (m, 6H), 1.93 (dd, *J* = 16.0, 4.8 Hz, 12H), 1.91–1.86 (m, 1H), 1.65 (d, *J* = 13.1 Hz, 3H), 1.58–1.53 (m, 3H), 1.51–1.40 (m, 6H), 1.38 (d, *J* = 13.9 Hz, 2H), 1.28–1.24 (m, 2H), 1.16–1.06 (m, 1H), 0.92–0.81 (m, 1H), 0.63 (tdd, *J* = 13.3, 9.3, 4.0 Hz, 1H), 0.29 (dtd, *J* = 13.0, 9.4, 3.9 Hz, 1H).

^{13}**C NMR** (151 MHz, $CDCl_3$): δ = 162.86, 162.48, 151.35, 151.20, 145.73, 144.74, 140.20, 137.86, 137.01, 136.36, 136.20, 133.21, 131.89, 130.12, 129.35, 129.29, 129.26, 129.22, 129.06, 128.30, 127.07, 126.50, 126.44, 126.25, 126.17, 125.98, 125.18, 125.10, 122.85, 122.06, 121.79, 121.22, 101.13, 45.84, 45.18, 30.75, 30.59, 29.72, 28.06, 27.74, 27.47, 27.42, 26.73, 26.71, 26.12, 25.32.

HR-MS (ESI): *m/z* calcd for $C_{24}H_{21}BrN_2O$ $[M+H]^+$ 433.0911, 435.0890, found 433.0933, 435.0918.

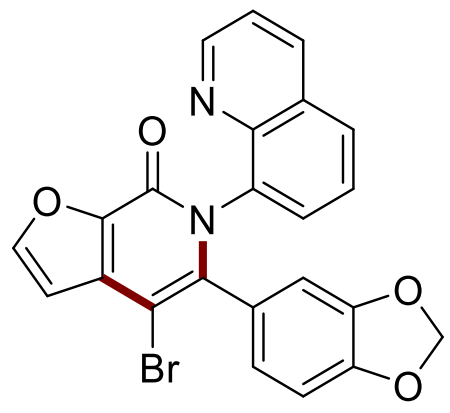

5-(Benzo[d][1,3]dioxol-5-yl)-4-bromo-6-(quinolin-8-yl)furo[2,3-c]pyridin-7(6*H*)-one (45)

Prepared according to the general procedure **A** using **1t** (71.4 mg, 0.30 mmol) and **2e** (101.3 mg, 0.45 mmol), purification by column chromatography on silica gel (petroleum ether/EtOAc/DCM = 1:2:1) yielded **45** (62.1 mg, 45%) as a colorless solid.

^{1}H NMR (600 MHz, $CDCl_3$, compound exists as a mixture of rotamers): δ = 8.89 (td, J = 4.4, 1.7 Hz, 2H), 8.09 (ddd, J = 7.9, 5.8, 1.7 Hz, 2H), 7.84 (d, J = 2.0 Hz, 2H), 7.73 (ddd, J = 7.6, 4.6, 1.8 Hz, 2H), 7.50–7.42 (m, 4H), 7.38 (dt, J = 7.9, 3.8 Hz, 2H), 6.84 (d, J = 2.0 Hz, 2H), 6.66–6.63 (m, 2H), 6.58 (d, J = 8.0 Hz, 1H), 6.36–6.33 (m, 2H), 6.19 (d, J = 8.1 Hz, 1H), 5.85 (d, J = 1.4 Hz, 1H), 5.82 (d, J = 1.4 Hz, 1H), 5.75 (d, J = 1.4 Hz, 1H), 5.66 (d, J = 1.4 Hz, 1H).

^{13}C NMR (151 MHz, CDCl3): δ = 153.4, 151.2, 151.1, 148.6, 147.6, 147.5, 146.9, 146.6, 144.5, 144.3, 142.8, 142.8, 142.1, 142.1, 136.9, 136.8, 136.3, 136.3, 135.1, 131.1, 130.8, 129.4, 129.0, 128.9, 128.7, 128.5, 125.9, 125.9, 124.5, 123.4, 121.9, 121.8, 110.6, 109.8, 108.8, 108.8, 107.6, 107.4, 101.3, 101.1, 95.1.

HR-MS (ESI): *m/z* calcd for $C_{23}H_{13}BrN_2O_4$ $[M+Na]^+$ 482.9951, 484.9931, found 482.9949, 484.9930.

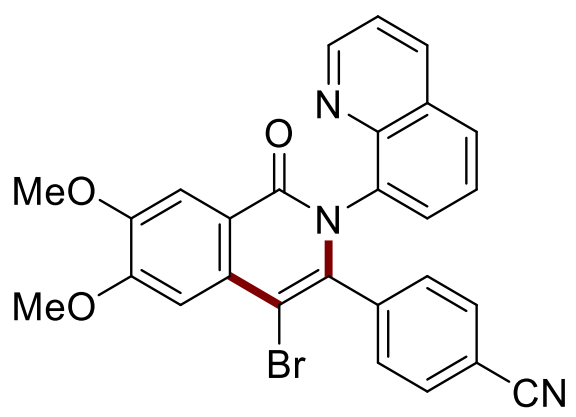

4-(4-Bromo-6,7-dimethoxy-1-oxo-2-(quinolin-8-yl)-1,2-dihydroisoquinolin-3-yl)benzonitrile (46)

Prepared according to the general procedure **A** using **1r** (92.4 mg, 0.30 mmol) and **2k** (92.7 mg, 0.45 mmol), purification by column chromatography on silica gel (petroleum ether/EtOAc/DCM = 1:1:1) yielded **46** (76.8 mg, 50%) as a colorless solid.

^{1}H NMR (600 MHz, $CDCl_3$): δ = 8.88 (dd, J = 4.2, 1.7 Hz, 1H), 8.08 (dd, J = 8.4, 1.7 Hz, 1H), 7.90 (s, 1H), 7.72 (dd, J = 8.2, 1.4 Hz, 1H), 7.48 (dd, J = 7.3, 1.4 Hz, 1H), 7.44 (s, 1H), 7.43 (dd, J = 8.3, 1.5 Hz, 1H), 7.42–7.35 (m, 3H), 7.08 (dd, J = 8.0, 1.7 Hz, 1H), 7.06 (dd, J = 8.0, 1.7 Hz, 1H), 4.09 (s, 3H), 4.01 (s, 3H).

^{13}C NMR (150 MHz, $CDCl_3$): δ = 161.4, 154.4, 151.2, 150.2, 144.2, 140.6, 139.4, 137.1, 136.3, 131.4, 131.3, 131.2, 131.1, 130.9, 130.2, 129.5, 128.9, 125.9, 122.0, 119.8, 118.3,

112.3, 108.8, 107.6, 101.6, 56.5, 56.4.

HR-MS (ESI): *m/z* calcd for $C_{27}H_{18}BrN_3O_3$ $[M+Na]^+$ 534.0424, 536.0404, found 534.0425, 536.0406.

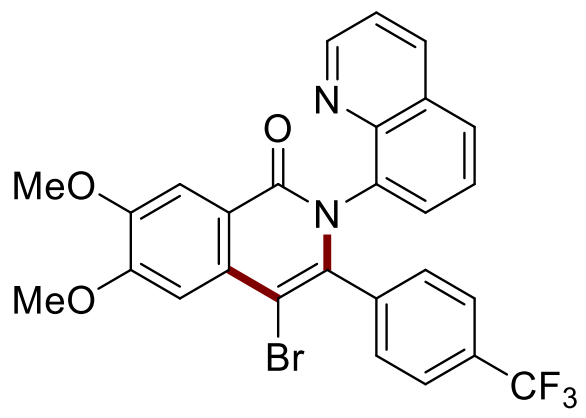

4-Bromo-6,7-dimethoxy-2-(quinolin-8-yl)-3-(4-(trifluoromethyl)phenyl)isoquinolin-1(2*H*)-one (47)

Prepared according to the general procedure **A** using **1r** (92.4 mg, 0.30 mmol) and **2j** (112.1 mg, 0.45 mmol), purification by column chromatography on silica gel (petroleum ether/EtOAc/DCM = 1:1:1) yielded **47** (99.9 mg, 60%) as a colorless solid.

^{1}H NMR (600 MHz, $CDCl_3$): δ = 8.90 (dd, J = 4.2, 1.7 Hz, 1H), 8.08 (dd, J = 8.3, 1.7 Hz, 1H), 7.92 (s, 1H), 7.70 (dd, J = 8.2, 1.4 Hz, 1H), 7.47 (s, 1H), 7.46 (dd, J = 7.4, 1.5 Hz, 1H), 7.44–7.34 (m, 4H), 7.06 (d, J = 8.1 Hz, 1H), 7.03 (dd, J = 8.2, 1.7 Hz, 1H), 4.10 (s, 3H), 4.01 (s, 3H).

^{13}C NMR (150 MHz, $CDCl_3$): δ = 161.6, 154.4, 151.2, 150.1, 144.4, 140.0, 139.8, 137.4, 136.3, 131.6, 130.8, 130.8, 130.4 (q, $^2J_{C-F}$ = 33.0 Hz), 129.7, 129.4, 128.9, 125.9, 124.6 (q, $^3J_{C-F}$ = 3.7 Hz), 124.3 (q, $^3J_{C-F}$ = 3.4 Hz), 123.7 (q, $^1J_{C-F}$ = 272.5 Hz), 121.9, 119.8, 108.9, 107.7, 101.8, 56.5, 56.4.

^{19}F NMR (565 MHz, CDCl3) δ -62.92.

HR-MS (ESI): *m/z* calcd for $C_{27}H_{18}BrF_3N_2O_3$ $[M+Na]^+$ 577.0345, 579.0325, found 577.0346, 579.0326.

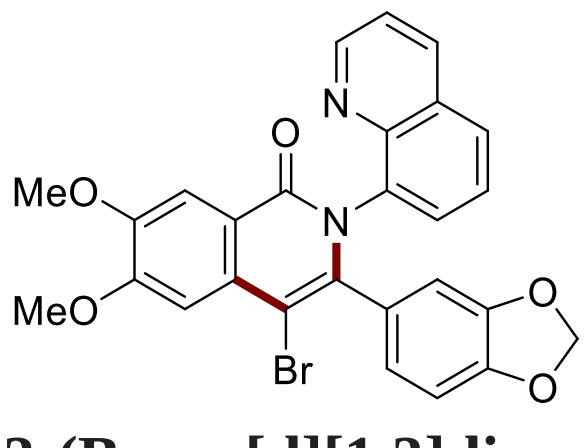

3-(Benzo[d][1,3]dioxol-5-yl)-4-bromo-6,7-dimethoxy-2-(quinolin-8-yl)isoquinolin-1(2*H*)-one (48)

Prepared according to the general procedure **A** using **1r** (92.4 mg, 0.30 mmol) and **2e** (101.3 mg, 0.45 mmol), purification by column chromatography on silica gel (petroleum ether/EtOAc/DCM = 1:1:1) yielded **48** (111.3 mg, 70%) as a colorless solid.

^{1}H NMR (600 MHz, $CDCl_3$, compound exists as a mixture of rotamers): δ = 8.89 (s, 2H), 8.12–8.07 (m, 2H), 7.90 (d, J = 1.4 Hz, 2H), 7.74–7.70 (m, 2H), 7.48 (d, J = 1.5

Hz, 2H), 7.46 (dd, J = 7.4, 3.1 Hz, 1H), 7.45–7.41 (m, 3H), 7.41–7.36 (m, 2H), 6.74 (d, J = 1.6 Hz, 1H), 6.72 (dd, J = 8.0, 1.7 Hz, 1H), 6.61 (d, J = 8.0 Hz, 1H), 6.39 (s, 1H), 6.37 (d, J = 8.4 Hz, 1H), 6.20 (d, J = 8.0 Hz, 1H), 5.87 (d, J = 1.4 Hz, 1H), 5.84 (d, J = 1.4 Hz, 1H), 5.75 (d, J = 1.5 Hz, 1H), 5.67 (d, J = 1.5 Hz, 1H), 4.09 (s, 6H), 4.00 (s, 6H).

^{13}C NMR (151 MHz, CDCl3): δ = 161.7, 154.3, 151.2, 151.1, 149.9, 147.4, 147.3, 146.9, 146.6, 144.6, 141.0, 140.9, 137.9, 137.8, 136.2, 131.9, 131.9, 130.9, 130.6, 130.1, 129.9, 129.0, 129.0, 128.9, 128.9, 126.0, 125.9, 124.5, 123.2, 121.8, 121.7, 119.7, 110.7, 109.8, 108.8, 107.7, 107.7, 107.6, 107.4, 102.4, 101.2, 101.0, 56.4, 56.4.

HR-MS (ESI): *m/z* calcd for $C_{27}H_{19}BrN_2O_5$ $[M+Na]^+$ 553.0370, 555.0350, found 553.0370, 555.0350.

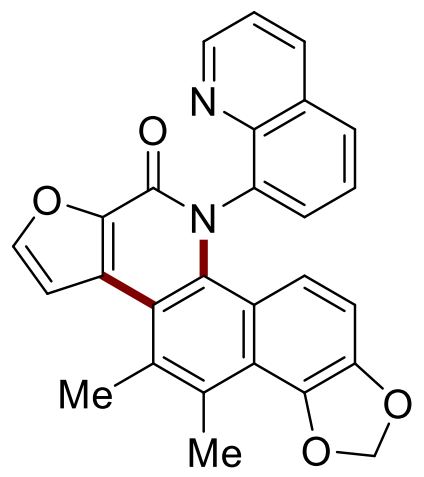

11,12-Dimethyl-6-(quinolin-8-yl)-[1,3]dioxolo[4',5':5,6]benzo[1,2-h]furo[2,3-*c*]quinolin-7(6*H*)-one (49)

Prepared according to the general procedure **C** using **45** (92.0 mg, 0.20 mmol) and but-2-yne (43.2 mg, 0.80 mmol), purification by column chromatography on silica gel (petroleum ether/EtOAc/DCM = 1:1:1) yielded **49** (38.2 mg, 44%) as a colorless solid.

^{1}H NMR (600 MHz, $CDCl_3$): δ = 8.96 (d, J = 4.5 Hz, 1H), 8.25 (d, J = 8.3 Hz, 1H), 7.87 (s, 1H), 7.85 (d, J = 8.3 Hz, 1H), 7.46 (dd, J = 8.3, 4.5 Hz, 1H), 7.43 (t, J = 8.1 Hz, 1H), 7.35 (s, 1H), 7.23 (d, J = 7.4 Hz, 1H), 7.14 (dd, J = 9.1, 1.8 Hz, 1H), 6.32 (dd, J = 9.1, 1.8 Hz, 1H), 5.98 (d, J = 19.0 Hz, 2H), 2.84 (s, 3H), 2.82 (s, 3H).

^{13}C NMR (150 MHz, $CDCl_3$): δ = 154.3, 151.3, 147.8, 145.4, 144.1, 143.1, 141.9, 141.8, 136.7, 136.6, 130.5, 130.4, 129.7, 129.1, 128.7, 126.5, 125.8, 121.9, 121.7, 121.1, 120.5, 115.2, 110.5, 107.5, 100.6, 18.9, 17.6.

HR-MS (ESI): *m/z* calcd for $C_{27}H_{18}N_2O_4$ $[M+Na]^+$ 457.1159, found 457.1157.

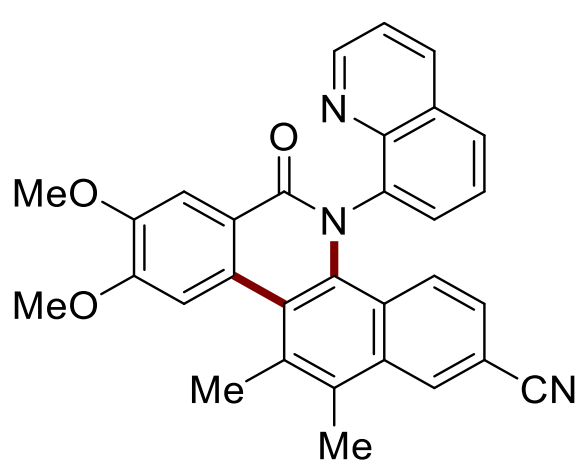

8,9-Dimethoxy-11,12-dimethyl-6-oxo-5-(quinolin-8-yl)-5,6-dihydrobenzo[*c*]phen-

anthridine-2-carbonitrile (50)

Prepared according to the general procedure **C** using **46** (102.4 mg, 0.20 mmol) and but-2-yne (43.2 mg, 0.80 mmol), purification by column chromatography on silica gel (petroleum ether/EtOAc/DCM = 1:1:1) yielded **50** (77.8 mg, 80%) as a colorless solid.

^{1}H NMR (600 MHz, $CDCl_3$): δ = 8.99 (d, J = 4.1 Hz, 1H), 8.32 (s, 1H), 8.31 (d, J = 8.4 Hz, 1H), 7.96 (s, 1H), 7.91 (d, J = 8.2 Hz, 1H), 7.55 (s, 1H), 7.52 (dd, J = 8.3, 4.1 Hz, 1H), 7.45 (t, J = 7.7 Hz, 1H), 7.34 (d, J = 9.0 Hz, 1H), 7.22 (d, J = 7.4 Hz, 1H), 6.81 (d, J = 9.0 Hz, 1H), 4.06 (s, 3H), 4.02 (s, 3H), 3.02 (s, 3H), 2.71 (s, 3H).

^{13}C NMR (150 MHz, $CDCl_3$): δ = 162.4, 152.3, 151.7, 149.8, 145.5, 141.5, 136.8, 135.1, 131.7, 130.8, 129.9, 129.6, 129.5, 129.3, 129.0, 128.4, 126.7, 126.1, 124.5, 123.9, 122.1, 121.7, 121.0, 119.4, 109.3, 109.2, 108.7, 56.4, 56.3, 22.5, 15.4.

HR-MS (ESI): *m/z* calcd for $C_{31}H_{23}N_3O_3$ $[M+Na]^+$ 508.1632, found 508.1632.

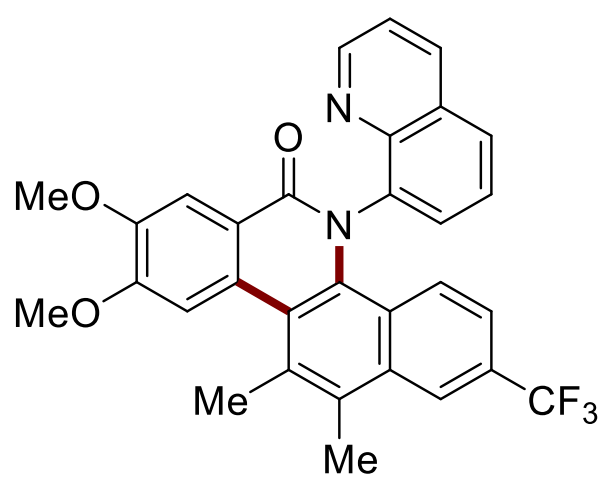

8,9-Dimethoxy-11,12-dimethyl-5-(quinolin-8-yl)-2-(trifluoromethyl)benzo[*c*]phenanthridin-6(5*H*)-one (51)

Prepared according to the general procedure **C** using **47** (111.0 mg, 0.20 mmol) and but-2-yne (43.2 mg, 0.80 mmol), purification by column chromatography on silica gel (petroleum ether/EtOAc/DCM = 1:1:1) yielded **51** (52.9 mg, 50%) as a colorless solid.

^{1}H NMR (600 MHz, $CDCl_3$): δ = 9.01 (d, J = 4.2 Hz, 1H), 8.31 (d, J = 8.5 Hz, 1H), 8.23 (s, 1H), 7.97 (s, 1H), 7.91 (d, J = 8.2 Hz, 1H), 7.56 (s, 1H), 7.52 (dd, J = 8.5, 4.2 Hz, 1H), 7.45 (t, J = 7.8 Hz, 1H), 7.36 (d, J = 9.2 Hz, 1H), 7.21 (d, J = 7.3 Hz, 1H), 6.85 (d, J = 9.2 Hz, 1H), 4.06 (s, 3H), 4.02 (s, 3H), 3.02 (s, 3H), 2.74 (s, 3H).

^{13}C NMR (150 MHz, $CDCl_3$): δ = 162.6, 152.2, 151.7, 149.6, 145.7, 141.9, 136.8, 135.2, 131.8, 130.1 (q, $^2J_{C-F}$ = 33.5 Hz), 129.8, 129.5, 129.3, 128.9, 128.8, 126.8, 126.1 (q, $^3J_{C-F}$ = 3.4 Hz), 124.4 (q, $^3J_{C-F}$ = 3.4 Hz), 123.7 (q, $^1J_{C-F}$ = 273.5 Hz), 122.1, 121.5, 120.9, 120.9, 119.0, 117.2, 109.3, 109.2, 56.4, 56.3, 22.5, 15.5.

^{19}F NMR (565 MHz, CDCl3) δ -62.35.

HR-MS (ESI): *m/z* calcd for $C_{31}H_{23}F_3N_2O_3$ $[M+Na]^+$ 551.1553, found 551.1552.

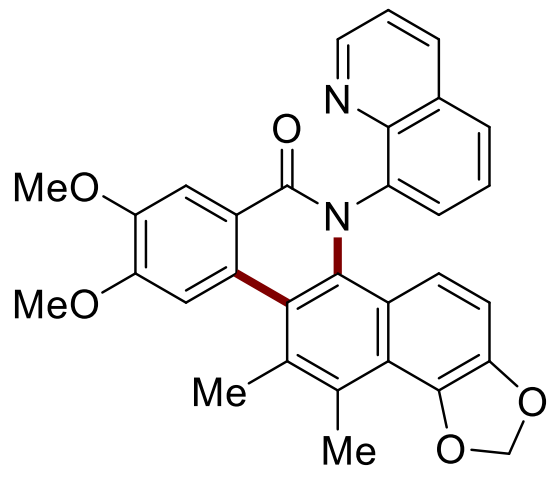

9,10-Dimethoxy-12,13-dimethyl-6-(quinolin-8-yl)-[1,3]dioxolo[4',5':5,6]benzo[1,-2-*c*]phenanthridin-7(6*H*)-one (52)

Prepared according to the general procedure **C** using **48** (106.2 mg, 0.20 mmol) and but-2-yne (43.2 mg, 0.80 mmol), purification by column chromatography on silica gel (petroleum ether/EtOAc/DCM = 1:1:1) yielded **52** (45.4 mg, 45%) as a colorless solid.

^{1}H NMR (600 MHz, $CDCl_3$): δ = 9.05 (d, J = 4.1 Hz, 1H), 8.29 (d, J = 8.3 Hz, 1H), 7.91 (s, 1H), 7.85 (d, J = 8.2 Hz, 1H), 7.52 (dd, J = 8.3, 4.1 Hz, 1H), 7.48 (s, 1H), 7.41 (t, J = 7.8 Hz, 1H), 7.17 (d, J = 7.3 Hz, 1H), 6.93 (d, J = 9.1 Hz, 1H), 6.34 (d, J = 9.1 Hz, 1H), 6.00 (d, J = 1.5 Hz, 2H), 4.04 (s, 3H), 3.99 (s, 3H), 2.89 (s, 3H), 2.81 (s, 3H).

^{13}C NMR (150 MHz, $CDCl_3$): δ = 162.8, 152.2, 151.5, 148.9, 144.0, 142.6, 141.7, 136.1, 130.3, 129.3, 129.3, 129.1, 128.4, 126.7, 125.7, 121.9, 120.9, 120.4, 120.2, 120.0, 118.6, 109.1, 108.9, 107.7, 100.6, 56.3, 56.2, 21.6, 17.2.

HR-MS (ESI): *m/z* calcd for $C_{31}H_{24}N_2O_5$ $[M+Na]^+$ 527.1577, found 527.1576.

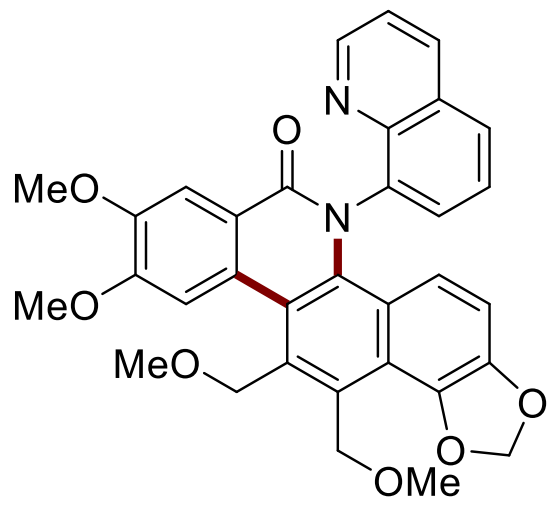

9,10-Dimethoxy-12,13-bis(methoxymethyl)-6-(quinolin-8-yl)-[1,3]dioxolo[4',-5':5,6]benzo[1,2-*c*]phenanthridin-7(6*H*)-one (53)

Prepared according to the general procedure **D** using **48** (106.2 mg, 0.20 mmol) and 1,2-diethoxyethyne (91.2 mg, 0.80 mmol), purification by column chromatography on silica gel (petroleum ether/EtOAc/DCM = 1:1:1) yielded **53** (45.1 mg, 40%) as a colorless solid.

^{1}H NMR (600 MHz, $CDCl_3$): δ = 9.01 (s, 1H), 8.27 (d, J = 8.4 Hz, 1H), 8.07 (d, J = 2.0 Hz, 1H), 7.91 (d, J = 1.9 Hz, 1H), 7.85 (d, J = 8.3 Hz, 1H), 7.50 (dd, J = 9.1, 4.1 Hz, 1H), 7.39 (t, J = 7.9 Hz, 1H), 7.18 (d, J = 7.4 Hz, 1H), 6.99 (d, J = 8.3 Hz, 1H), 6.41 (dd, J = 9.1, 1.9 Hz, 1H), 6.08 (s, 1H), 6.04 (s, 1H), 5.31 (d, J = 10.0 Hz, 1H), 4.99 (d, J = 10.9 Hz, 1H), 4.89–4.82 (m, 2H), 4.08 (s, 3H), 4.00 (s, 3H), 3.74 (s, 3H), 3.54 (s, 3H).

^{13}C NMR (150 MHz, $CDCl_3$): δ = 162.8, 153.2, 151.5, 149.3, 145.6, 144.4, 142.2, 141.8, 138.0, 136.8, 130.6, 129.9, 129.7, 129.1, 128.6, 128.4, 126.6, 121.9, 121.3, 120.6, 119.9, 119.8, 117.6, 109.1, 108.9, 108.3, 101.0, 71.4, 68.3, 59.2, 58.7, 56.3, 56.1.

HR-MS (ESI): *m/z* calcd for $C_{33}H_{28}N_2O_7$ $[M+Na]^+$ 587.1789, found 587.1788.

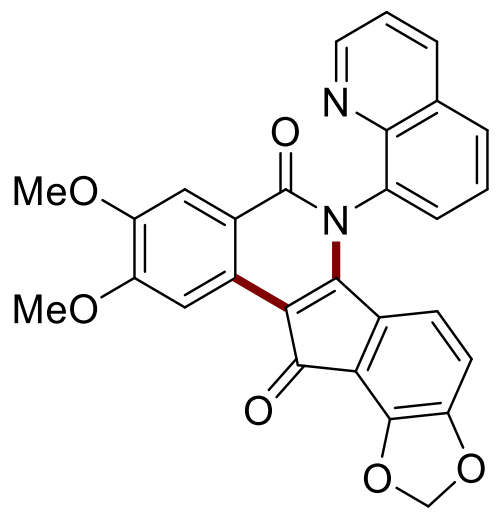

9,10-Dimethoxy-6-(quinolin-8-yl)-6*H*-[1,3]dioxolo[4',5':4,5]indeno[1,2-*c*]isoquin-oline-7,12-dione (52)
Prepared according to the general procedure **B** using **48** (106.2 mg, 0.20 mmol) and CO atmosphere, purification by column chromatography on silica gel (petroleum ether/EtOAc/DCM = 1:1:1) yielded **54** (86.0 mg, 90%) as a colorless solid.

^{1}H NMR (400 MHz, d_6-DMSO): δ = 8.80 (d, J = 4.2 Hz, 1H), 8.56 (d, J = 8.4 Hz, 1H), 8.32 (d, J = 8.3 Hz, 1H), 8.12 (d, J = 7.3 Hz, 1H), 8.06 (s, 1H), 7.89 (t, J = 7.8 Hz, 1H), 7.61 (dd, J = 8.4, 4.1 Hz, 1H), 7.52 (s, 1H), 6.25 (d, J = 7.9 Hz, 1H), 6.09 (s, 2H), 4.63 (d, J = 7.9 Hz, 1H), 3.97 (s, 3H), 3.85 (s, 3H).

^{13}C NMR (101 MHz, d_6-DMSO): δ = 186.5, 161.5, 154.6, 151.8, 151.4, 148.7, 146.0, 143.9, 143.5, 136.3, 134.7, 130.2, 130.0, 128.6, 128.6, 127.9, 126.3, 122.2, 116.8, 115.9, 113.6, 108.4, 108.1, 106.5, 103.2, 103.0, 55.6, 55.4.

HR-MS (ESI): *m/z* calcd for $C_{28}H_{18}N_2O_6$ $[M+Na]^+$ 501.1057, found 501.1056.

10. Reaction with TIPS-substituted bromoalkyne

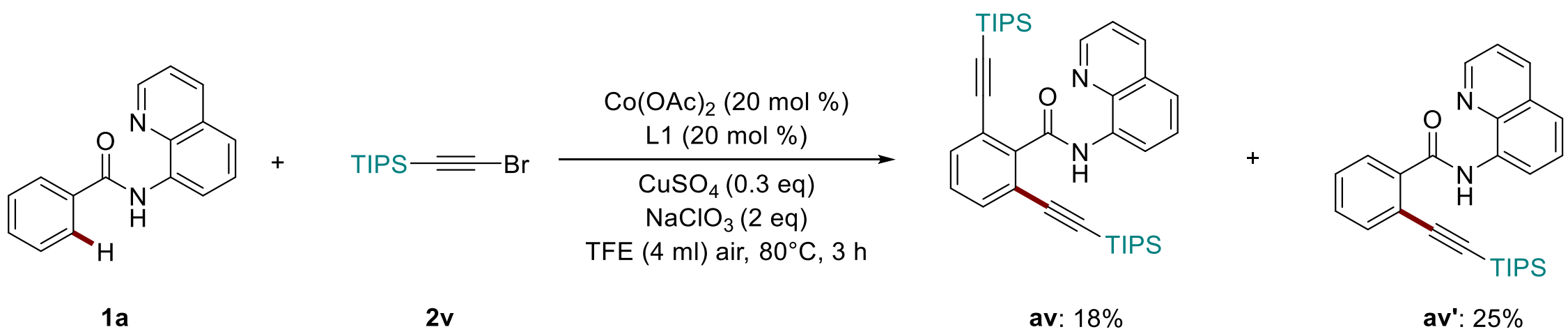

Prepared according to the general procedure **A** using **1a** (74.4 mg, 0.30 mmol) and **2v** (117.0 mg, 0.45 mmol), purification by column chromatography on silica gel (petroleum ether/EtOAc = 10:1) yielded **av** (33.0 mg, 18%) as a colorless solid and **av'** (32.1 mg, 25%) as a colorless solid.

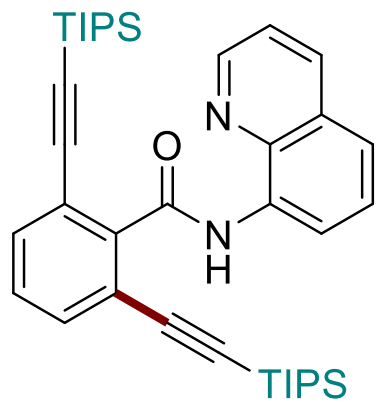

***N*-(quinolin-8-yl)-2,6-bis((triisopropylsilyl)ethynyl)benzamide (av):**

^{1}H NMR (600 MHz, $CDCl_3$): δ = 10.04 (s, 1H), 8.97 (dd, J = 7.5, 1.4 Hz, 1H), 8.72 (dd, J = 4.2, 1.7 Hz, 1H), 8.15 (dd, J = 8.2, 1.7 Hz, 1H), 7.58–7.48 (m, 4H), 7.41 (dd, J = 8.3, 4.2 Hz, 1H), 7.32 (t, J = 7.9 Hz, 1H), 0.85 (s, 42H).

^{13}C NMR (151 MHz, $CDCl_3$): δ = 165.7, 148.1, 138.7, 136.2, 135.0, 132.5, 128.9, 127.4, 121.6, 121.6, 121.5, 117.2, 103.2, 96.0, 18.5, 11.2.

HR-MS (ESI): *m/z* calcd for $C_{38}H_{52}N_2OSi_2$ $[M+H]^+$ 609.3691, found 609.3688.

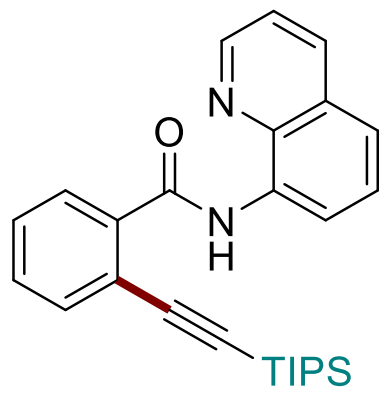

***N*-(quinolin-8-yl)-2-((triisopropylsilyl)ethynyl)benzamide (av')**

^{1}H NMR (600 MHz, $CDCl_3$): δ = 10.50 (s, 1H), 8.93 (dd, J = 7.6, 1.3 Hz, 1H), 8.76 (dd, J = 4.2, 1.7 Hz, 1H), 8.16 (dd, J = 8.2, 1.7 Hz, 1H), 7.80 (dd, J = 5.8, 3.3 Hz, 1H), 7.63 (dd, J = 5.8, 3.4 Hz, 1H), 7.55 (ddd, J = 15.2, 8.5, 2.2 Hz, 2H), 7.46–7.41 (m, 3H), 0.83 (d, J = 5.1 Hz, 21H).

^{13}C NMR (151 MHz, $CDCl_3$): δ = 166.6, 148.4, 139.4, 139.0, 136.3, 134.8, 134.0, 130.3, 128.82, 128.1, 127.6, 127.5, 122.0, 121.6, 120.9, 117.3, 104.0, 97.3, 18.5, 11.2.

HR-MS (ESI): *m/z* calcd for $C_{27}H_{33}N_2OSi$ $[M+H]^+$ 429.2357, found 429.2357.

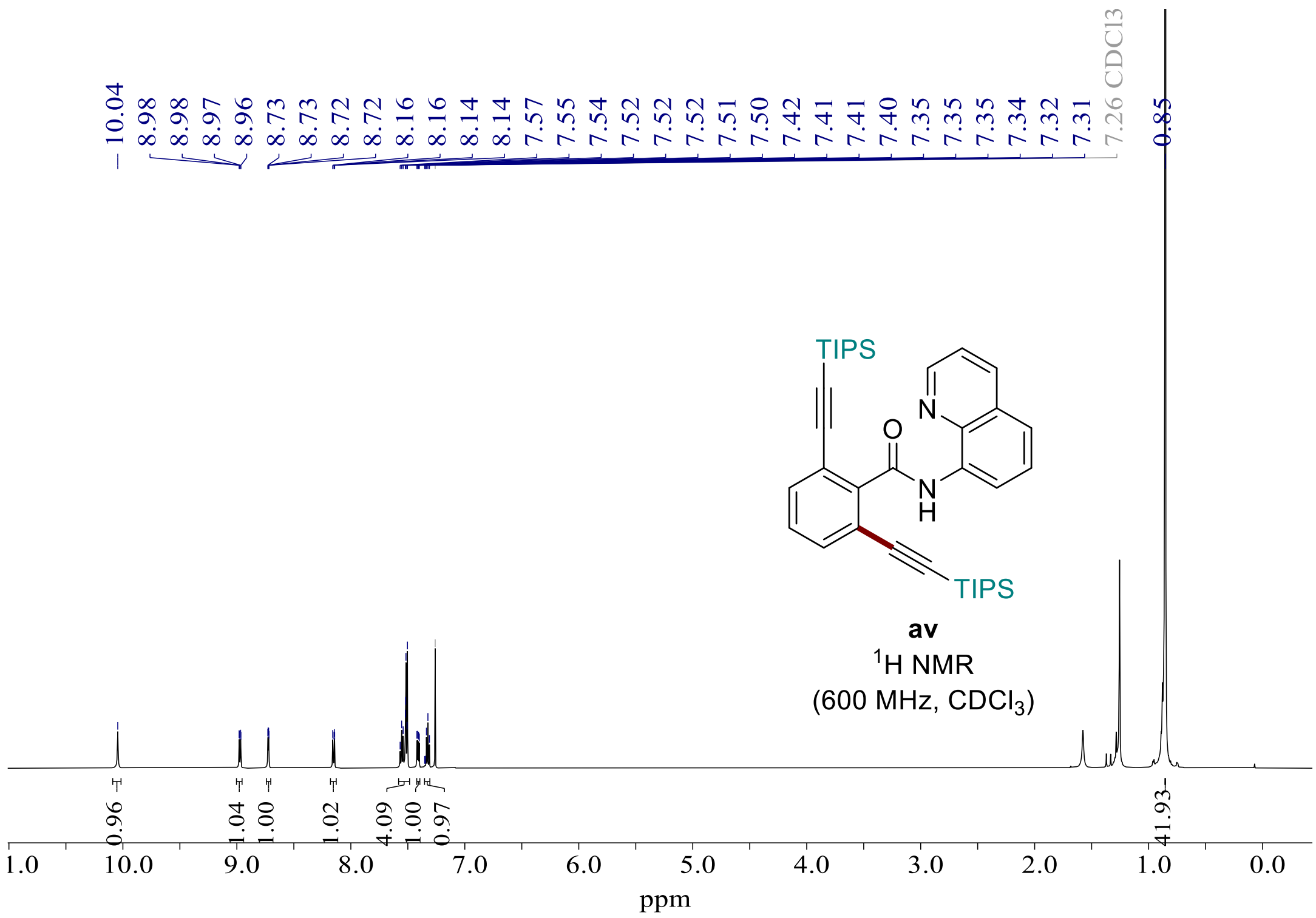

10.04
8.98
8.98
8.97
8.96
8.73
8.73
8.72
8.72
8.16
8.16
8.14
8.14
7.57
7.55
7.54
7.52
7.52
7.52
7.51
7.50
7.42
7.41
7.41
7.40
7.35
7.35
7.35
7.34
7.32
7.31
7.26 CDCl3
0.85
TIPS
N
O
N
H
TIPS
av
1H NMR
(600 MHz, CDCl3)
0.96
1.04
1.00
1.02
4.09
1.00
0.97
41.93
1.0
10.0
9.0
8.0
7.0
6.0
5.0
4.0
3.0
2.0
1.0
0.0
ppm

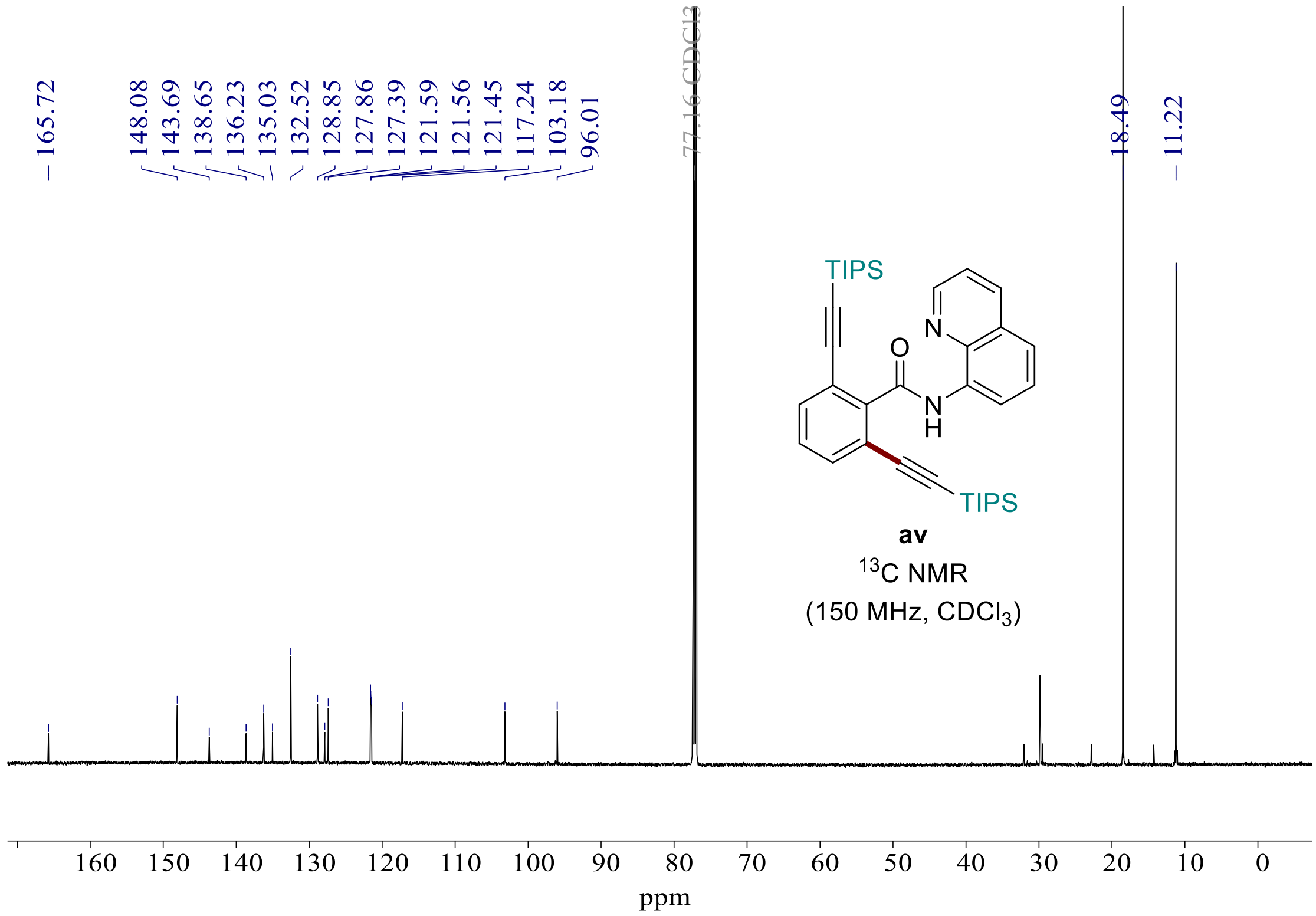

165.72
148.08
143.69
138.65
136.23
135.03
132.52
128.85
127.86
127.39
121.59
121.56
121.45
117.24
103.18
96.01
77.16 CDCl3
18.49
11.22
TIPS
N
O
N
H
TIPS
av
13C NMR
(150 MHz, CDCl3)
160
150
140
130
120
110
100
90
80
70
60
50
40
30
20
10
0
ppm

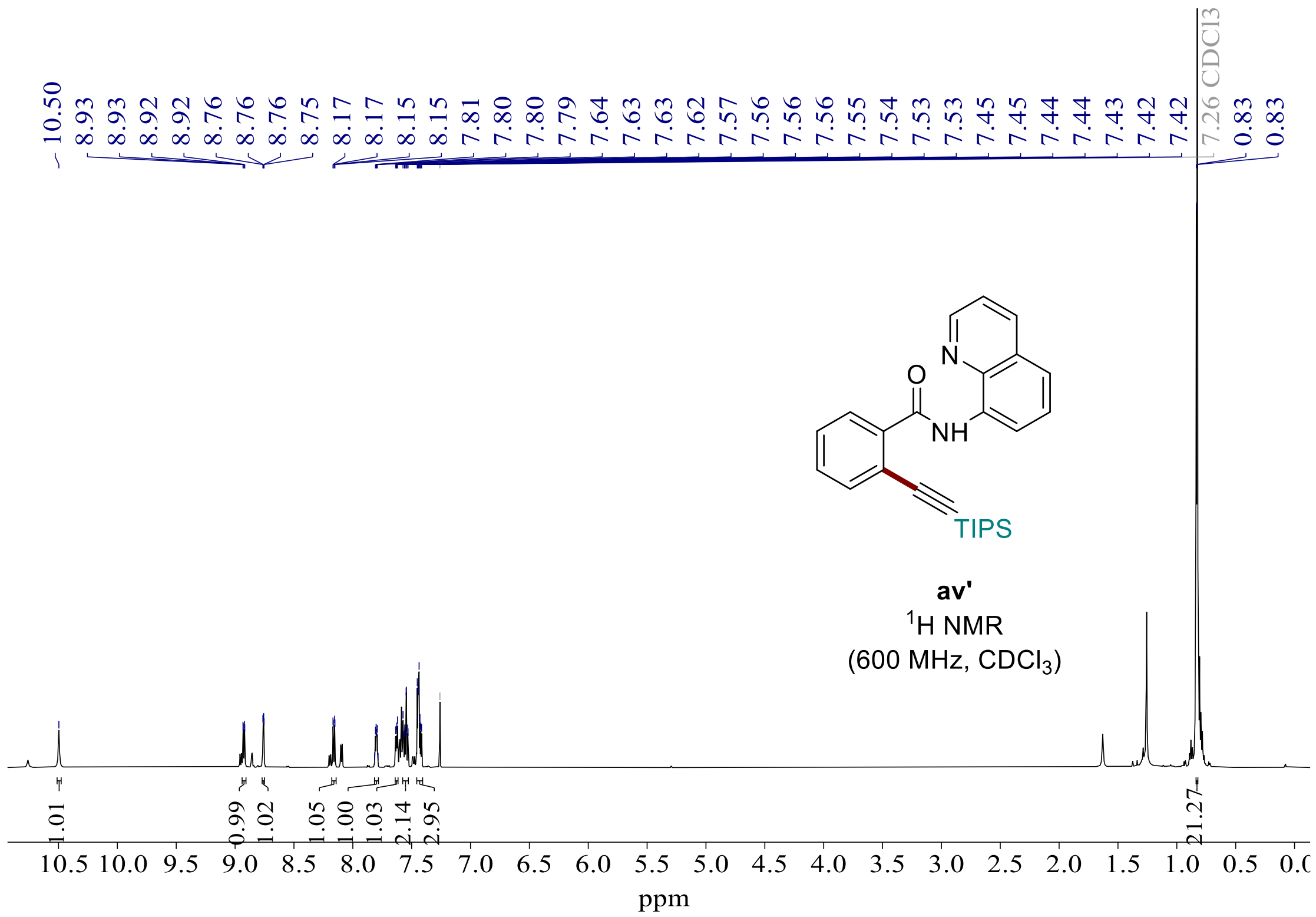

TIPS
av'
1H NMR
(600 MHz, CDCl3)
ppm

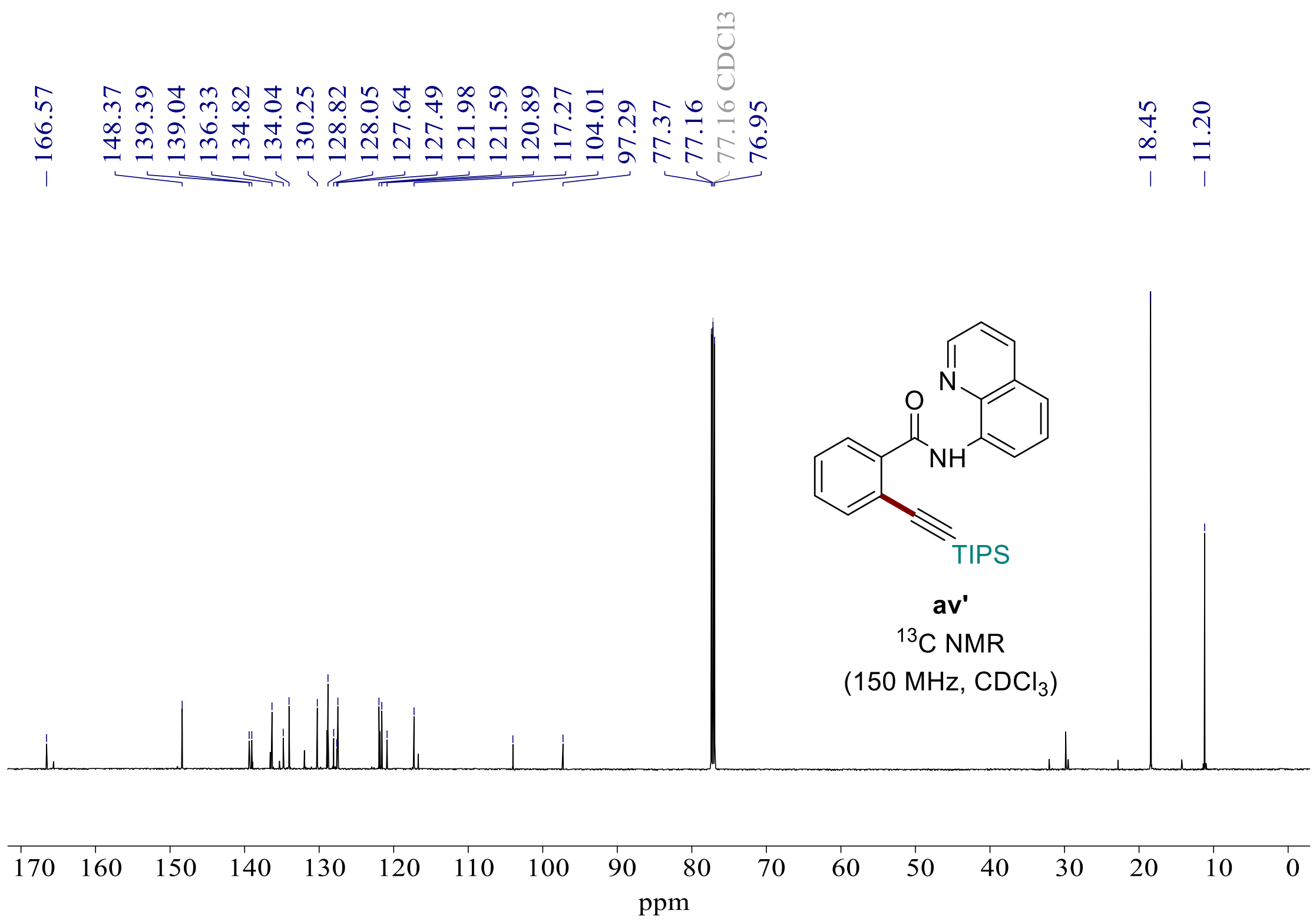

TIPS
av'
13C NMR
(150 MHz, CDCl3)
ppm

11. References

[1] Y. He, N. Zhao, L. Qiu, X. Zhang, X. Fan, *Org. Lett.* **2016**, *18* , 6054–6057.

[2] G. Wang, J. Han, K. Wang, H. Li, G. Duan, C. Xia, F. Li, *Catal. Commun.* **2018**, *114*, 37–41.

[3] T. Yao, M. A. Campo, R. C. Larock, *Org. Lett.* **2004**, *6*, 2677–2680.

[4] Zhong, H.; Cai, S.; Hu, S.; Liu, Y.; Gao, Y.; Chen, Q.; Huo, Y.; Li, X., *ACS Catal.* **2025**, 15, 19994−20003.

[5] Hao, X.-Q.; Du, C.; Zhu, X.; Li, P.-X.; Zhng, J.-H.; Niu, J.-L.; Song, M.-P., *Org. Lett.* **2016**, *18*, 3610–3613.

12. NMR Spectra

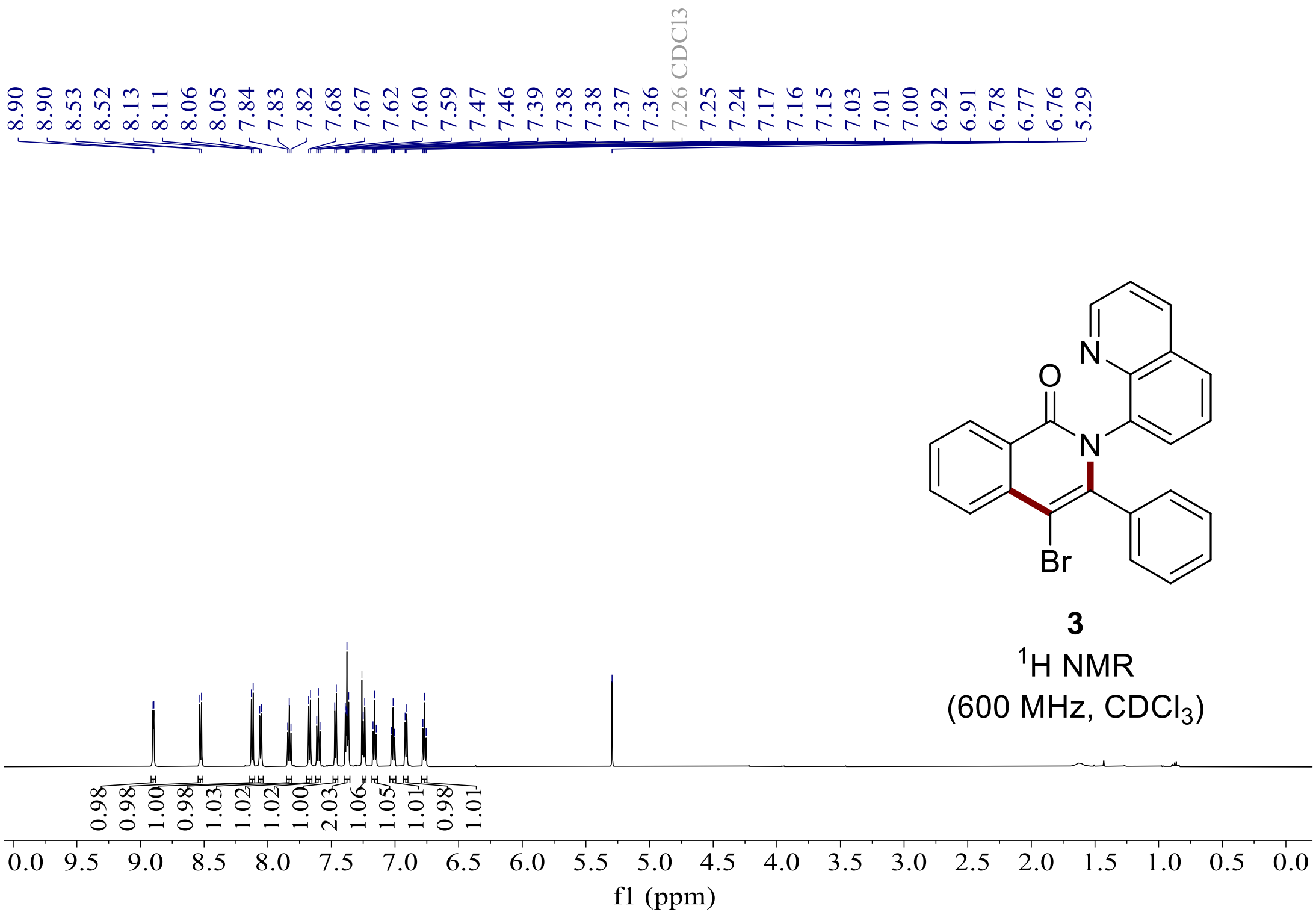

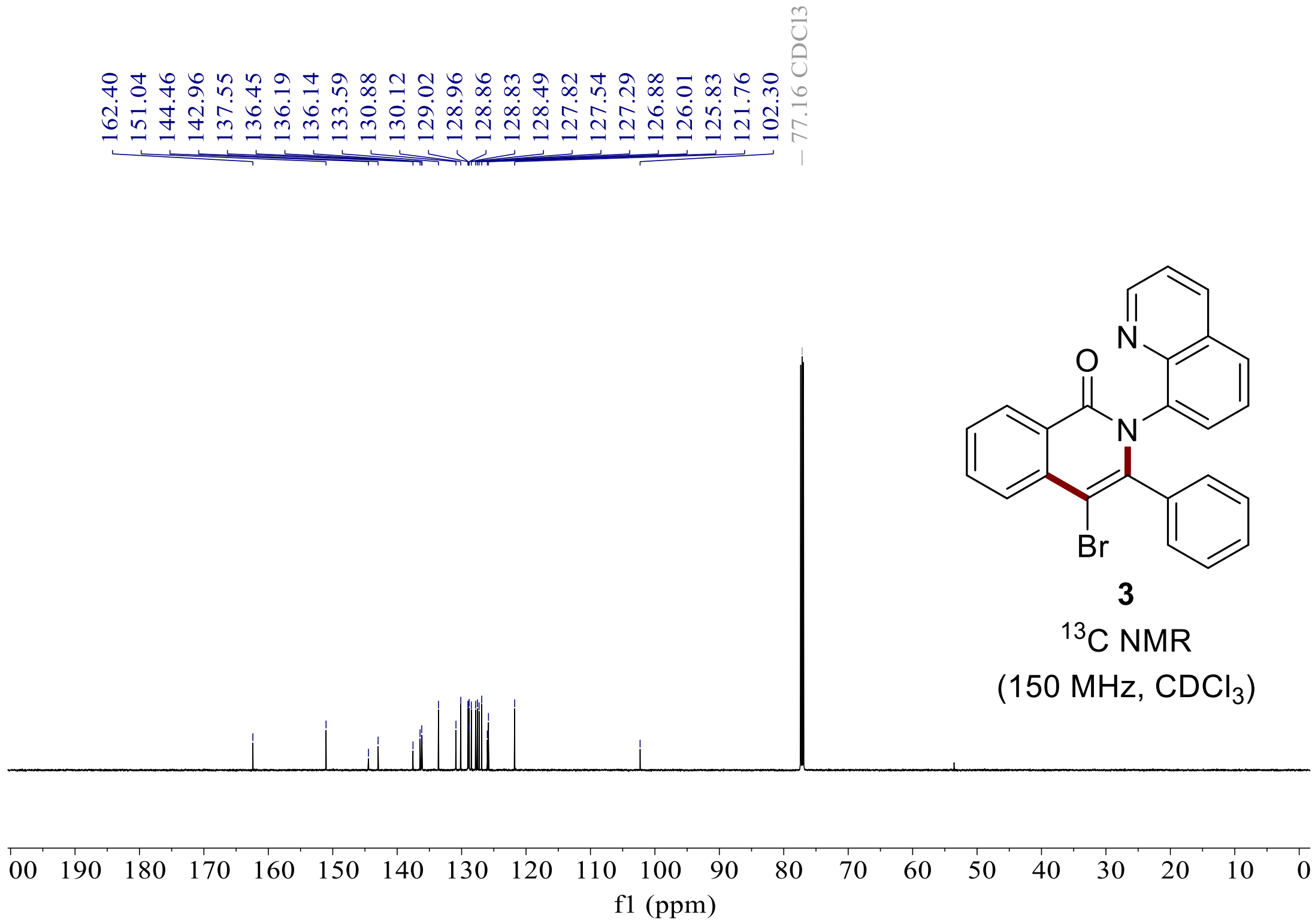
162.40
151.04
144.46
142.96
137.55
136.45
136.19
136.14
133.59
130.88
130.12
129.02
128.96
128.86
128.83
128.49
127.82
127.54
127.29
126.88
126.01
125.83
121.76
102.30
77.16 CDCl3
O
N
N
Br
3
13C NMR
(150 MHz, CDCl3)
00 190 180 170 160 150 140 130 120 110 100 90 80 70 60 50 40 30 20 10 0
f1 (ppm)

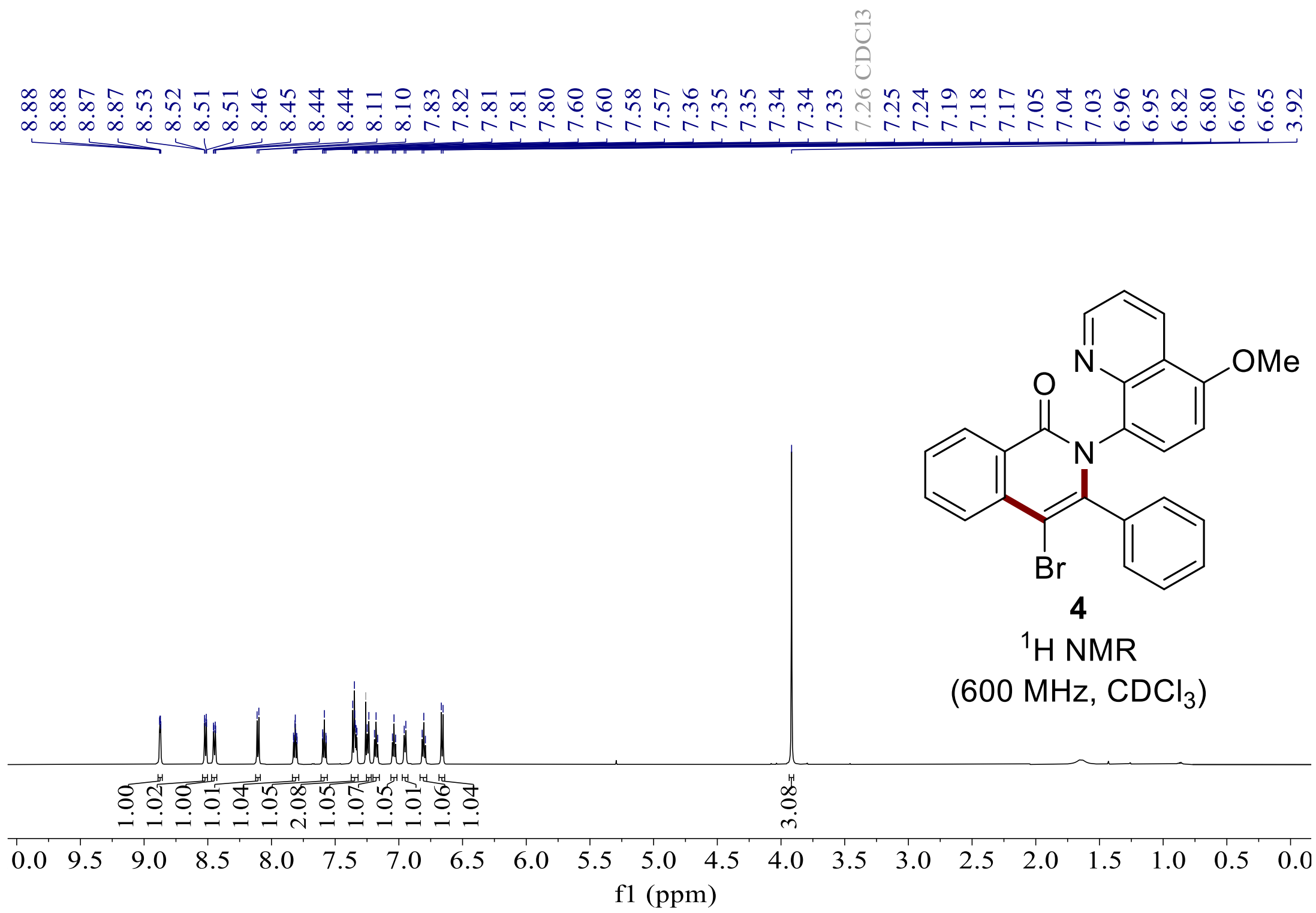
8.88
8.88
8.87
8.87
8.53
8.52
8.51
8.51
8.46
8.45
8.44
8.44
8.11
8.10
7.83
7.82
7.81
7.81
7.80
7.60
7.60
7.58
7.57
7.36
7.35
7.35
7.34
7.34
7.33
7.26 CDCl3
7.25
7.24
7.19
7.18
7.17
7.05
7.04
7.03
6.96
6.95
6.82
6.80
6.67
6.65
3.92
N
OMe
O
N
Br
4
1H NMR
(600 MHz, CDCl3)
1.00
1.02
1.00
1.01
1.04
1.05
2.08
1.05
1.07
1.05
1.01
1.06
1.04
3.08
0.0 9.5 9.0 8.5 8.0 7.5 7.0 6.5 6.0 5.5 5.0 4.5 4.0 3.5 3.0 2.5 2.0 1.5 1.0 0.5 0.0
f1 (ppm)

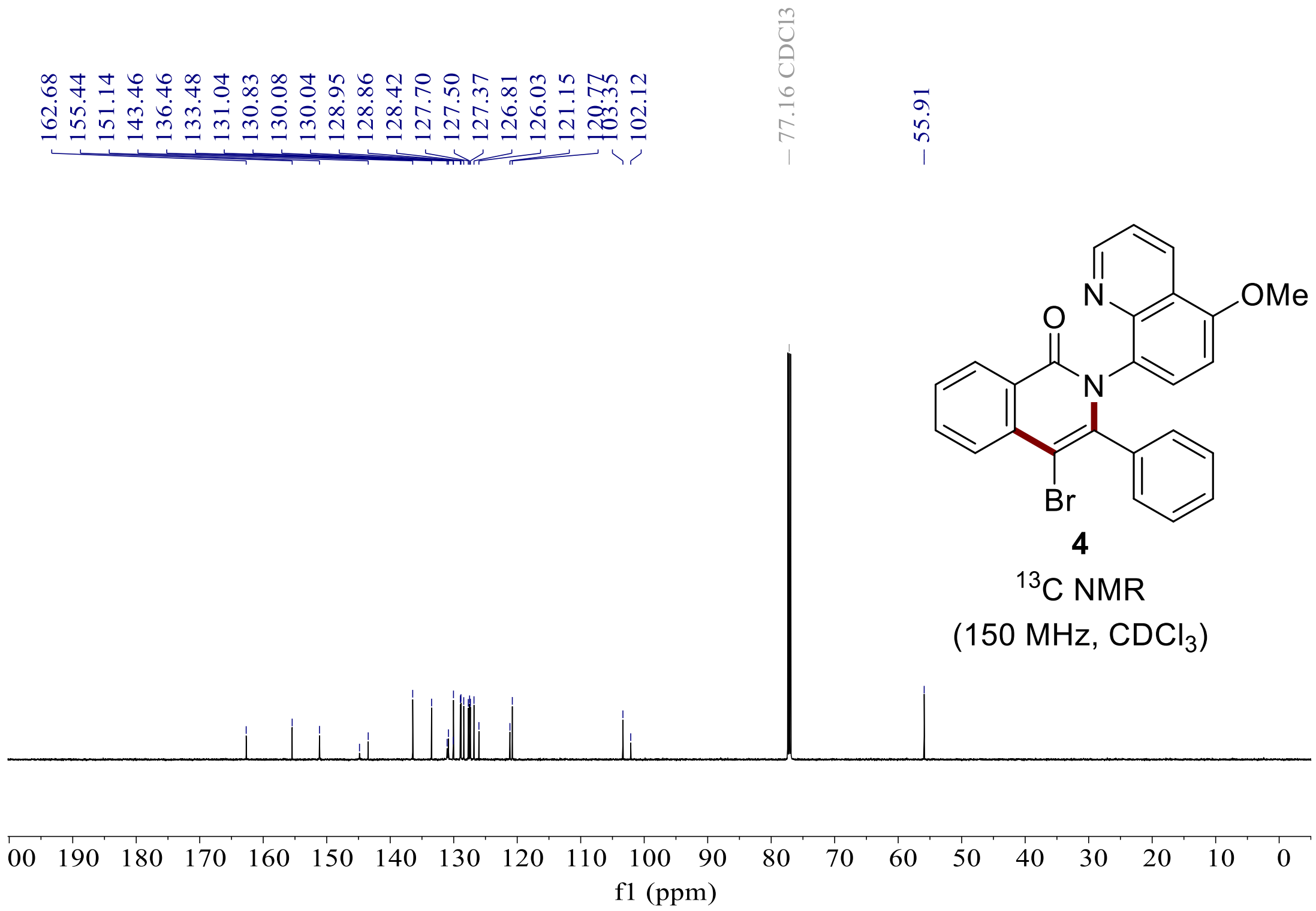
162.68
155.44
151.14
143.46
136.46
133.48
131.04
130.83
130.08
130.04
128.95
128.86
128.42
127.70
127.50
127.37
126.81
126.03
121.15
120.77
103.35
102.12
77.16 CDCl3
55.91
N
OMe
O
N
Br
4
13C NMR
(150 MHz, CDCl3)
00 190 180 170 160 150 140 130 120 110 100 90 80 70 60 50 40 30 20 10 0
f1 (ppm)

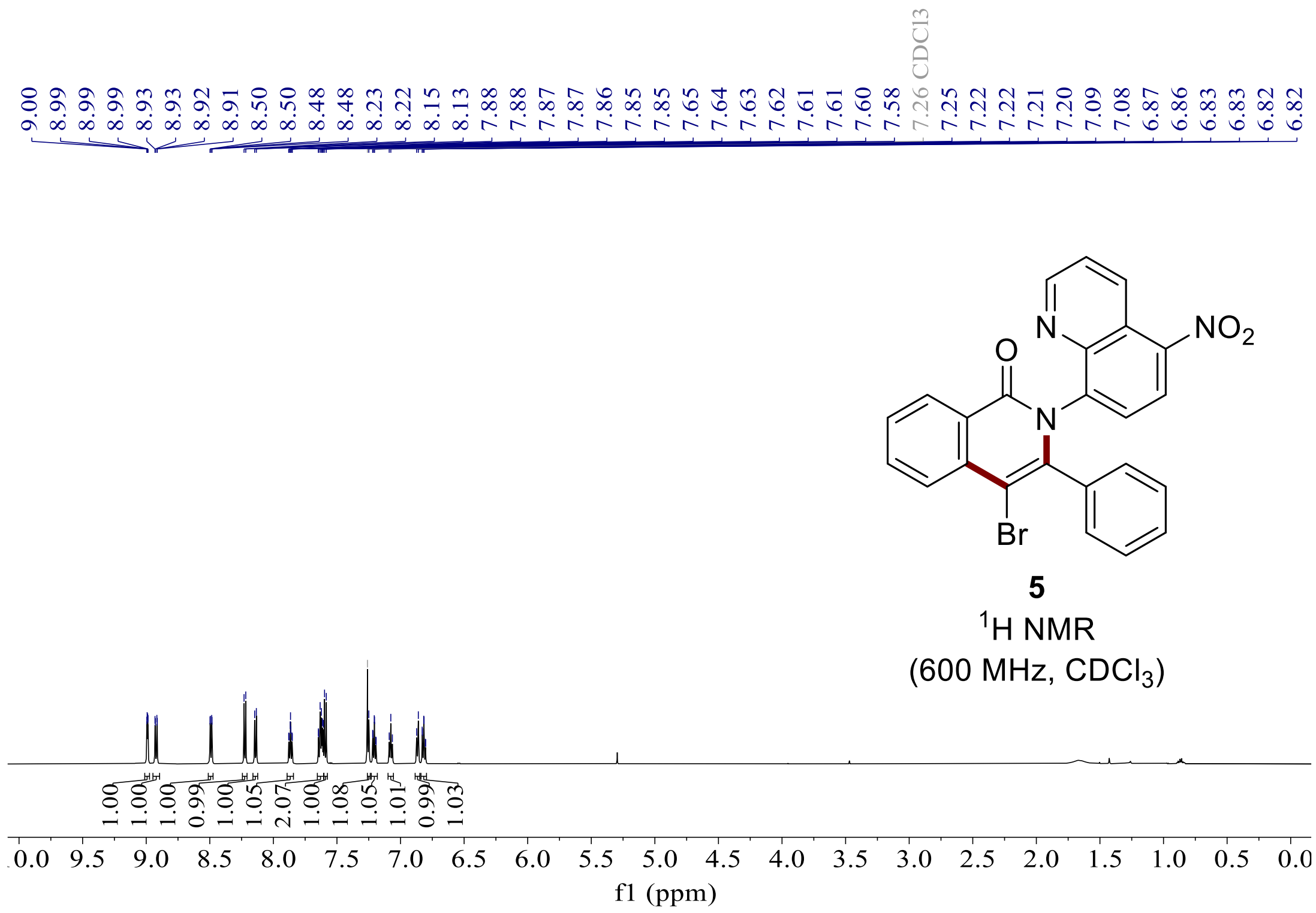
9.00
8.99
8.99
8.99
8.93
8.93
8.92
8.91
8.50
8.50
8.48
8.48
8.23
8.22
8.15
8.13
7.88
7.88
7.87
7.87
7.86
7.85
7.85
7.65
7.64
7.63
7.62
7.61
7.61
7.60
7.58
7.26 CDCl3
7.25
7.22
7.22
7.21
7.20
7.09
7.08
6.87
6.86
6.83
6.83
6.82
6.82
N
NO2
O
N
Br
5
1H NMR
(600 MHz, CDCl3)
1.00
1.00
1.00
0.99
1.00
1.05
2.07
1.00
1.08
1.05
1.01
0.99
1.03
.0.0 9.5 9.0 8.5 8.0 7.5 7.0 6.5 6.0 5.5 5.0 4.5 4.0 3.5 3.0 2.5 2.0 1.5 1.0 0.5 0.0
f1 (ppm)

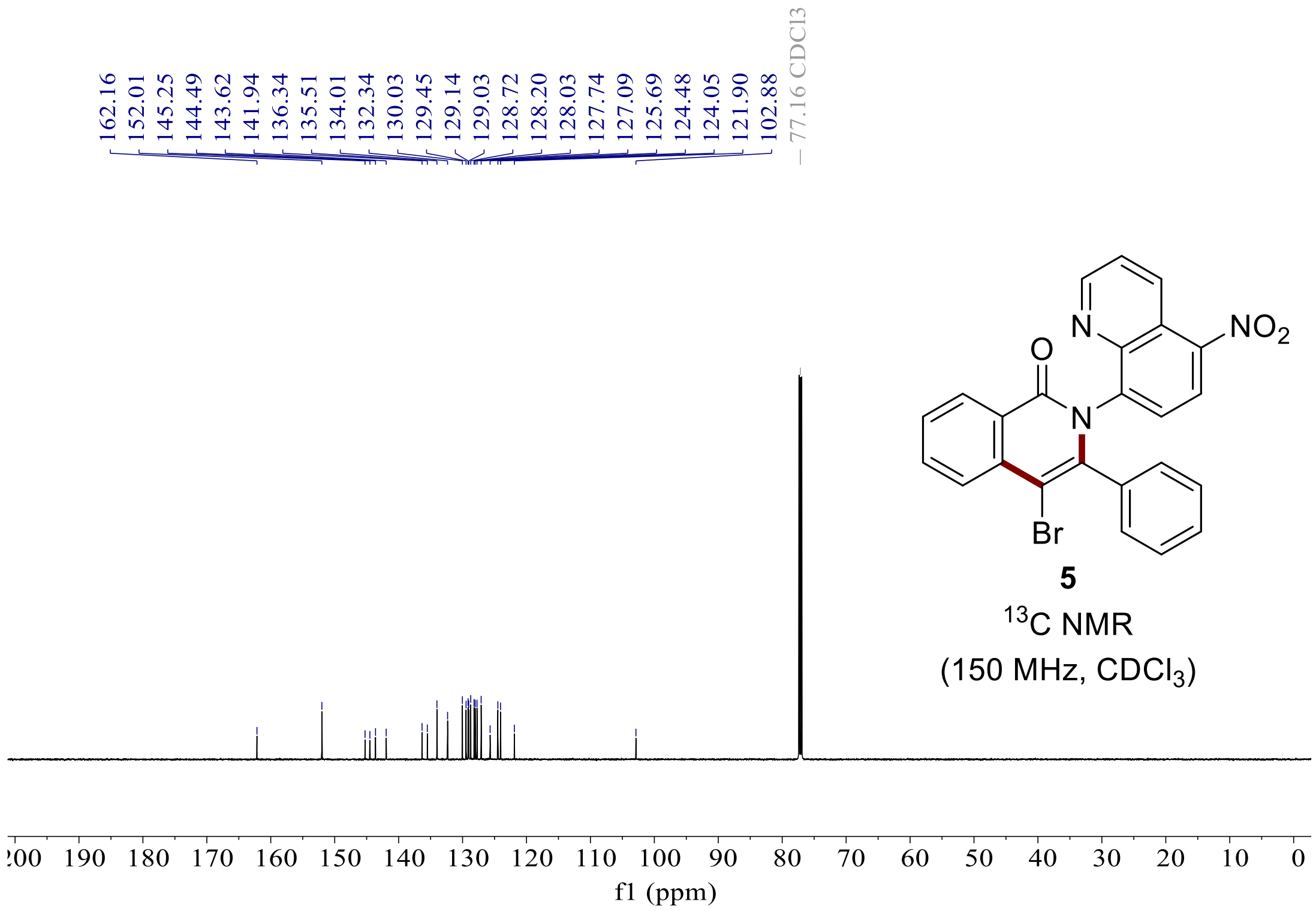
162.16
152.01
145.25
144.49
143.62
141.94
136.34
135.51
134.01
132.34
130.03
129.45
129.14
129.03
128.72
128.20
128.03
127.74
127.09
125.69
124.48
124.05
121.90
102.88
77.16 CDCl3
N
NO2
O
N
Br
5
13C NMR
(150 MHz, CDCl3)
200 190 180 170 160 150 140 130 120 110 100 90 80 70 60 50 40 30 20 10 0
f1 (ppm)

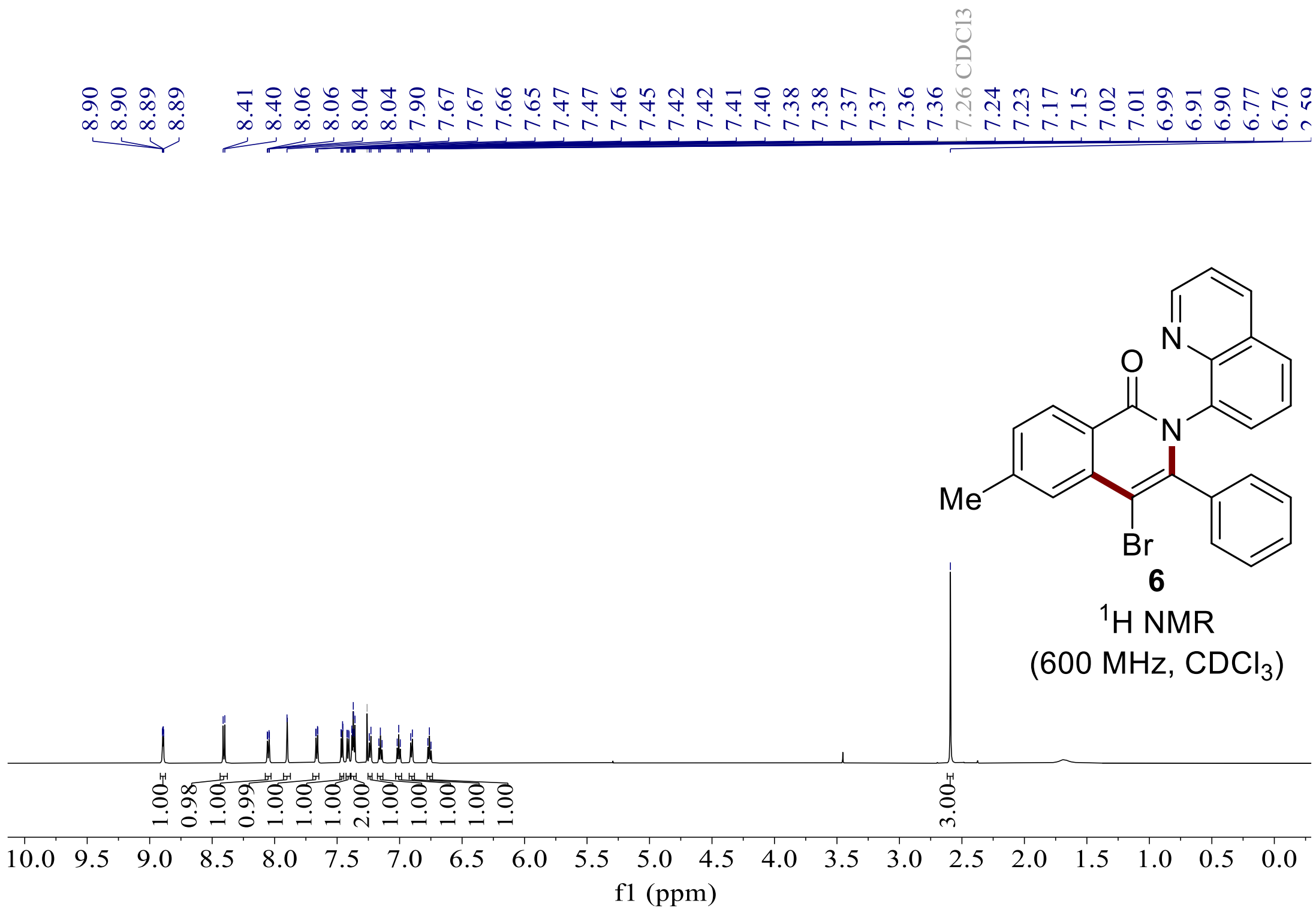
8.90
8.90
8.89
8.89
8.41
8.40
8.06
8.06
8.04
8.04
7.90
7.67
7.67
7.66
7.65
7.47
7.47
7.46
7.45
7.42
7.42
7.41
7.40
7.38
7.38
7.37
7.37
7.36
7.36
7.26 CDCl3
7.24
7.23
7.17
7.15
7.02
7.01
6.99
6.91
6.90
6.77
6.76
N
O
N
Me
Br
6
1H NMR
(600 MHz, CDCl3)
1.00
0.98
1.00
0.99
1.00
1.00
1.00
2.00
1.00
1.00
1.00
1.00
1.00
3.00
10.0 9.5 9.0 8.5 8.0 7.5 7.0 6.5 6.0 5.5 5.0 4.5 4.0 3.5 3.0 2.5 2.0 1.5 1.0 0.5 0.0
f1 (ppm)

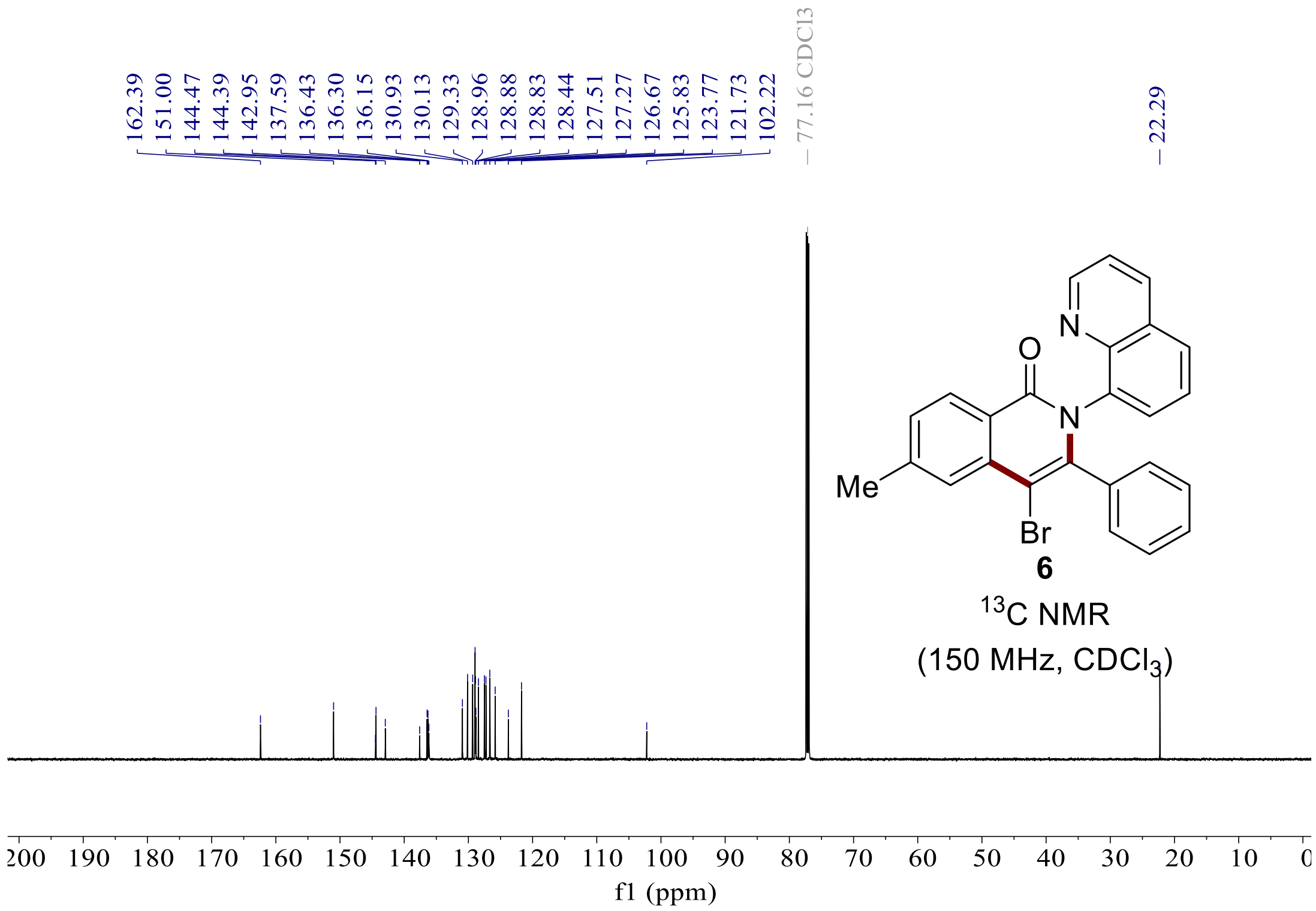
162.39
151.00
144.47
144.39
142.95
137.59
136.43
136.30
136.15
130.93
130.13
129.33
128.96
128.88
128.83
128.44
127.51
127.27
126.67
125.83
123.77
121.73
102.22
77.16 CDCl3
22.29
O
N
N
Me
Br
6
^{13}C NMR
(150 MHz, $CDCl_3$)
200 190 180 170 160 150 140 130 120 110 100 90 80 70 60 50 40 30 20 10 0
f1 (ppm)

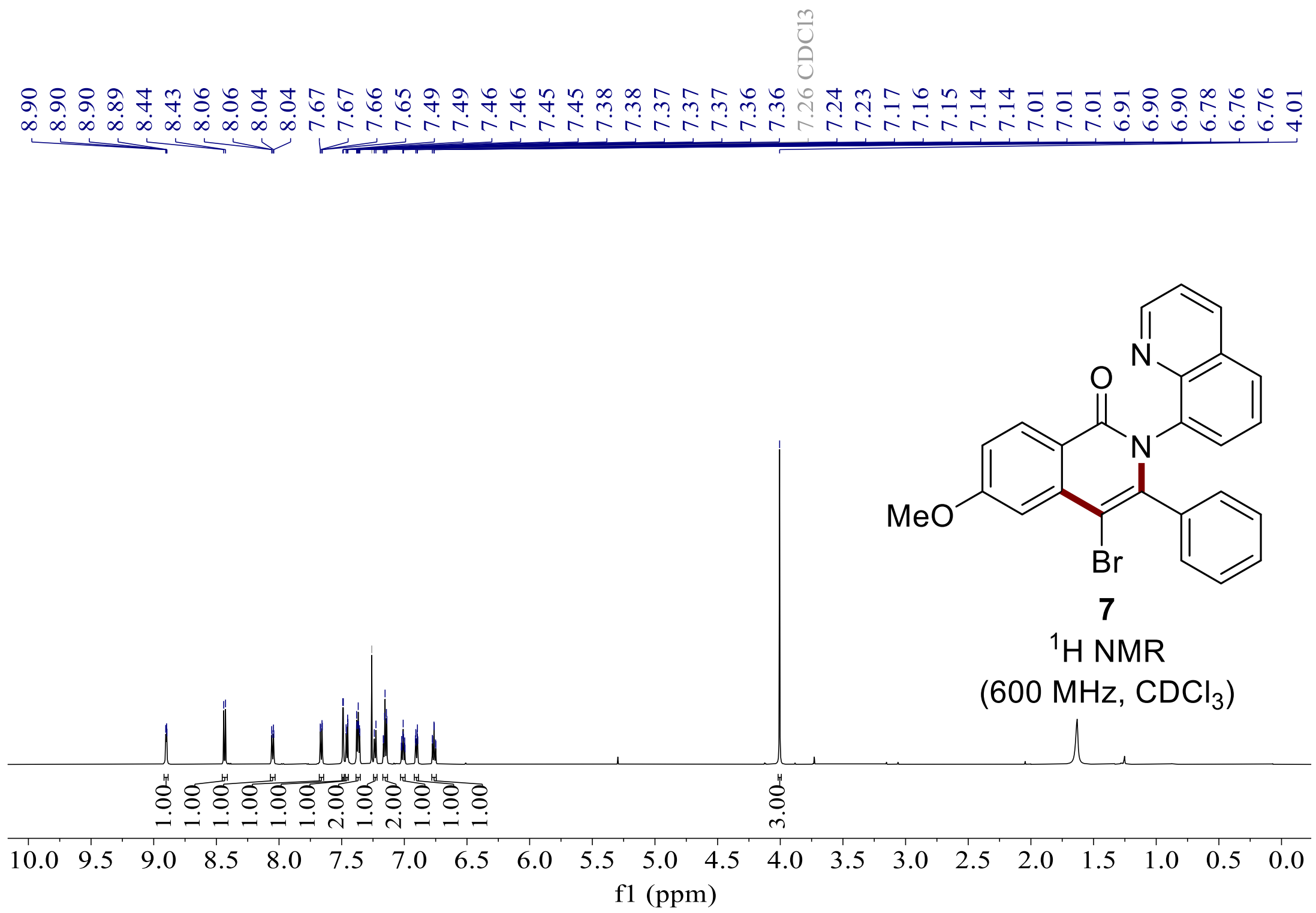
8.90
8.90
8.90
8.89
8.44
8.43
8.06
8.06
8.04
8.04
7.67
7.67
7.66
7.65
7.49
7.49
7.46
7.46
7.45
7.45
7.38
7.38
7.37
7.37
7.37
7.36
7.36
7.26 CDCl3
7.24
7.23
7.17
7.16
7.15
7.14
7.14
7.01
7.01
7.01
6.91
6.90
6.90
6.78
6.76
6.76
4.01
O
N
N
MeO
Br
7
^{1}H NMR
(600 MHz, $CDCl_3$)
1.00
1.00
1.00
1.00
1.00
2.00
1.00
2.00
1.00
1.00
1.00
3.00
10.0 9.5 9.0 8.5 8.0 7.5 7.0 6.5 6.0 5.5 5.0 4.5 4.0 3.5 3.0 2.5 2.0 1.5 1.0 0.5 0.0
f1 (ppm)

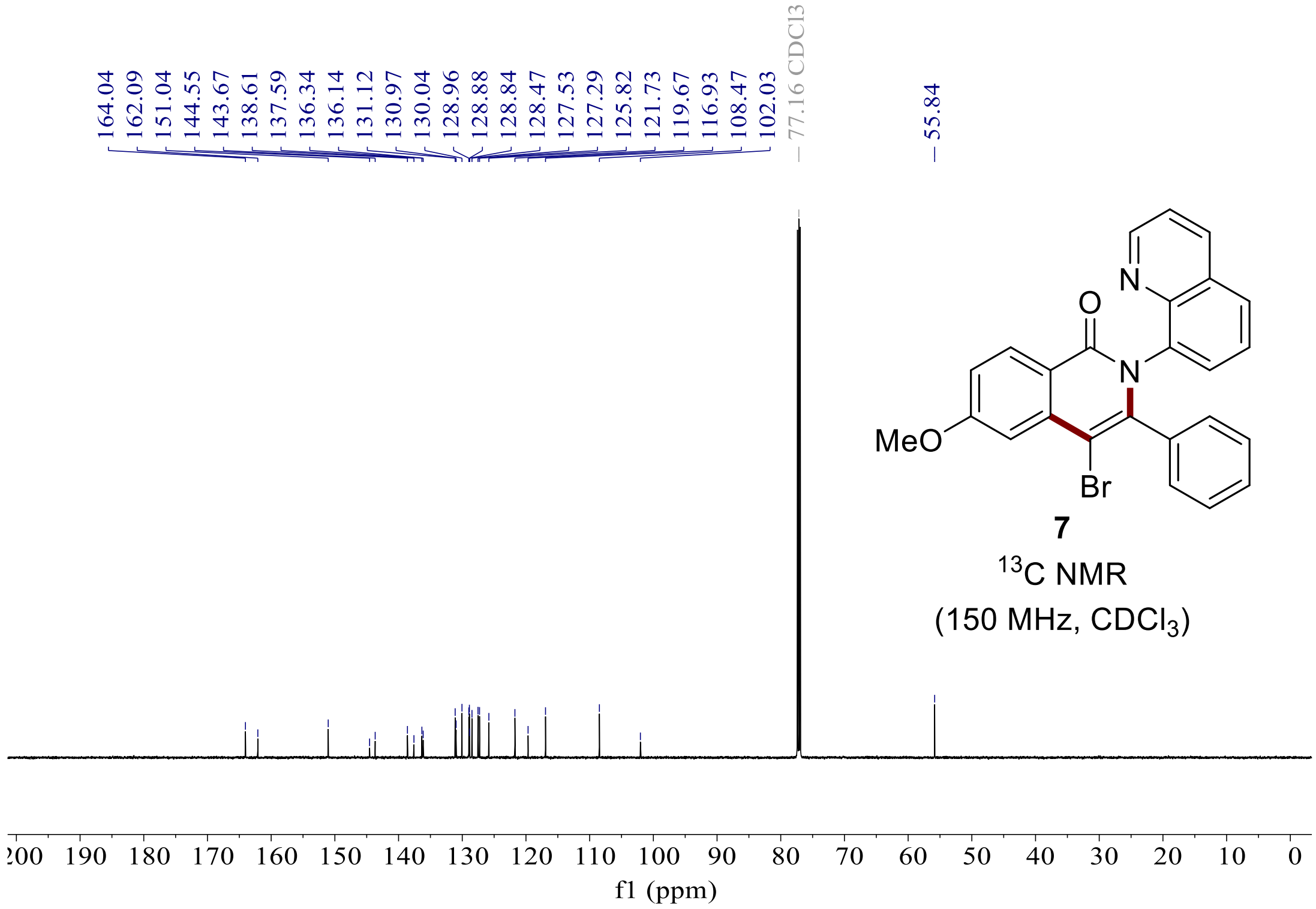
164.04
162.09
151.04
144.55
143.67
138.61
137.59
136.34
136.14
131.12
130.97
130.04
128.96
128.88
128.84
128.47
127.53
127.29
125.82
121.73
119.67
116.93
108.47
102.03
77.16 CDCl3
55.84
O
N
N
MeO
Br
7
^{13}C NMR
(150 MHz, $CDCl_3$)
200 190 180 170 160 150 140 130 120 110 100 90 80 70 60 50 40 30 20 10 0
f1 (ppm)

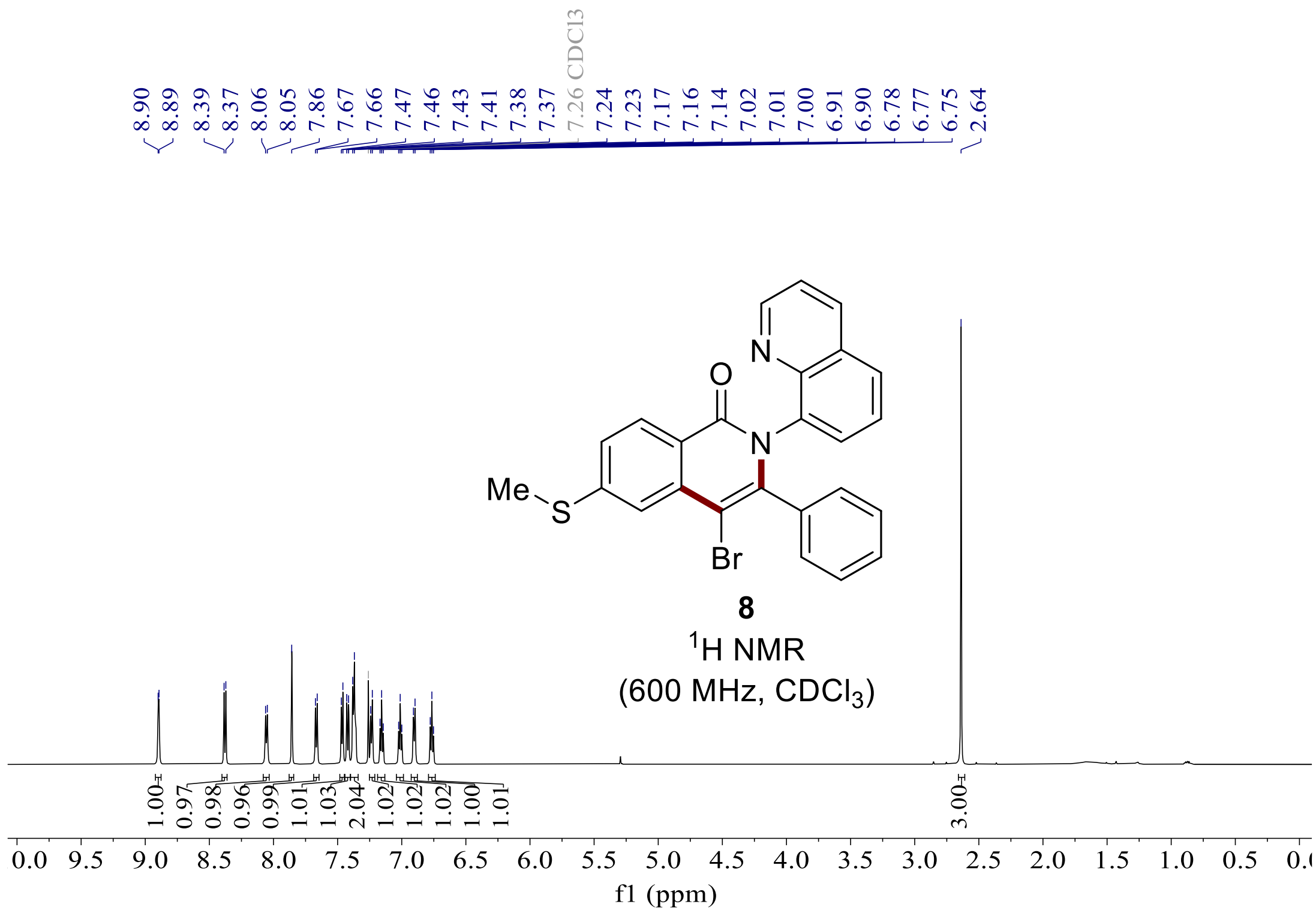
8.90
8.89
8.39
8.37
8.06
8.05
7.86
7.67
7.66
7.47
7.46
7.43
7.41
7.38
7.37
7.26 CDCl3
7.24
7.23
7.17
7.16
7.14
7.02
7.01
7.00
6.91
6.90
6.78
6.77
6.75
2.64
O
N
N
Me
S
Br
8
^{1}H NMR
(600 MHz, $CDCl_3$)
1.00
0.97
0.98
0.96
0.99
1.01
1.03
2.04
1.02
1.02
1.02
1.00
1.01
3.00
10.0 9.5 9.0 8.5 8.0 7.5 7.0 6.5 6.0 5.5 5.0 4.5 4.0 3.5 3.0 2.5 2.0 1.5 1.0 0.5 0.0
f1 (ppm)

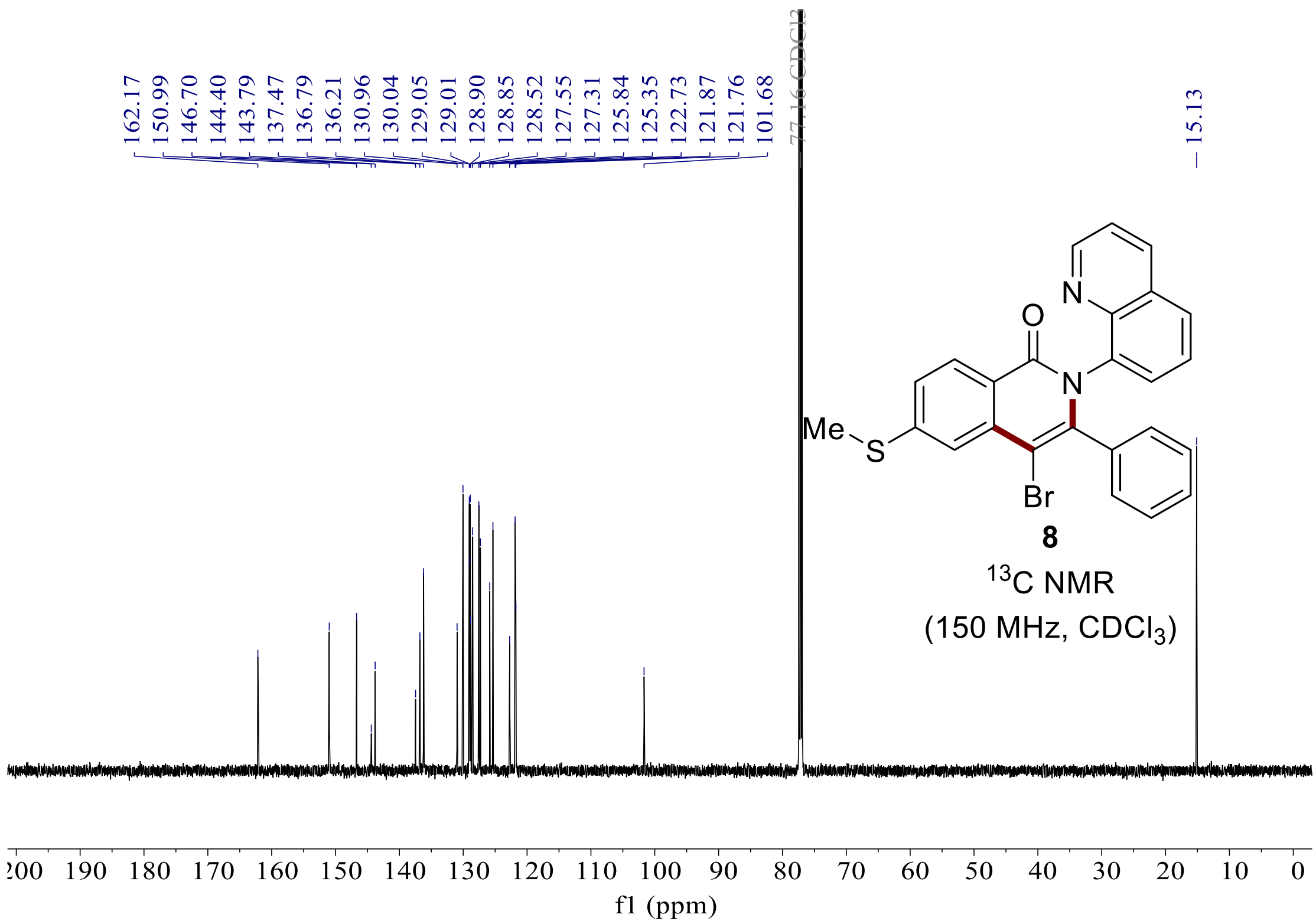

162.17
150.99
146.70
144.40
143.79
137.47
136.79
136.21
130.96
130.04
129.05
129.01
128.90
128.85
128.52
127.55
127.31
125.84
125.35
122.73
121.87
121.76
101.68
77.16 CDCl3
15.13
O
N
N
Me
S
Br
8
^{13}C NMR
(150 MHz, $CDCl_3$)
200 190 180 170 160 150 140 130 120 110 100 90 80 70 60 50 40 30 20 10 0
f1 (ppm)

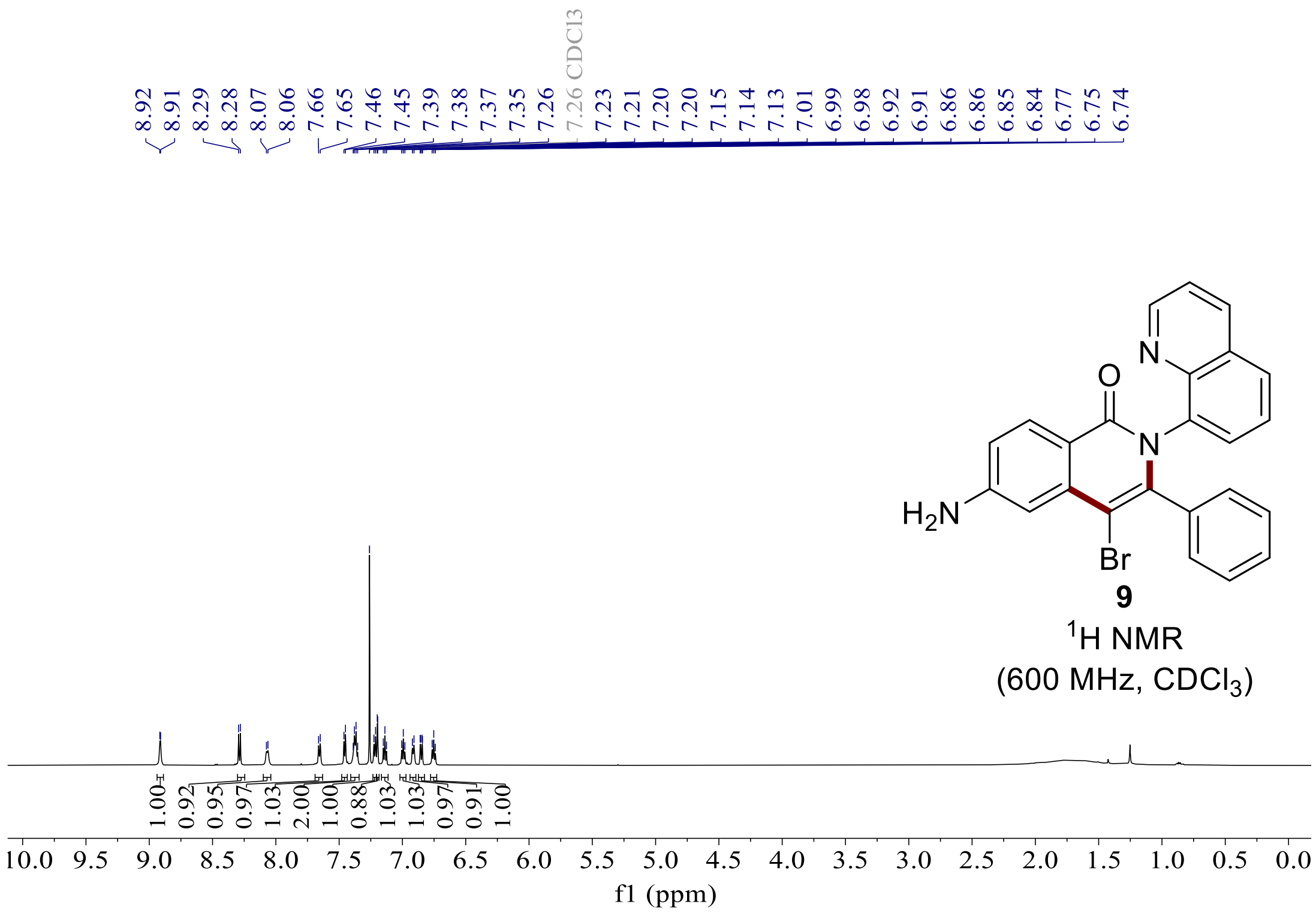

8.92
8.91
8.29
8.28
8.07
8.06
7.66
7.65
7.46
7.45
7.39
7.38
7.37
7.35
7.26
7.26 CDCl3
7.23
7.21
7.20
7.20
7.15
7.14
7.13
7.01
6.99
6.98
6.92
6.91
6.86
6.86
6.85
6.84
6.77
6.75
6.74
O
N
N
H_2N
Br
9
^{1}H NMR
(600 MHz, $CDCl_3$)
1.00
0.92
0.95
0.97
1.03
2.00
1.00
0.88
1.03
1.03
0.97
0.91
1.00
10.0 9.5 9.0 8.5 8.0 7.5 7.0 6.5 6.0 5.5 5.0 4.5 4.0 3.5 3.0 2.5 2.0 1.5 1.0 0.5 0.0
f1 (ppm)

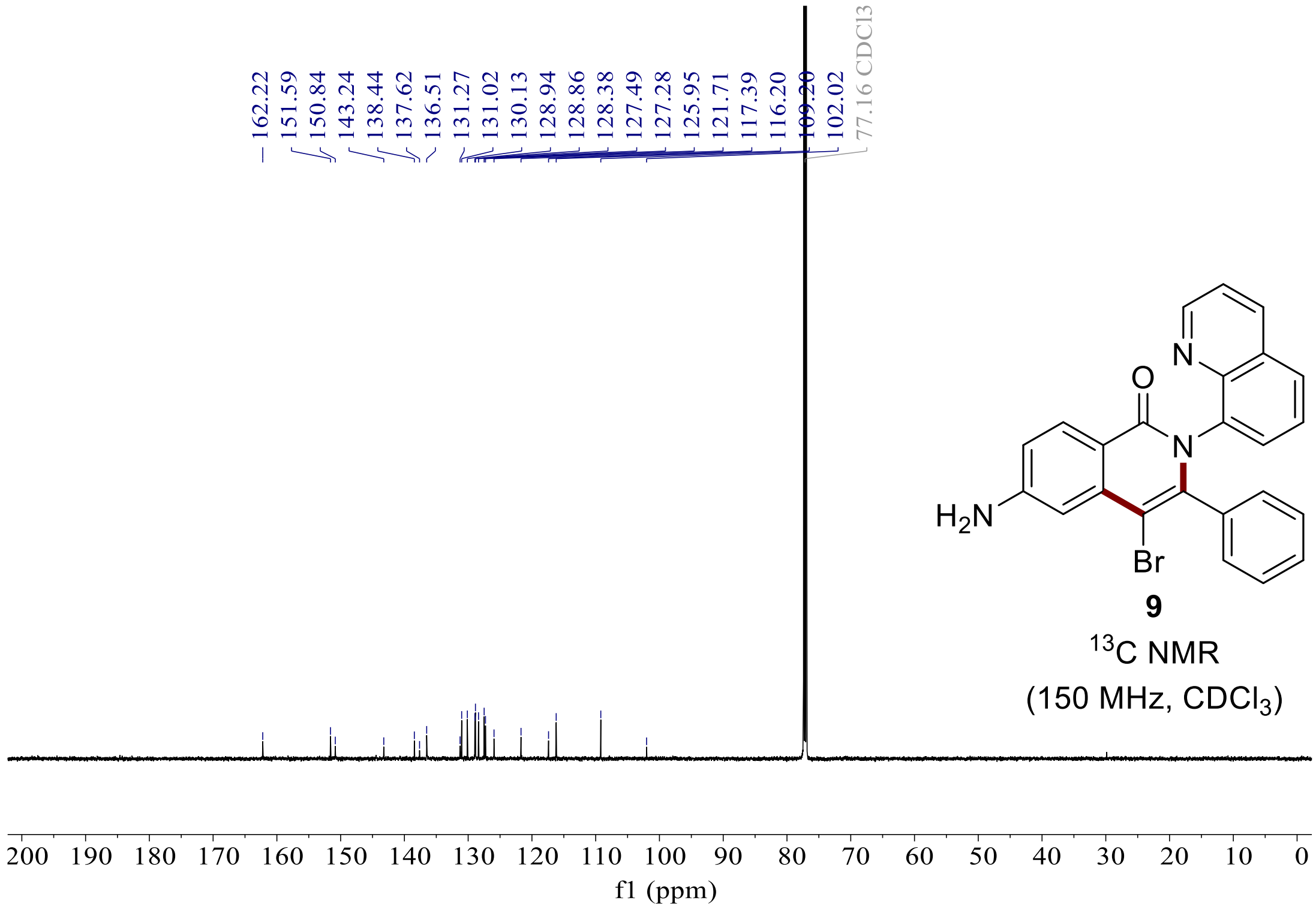
162.22
151.59
150.84
143.24
138.44
137.62
136.51
131.27
131.02
130.13
128.94
128.86
128.38
127.49
127.28
125.95
121.71
117.39
116.20
109.20
102.02
77.16 CDCl3
O
N
N
H2N
Br
9
13C NMR
(150 MHz, CDCl3)
200 190 180 170 160 150 140 130 120 110 100 90 80 70 60 50 40 30 20 10 0
f1 (ppm)

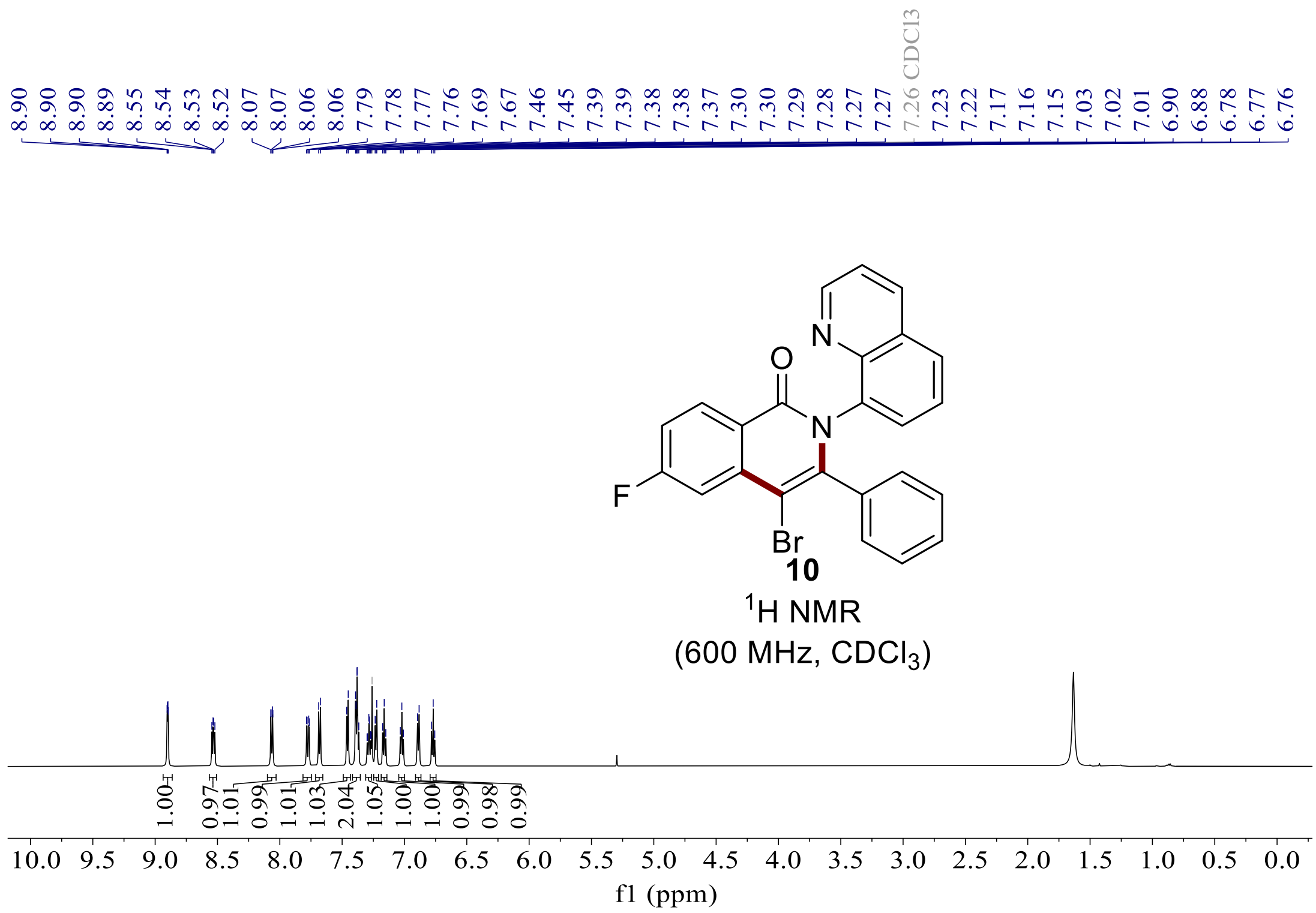
8.90
8.90
8.90
8.89
8.55
8.54
8.53
8.52
8.07
8.07
8.06
8.06
7.79
7.78
7.77
7.76
7.69
7.67
7.46
7.45
7.39
7.39
7.38
7.38
7.37
7.30
7.30
7.29
7.28
7.27
7.27
7.26 CDCl3
7.23
7.22
7.17
7.16
7.15
7.03
7.02
7.01
6.90
6.88
6.78
6.77
6.76
O
N
N
F
Br
10
1H NMR
(600 MHz, CDCl3)
1.00
0.97
1.01
0.99
1.01
1.03
2.04
1.05
1.00
1.00
0.99
0.98
0.99
10.0 9.5 9.0 8.5 8.0 7.5 7.0 6.5 6.0 5.5 5.0 4.5 4.0 3.5 3.0 2.5 2.0 1.5 1.0 0.5 0.0
f1 (ppm)

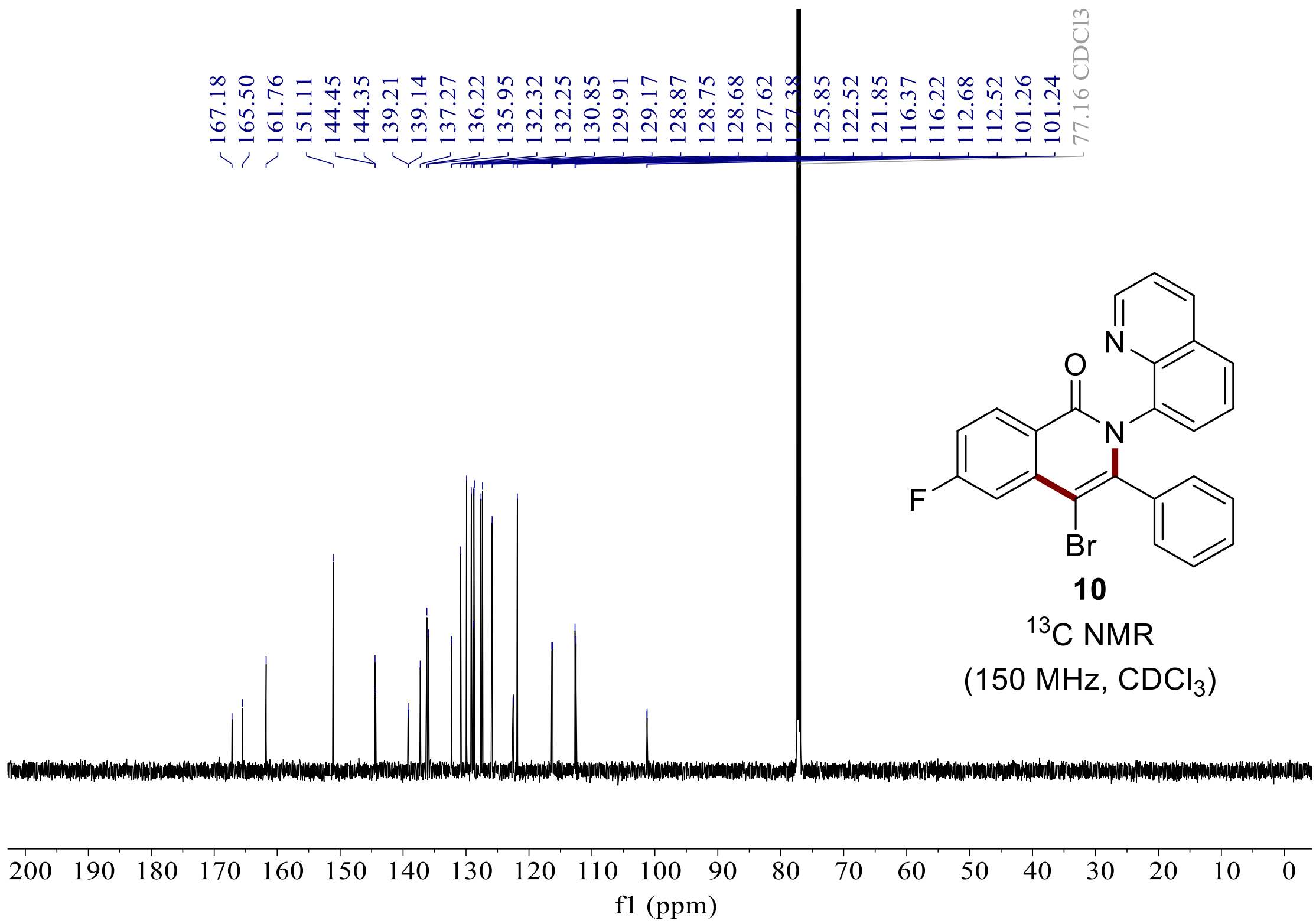
167.18
165.50
161.76
151.11
144.45
144.35
139.21
139.14
137.27
136.22
135.95
132.32
132.25
130.85
129.91
129.17
128.87
128.75
128.68
127.62
127.38
125.85
122.52
121.85
116.37
116.22
112.68
112.52
101.26
101.24
77.16 CDCl3
O
N
N
F
Br
10
13C NMR
(150 MHz, CDCl3)
200 190 180 170 160 150 140 130 120 110 100 90 80 70 60 50 40 30 20 10 0
f1 (ppm)

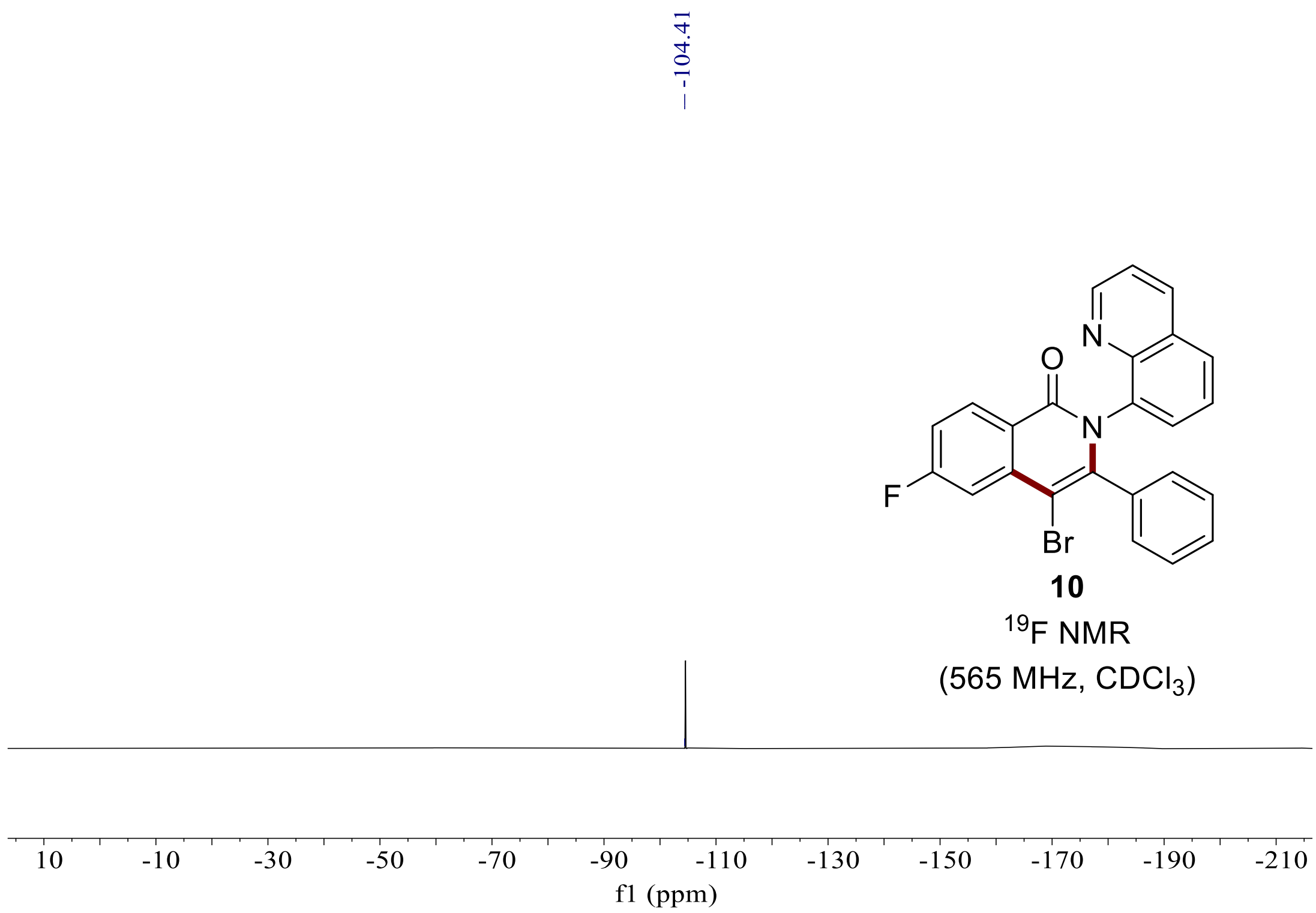
-104.41
O
N
N
F
Br
10
19F NMR
(565 MHz, CDCl3)
10 -10 -30 -50 -70 -90 -110 -130 -150 -170 -190 -210
f1 (ppm)

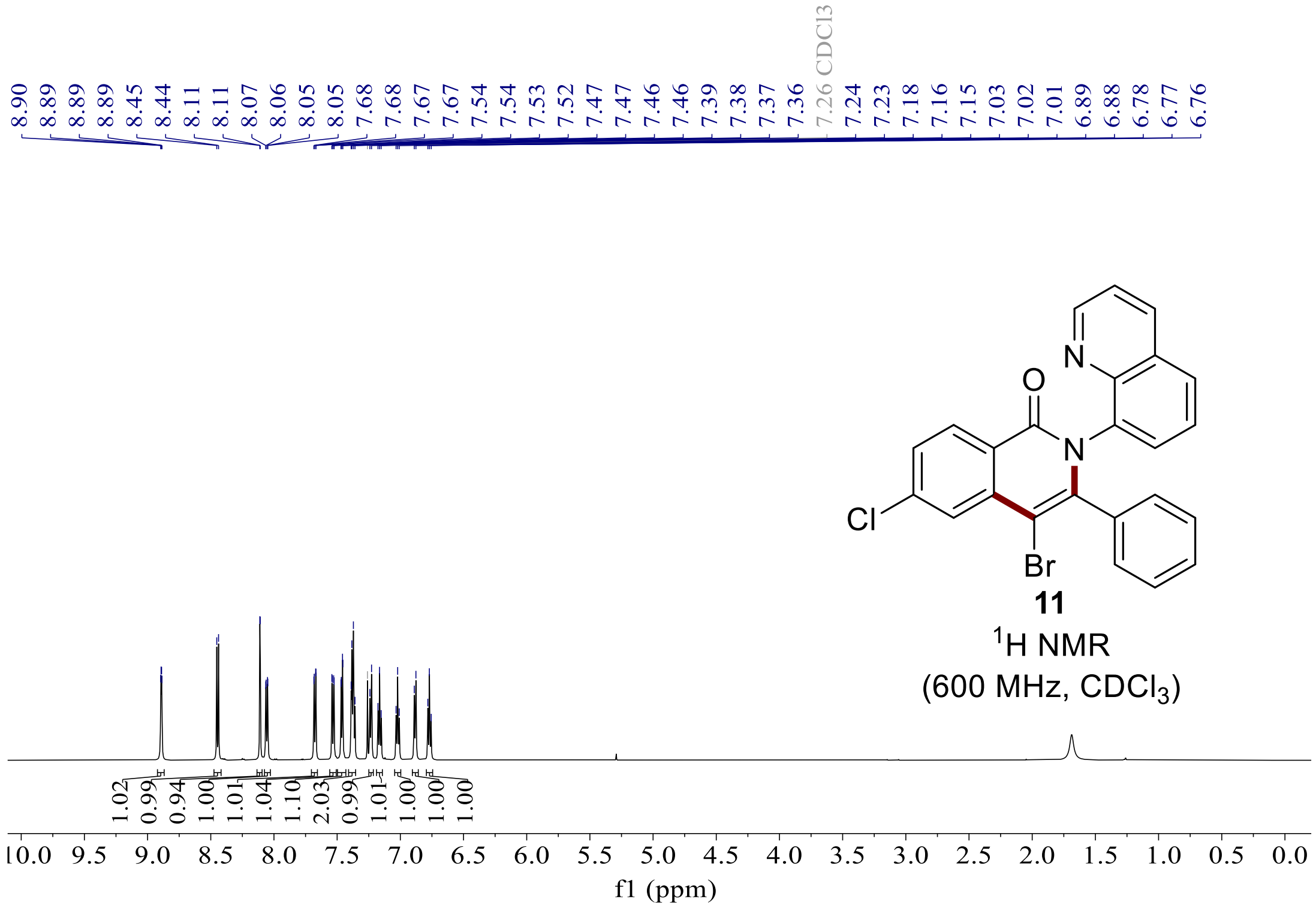
8.90 8.89 8.89 8.89 8.45 8.44 8.11 8.11 8.07 8.06 8.05 8.05 7.68 7.68 7.67 7.67 7.54 7.54 7.53 7.52 7.47 7.47 7.46 7.46 7.39 7.38 7.37 7.36 7.26 CDCl3 7.24 7.23 7.18 7.16 7.15 7.03 7.02 7.01 6.89 6.88 6.78 6.77 6.76
1.02 0.99 0.94 1.00 1.01 1.04 1.10 2.03 0.99 1.01 1.00 1.00 1.00
10.0 9.5 9.0 8.5 8.0 7.5 7.0 6.5 6.0 5.5 5.0 4.5 4.0 3.5 3.0 2.5 2.0 1.5 1.0 0.5 0.0
f1 (ppm)
O
N
N
Cl
Br
11
1H NMR
(600 MHz, CDCl3)

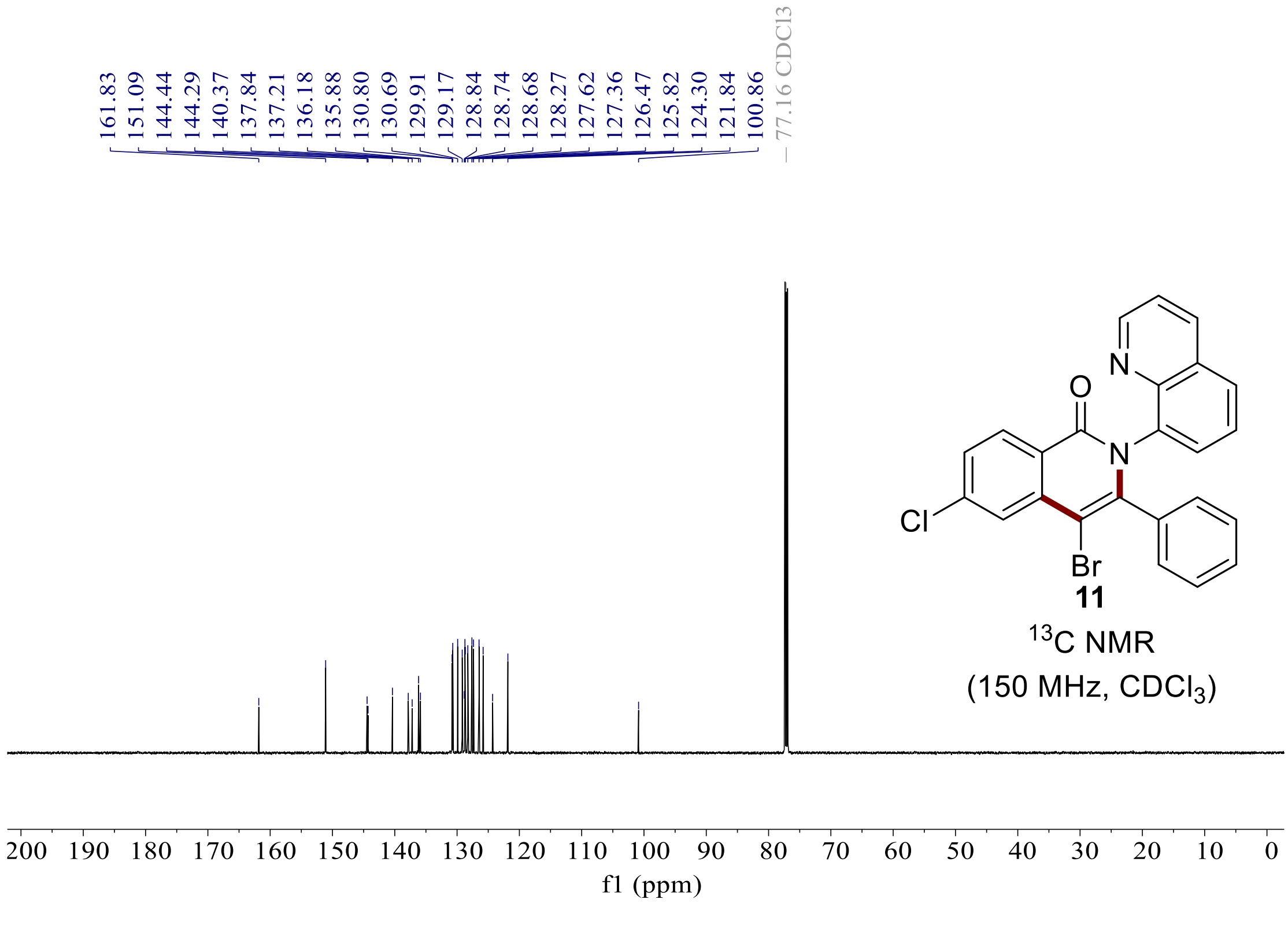
161.83 151.09 144.44 144.29 140.37 137.84 137.21 136.18 135.88 130.80 130.69 129.91 129.17 128.84 128.74 128.68 128.27 127.62 127.36 126.47 125.82 124.30 121.84 100.86
77.16 CDCl3
200 190 180 170 160 150 140 130 120 110 100 90 80 70 60 50 40 30 20 10 0
f1 (ppm)
O
N
N
Cl
Br
11
13C NMR
(150 MHz, CDCl3)

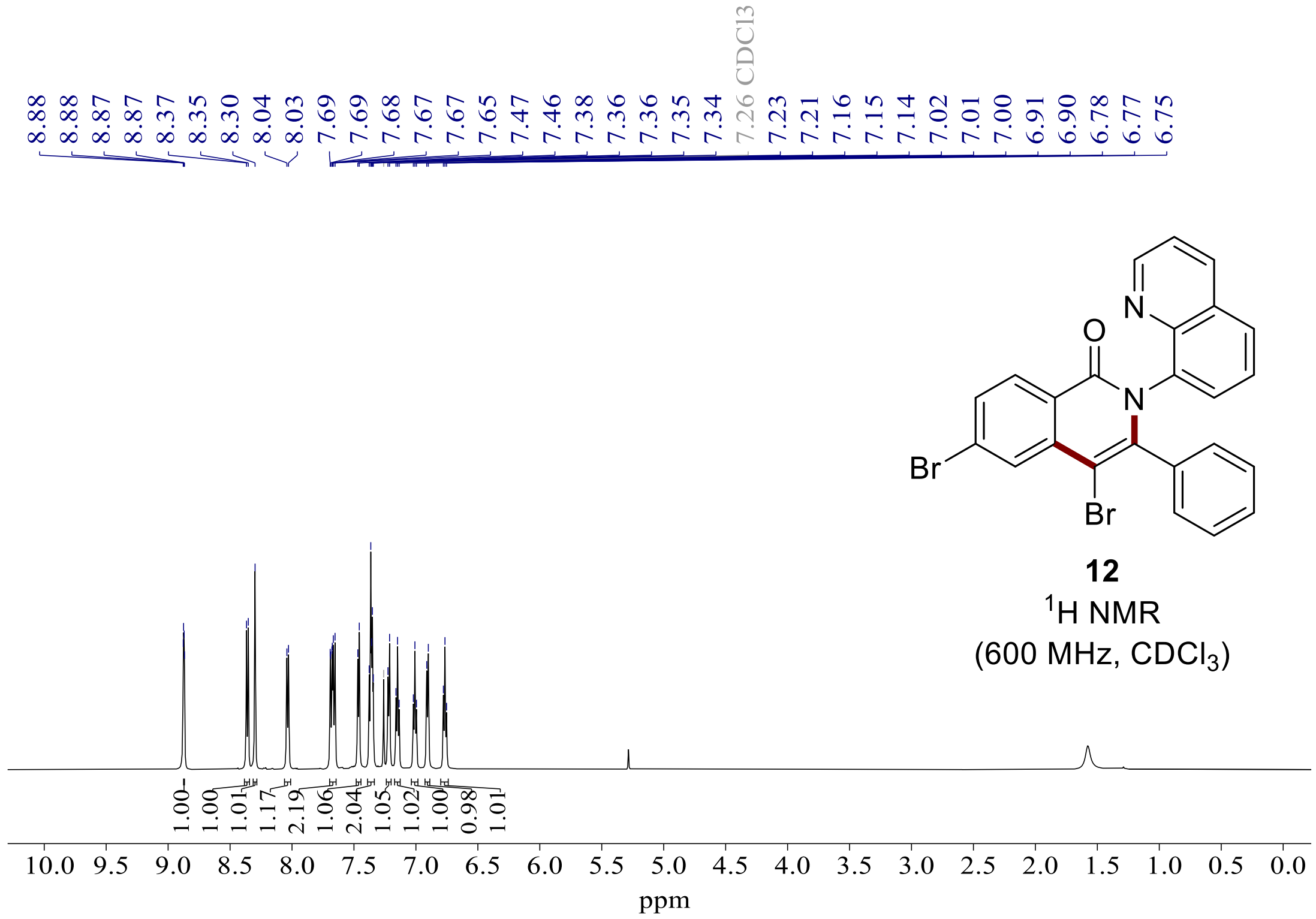

12

^{1}H NMR

(600 MHz, $CDCl_3$)

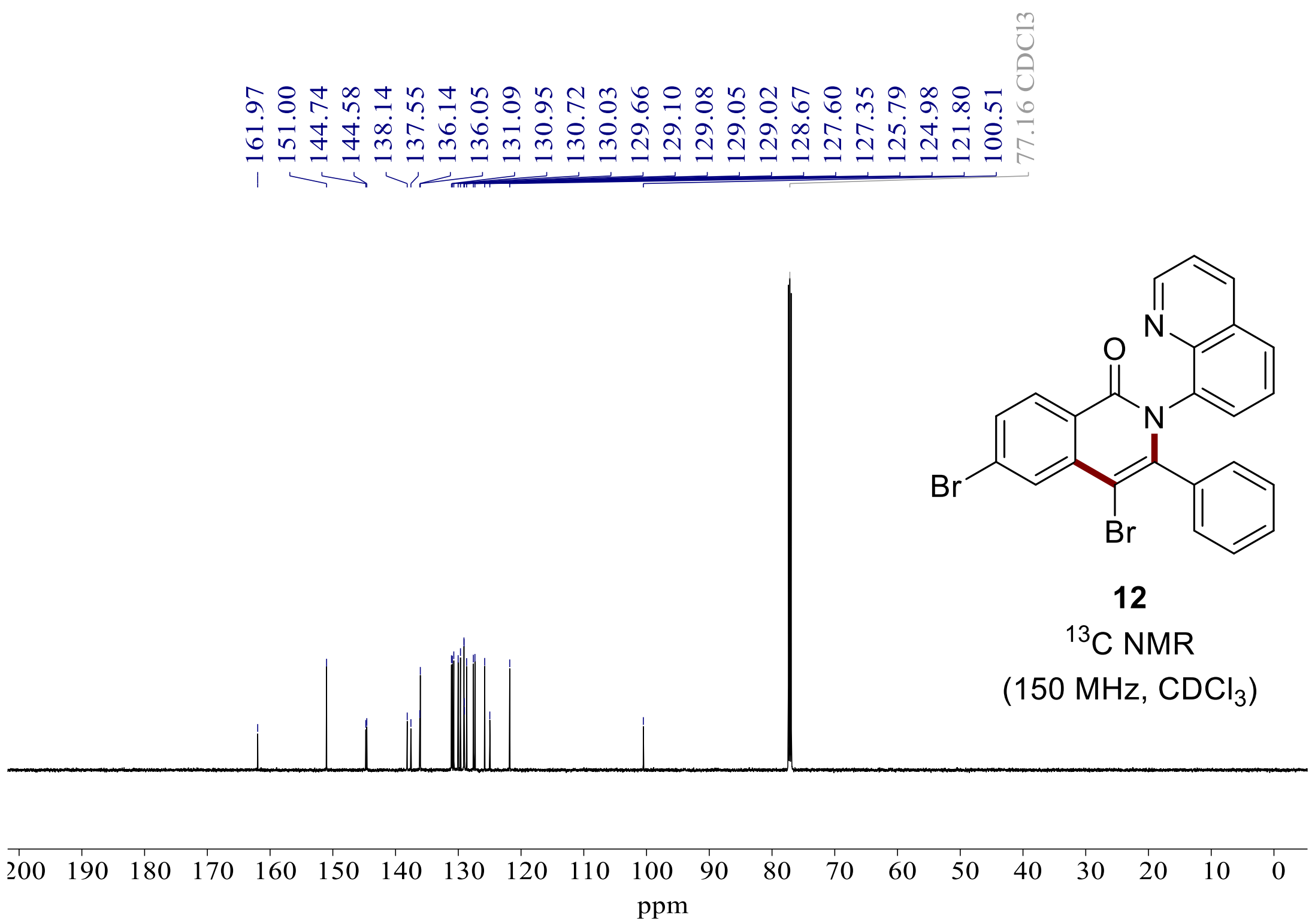

12

^{13}C NMR

(150 MHz, $CDCl_3$)

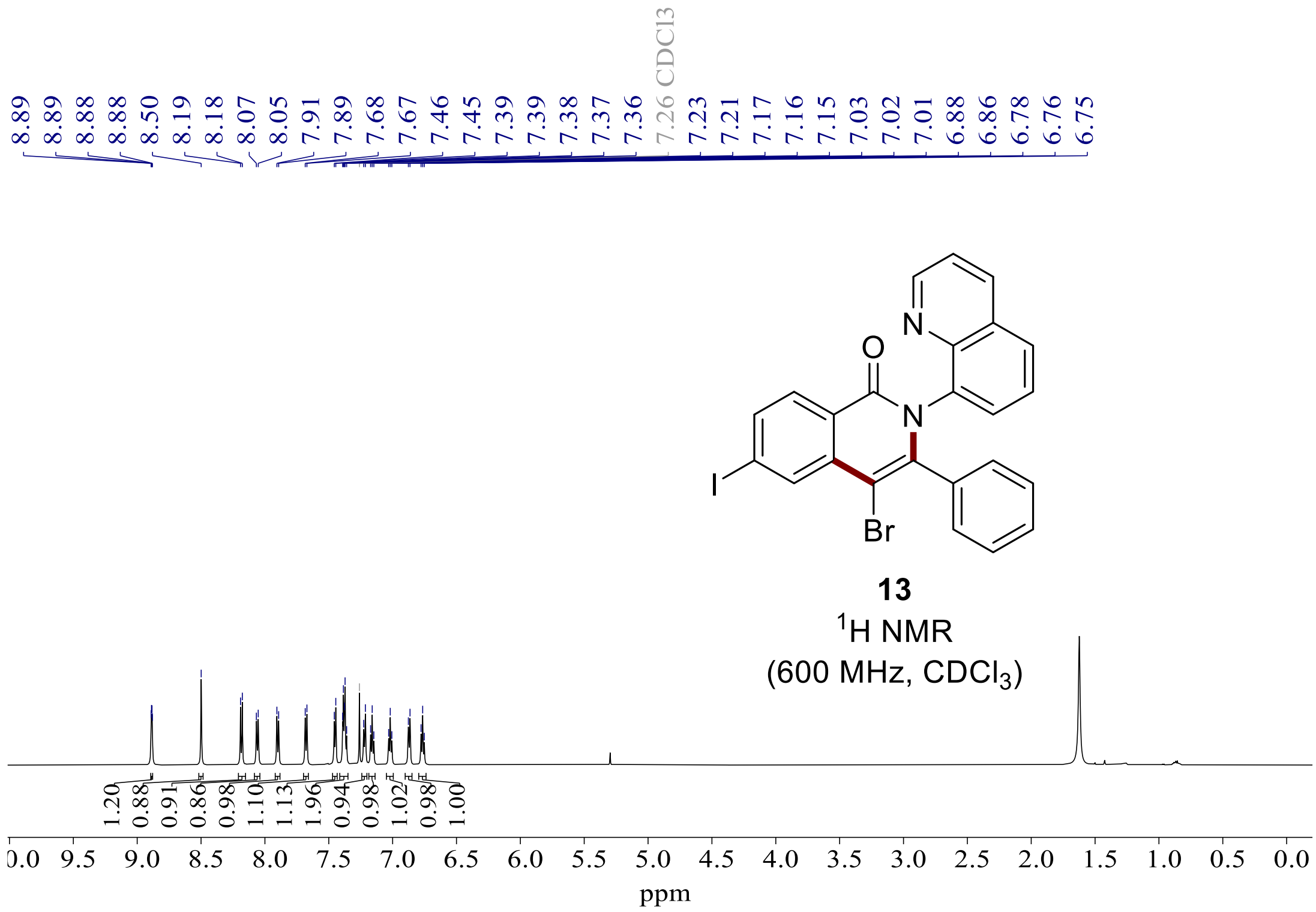
8.89 8.89 8.88 8.88 8.50 8.19 8.18 8.07 8.05 7.91 7.89 7.68 7.67 7.46 7.45 7.39 7.39 7.38 7.37 7.36 7.26 CDCl3 7.23 7.21 7.17 7.16 7.15 7.03 7.02 7.01 6.88 6.86 6.78 6.76 6.75
O
N
I
Br
13
1H NMR
(600 MHz, CDCl3)
1.20 0.88 0.91 0.86 0.98 1.10 1.13 1.96 0.94 0.98 1.02 0.98 1.00
9.5 9.0 8.5 8.0 7.5 7.0 6.5 6.0 5.5 5.0 4.5 4.0 3.5 3.0 2.5 2.0 1.5 1.0 0.5 0.0
ppm

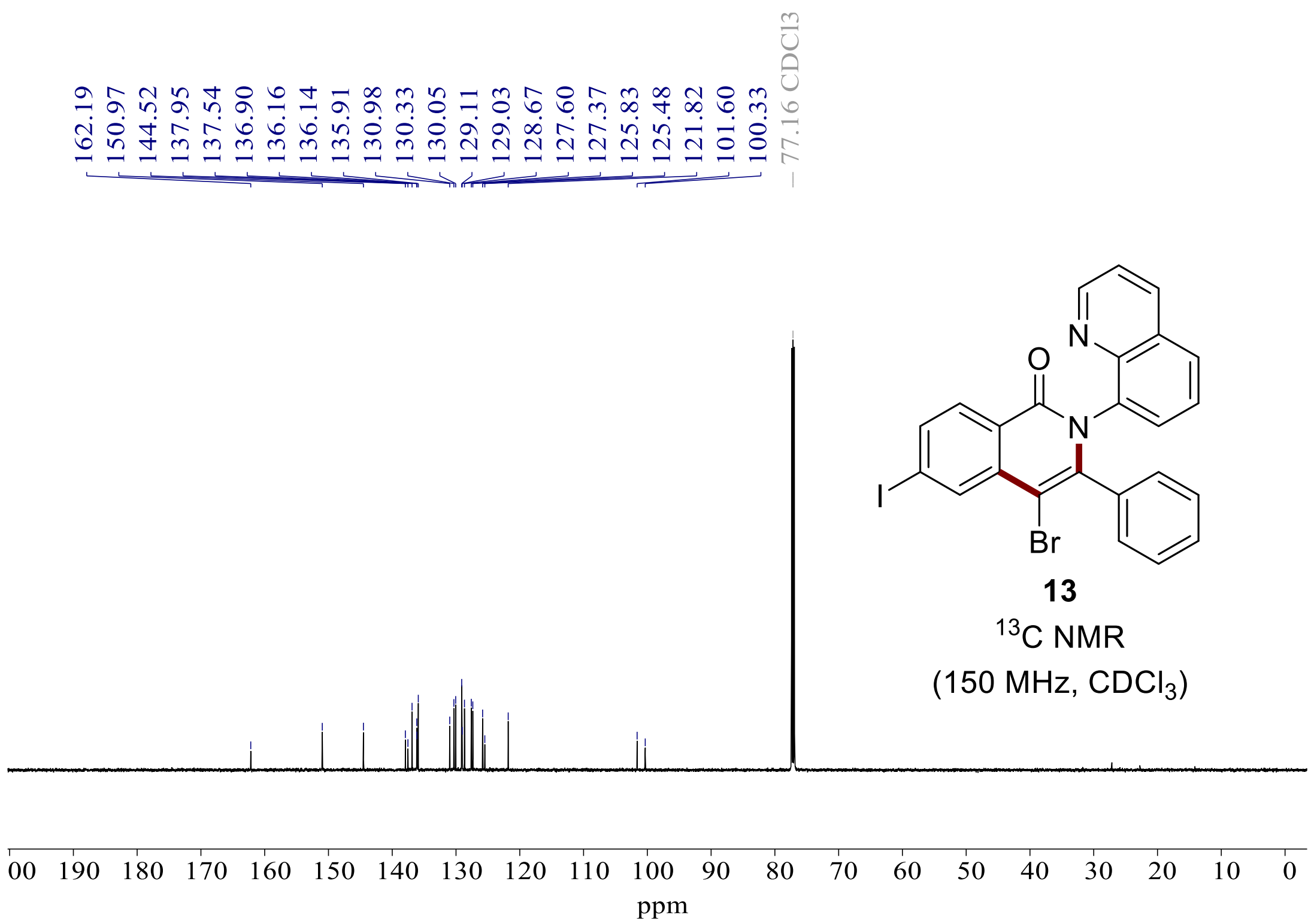
162.19 150.97 144.52 137.95 137.54 136.90 136.16 136.14 135.91 130.98 130.33 130.05 129.11 129.03 128.67 127.60 127.37 125.83 125.48 121.82 101.60 100.33
77.16 CDCl3
O
N
I
Br
13
13C NMR
(150 MHz, CDCl3)
190 180 170 160 150 140 130 120 110 100 90 80 70 60 50 40 30 20 10 0
ppm

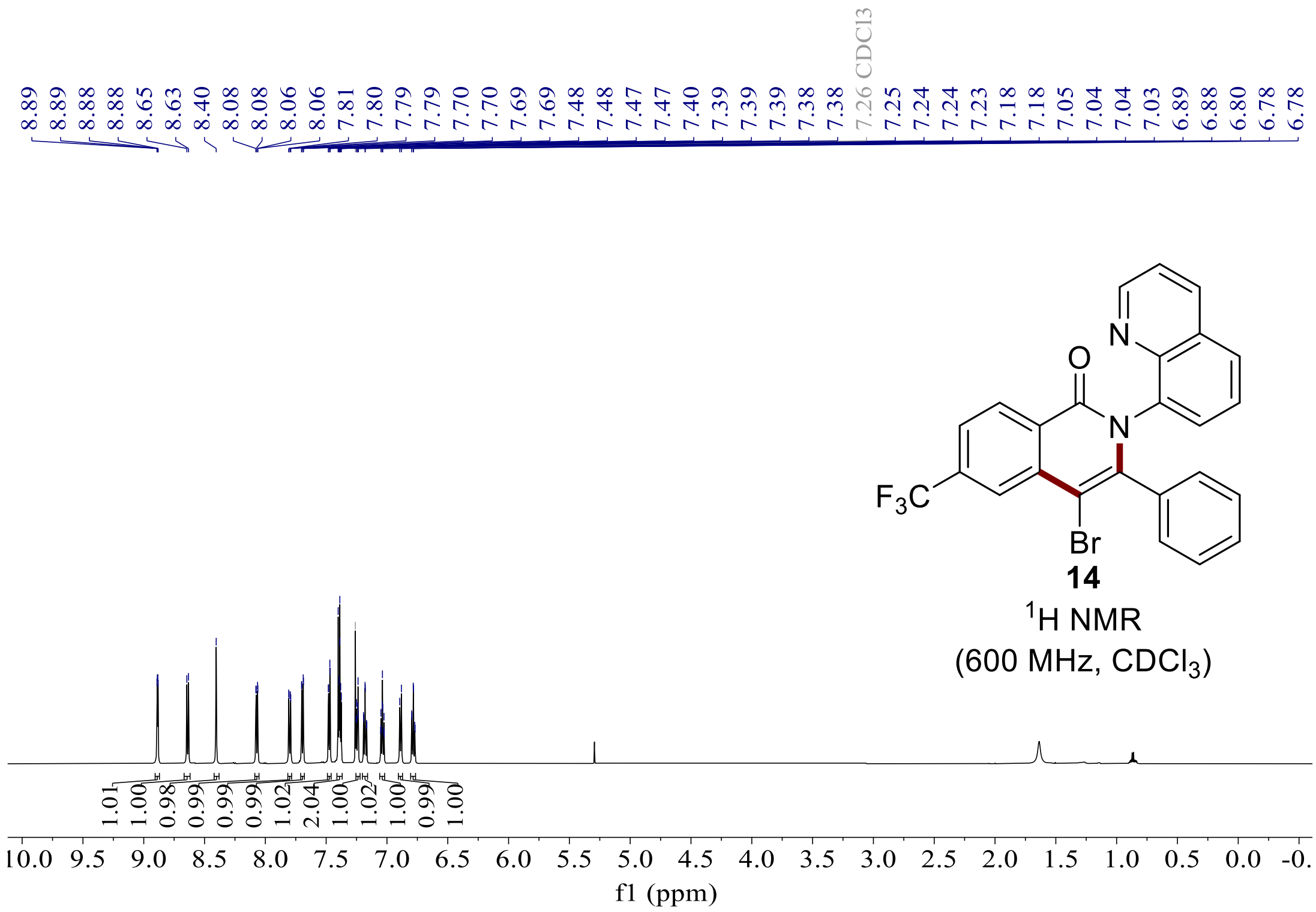

14
^{1}H NMR
(600 MHz, CDCl$_3$)
f1 (ppm)

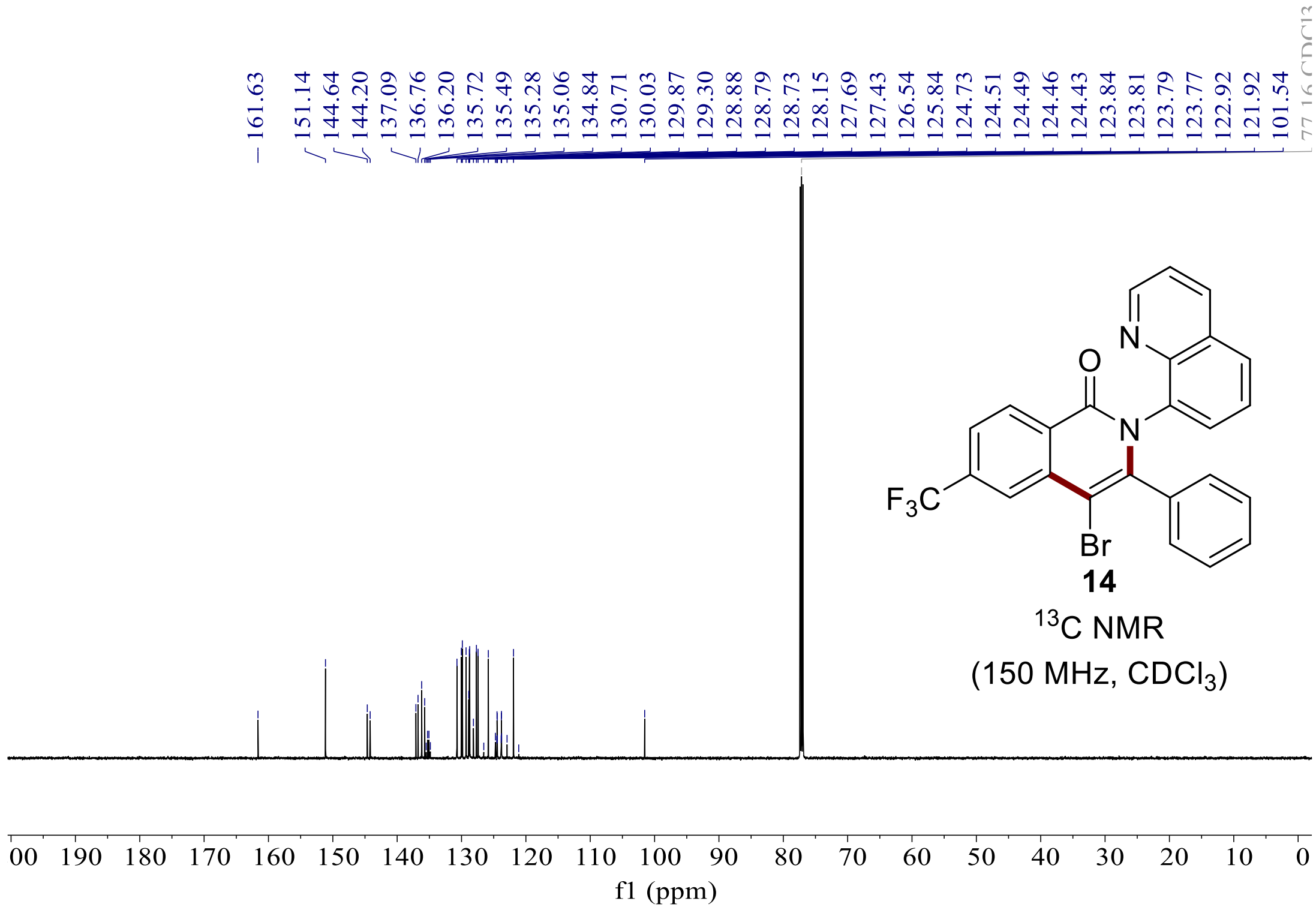

14
^{13}C NMR
(150 MHz, CDCl$_3$)
f1 (ppm)

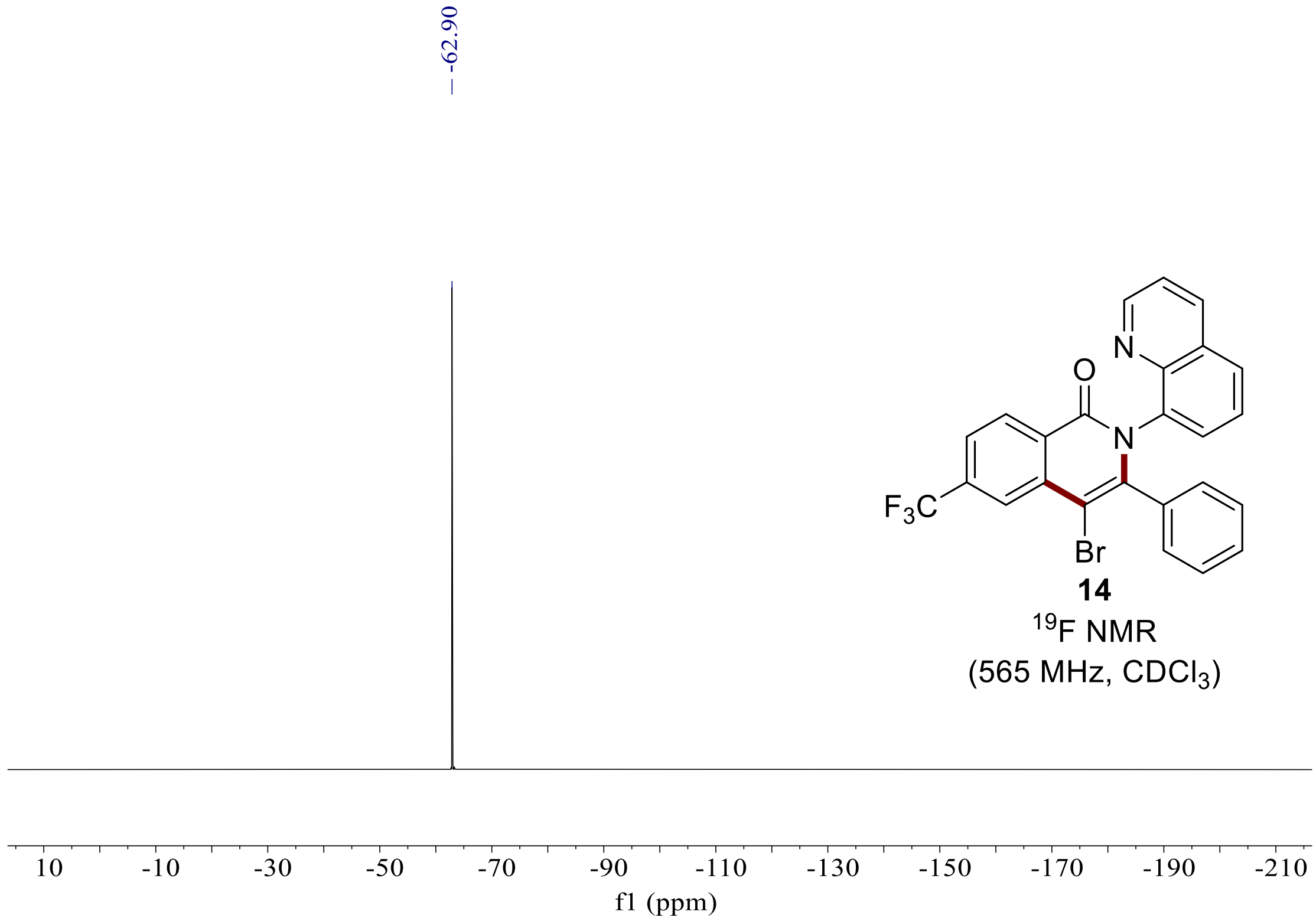
-62.90
F3C
O
N
N
Br
14
19F NMR
(565 MHz, CDCl3)
10
-10
-30
-50
-70
-90
-110
-130
-150
-170
-190
-210
f1 (ppm)

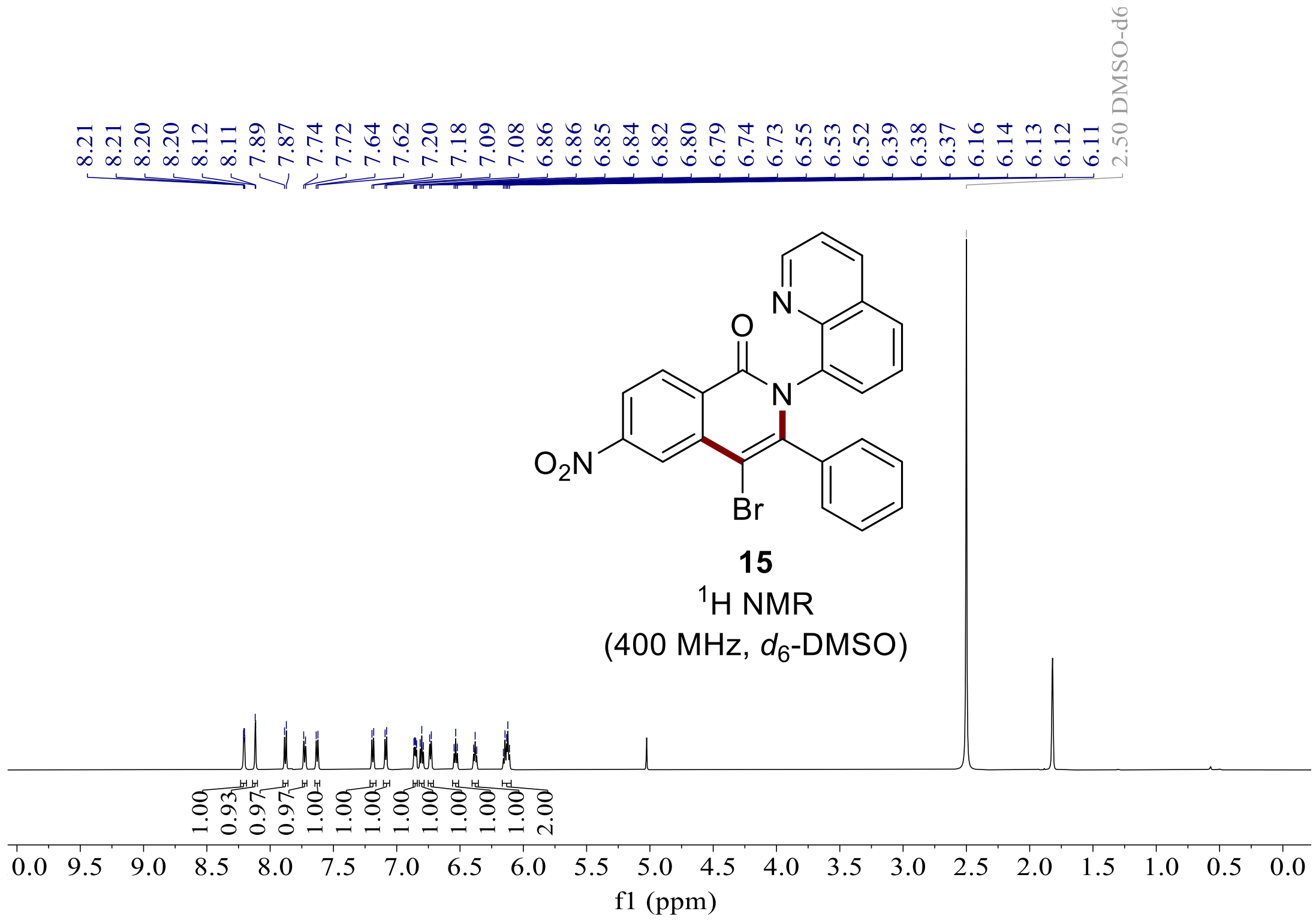
8.21
8.21
8.20
8.20
8.12
8.11
7.89
7.87
7.74
7.72
7.64
7.62
7.20
7.18
7.09
7.08
6.86
6.86
6.85
6.84
6.82
6.80
6.79
6.74
6.73
6.55
6.53
6.52
6.39
6.38
6.37
6.16
6.14
6.13
6.12
6.11
2.50 DMSO-d6
O2N
O
N
N
Br
15
1H NMR
(400 MHz, d6-DMSO)
1.00
0.93
0.97
0.97
1.00
1.00
1.00
1.00
1.00
1.00
1.00
1.00
2.00
0.0
9.5
9.0
8.5
8.0
7.5
7.0
6.5
6.0
5.5
5.0
4.5
4.0
3.5
3.0
2.5
2.0
1.5
1.0
0.5
0.0
f1 (ppm)

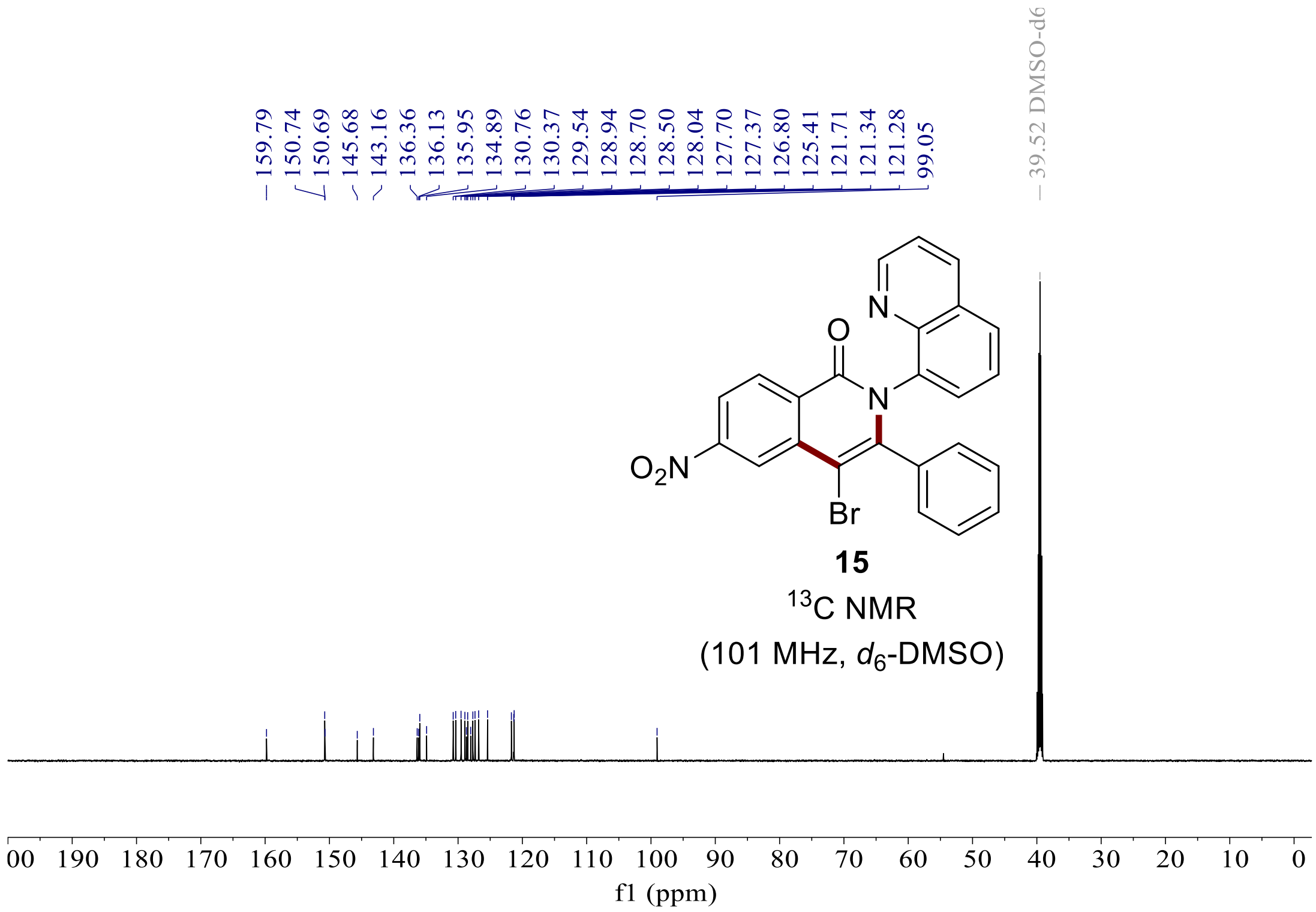
159.79
150.74
150.69
145.68
143.16
136.36
136.13
135.95
134.89
130.76
130.37
129.54
128.94
128.70
128.50
128.04
127.70
127.37
126.80
125.41
121.71
121.34
121.28
99.05
39.52 DMSO-d6
O
N
N
O_2N
Br
15
^{13}C NMR
(101 MHz, d_6-DMSO)
f1 (ppm)

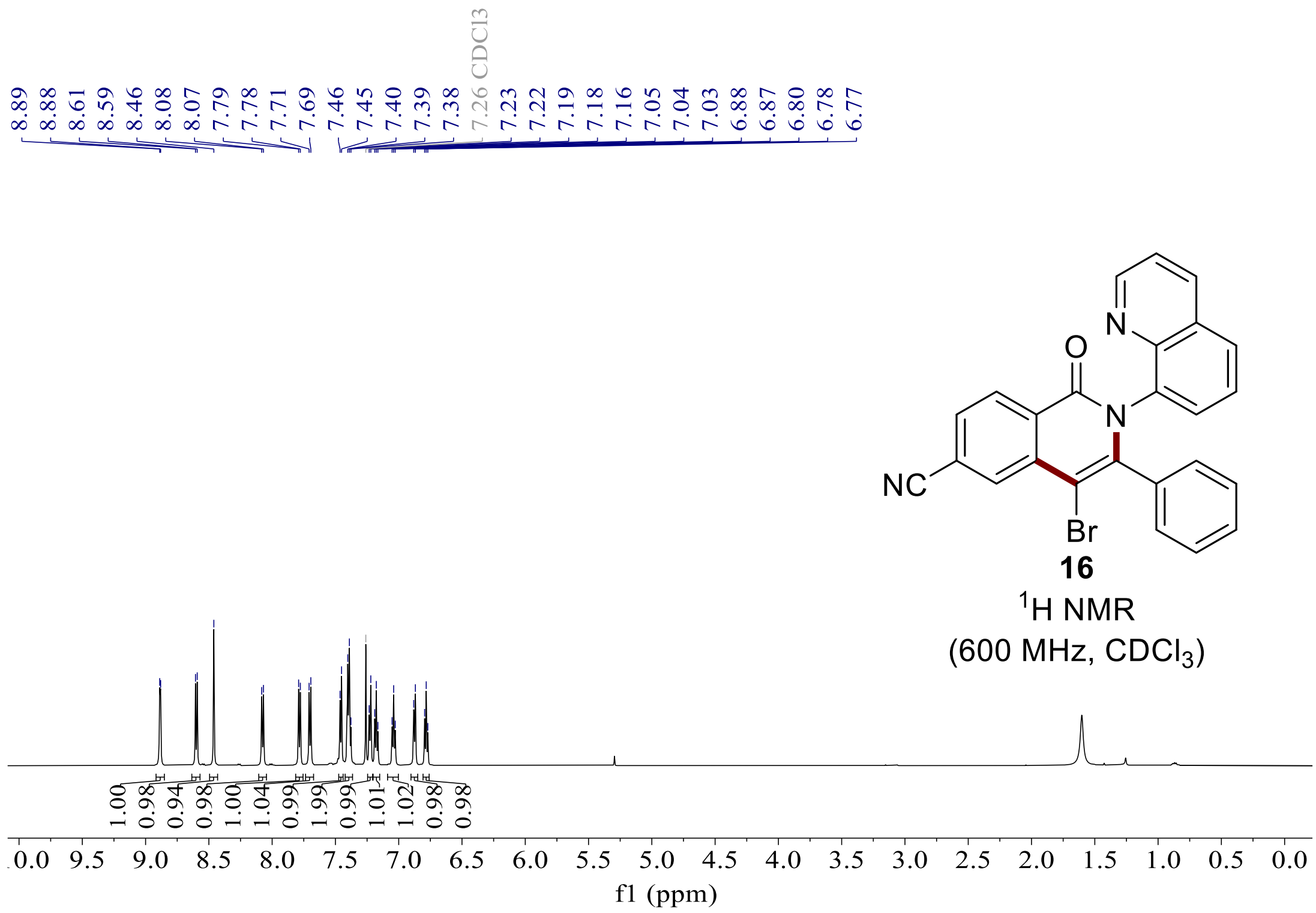
8.89
8.88
8.61
8.59
8.46
8.08
8.07
7.79
7.78
7.71
7.69
7.46
7.45
7.40
7.39
7.38
7.26 CDCl3
7.23
7.22
7.19
7.18
7.16
7.05
7.04
7.03
6.88
6.87
6.80
6.78
6.77
O
N
N
NC
Br
16
^1H NMR
(600 MHz, $CDCl_3$)
1.00
0.98
0.94
0.98
1.00
1.04
0.99
1.99
0.99
1.01
1.02
0.98
0.98
f1 (ppm)

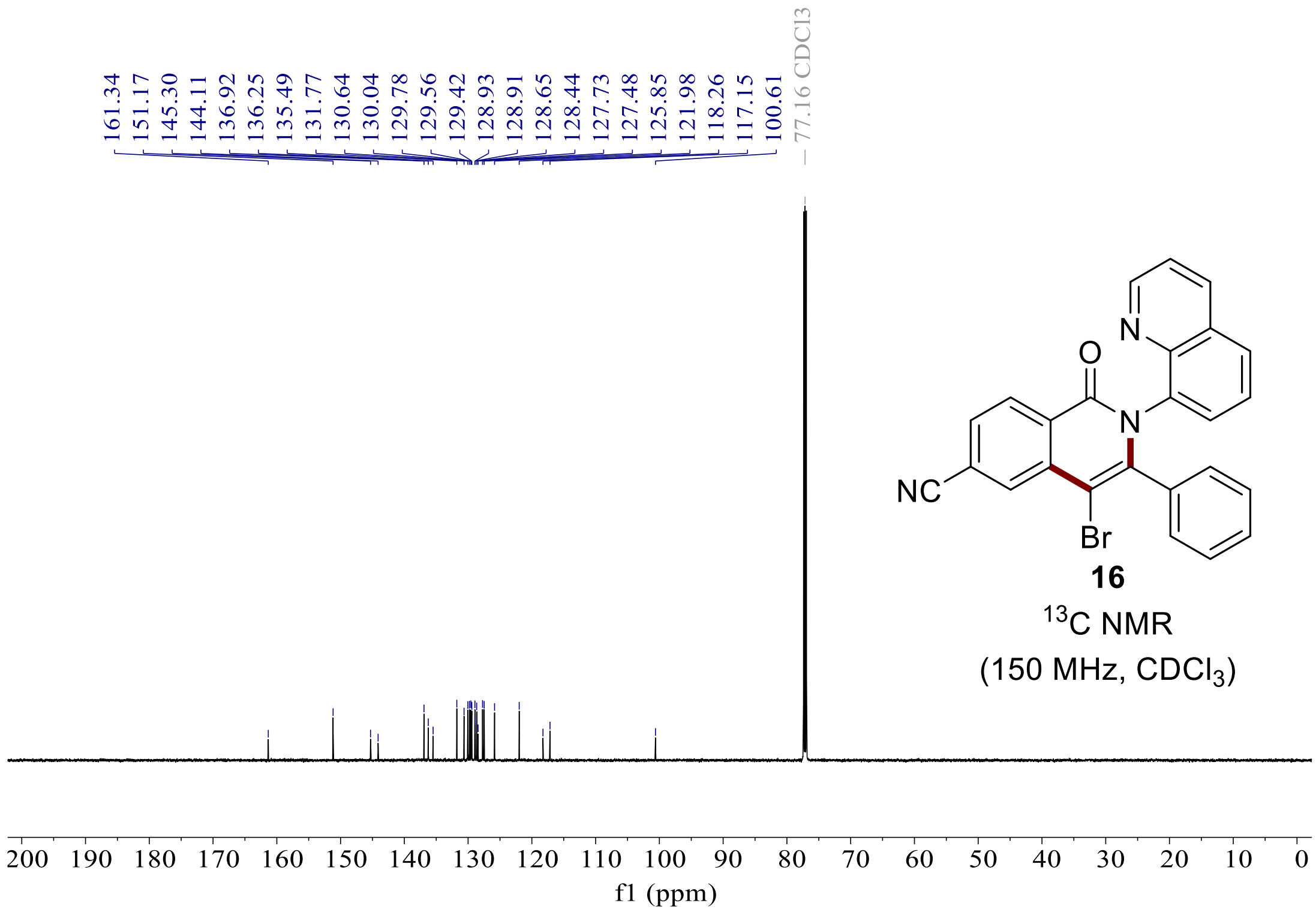
161.34
151.17
145.30
144.11
136.92
136.25
135.49
131.77
130.64
130.04
129.78
129.56
129.42
128.93
128.91
128.65
128.44
127.73
127.48
125.85
121.98
118.26
117.15
100.61
77.16 CDCl3
NC
O
N
N
Br
16
^{13}C NMR
(150 MHz, $CDCl_3$)
200 190 180 170 160 150 140 130 120 110 100 90 80 70 60 50 40 30 20 10 0
f1 (ppm)

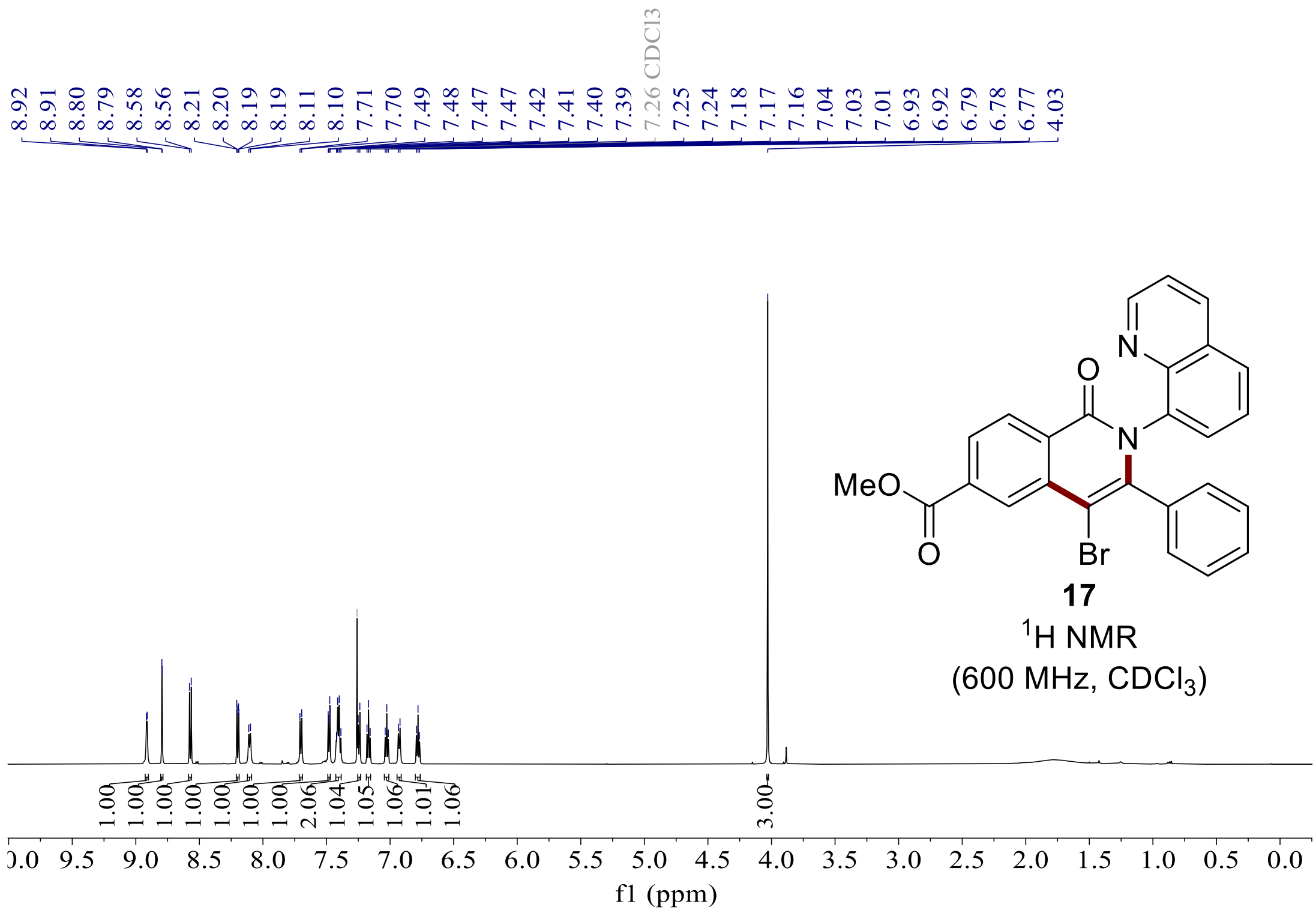
8.92
8.91
8.80
8.79
8.58
8.56
8.21
8.20
8.19
8.19
8.11
8.10
7.71
7.70
7.49
7.48
7.47
7.47
7.42
7.41
7.40
7.39
7.26 CDCl3
7.25
7.24
7.18
7.17
7.16
7.04
7.03
7.01
6.93
6.92
6.79
6.78
6.77
4.03
MeO
O
O
N
N
Br
17
^{1}H NMR
(600 MHz, $CDCl_3$)
1.00
1.00
1.00
1.00
1.00
1.00
2.06
1.04
1.05
1.06
1.01
1.06
3.00
9.5 9.0 8.5 8.0 7.5 7.0 6.5 6.0 5.5 5.0 4.5 4.0 3.5 3.0 2.5 2.0 1.5 1.0 0.5 0.0
f1 (ppm)

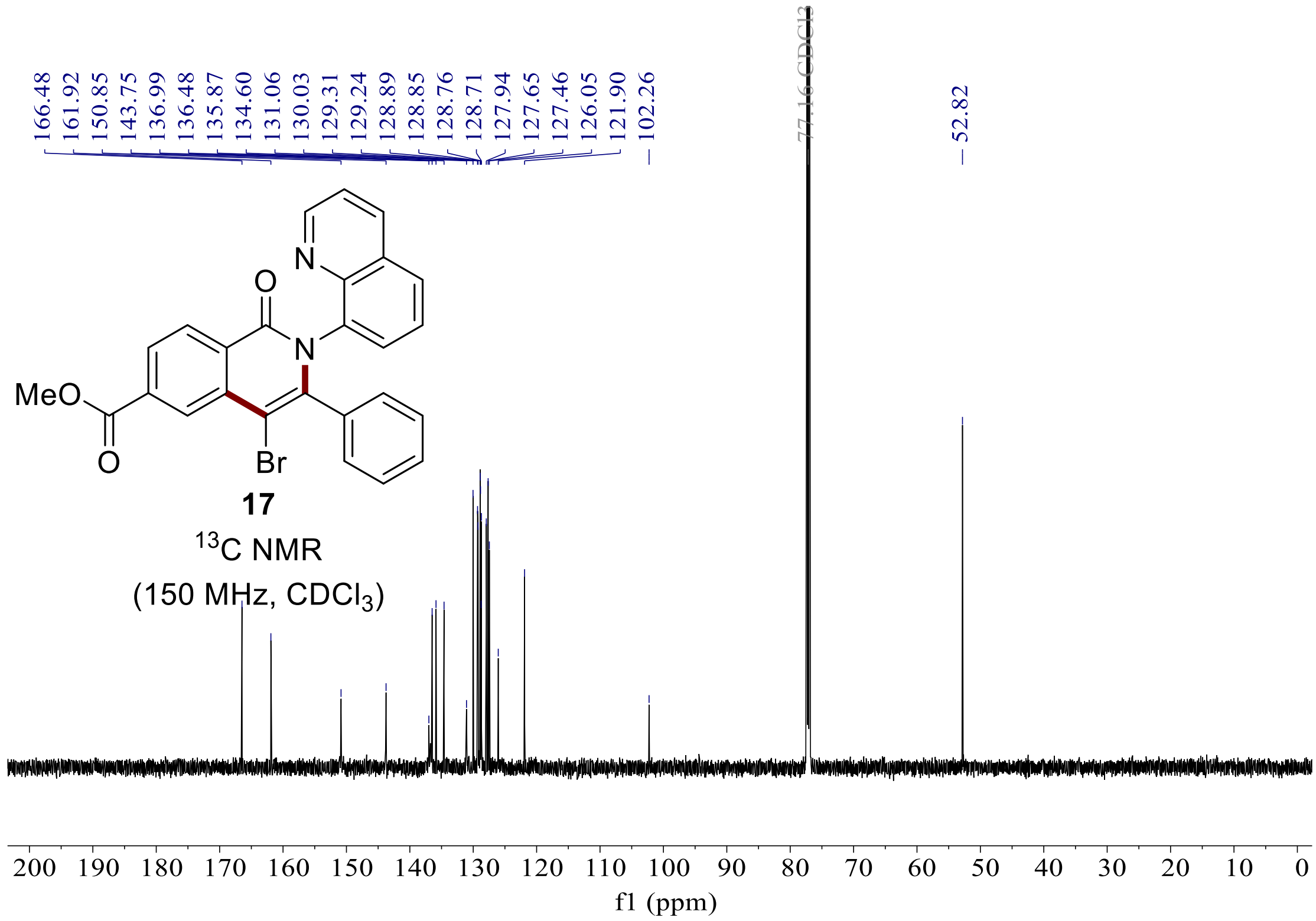
166.48
161.92
150.85
143.75
136.99
136.48
135.87
134.60
131.06
130.03
129.31
129.24
128.89
128.85
128.76
128.71
127.94
127.65
127.46
126.05
121.90
102.26
77.16 CDCl3
52.82
MeO
O
O
N
N
Br
17
^{13}C NMR
(150 MHz, CDCl$_3$)
200 190 180 170 160 150 140 130 120 110 100 90 80 70 60 50 40 30 20 10 0
f1 (ppm)

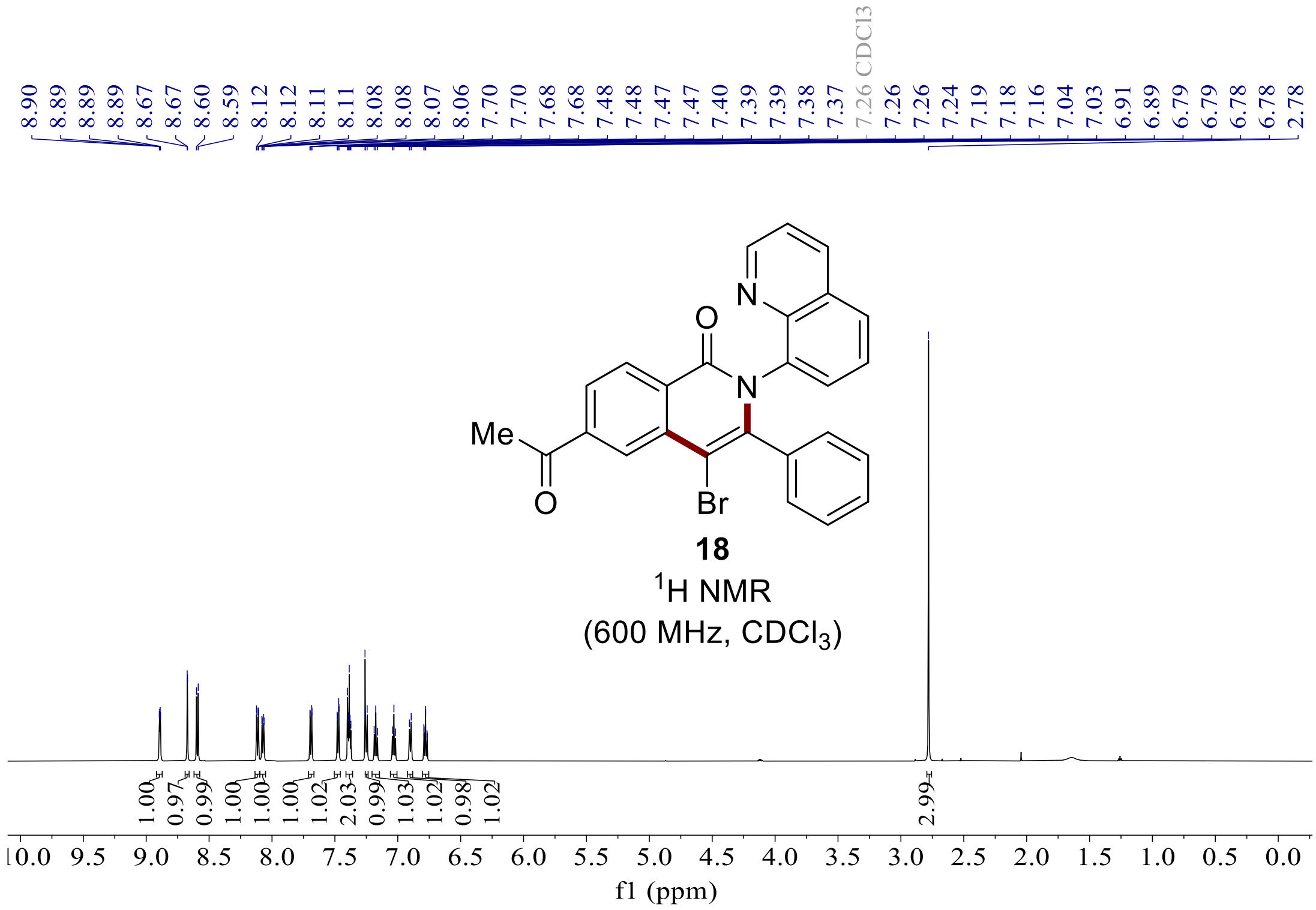
8.90
8.89
8.89
8.89
8.67
8.67
8.60
8.59
8.12
8.12
8.11
8.11
8.08
8.08
8.07
8.06
7.70
7.70
7.68
7.68
7.48
7.48
7.47
7.47
7.40
7.39
7.39
7.38
7.37
7.26 CDCl3
7.26
7.24
7.19
7.18
7.16
7.04
7.03
6.91
6.89
6.79
6.79
6.78
6.78
2.78
Me
O
O
N
N
Br
18
^{1}H NMR
(600 MHz, CDCl$_3$)
1.00
0.97
0.99
1.00
1.00
1.00
1.02
2.03
0.99
1.03
1.02
0.98
1.02
2.99
10.0 9.5 9.0 8.5 8.0 7.5 7.0 6.5 6.0 5.5 5.0 4.5 4.0 3.5 3.0 2.5 2.0 1.5 1.0 0.5 0.0
f1 (ppm)

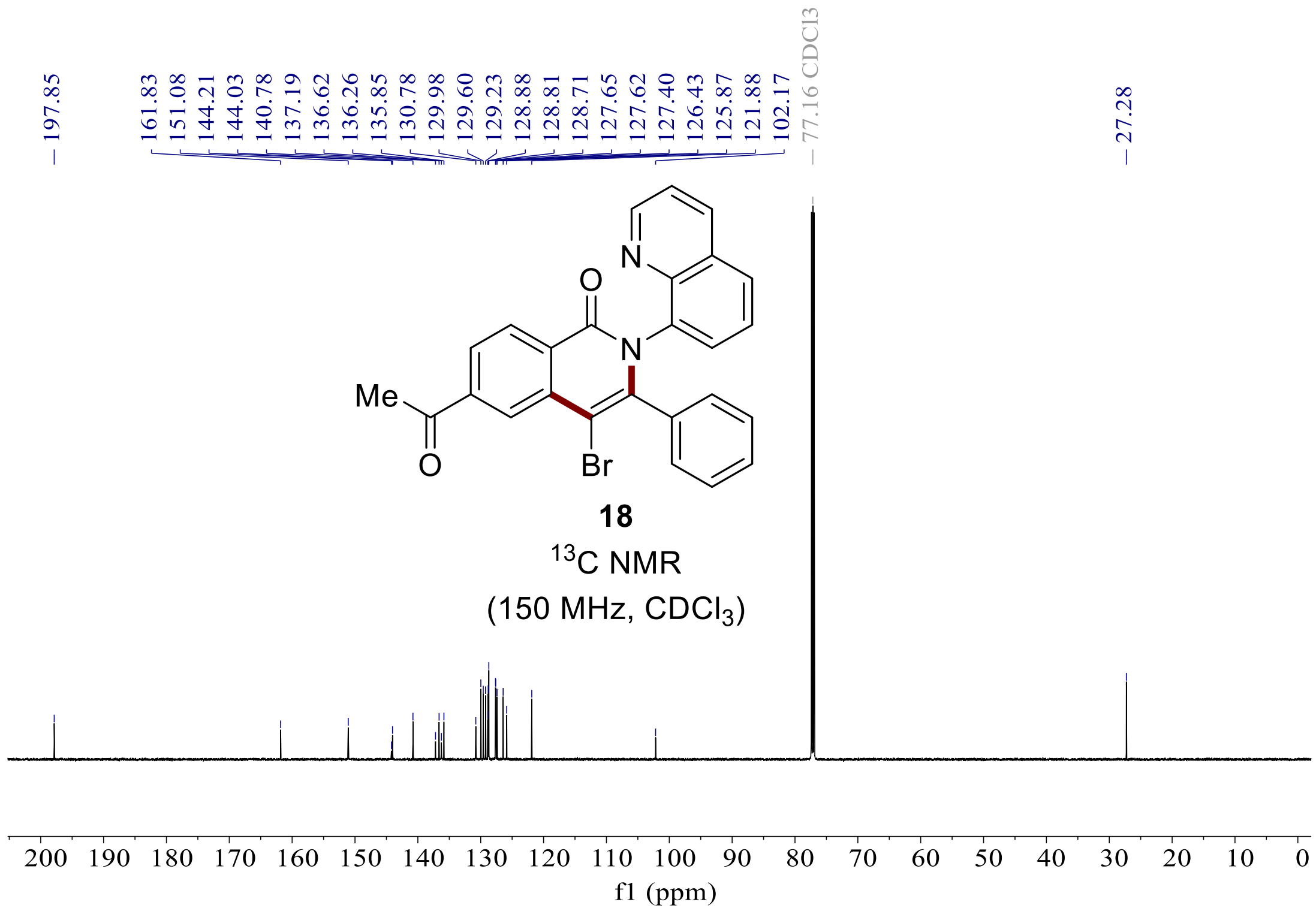
197.85
161.83
151.08
144.21
144.03
140.78
137.19
136.62
136.26
135.85
130.78
129.98
129.60
129.23
128.88
128.81
128.71
127.65
127.62
127.40
126.43
125.87
121.88
102.17
77.16 CDCl3
27.28
Me
O
N
N
Br
O
18
13C NMR
(150 MHz, CDCl3)
200 190 180 170 160 150 140 130 120 110 100 90 80 70 60 50 40 30 20 10 0
f1 (ppm)

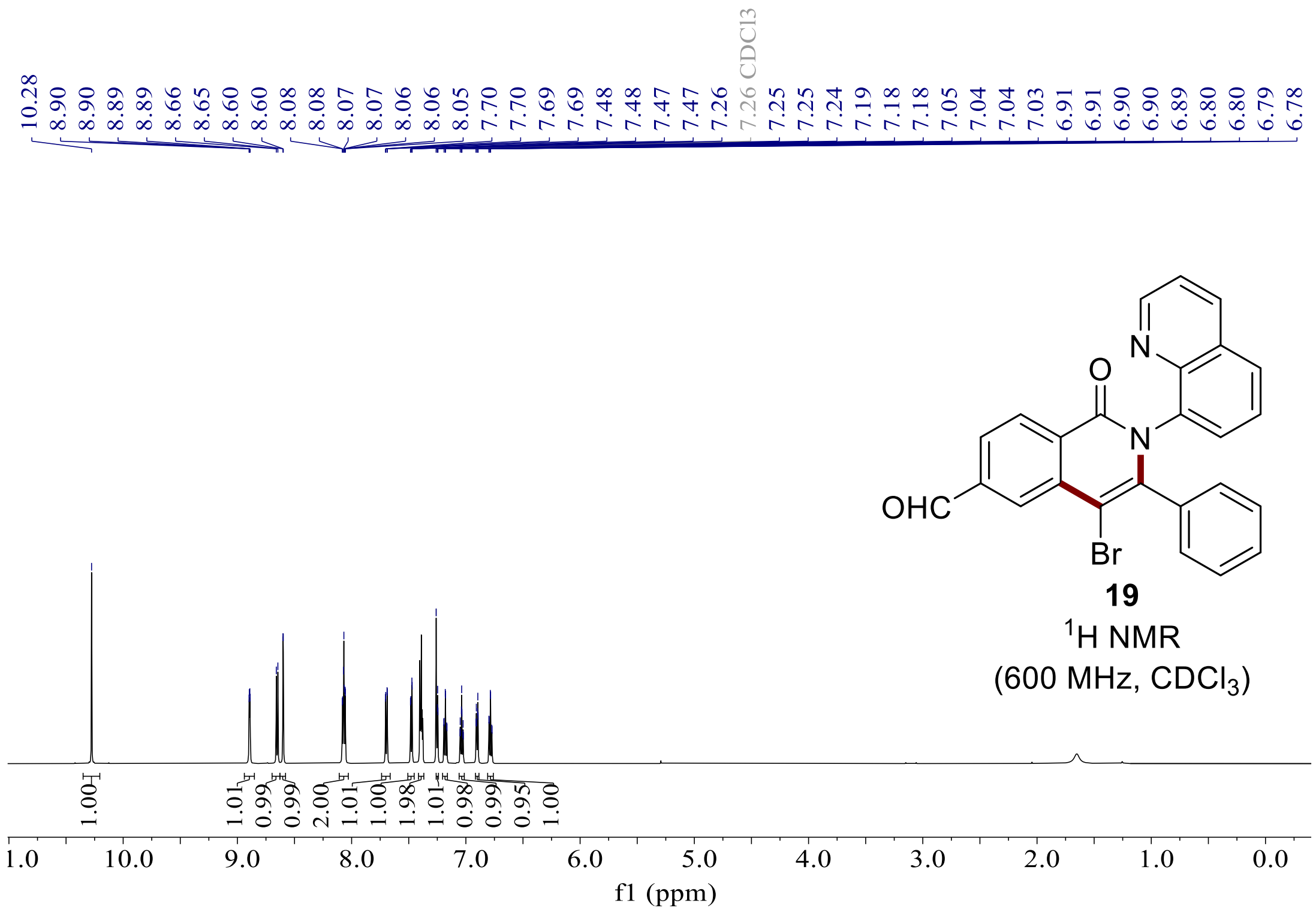
10.28
8.90
8.90
8.89
8.89
8.66
8.65
8.60
8.60
8.08
8.08
8.07
8.07
8.06
8.06
8.05
7.70
7.70
7.69
7.69
7.48
7.48
7.47
7.47
7.26
7.26 CDCl3
7.25
7.25
7.24
7.19
7.18
7.18
7.05
7.04
7.04
7.03
6.91
6.91
6.90
6.90
6.89
6.80
6.80
6.79
6.78
OHC
O
N
N
Br
19
1H NMR
(600 MHz, CDCl3)
1.00
1.01
0.99
0.99
2.00
1.01
1.00
1.98
1.01
0.98
0.99
0.95
1.00
1.0 10.0 9.0 8.0 7.0 6.0 5.0 4.0 3.0 2.0 1.0 0.0
f1 (ppm)

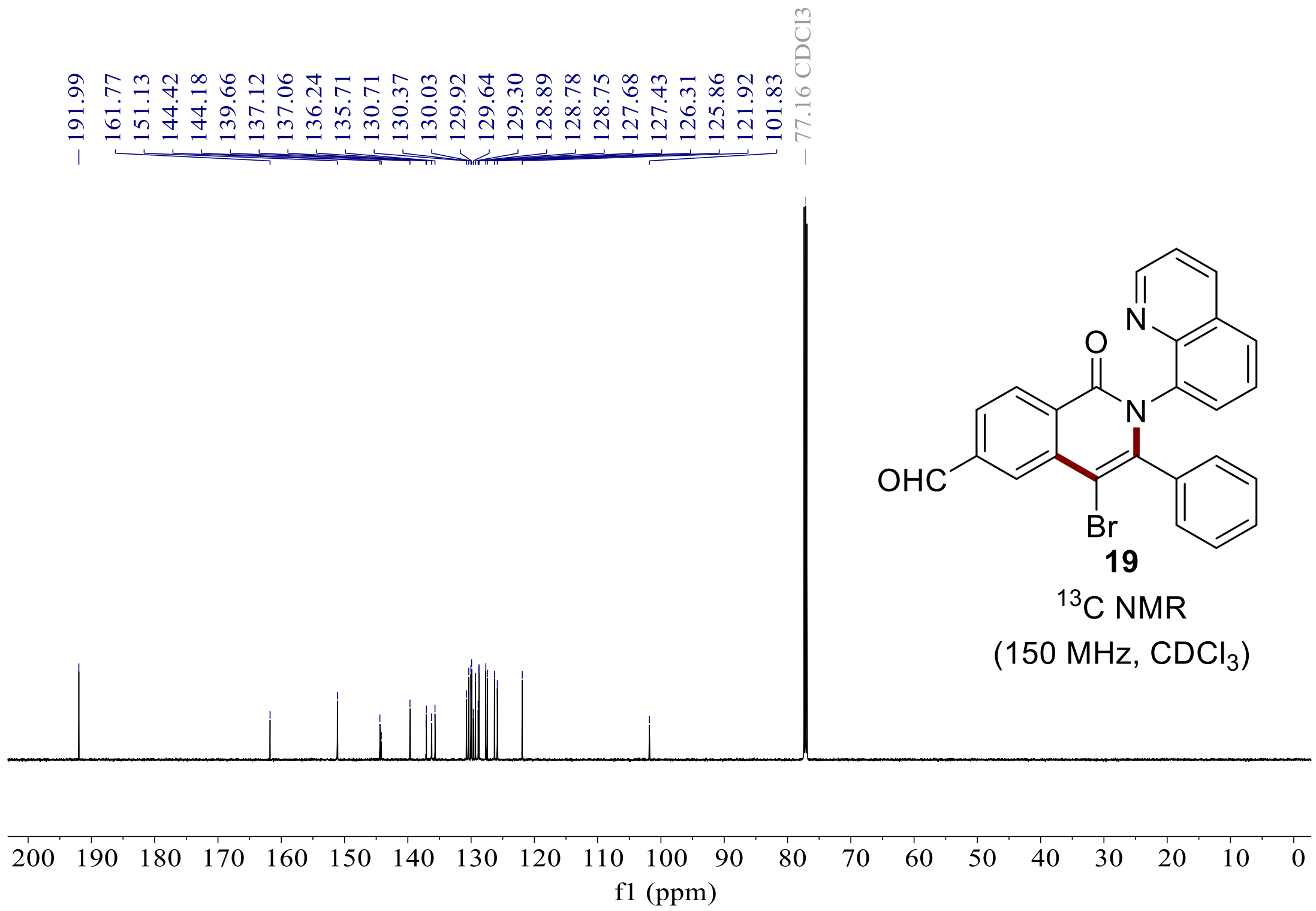

19

^{13}C NMR

(150 MHz, $CDCl_3$)

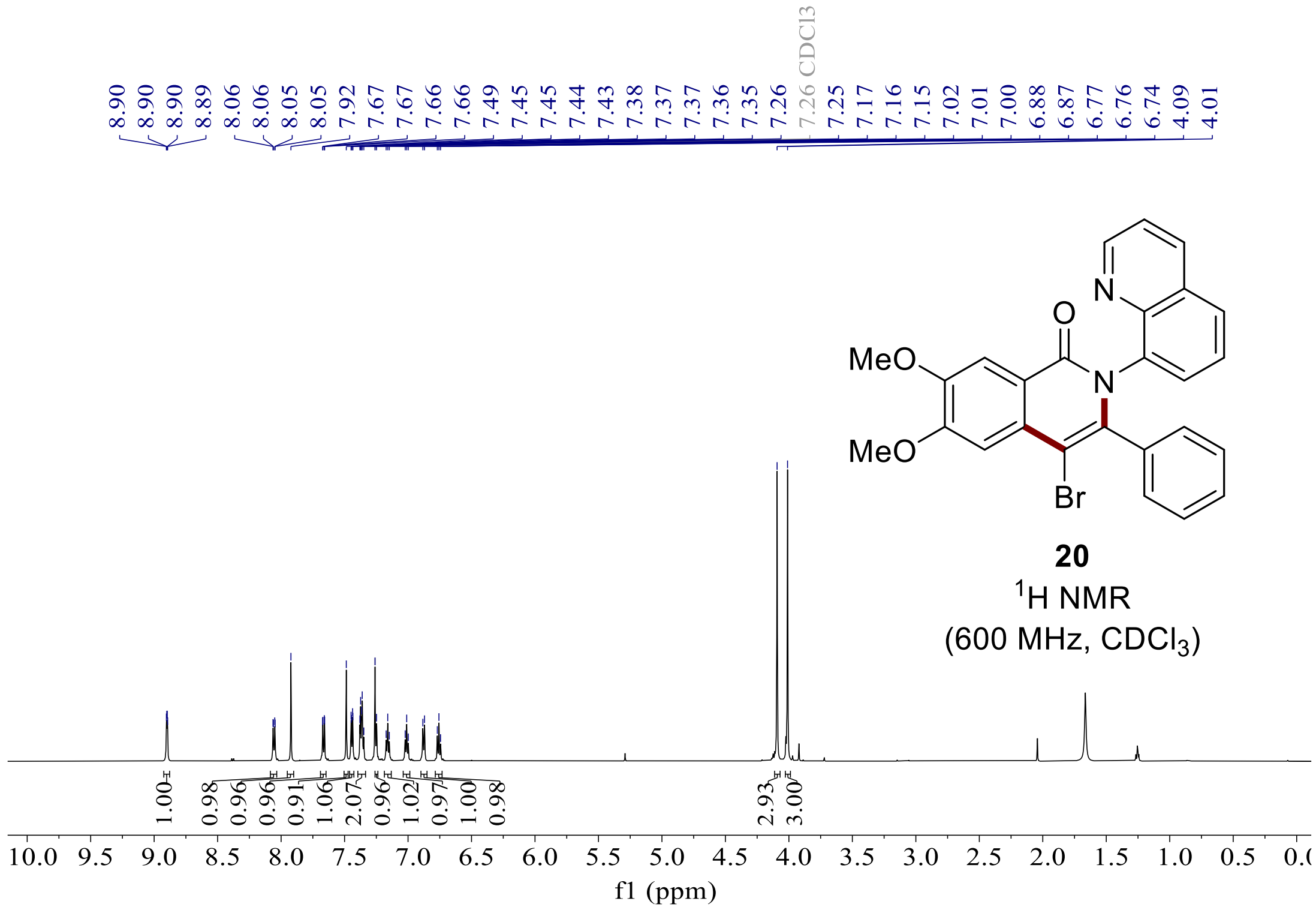

20

^{1}H NMR

(600 MHz, $CDCl_3$)

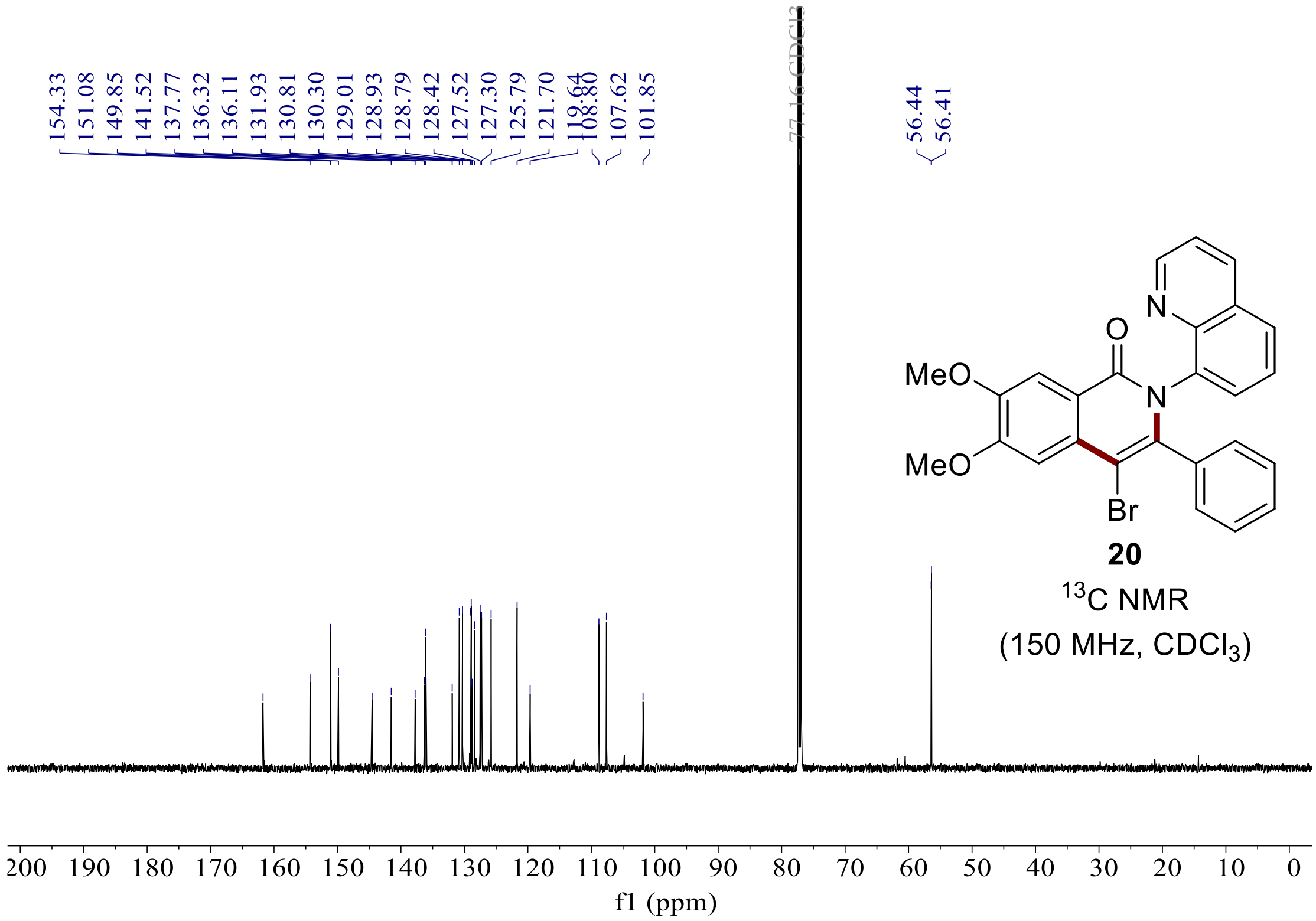
154.33
151.08
149.85
141.52
137.77
136.32
136.11
131.93
130.81
130.30
129.01
128.93
128.79
128.42
127.52
127.30
125.79
121.70
119.64
108.80
107.62
101.85
77.16 CDCl3
56.44
56.41
MeO
MeO
O
N
N
Br
20
^{13}C NMR
(150 MHz, $CDCl_3$)
200 190 180 170 160 150 140 130 120 110 100 90 80 70 60 50 40 30 20 10 0
f1 (ppm)

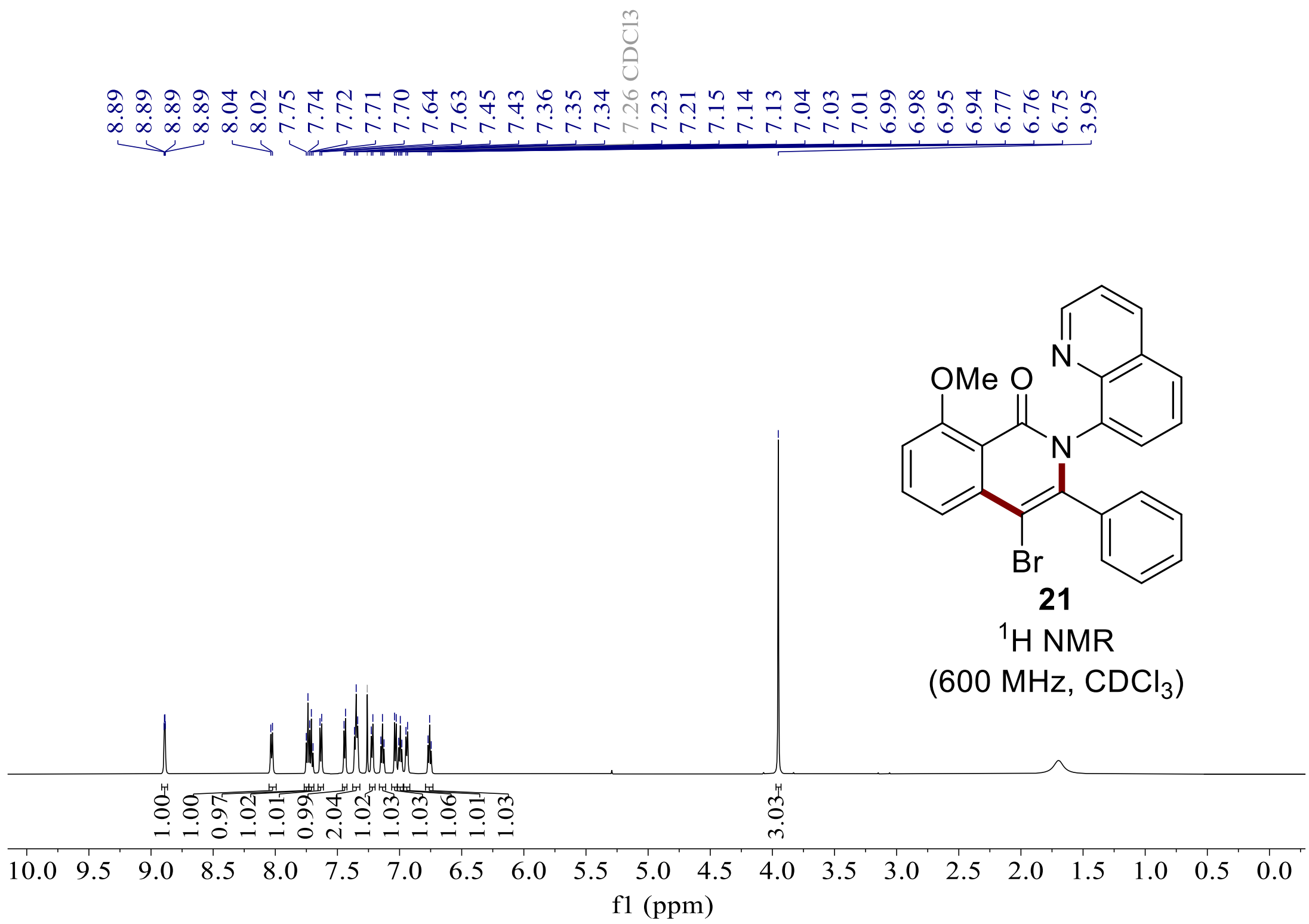
8.89
8.89
8.89
8.89
8.04
8.02
7.75
7.74
7.72
7.71
7.70
7.64
7.63
7.45
7.43
7.36
7.35
7.34
7.26 CDCl3
7.23
7.21
7.15
7.14
7.13
7.04
7.03
7.01
6.99
6.98
6.95
6.94
6.77
6.76
6.75
3.95
OMe
O
N
N
Br
21
^{1}H NMR
(600 MHz, $CDCl_3$)
1.00
1.00
0.97
1.02
1.01
0.99
2.04
1.02
1.03
1.03
1.06
1.01
1.03
3.03
10.0 9.5 9.0 8.5 8.0 7.5 7.0 6.5 6.0 5.5 5.0 4.5 4.0 3.5 3.0 2.5 2.0 1.5 1.0 0.5 0.0
f1 (ppm)

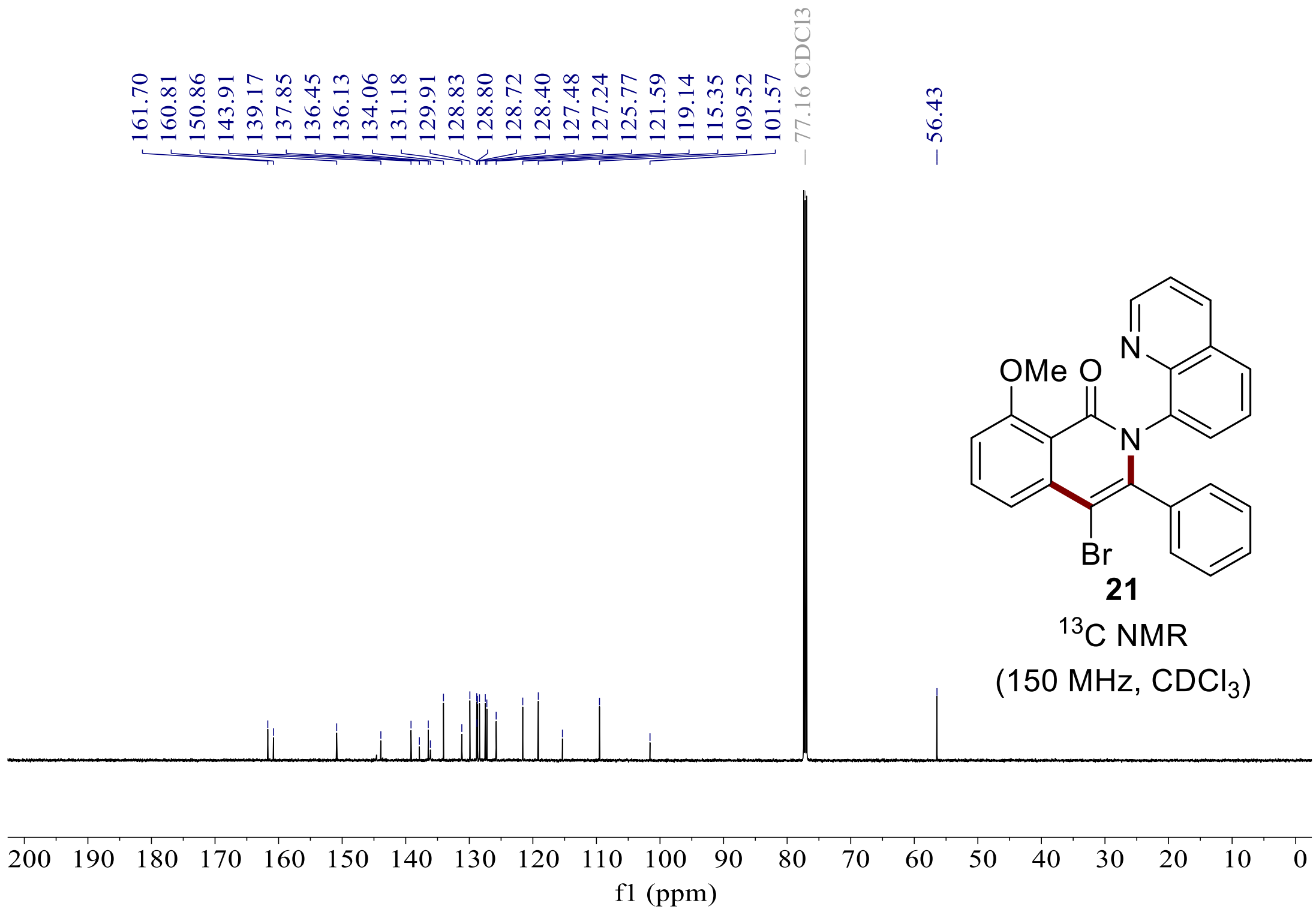
161.70
160.81
150.86
143.91
139.17
137.85
136.45
136.13
134.06
131.18
129.91
128.83
128.80
128.72
128.40
127.48
127.24
125.77
121.59
119.14
115.35
109.52
101.57
77.16 CDCl3
56.43
OMe
O
N
N
Br
21
^{13}C NMR
(150 MHz, $CDCl_3$)
200 190 180 170 160 150 140 130 120 110 100 90 80 70 60 50 40 30 20 10 0
f1 (ppm)

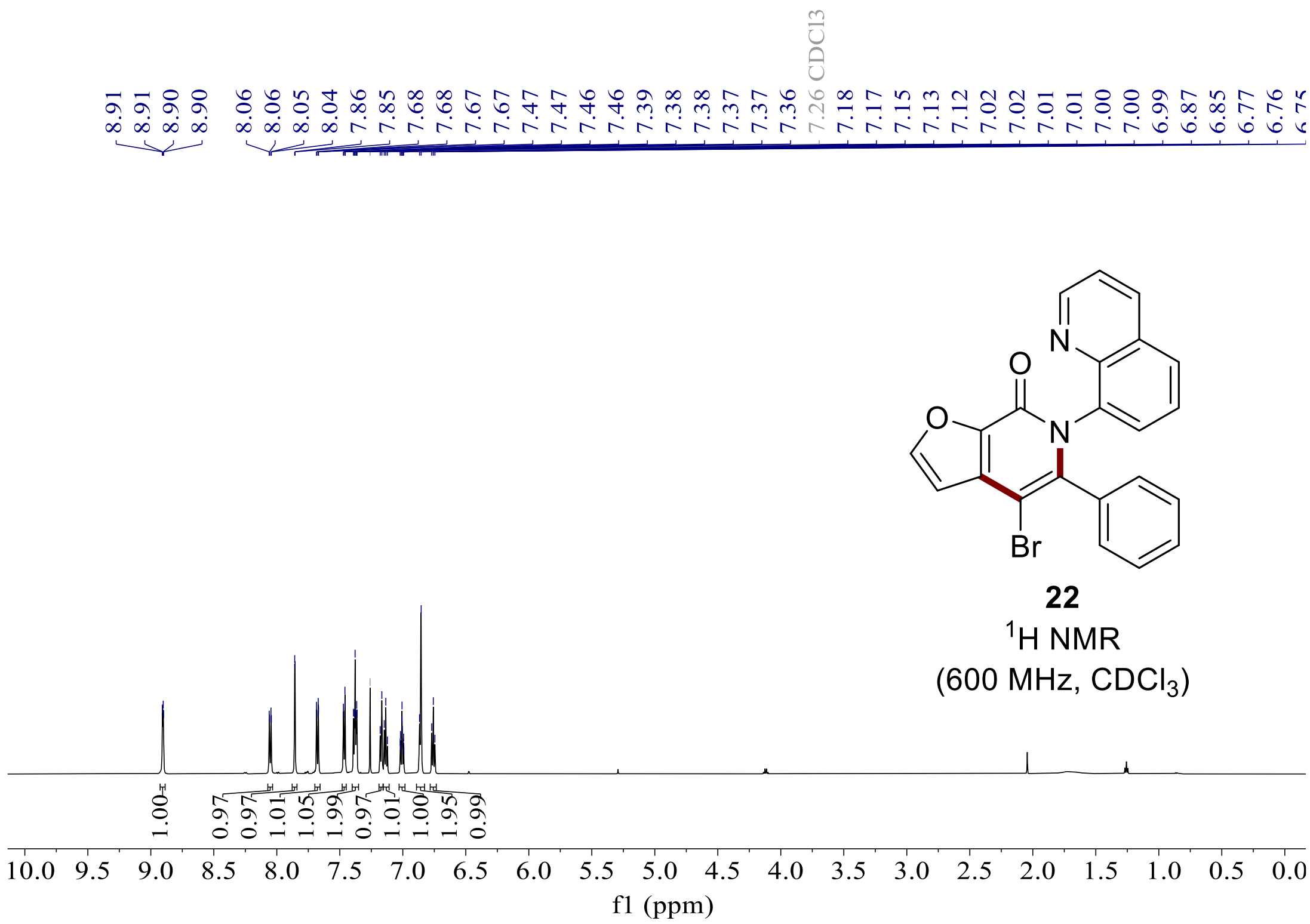
8.91
8.91
8.90
8.90
8.06
8.06
8.05
8.04
7.86
7.85
7.68
7.68
7.67
7.67
7.47
7.46
7.46
7.39
7.38
7.38
7.37
7.37
7.36
7.26 CDCl3
7.18
7.17
7.15
7.13
7.12
7.02
7.01
7.01
7.00
7.00
6.99
6.87
6.85
6.77
6.76
O
N
O
N
Br
22
^{1}H NMR
(600 MHz, $CDCl_3$)
1.00
0.97
0.97
1.01
1.05
1.99
0.97
1.01
1.00
1.95
0.99
10.0 9.5 9.0 8.5 8.0 7.5 7.0 6.5 6.0 5.5 5.0 4.5 4.0 3.5 3.0 2.5 2.0 1.5 1.0 0.5 0.0
f1 (ppm)

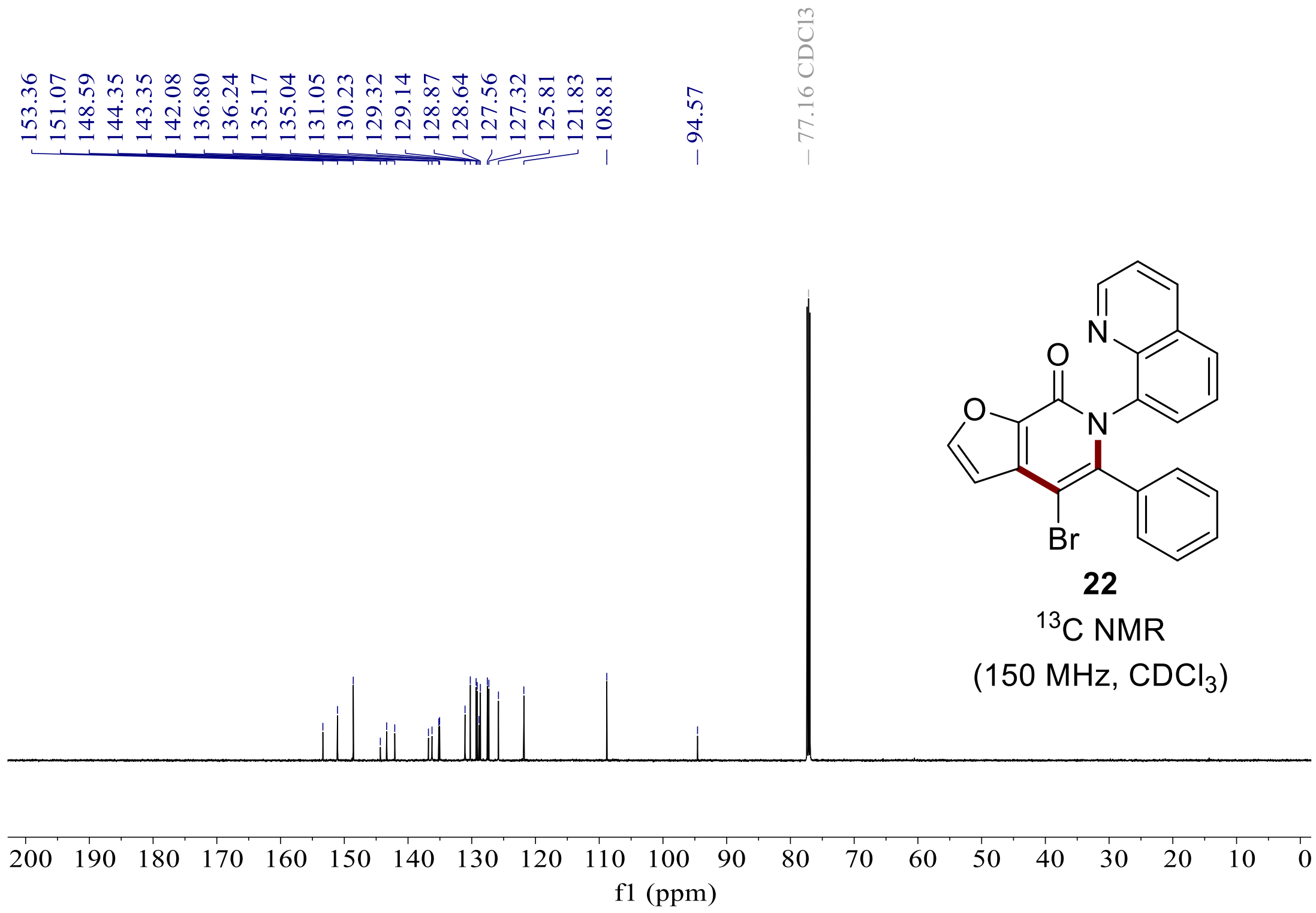
153.36
151.07
148.59
144.35
143.35
142.08
136.80
136.24
135.17
135.04
131.05
130.23
129.32
129.14
128.87
128.64
127.56
127.32
125.81
121.83
108.81
94.57
77.16 CDCl3
O
N
O
N
Br
22
^{13}C NMR
(150 MHz, $CDCl_3$)
200 190 180 170 160 150 140 130 120 110 100 90 80 70 60 50 40 30 20 10 0
f1 (ppm)

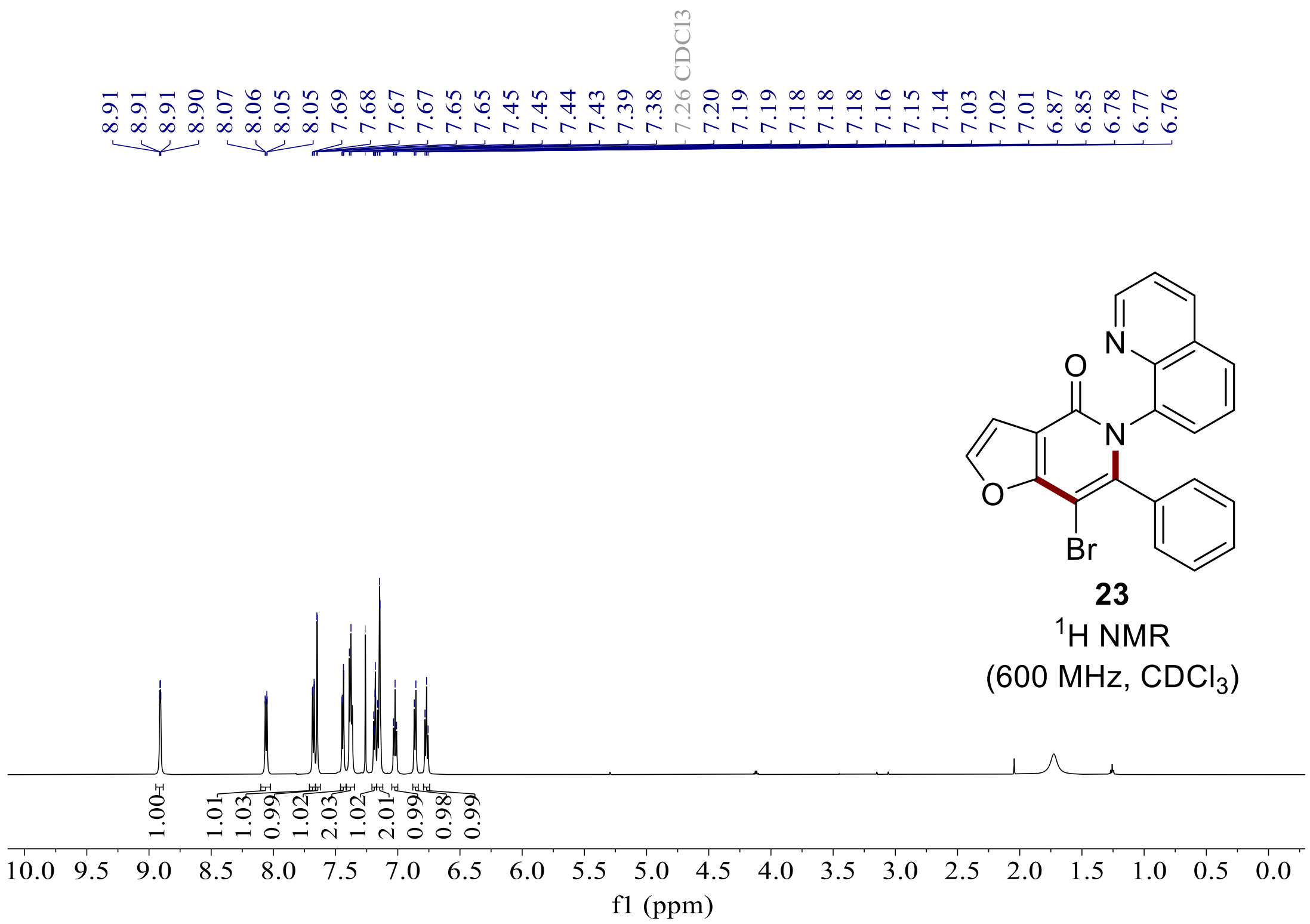
8.91
8.91
8.91
8.90
8.07
8.06
8.05
8.05
7.69
7.68
7.67
7.67
7.65
7.65
7.45
7.45
7.44
7.43
7.39
7.38
7.26 CDCl3
7.20
7.19
7.19
7.18
7.18
7.18
7.16
7.15
7.14
7.03
7.02
7.01
6.87
6.85
6.78
6.77
6.76
N
O
N
O
Br
23
^{1}H NMR
(600 MHz, $CDCl_3$)
1.00
1.01
1.03
0.99
1.02
2.03
1.02
2.01
0.99
0.98
0.99
10.0 9.5 9.0 8.5 8.0 7.5 7.0 6.5 6.0 5.5 5.0 4.5 4.0 3.5 3.0 2.5 2.0 1.5 1.0 0.5 0.0
f1 (ppm)

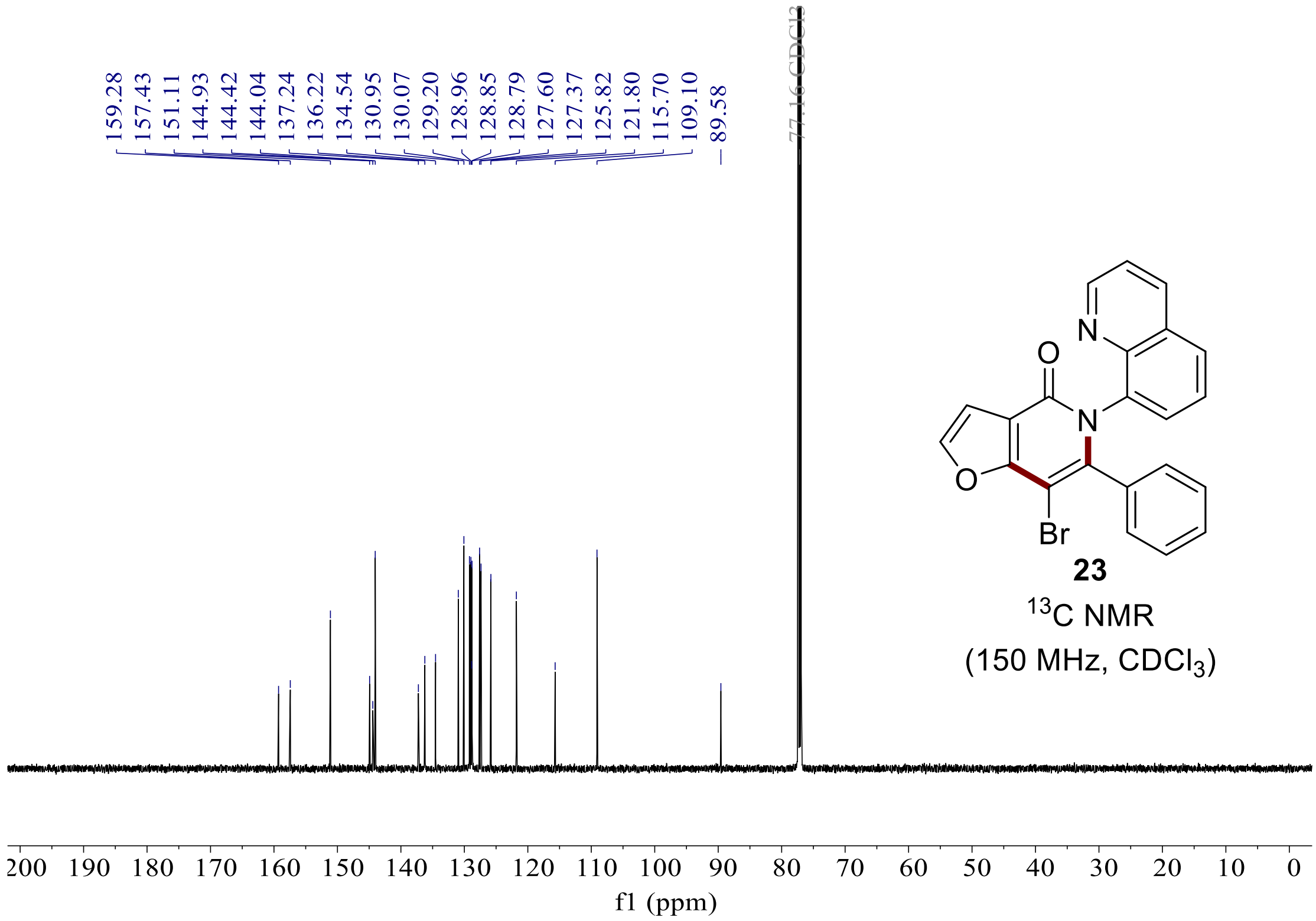
159.28 157.43 151.11 144.93 144.42 144.04 137.24 136.22 134.54 130.95 130.07 129.20 128.96 128.85 128.79 127.60 127.37 125.82 121.80 115.70 109.10 89.58
77.16 CDCl3
N
O
N
O
Br
23
13C NMR
(150 MHz, CDCl3)
200 190 180 170 160 150 140 130 120 110 100 90 80 70 60 50 40 30 20 10 0
f1 (ppm)

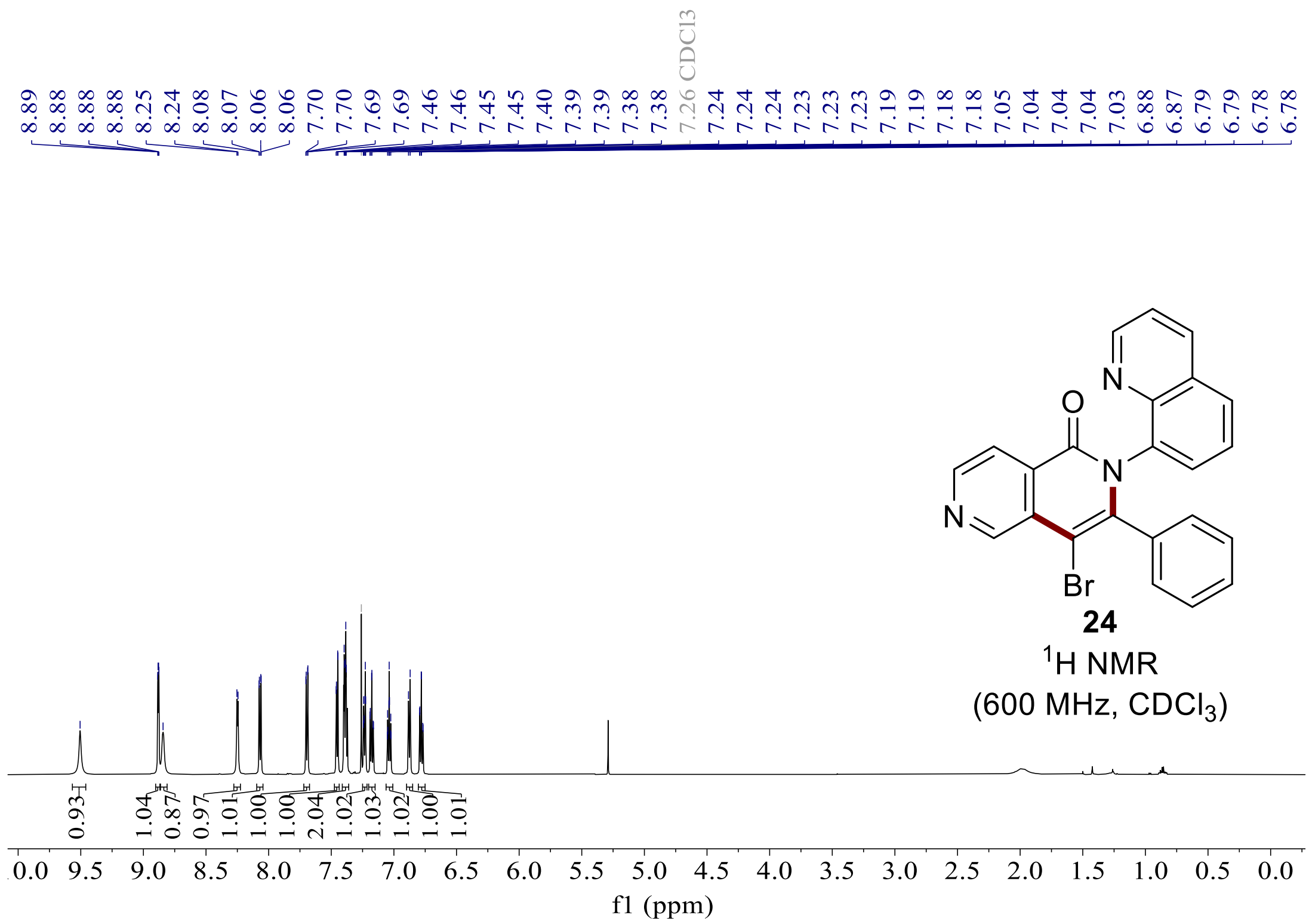
8.89 8.88 8.88 8.88 8.25 8.24 8.08 8.07 8.06 8.06 7.70 7.70 7.69 7.69 7.46 7.46 7.45 7.45 7.40 7.39 7.39 7.38 7.38 7.26 CDCl3 7.24 7.24 7.24 7.23 7.23 7.23 7.19 7.19 7.18 7.18 7.05 7.04 7.04 7.04 7.03 6.88 6.87 6.79 6.79 6.78 6.78
N
O
N
N
Br
24
1H NMR
(600 MHz, CDCl3)
0.93 1.04 0.87 0.97 1.01 1.00 1.00 2.04 1.02 1.03 1.02 1.00 1.01
10.0 9.5 9.0 8.5 8.0 7.5 7.0 6.5 6.0 5.5 5.0 4.5 4.0 3.5 3.0 2.5 2.0 1.5 1.0 0.5 0.0
f1 (ppm)

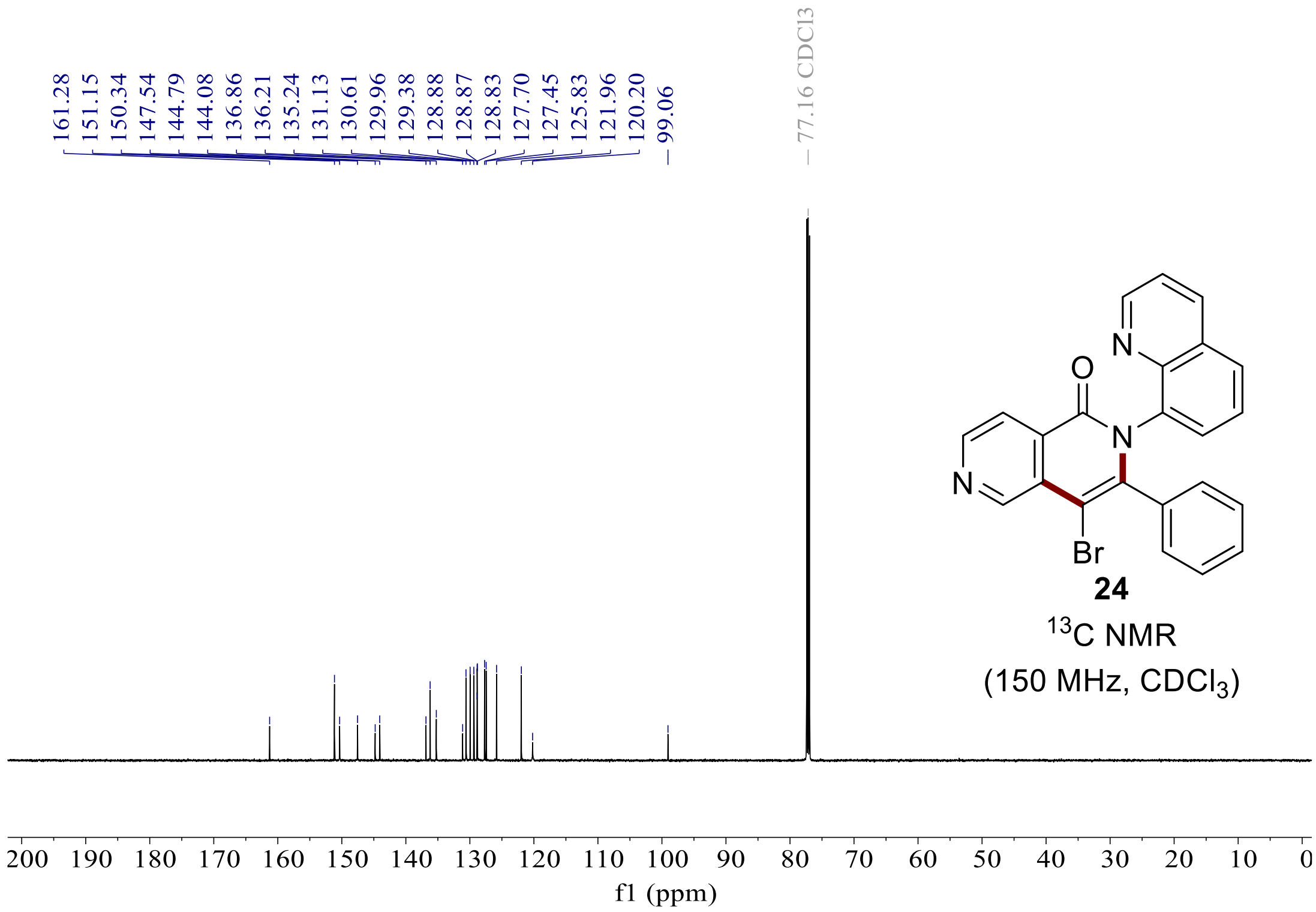

161.28
151.15
150.34
147.54
144.79
144.08
136.86
136.21
135.24
131.13
130.61
129.96
129.38
128.88
128.87
128.83
127.70
127.45
125.83
121.96
120.20
99.06
77.16 CDCl3
O
N
N
N
Br
24
^{13}C NMR
(150 MHz, $CDCl_3$)
200 190 180 170 160 150 140 130 120 110 100 90 80 70 60 50 40 30 20 10 0
f1 (ppm)

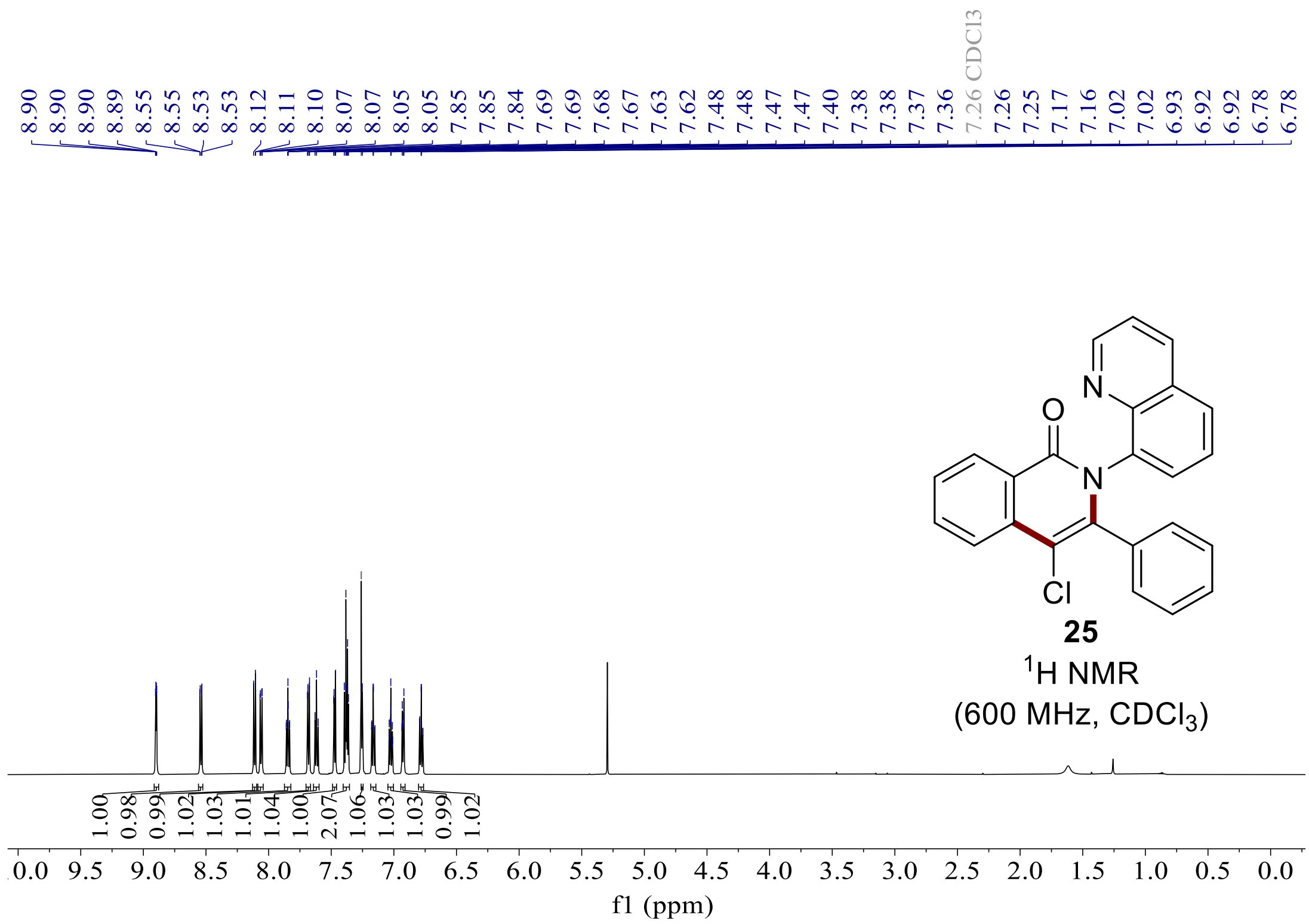

8.90
8.90
8.90
8.89
8.55
8.55
8.53
8.53
8.12
8.11
8.10
8.07
8.07
8.05
8.05
7.85
7.85
7.84
7.69
7.69
7.68
7.67
7.63
7.62
7.48
7.48
7.47
7.47
7.40
7.38
7.38
7.37
7.36
7.26 CDCl3
7.26
7.25
7.17
7.16
7.02
7.02
6.93
6.92
6.92
6.78
6.78
O
N
N
Cl
25
^{1}H NMR
(600 MHz, $CDCl_3$)
1.00
0.98
0.99
1.02
1.03
1.01
1.04
1.00
2.07
1.06
1.03
1.03
0.99
1.02
0.0 9.5 9.0 8.5 8.0 7.5 7.0 6.5 6.0 5.5 5.0 4.5 4.0 3.5 3.0 2.5 2.0 1.5 1.0 0.5 0.0
f1 (ppm)

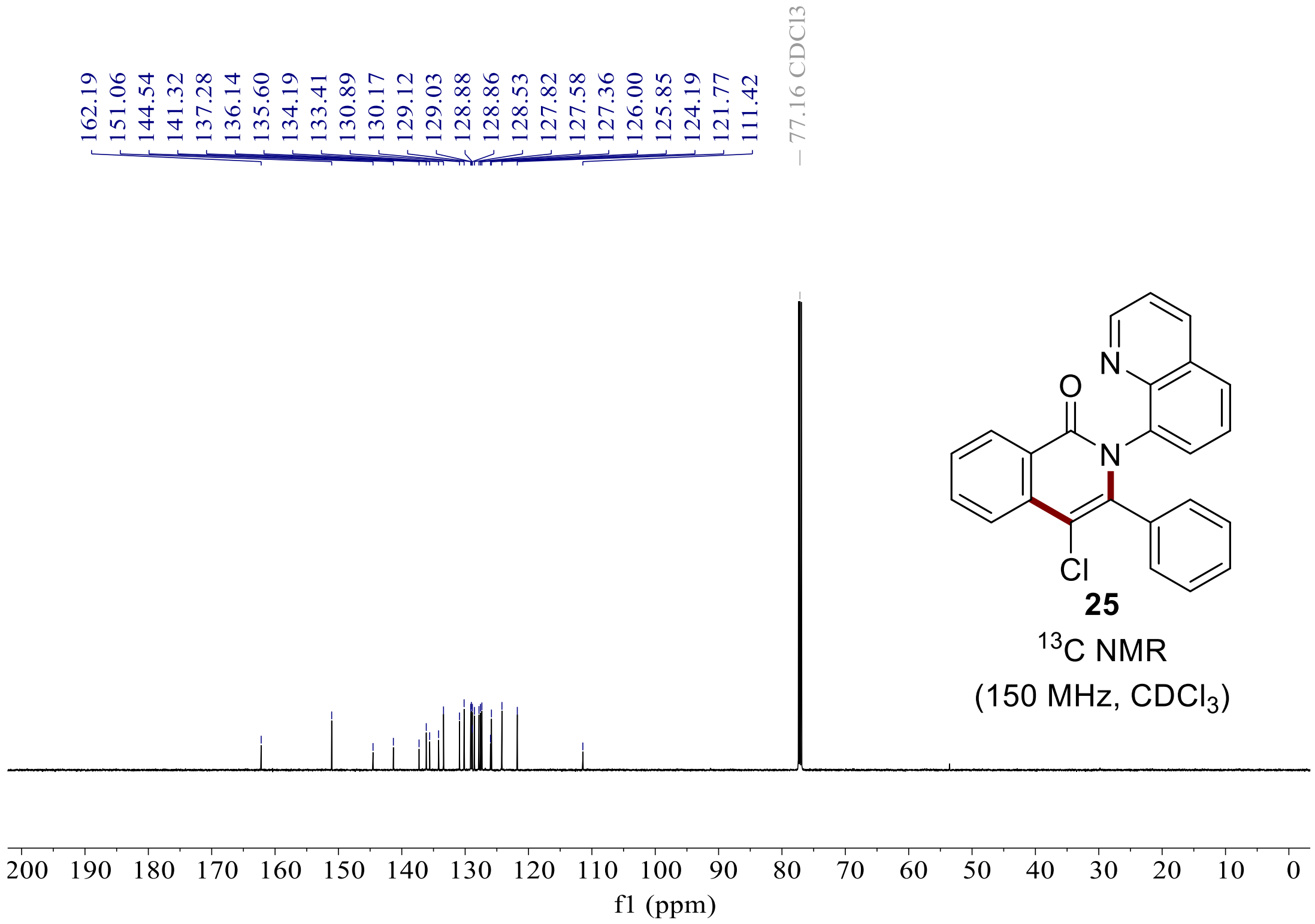
162.19
151.06
144.54
141.32
137.28
136.14
135.60
134.19
133.41
130.89
130.17
129.12
129.03
128.88
128.86
128.53
127.82
127.58
127.36
126.00
125.85
124.19
121.77
111.42
77.16 CDCl3
O
N
N
Cl
25
13C NMR
(150 MHz, CDCl3)
200 190 180 170 160 150 140 130 120 110 100 90 80 70 60 50 40 30 20 10 0
f1 (ppm)

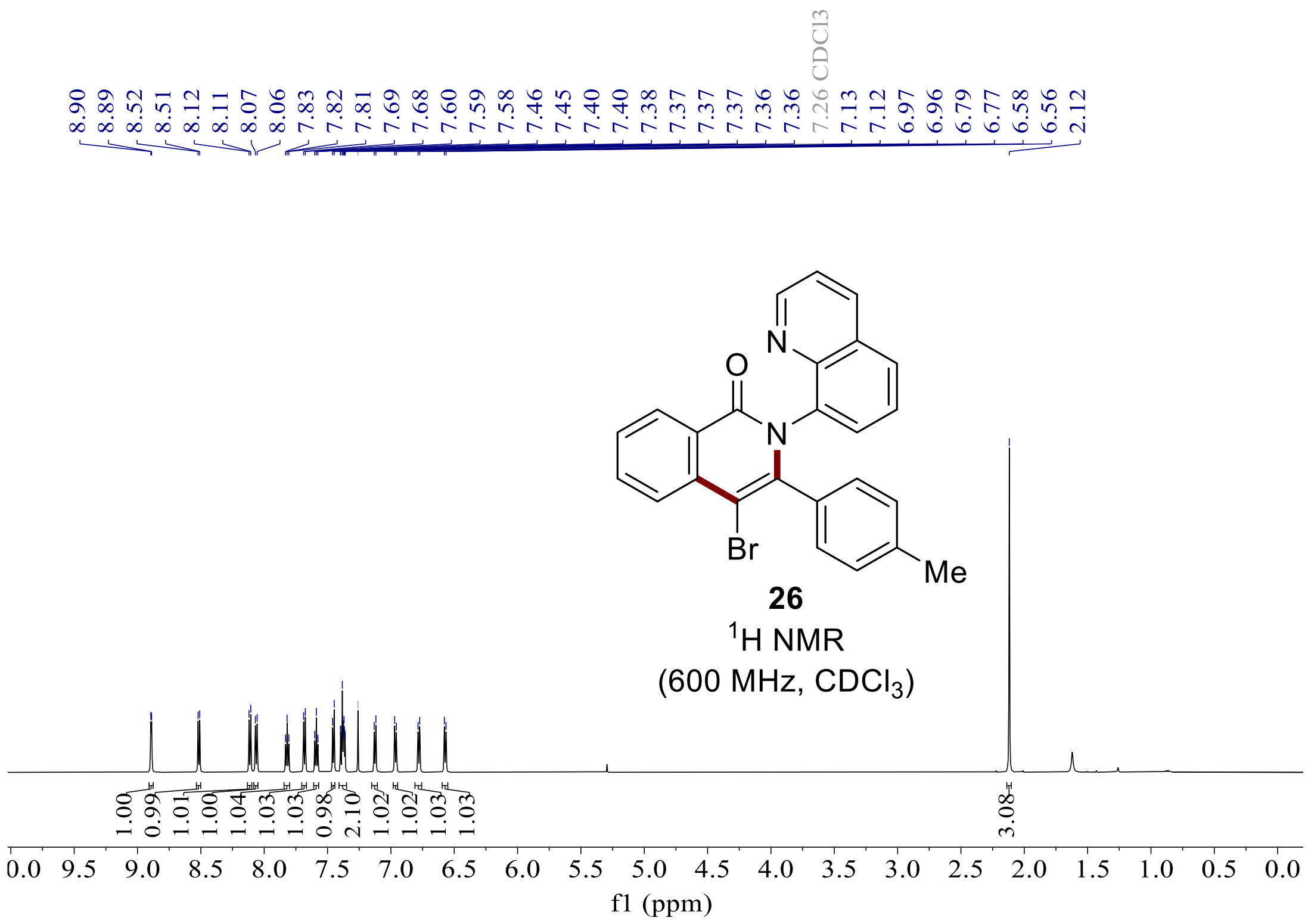
8.90
8.89
8.52
8.51
8.12
8.11
8.07
8.06
7.83
7.82
7.81
7.69
7.68
7.60
7.59
7.58
7.46
7.45
7.40
7.40
7.38
7.37
7.37
7.37
7.36
7.36
7.26 CDCl3
7.13
7.12
6.97
6.96
6.79
6.77
6.58
6.56
2.12
O
N
N
Br
Me
26
1H NMR
(600 MHz, CDCl3)
1.00
0.99
1.01
1.00
1.04
1.03
1.03
0.98
2.10
1.02
1.02
1.03
1.03
3.08
0.0 9.5 9.0 8.5 8.0 7.5 7.0 6.5 6.0 5.5 5.0 4.5 4.0 3.5 3.0 2.5 2.0 1.5 1.0 0.5 0.0
f1 (ppm)

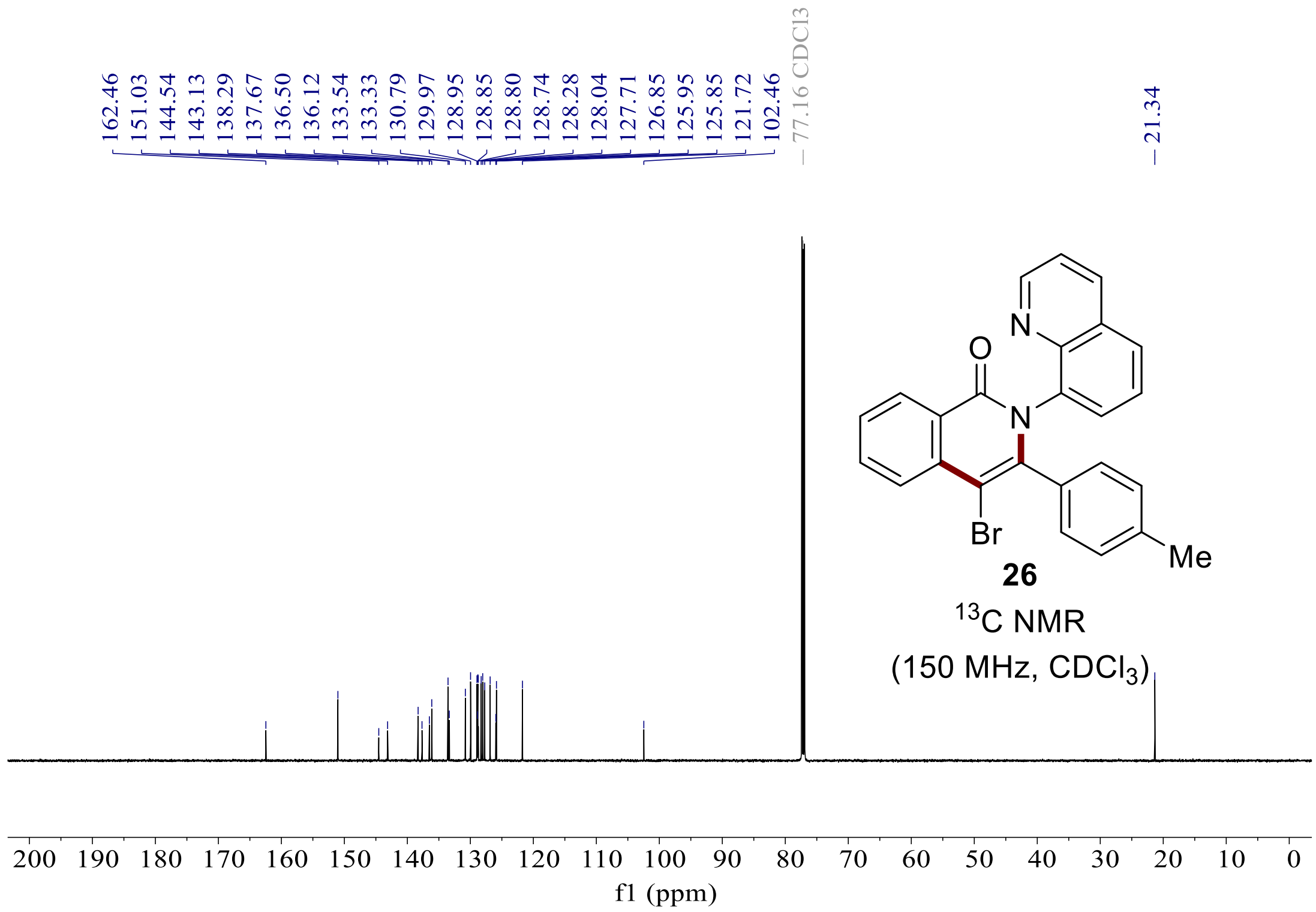
162.46
151.03
144.54
143.13
138.29
137.67
136.50
136.12
133.54
133.33
130.79
129.97
128.95
128.85
128.80
128.74
128.28
128.04
127.71
126.85
125.95
125.85
121.72
102.46
77.16 CDCl3
21.34
N
O
N
Br
Me
26
13C NMR
(150 MHz, CDCl3)
200 190 180 170 160 150 140 130 120 110 100 90 80 70 60 50 40 30 20 10 0
f1 (ppm)

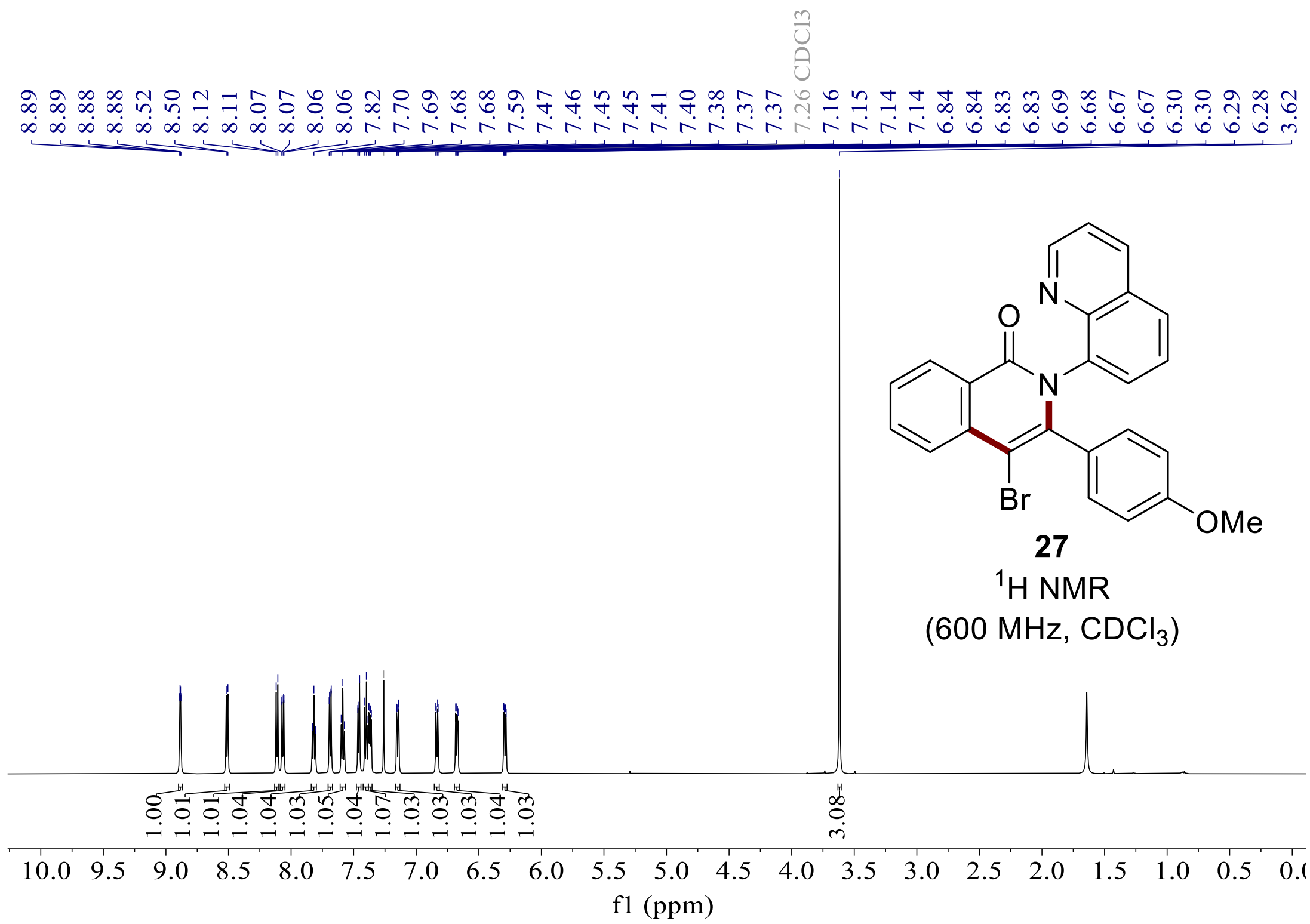
8.89
8.89
8.88
8.88
8.52
8.50
8.12
8.11
8.07
8.07
8.06
8.06
7.82
7.70
7.69
7.68
7.68
7.59
7.47
7.46
7.45
7.45
7.41
7.40
7.38
7.37
7.37
7.26 CDCl3
7.16
7.15
7.14
7.14
6.84
6.84
6.83
6.83
6.69
6.68
6.67
6.67
6.30
6.30
6.29
6.28
3.62
N
O
N
Br
OMe
27
1H NMR
(600 MHz, CDCl3)
1.00
1.01
1.01
1.04
1.04
1.03
1.05
1.04
1.07
1.03
1.03
1.03
1.04
1.03
3.08
10.0 9.5 9.0 8.5 8.0 7.5 7.0 6.5 6.0 5.5 5.0 4.5 4.0 3.5 3.0 2.5 2.0 1.5 1.0 0.5 0.0
f1 (ppm)

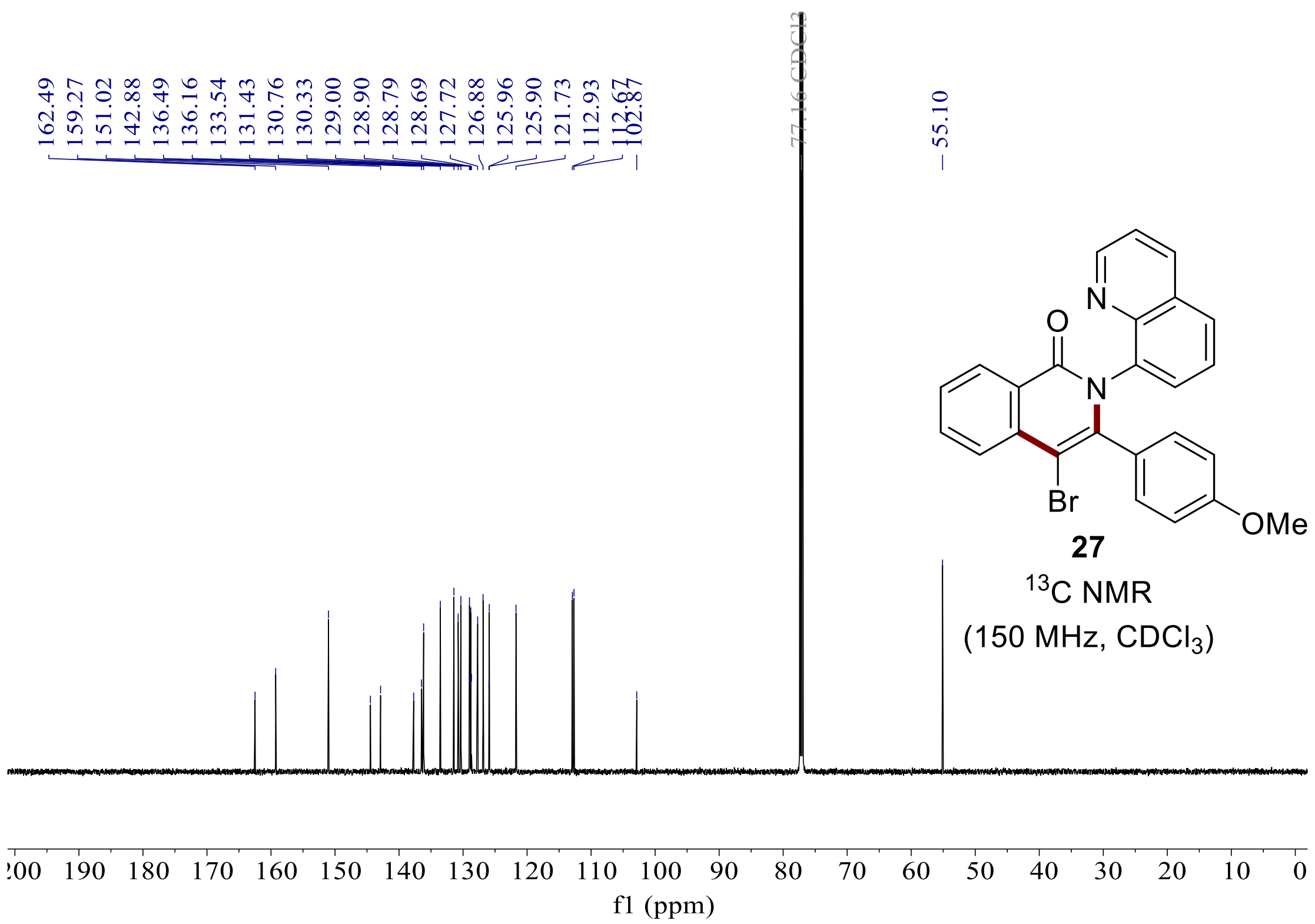
162.49
159.27
151.02
142.88
136.49
136.16
133.54
131.43
130.76
130.33
129.00
128.90
128.79
128.69
127.72
126.88
125.96
125.90
121.73
112.93
112.67
102.87
77.16 CDCl3
55.10
O
N
N
Br
OMe
27
^{13}C NMR
(150 MHz, $CDCl_3$)
200 190 180 170 160 150 140 130 120 110 100 90 80 70 60 50 40 30 20 10 0
f1 (ppm)

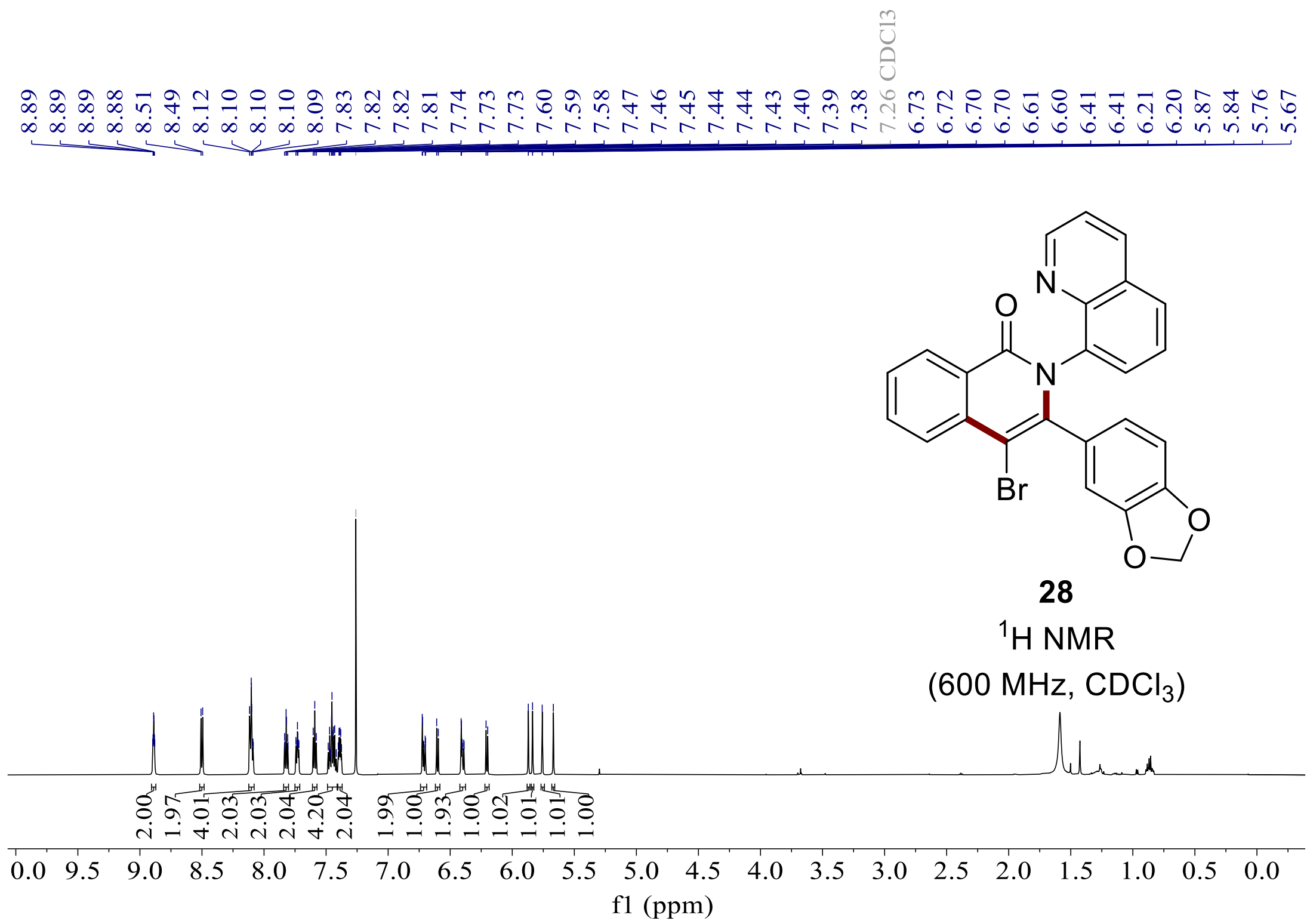
8.89
8.89
8.89
8.88
8.51
8.49
8.12
8.10
8.10
8.10
8.09
7.83
7.82
7.82
7.81
7.74
7.73
7.73
7.60
7.59
7.58
7.47
7.46
7.45
7.44
7.44
7.43
7.40
7.39
7.38
7.26 CDCl3
6.73
6.72
6.70
6.70
6.61
6.60
6.41
6.41
6.21
6.20
5.87
5.84
5.76
5.67
O
N
N
Br
O
O
28
^{1}H NMR
(600 MHz, $CDCl_3$)
2.00
1.97
4.01
2.03
2.03
2.04
4.20
2.04
1.99
1.00
1.93
1.00
1.02
1.01
1.01
1.00
10.0 9.5 9.0 8.5 8.0 7.5 7.0 6.5 6.0 5.5 5.0 4.5 4.0 3.5 3.0 2.5 2.0 1.5 1.0 0.5 0.0
f1 (ppm)

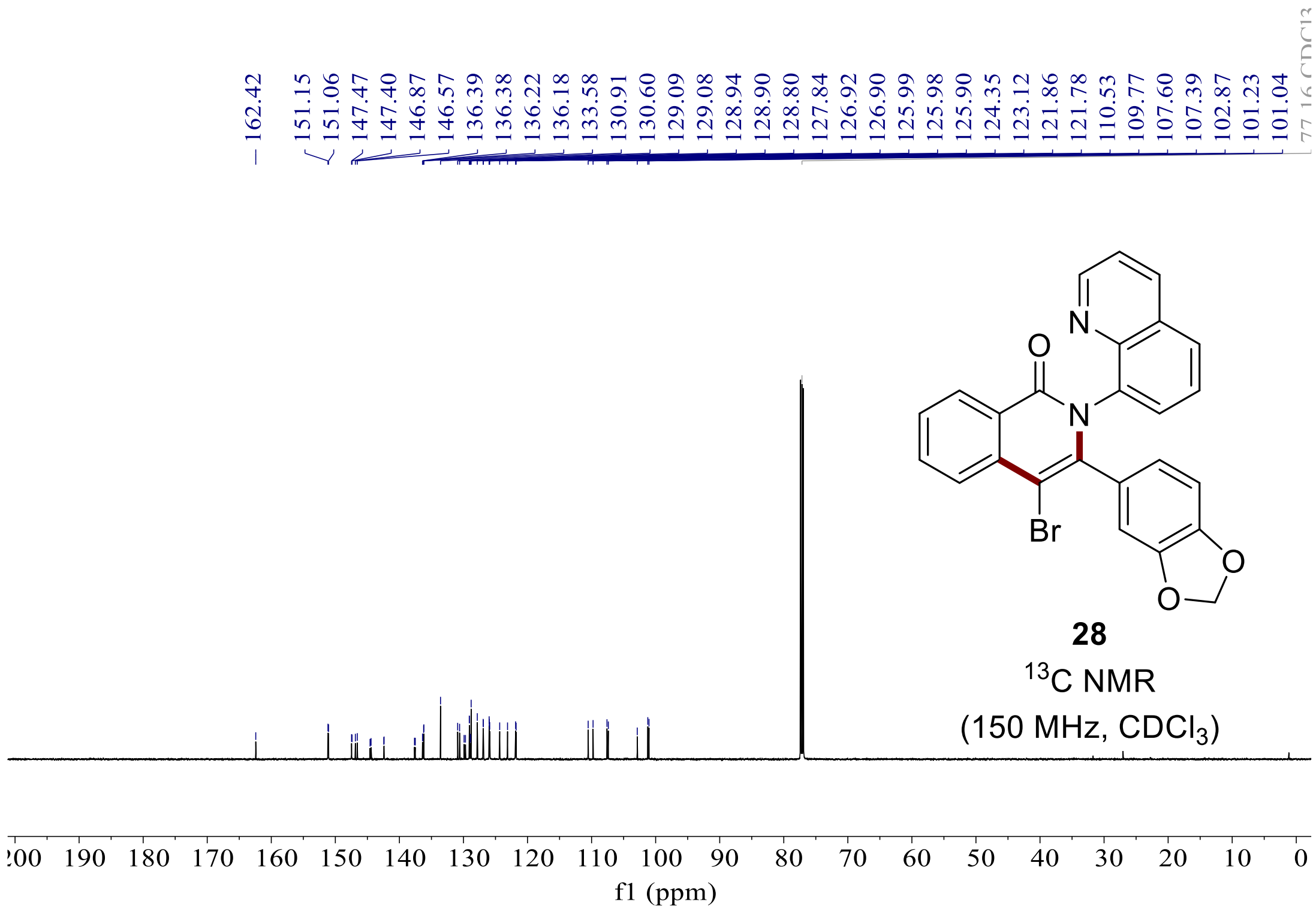
162.42
151.15
151.06
147.47
147.40
146.87
146.57
136.39
136.38
136.22
136.18
133.58
130.91
130.60
129.09
129.08
128.94
128.90
128.80
127.84
126.92
126.90
125.99
125.98
125.90
124.35
123.12
121.86
121.78
110.53
109.77
107.60
107.39
102.87
101.23
101.04
77.16 CDCl3
N
O
N
Br
O
O
28
^{13}C NMR
(150 MHz, CDCl$_3$)
f1 (ppm)

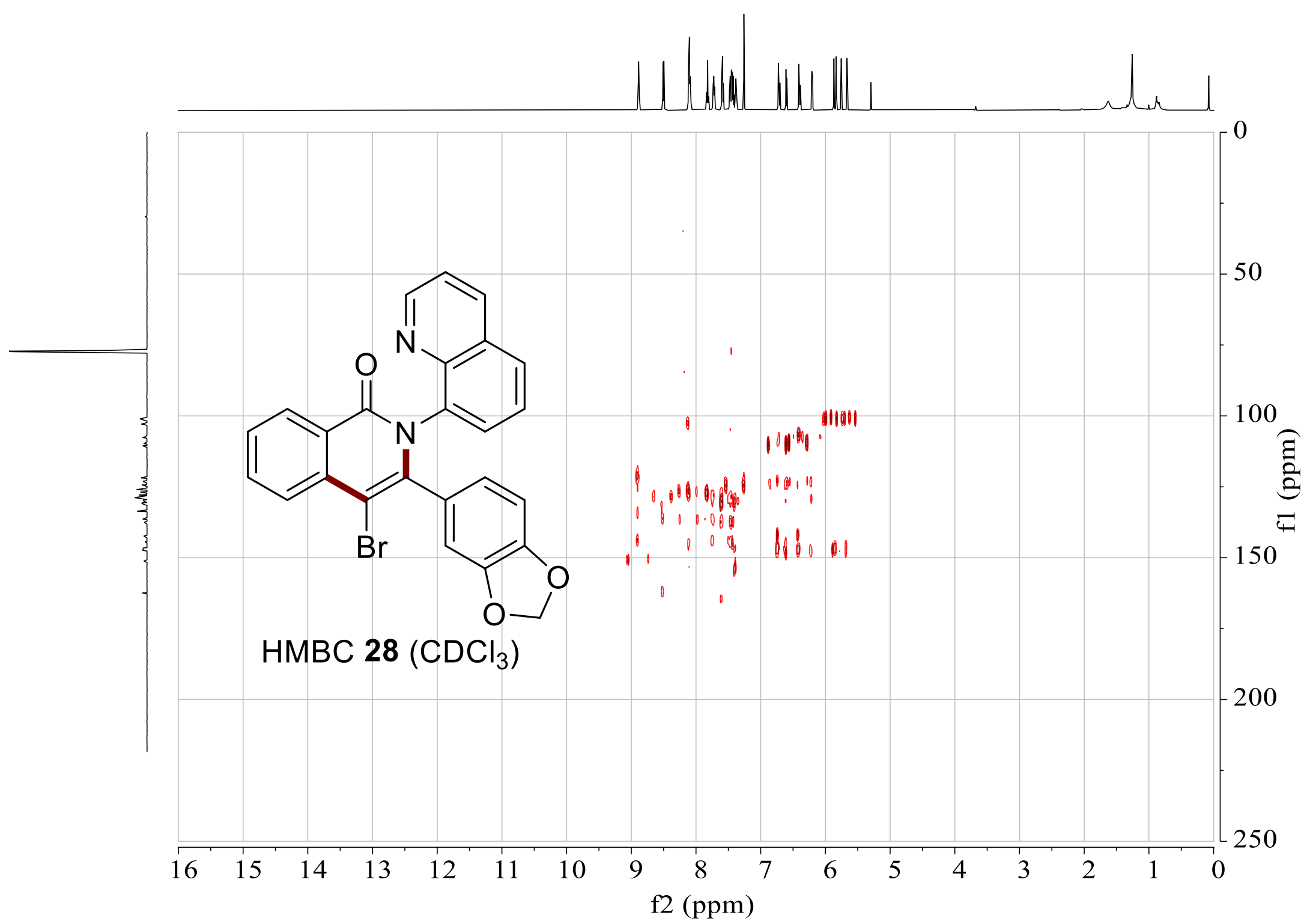
N
O
N
Br
O
O
HMBC 28 (CDCl$_3$)
f2 (ppm)
f1 (ppm)

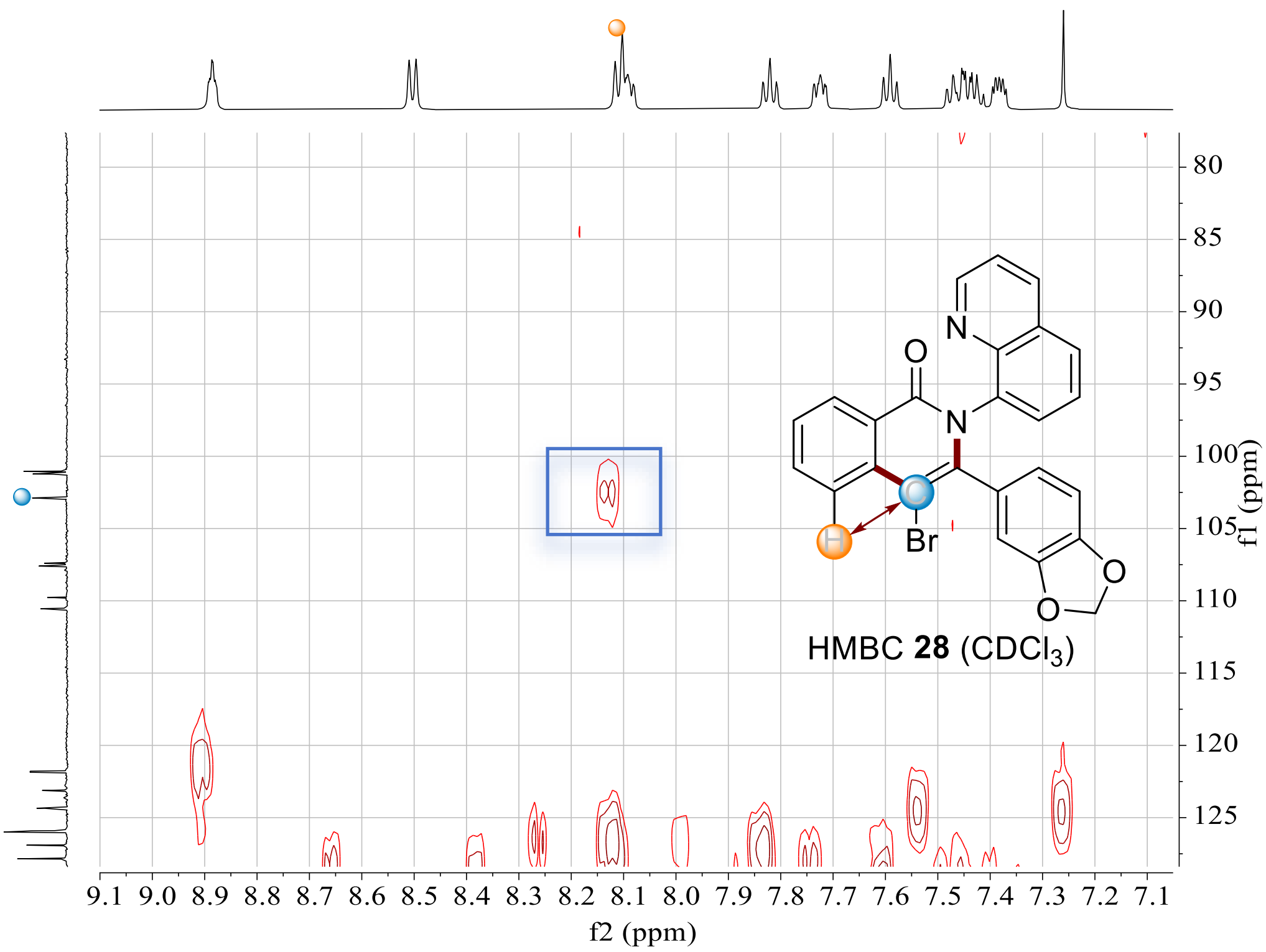
HMBC 28 (CDCl3)
f2 (ppm)
f1 (ppm)

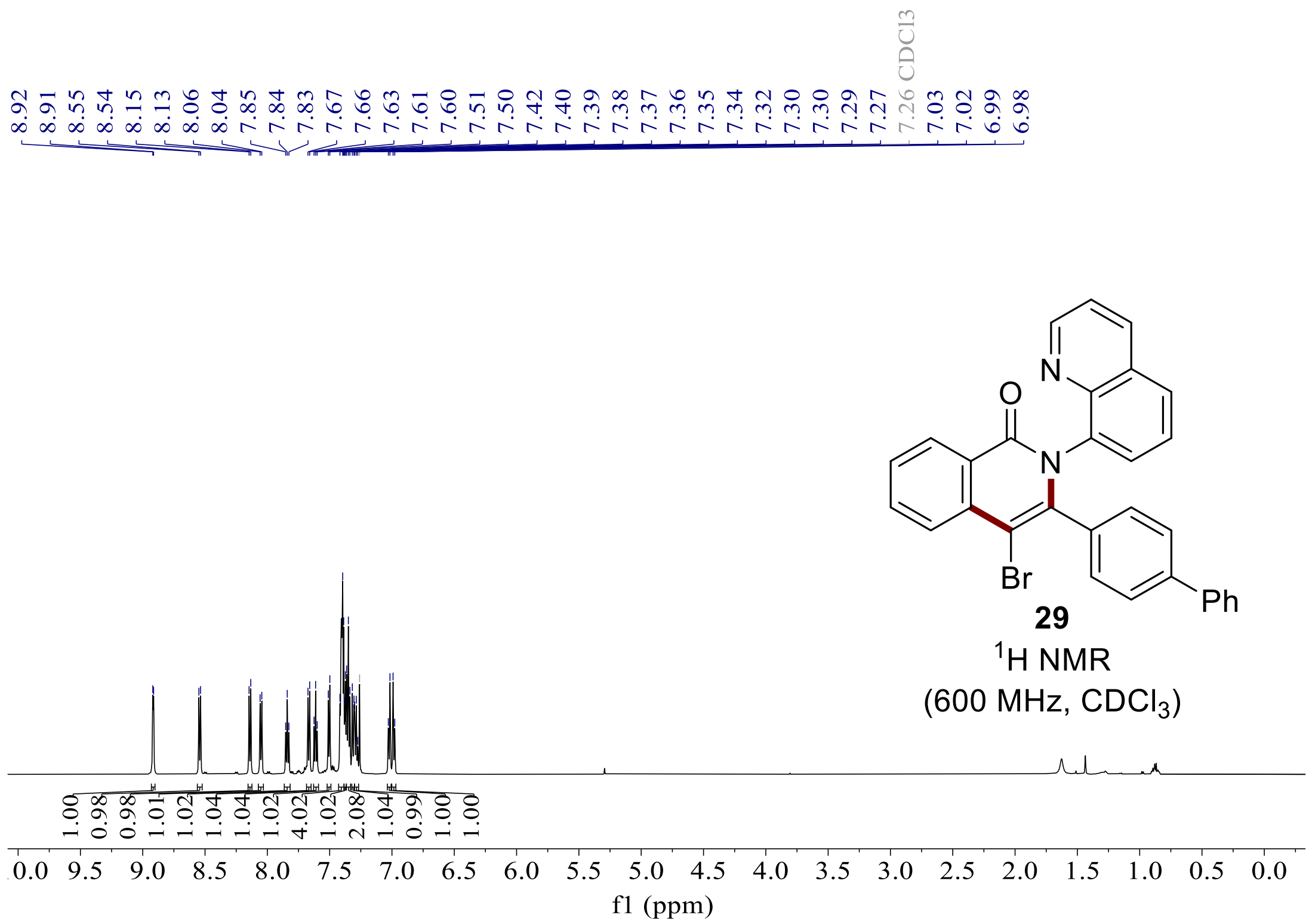
29
1H NMR
(600 MHz, CDCl3)
f1 (ppm)

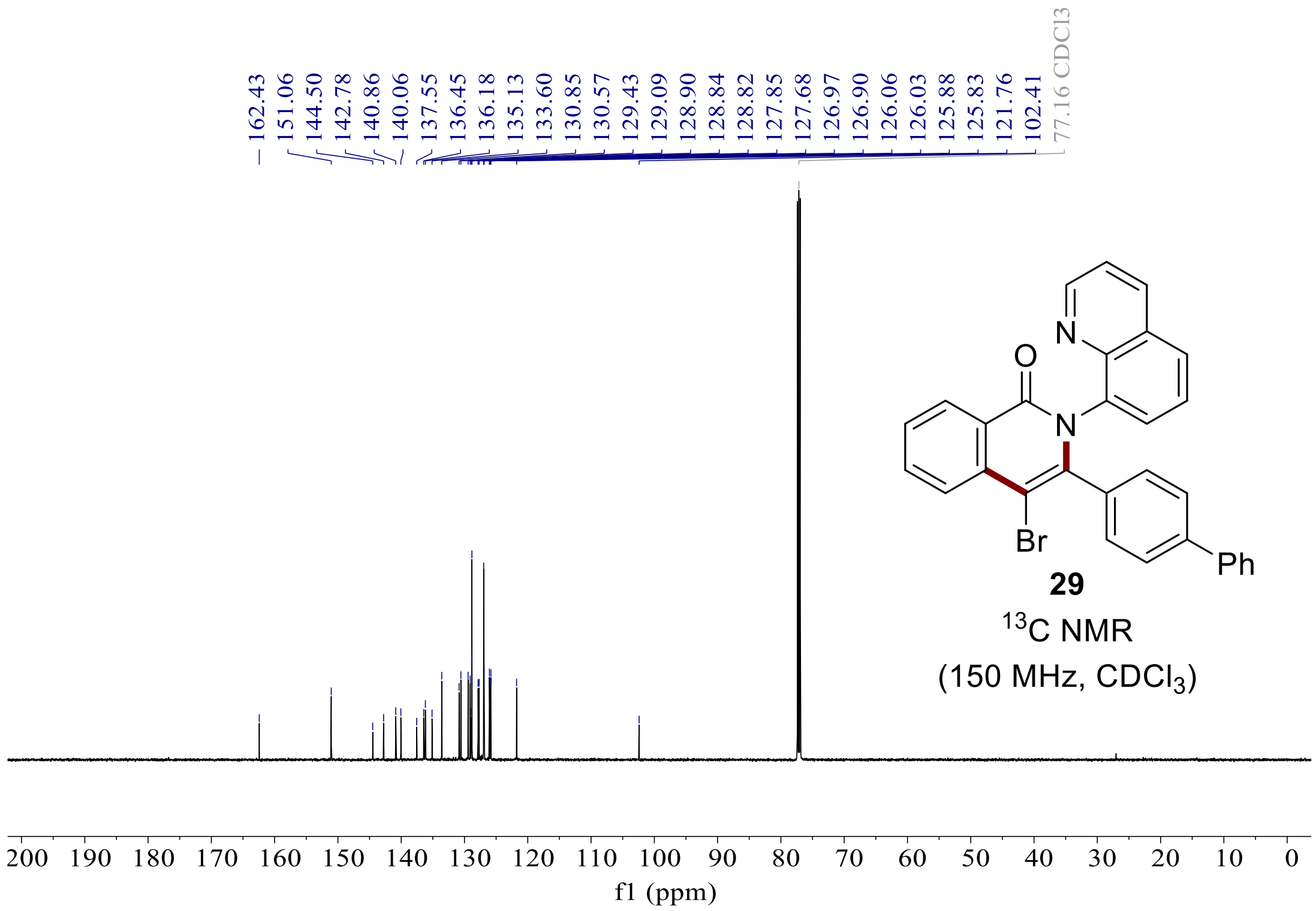

162.43
151.06
144.50
142.78
140.86
140.06
137.55
136.45
136.18
135.13
133.60
130.85
130.57
129.43
129.09
128.90
128.84
128.82
127.85
127.68
126.97
126.90
126.06
126.03
125.88
125.83
121.76
102.41
77.16 CDCl3
O
N
N
Br
Ph
29
^{13}C NMR
(150 MHz, CDCl$_3$)
200 190 180 170 160 150 140 130 120 110 100 90 80 70 60 50 40 30 20 10 0
f1 (ppm)

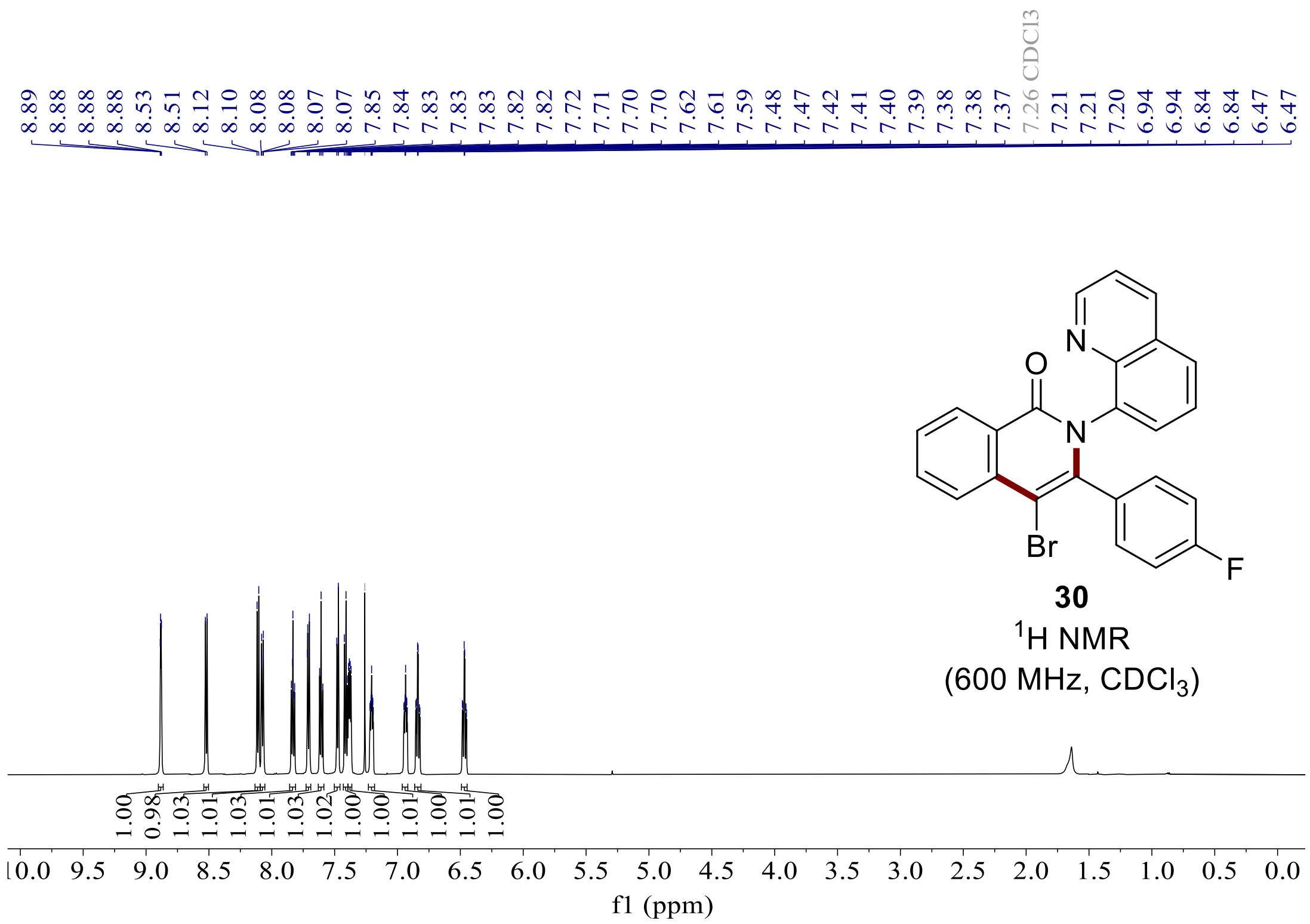

8.89
8.88
8.88
8.88
8.53
8.51
8.12
8.10
8.08
8.08
8.07
8.07
7.85
7.84
7.83
7.83
7.82
7.82
7.72
7.71
7.70
7.70
7.62
7.61
7.59
7.48
7.47
7.42
7.41
7.40
7.39
7.38
7.38
7.37
7.26 CDCl3
7.21
7.21
7.20
6.94
6.94
6.84
6.84
6.47
6.47
O
N
N
Br
F
30
^{1}H NMR
(600 MHz, CDCl$_3$)
1.00
0.98
1.03
1.01
1.03
1.01
1.03
1.02
1.00
1.00
1.01
1.00
1.01
1.00
10.0 9.5 9.0 8.5 8.0 7.5 7.0 6.5 6.0 5.5 5.0 4.5 4.0 3.5 3.0 2.5 2.0 1.5 1.0 0.5 0.0
f1 (ppm)

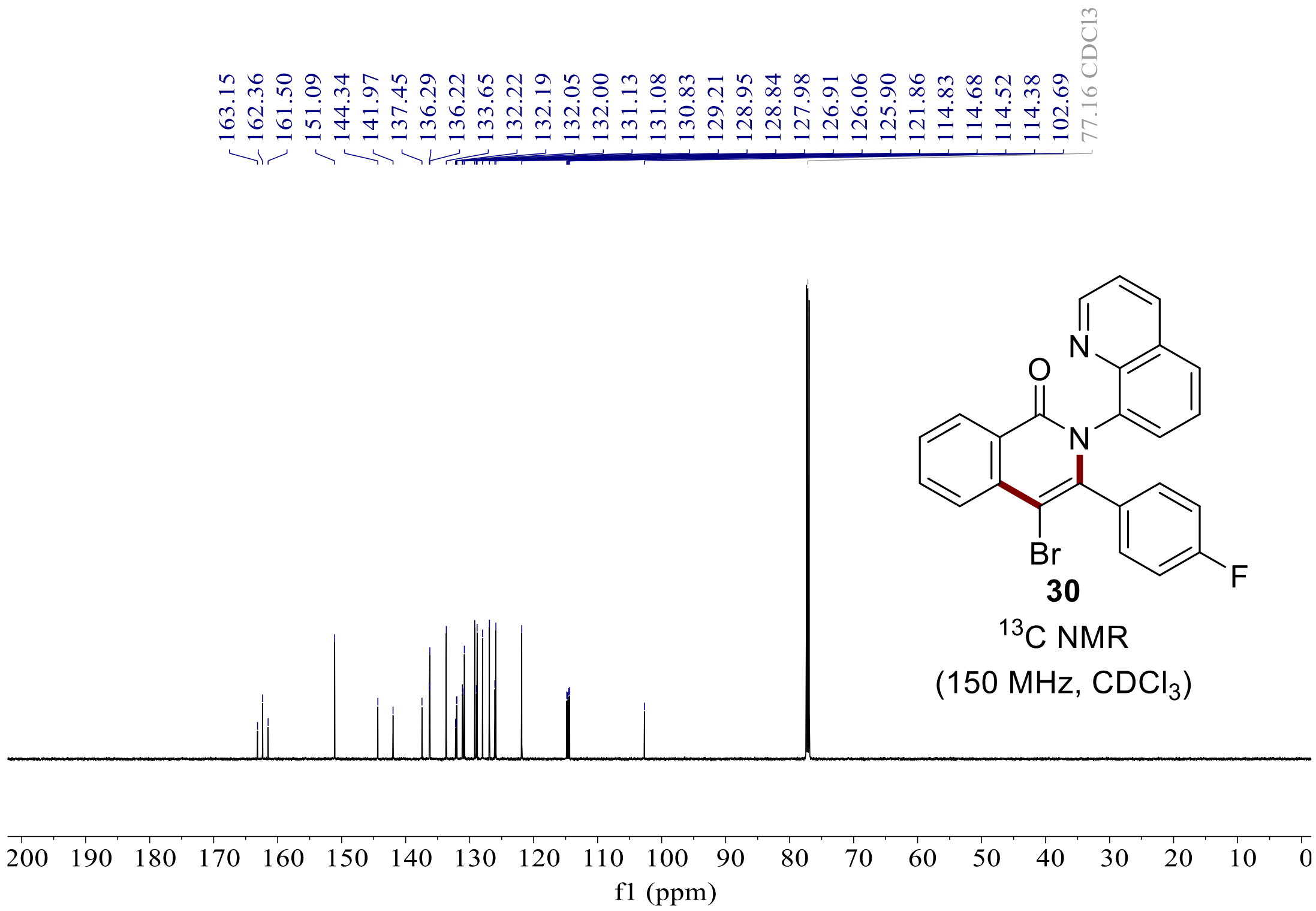
163.15
162.36
161.50
151.09
144.34
141.97
137.45
136.29
136.22
133.65
132.22
132.19
132.05
132.00
131.13
131.08
130.83
129.21
128.95
128.84
127.98
126.91
126.06
125.90
121.86
114.83
114.68
114.52
114.38
102.69
77.16 CDCl3
O
N
N
Br
F
30
13C NMR
(150 MHz, CDCl3)
200 190 180 170 160 150 140 130 120 110 100 90 80 70 60 50 40 30 20 10 0
f1 (ppm)

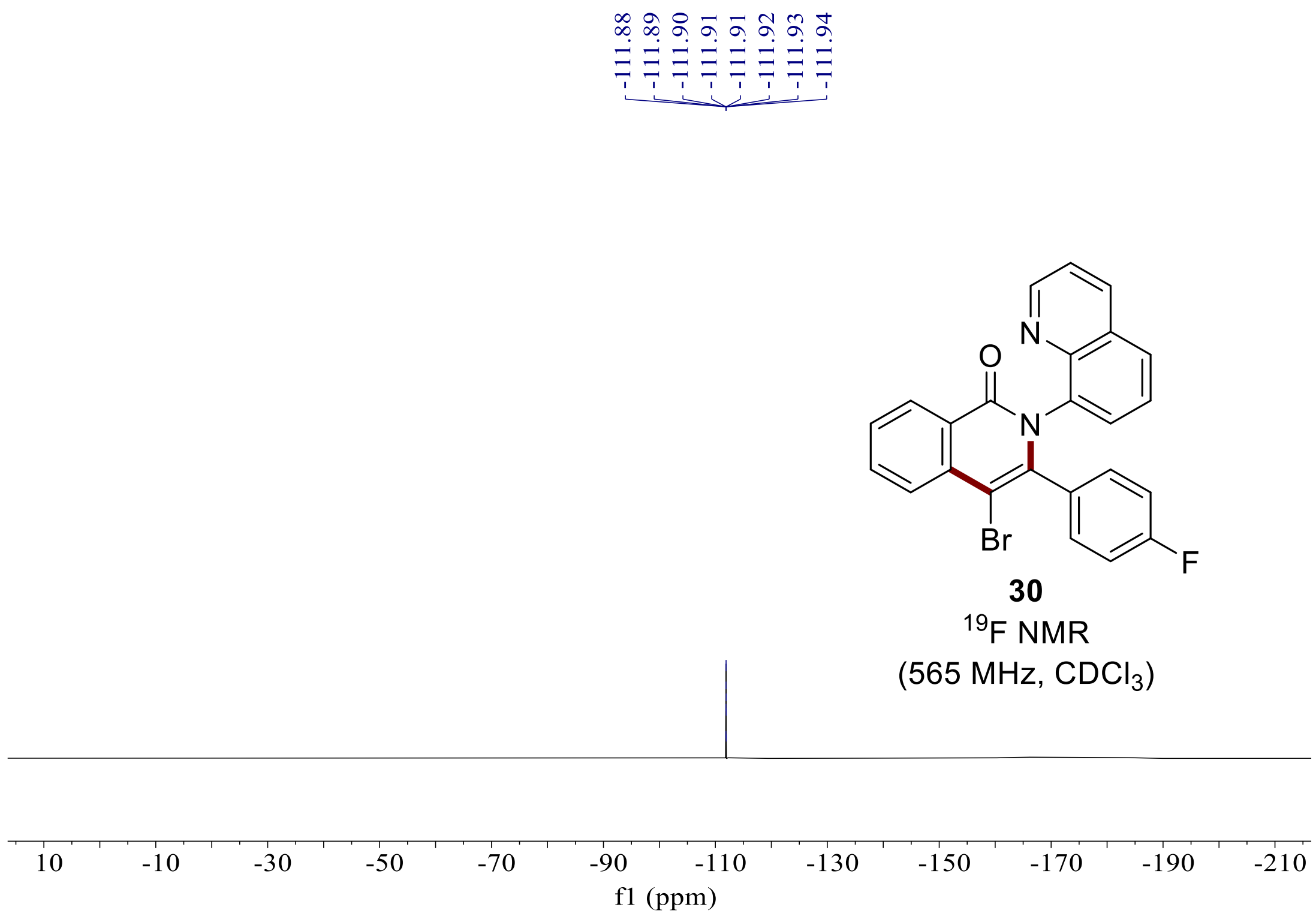
-111.88
-111.89
-111.90
-111.91
-111.91
-111.92
-111.93
-111.94
O
N
N
Br
F
30
19F NMR
(565 MHz, CDCl3)
10 -10 -30 -50 -70 -90 -110 -130 -150 -170 -190 -210
f1 (ppm)

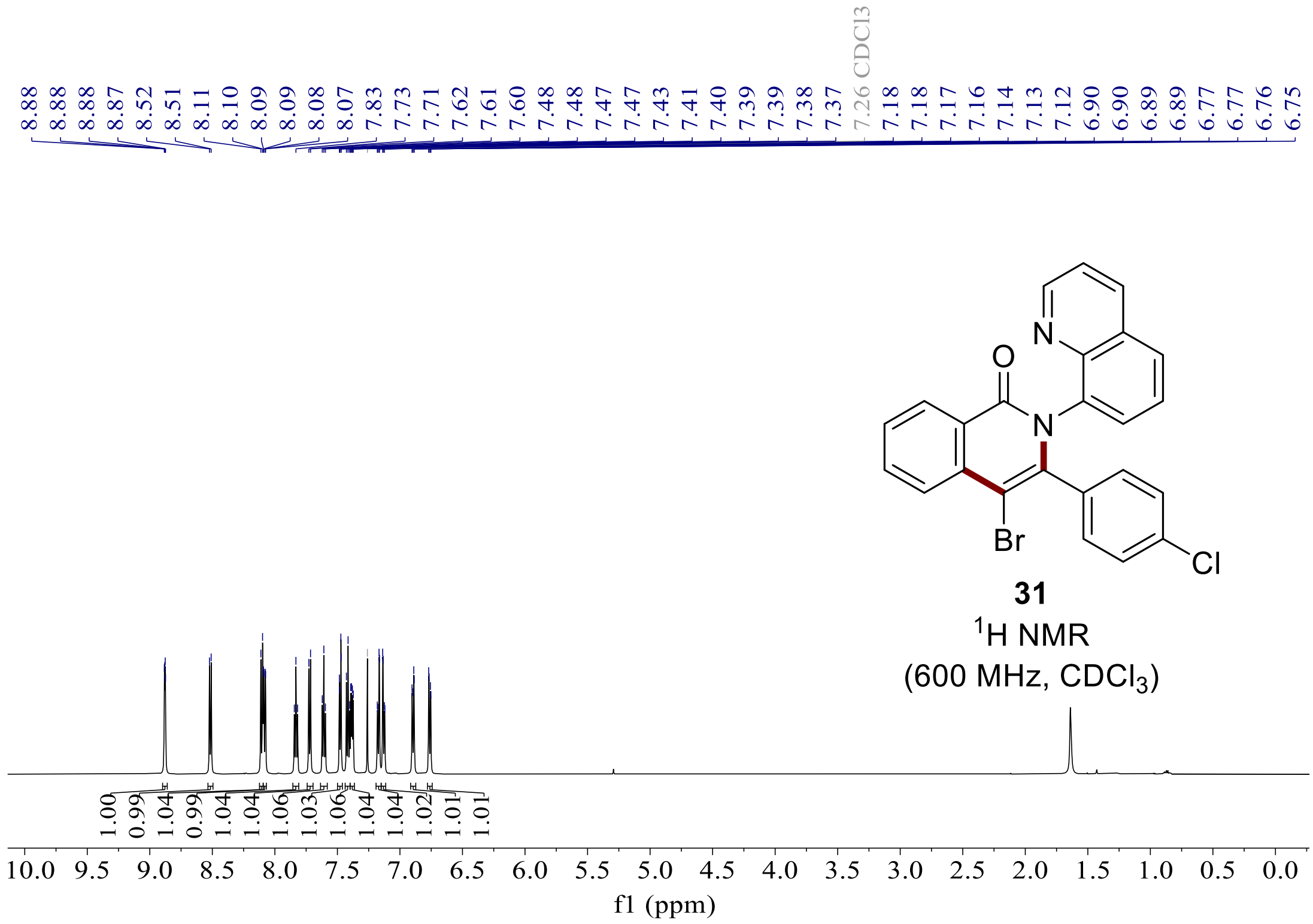

8.88
8.88
8.88
8.87
8.52
8.51
8.11
8.10
8.09
8.09
8.08
8.07
7.83
7.73
7.71
7.62
7.61
7.60
7.48
7.48
7.47
7.47
7.43
7.41
7.40
7.39
7.39
7.38
7.37
7.26 CDCl3
7.18
7.18
7.17
7.16
7.14
7.13
7.12
6.90
6.90
6.89
6.89
6.77
6.77
6.76
6.75
O
N
N
Br
Cl
31
^{1}H NMR
(600 MHz, $CDCl_3$)
1.00
0.99
1.04
0.99
1.04
1.04
1.06
1.03
1.06
1.04
1.04
1.02
1.01
1.01
10.0 9.5 9.0 8.5 8.0 7.5 7.0 6.5 6.0 5.5 5.0 4.5 4.0 3.5 3.0 2.5 2.0 1.5 1.0 0.5 0.0
f1 (ppm)

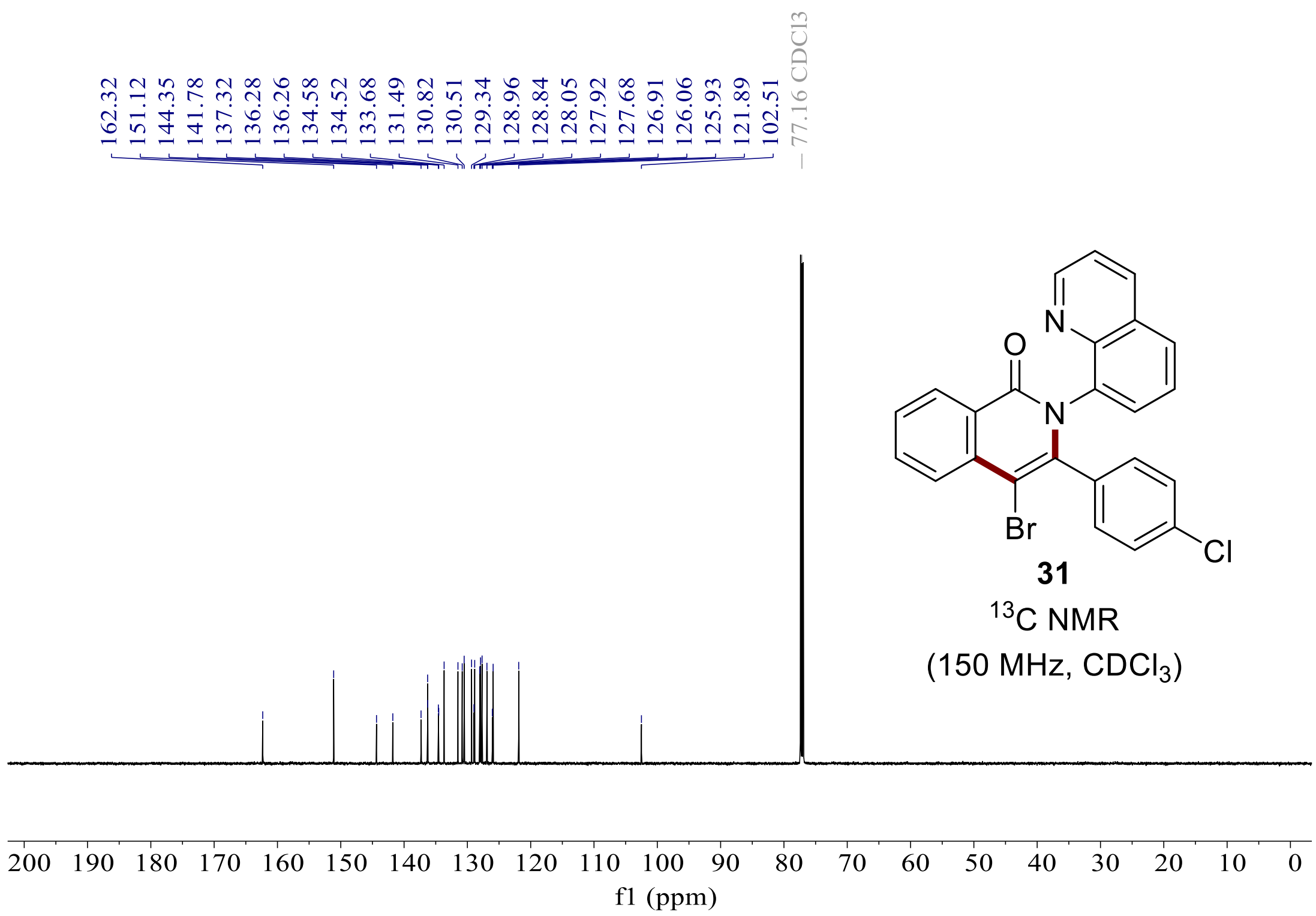

162.32
151.12
144.35
141.78
137.32
136.28
136.26
134.58
134.52
133.68
131.49
130.82
130.51
129.34
128.96
128.84
128.05
127.92
127.68
126.91
126.06
125.93
121.89
102.51
77.16 CDCl3
O
N
N
Br
Cl
31
^{13}C NMR
(150 MHz, $CDCl_3$)
200 190 180 170 160 150 140 130 120 110 100 90 80 70 60 50 40 30 20 10 0
f1 (ppm)

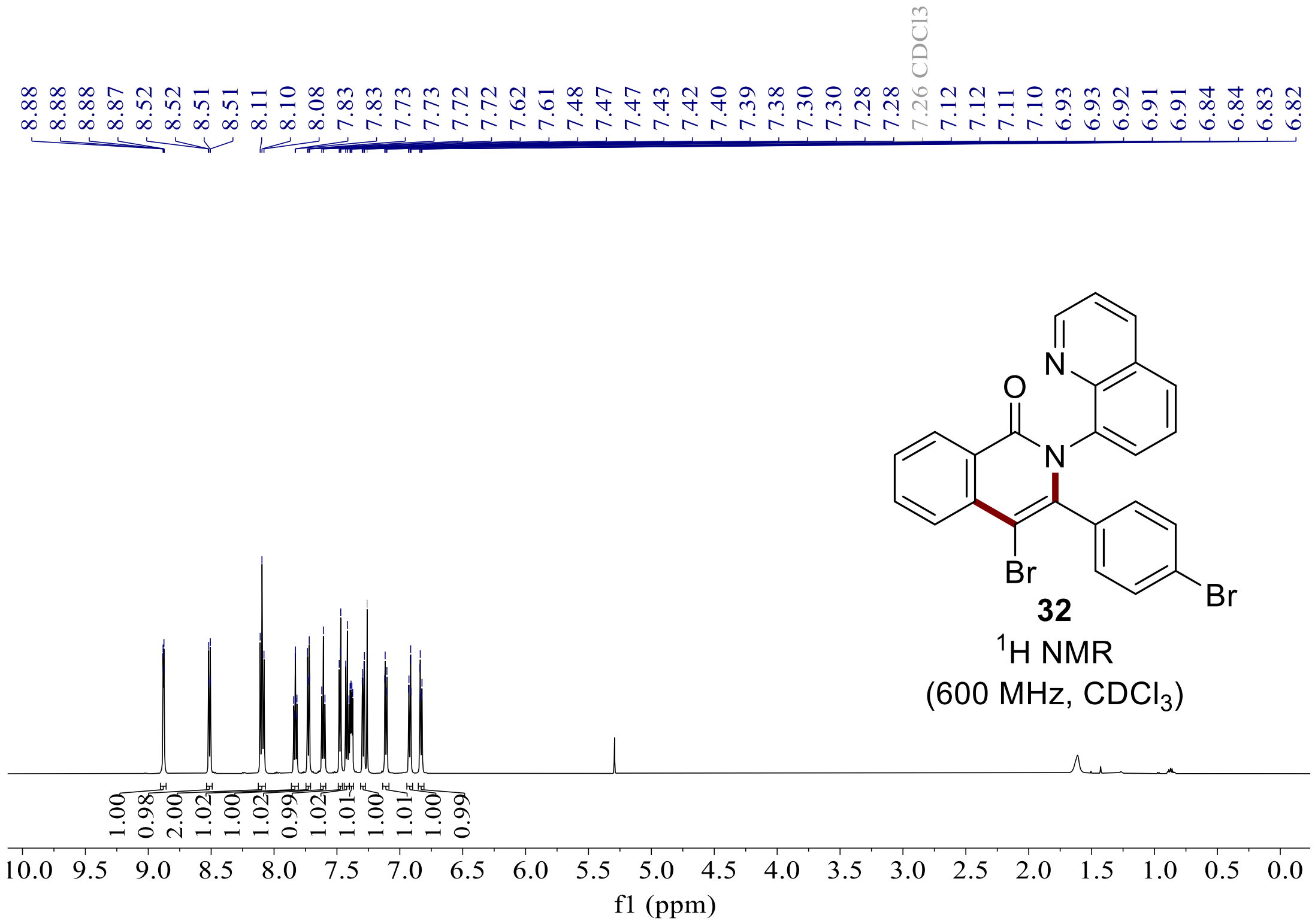

8.88 8.88 8.88 8.87 8.52 8.52 8.51 8.51 8.11 8.10 8.08 7.83 7.83 7.73 7.73 7.72 7.72 7.62 7.61 7.48 7.47 7.47 7.43 7.42 7.40 7.39 7.38 7.30 7.30 7.28 7.28 7.26 CDCl3 7.12 7.12 7.11 7.10 6.93 6.93 6.92 6.91 6.91 6.84 6.84 6.83 6.82
1.00 0.98 2.00 1.02 1.00 1.02 0.99 1.02 1.01 1.00 1.01 1.00 0.99
10.0 9.5 9.0 8.5 8.0 7.5 7.0 6.5 6.0 5.5 5.0 4.5 4.0 3.5 3.0 2.5 2.0 1.5 1.0 0.5 0.0
f1 (ppm)
O
N
N
Br
Br
32
1H NMR
(600 MHz, CDCl3)

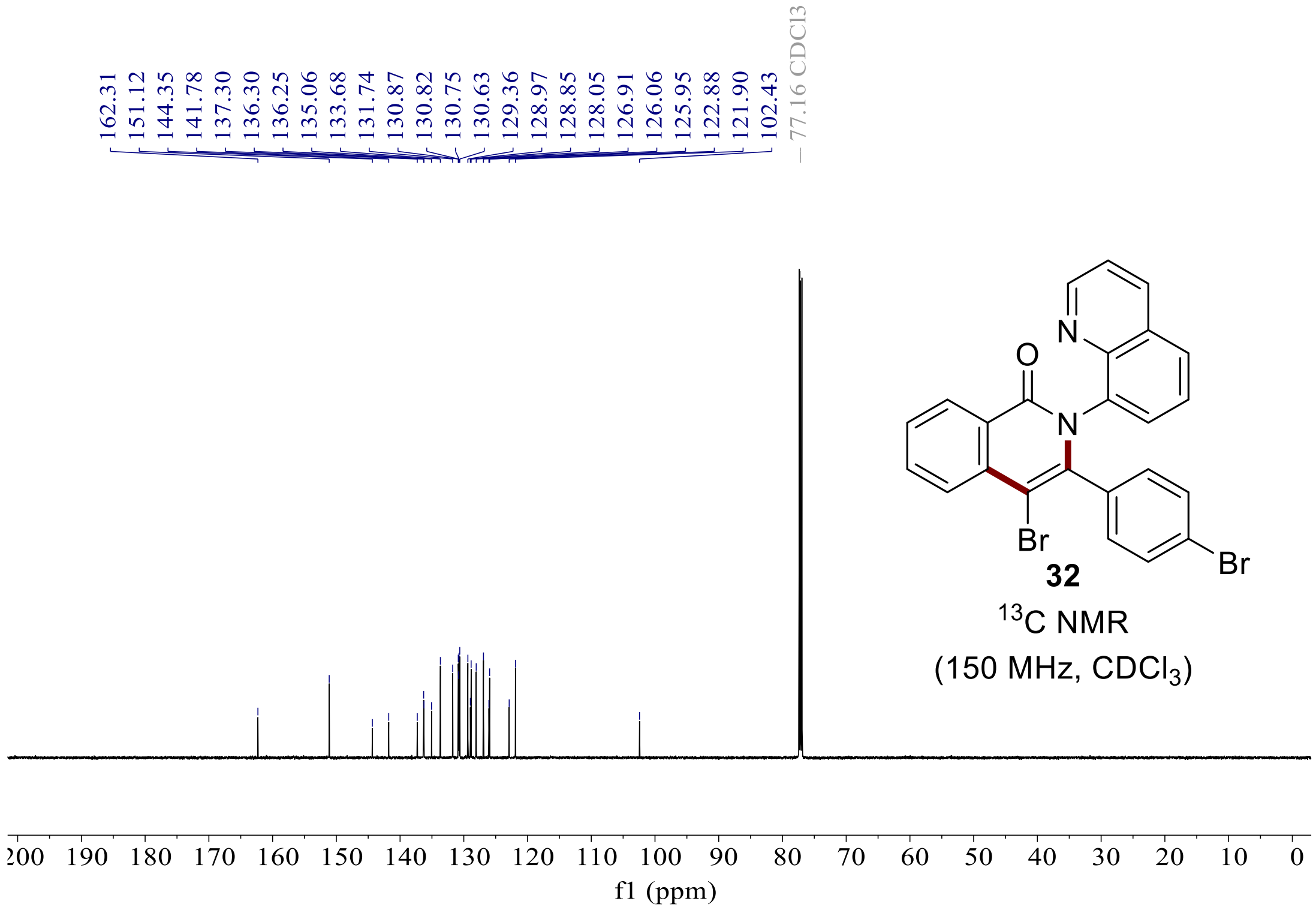

162.31 151.12 144.35 141.78 137.30 136.30 136.25 135.06 133.68 131.74 130.87 130.82 130.75 130.63 129.36 128.97 128.85 128.05 126.91 126.06 125.95 122.88 121.90 102.43
77.16 CDCl3
200 190 180 170 160 150 140 130 120 110 100 90 80 70 60 50 40 30 20 10 0
f1 (ppm)
O
N
N
Br
Br
32
13C NMR
(150 MHz, CDCl3)

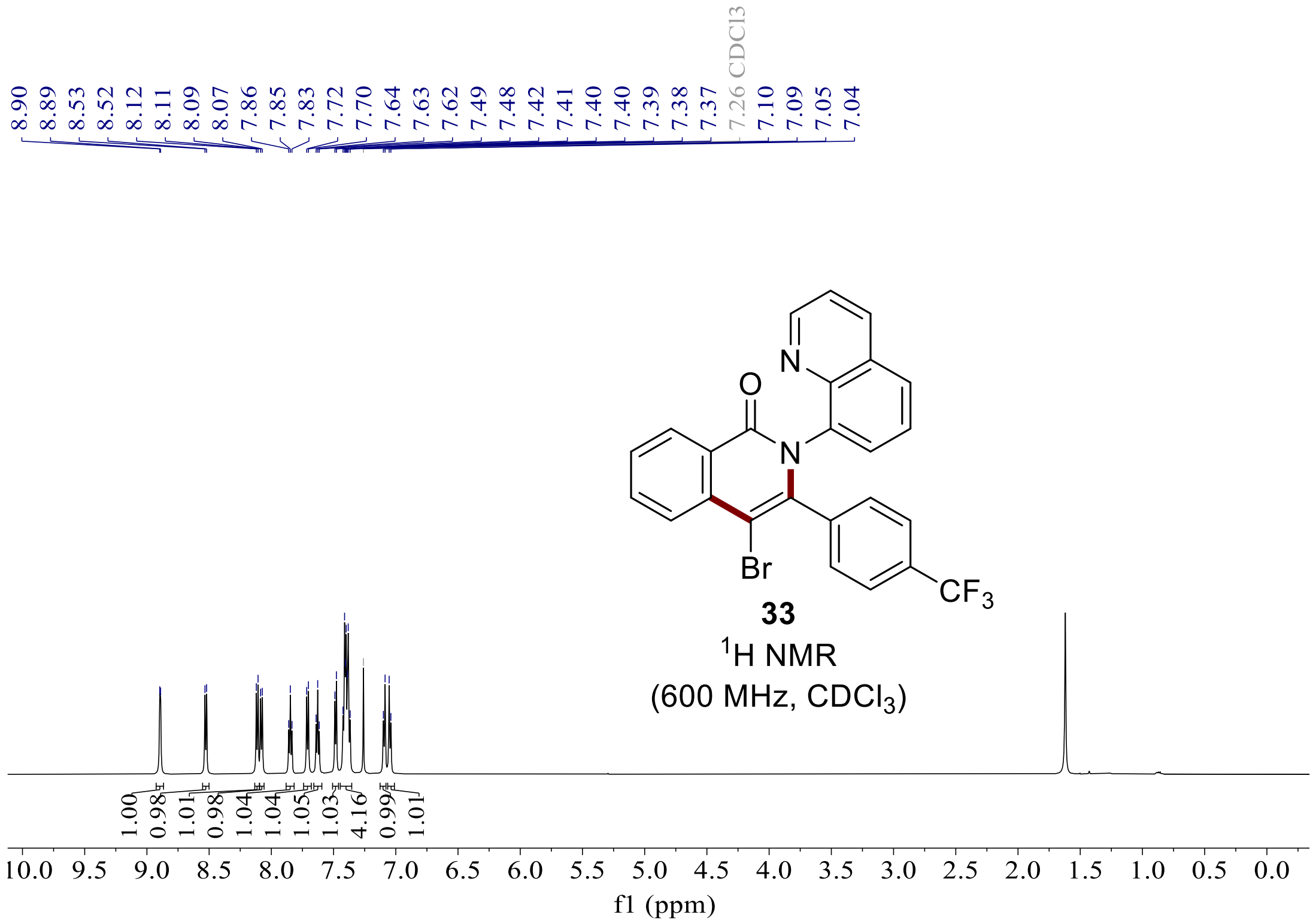
8.90
8.89
8.53
8.52
8.12
8.11
8.09
8.07
7.86
7.85
7.83
7.72
7.70
7.64
7.63
7.62
7.49
7.48
7.42
7.41
7.40
7.40
7.39
7.38
7.37
7.26 CDCl3
7.10
7.09
7.05
7.04
O
N
N
Br
CF3
33
1H NMR
(600 MHz, CDCl3)
1.00
0.98
1.01
0.98
1.04
1.04
1.05
1.03
4.16
0.99
1.01
10.0 9.5 9.0 8.5 8.0 7.5 7.0 6.5 6.0 5.5 5.0 4.5 4.0 3.5 3.0 2.5 2.0 1.5 1.0 0.5 0.0
f1 (ppm)

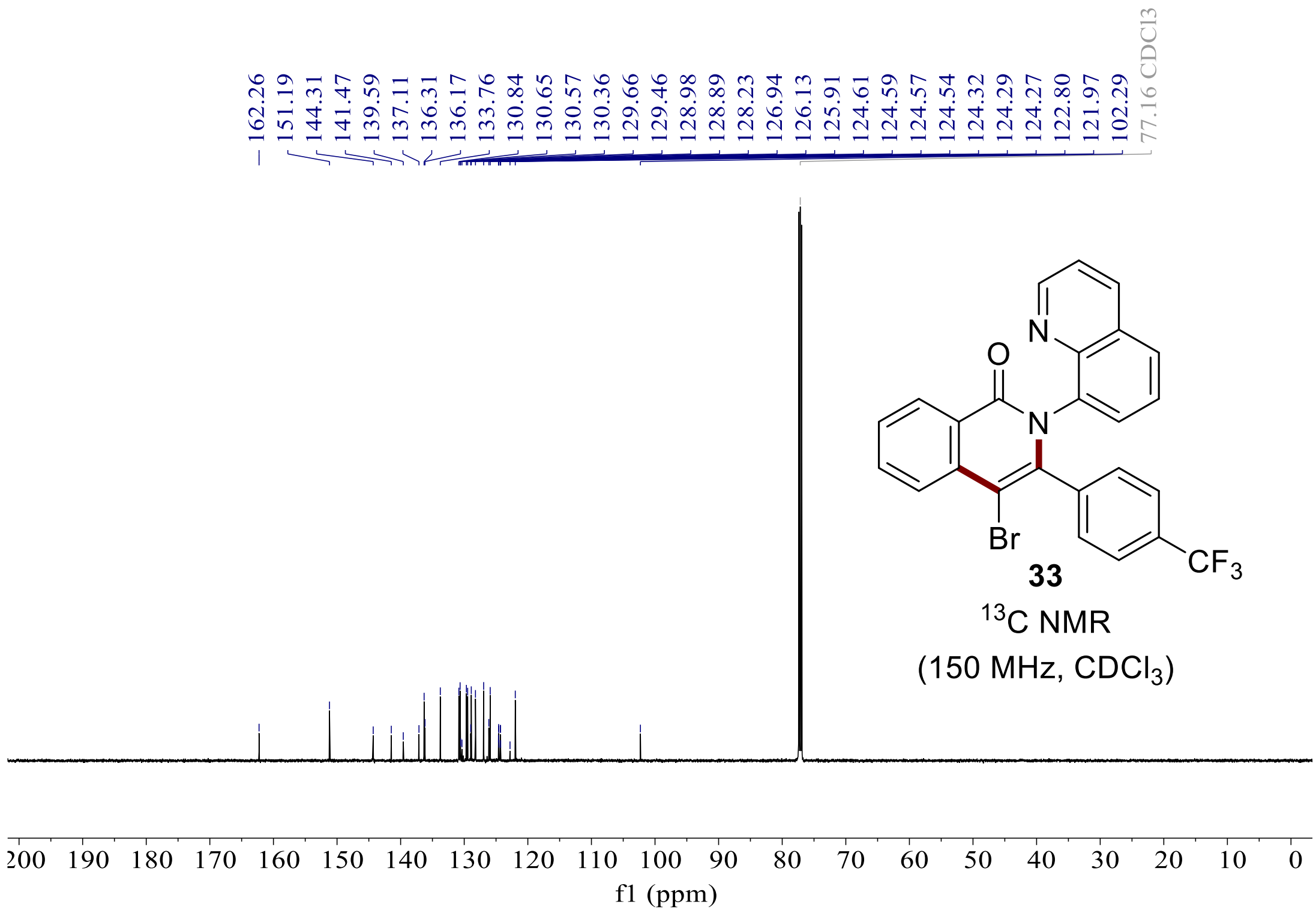
162.26
151.19
144.31
141.47
139.59
137.11
136.31
136.17
133.76
130.84
130.65
130.57
130.36
129.66
129.46
128.98
128.89
128.23
126.94
126.13
125.91
124.61
124.59
124.57
124.54
124.32
124.29
124.27
122.80
121.97
102.29
77.16 CDCl3
O
N
N
Br
CF3
33
13C NMR
(150 MHz, CDCl3)
200 190 180 170 160 150 140 130 120 110 100 90 80 70 60 50 40 30 20 10 0
f1 (ppm)

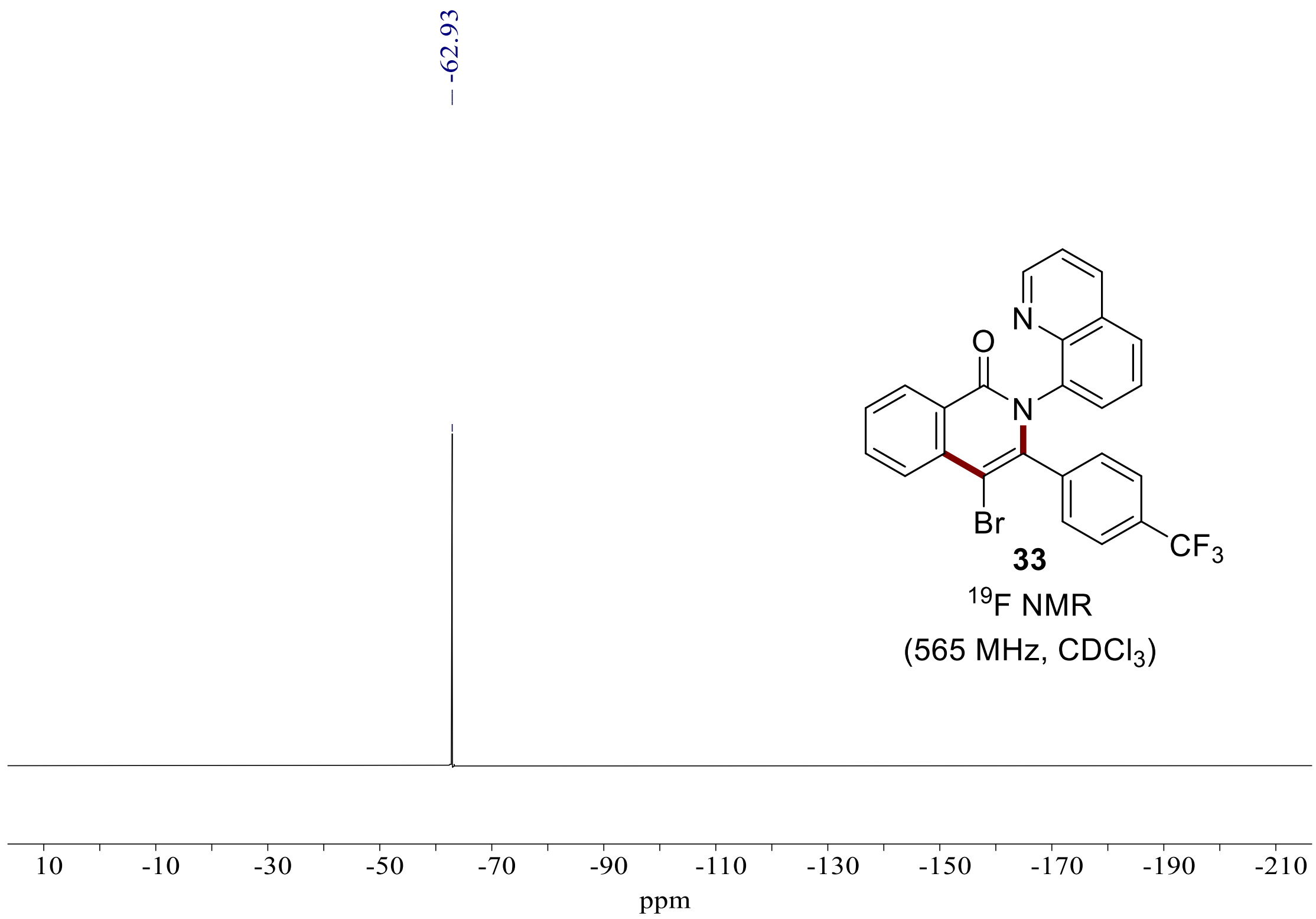
-62.93
O
N
N
Br
CF3
33
19F NMR
(565 MHz, CDCl3)
10 -10 -30 -50 -70 -90 -110 -130 -150 -170 -190 -210
ppm

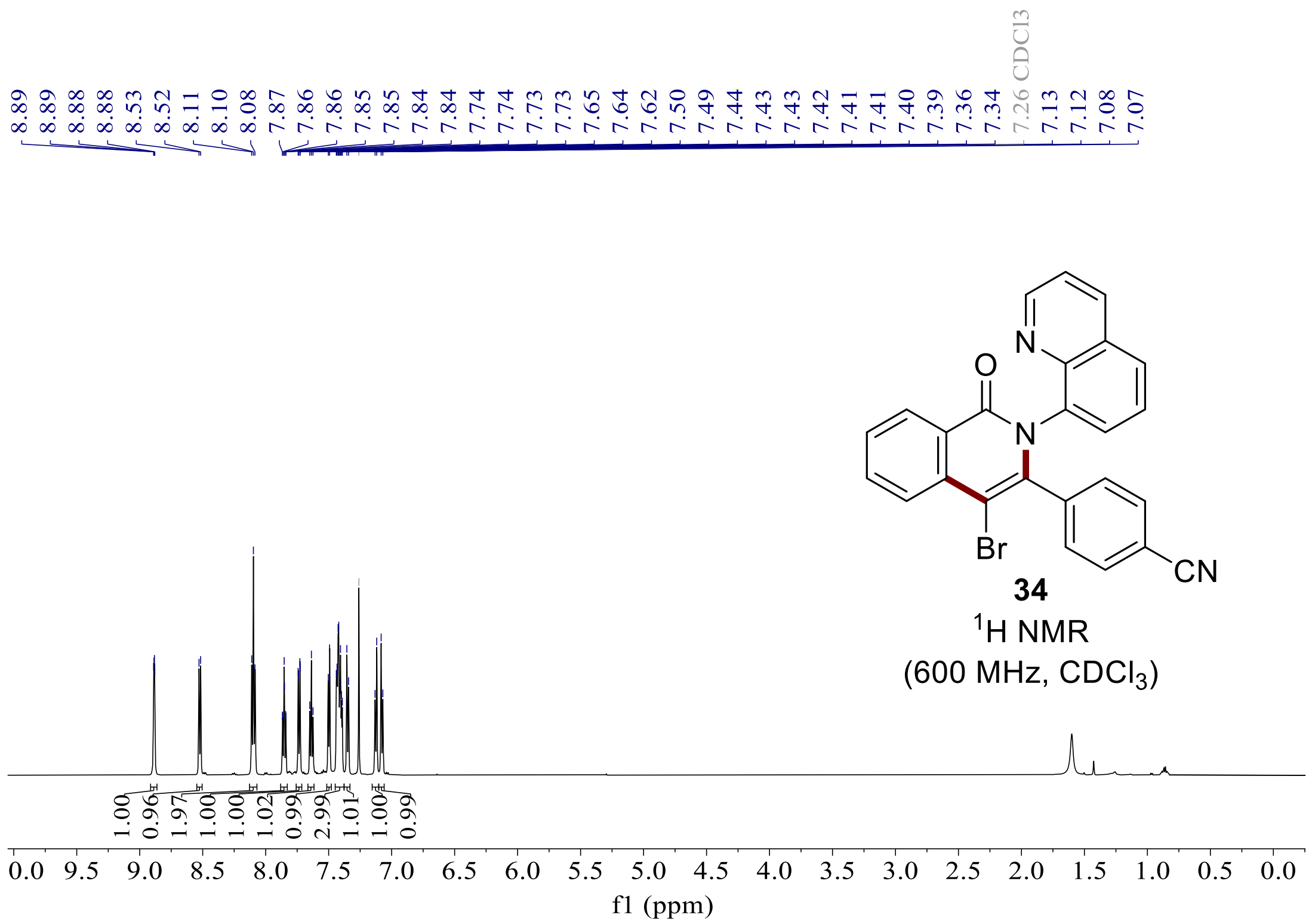
8.89 8.89 8.88 8.88 8.53 8.52 8.11 8.10 8.08 7.87 7.86 7.86 7.85 7.85 7.84 7.84 7.74 7.74 7.73 7.73 7.65 7.64 7.62 7.50 7.49 7.44 7.43 7.43 7.42 7.41 7.41 7.40 7.39 7.36 7.34 7.26 CDCl3 7.13 7.12 7.08 7.07
O
N
N
Br
CN
34
1H NMR
(600 MHz, CDCl3)
1.00 0.96 1.97 1.00 1.00 1.02 0.99 2.99 1.01 1.00 0.99
0.0 9.5 9.0 8.5 8.0 7.5 7.0 6.5 6.0 5.5 5.0 4.5 4.0 3.5 3.0 2.5 2.0 1.5 1.0 0.5 0.0
f1 (ppm)

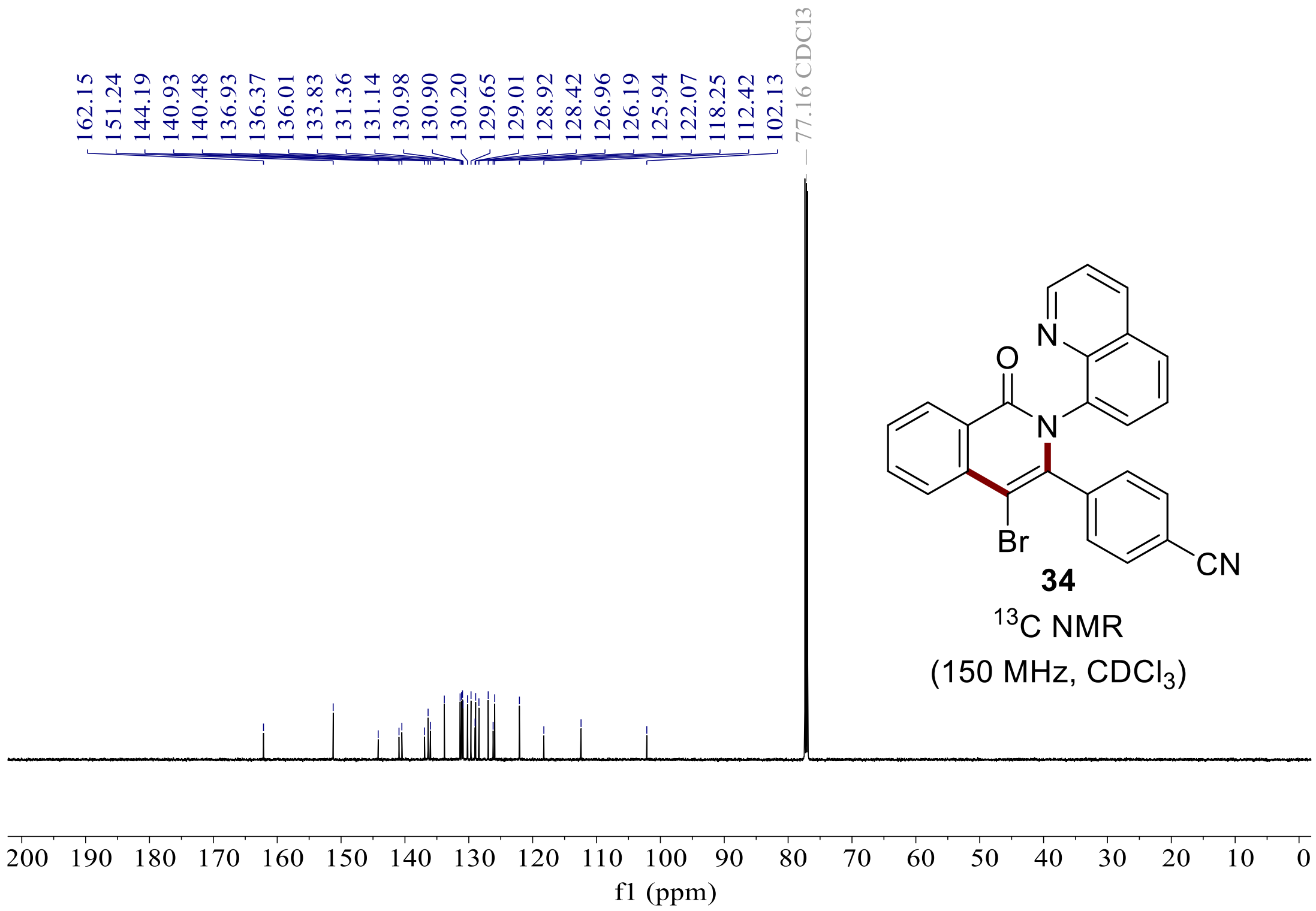
162.15 151.24 144.19 140.93 140.48 136.93 136.37 136.01 133.83 131.36 131.14 130.98 130.90 130.20 129.65 129.01 128.92 128.42 126.96 126.19 125.94 122.07 118.25 112.42 102.13
77.16 CDCl3
O
N
N
Br
CN
34
^{13}C NMR
(150 MHz, $CDCl_3$)
200 190 180 170 160 150 140 130 120 110 100 90 80 70 60 50 40 30 20 10 0
f1 (ppm)

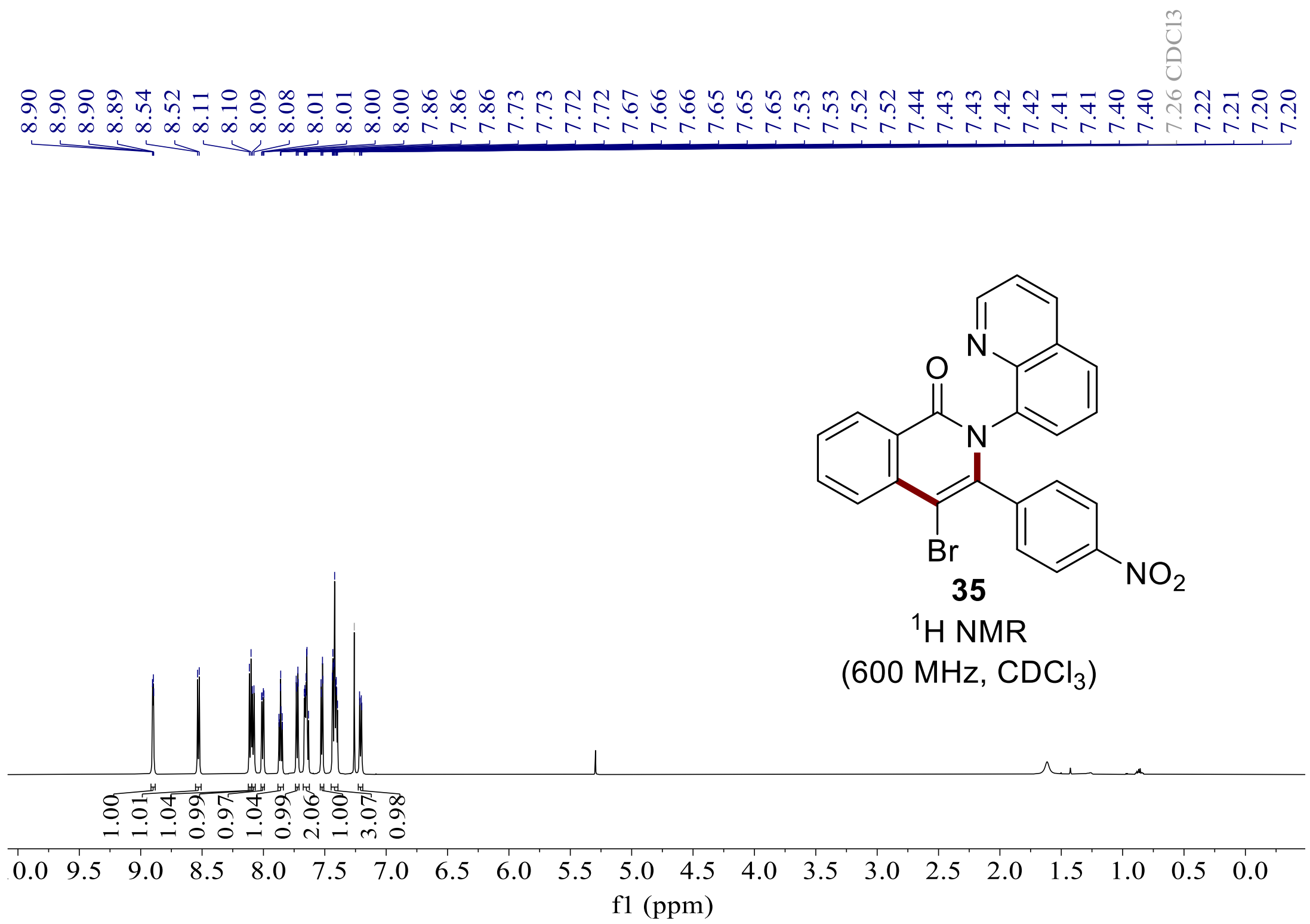
8.90 8.90 8.90 8.89 8.54 8.52 8.11 8.10 8.09 8.08 8.01 8.01 8.00 8.00 7.86 7.86 7.86 7.73 7.73 7.72 7.72 7.67 7.66 7.65 7.65 7.65 7.53 7.53 7.52 7.52 7.44 7.43 7.43 7.42 7.42 7.41 7.41 7.40 7.40 7.26 CDCl3 7.22 7.21 7.20 7.20
O
N
N
Br
NO_2
35
^{1}H NMR
(600 MHz, $CDCl_3$)
1.00 1.01 1.04 0.99 0.97 1.04 0.99 2.06 1.00 3.07 0.98
0.0 9.5 9.0 8.5 8.0 7.5 7.0 6.5 6.0 5.5 5.0 4.5 4.0 3.5 3.0 2.5 2.0 1.5 1.0 0.5 0.0
f1 (ppm)

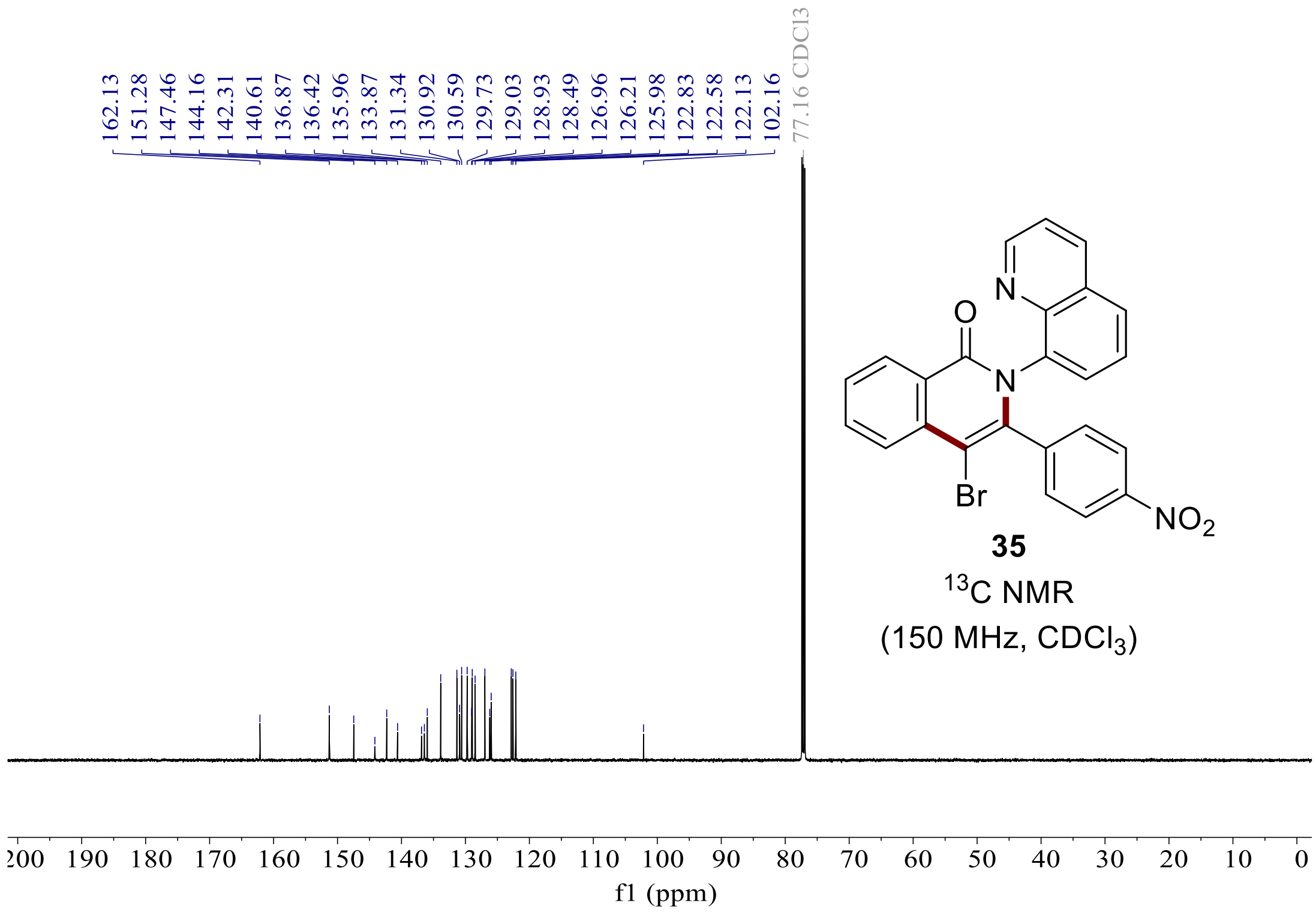
162.13
151.28
147.46
144.16
142.31
140.61
136.87
136.42
135.96
133.87
131.34
130.92
130.59
129.73
129.03
128.93
128.49
126.96
126.21
125.98
122.83
122.58
122.13
102.16
77.16 CDCl3
O
N
N
Br
NO_2
35
^{13}C NMR
(150 MHz, $CDCl_3$)
200 190 180 170 160 150 140 130 120 110 100 90 80 70 60 50 40 30 20 10 0
f1 (ppm)

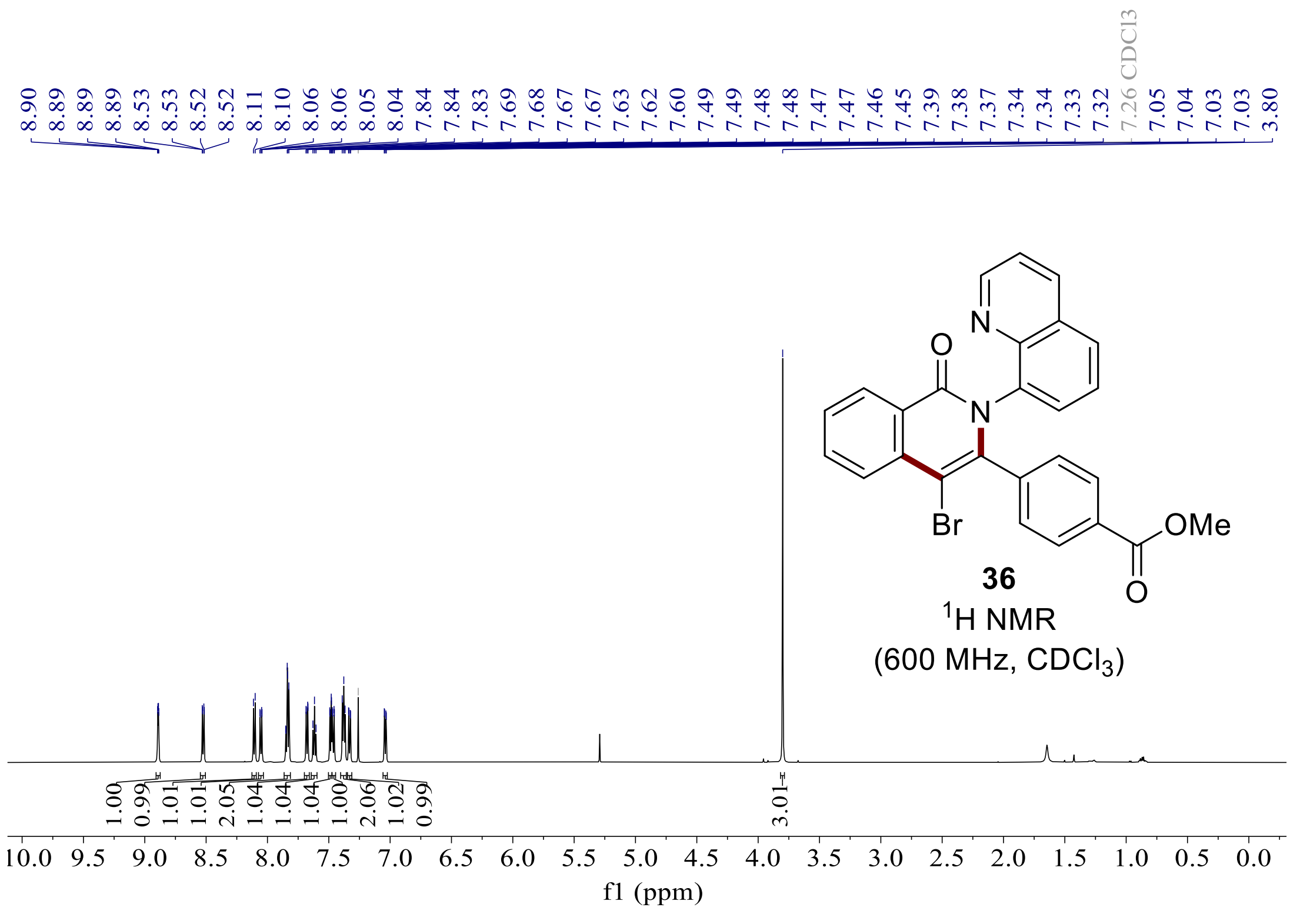
8.90
8.89
8.89
8.89
8.53
8.53
8.52
8.52
8.11
8.10
8.06
8.06
8.05
8.04
7.84
7.84
7.83
7.69
7.68
7.67
7.67
7.63
7.62
7.60
7.49
7.49
7.48
7.48
7.47
7.47
7.46
7.45
7.39
7.38
7.37
7.34
7.34
7.33
7.32
7.26 CDCl3
7.05
7.04
7.03
7.03
3.80
O
N
N
Br
OMe
O
36
^{1}H NMR
(600 MHz, $CDCl_3$)
1.00
0.99
1.01
1.01
2.05
1.04
1.04
1.04
1.00
2.06
1.02
0.99
3.01
10.0 9.5 9.0 8.5 8.0 7.5 7.0 6.5 6.0 5.5 5.0 4.5 4.0 3.5 3.0 2.5 2.0 1.5 1.0 0.5 0.0
f1 (ppm)

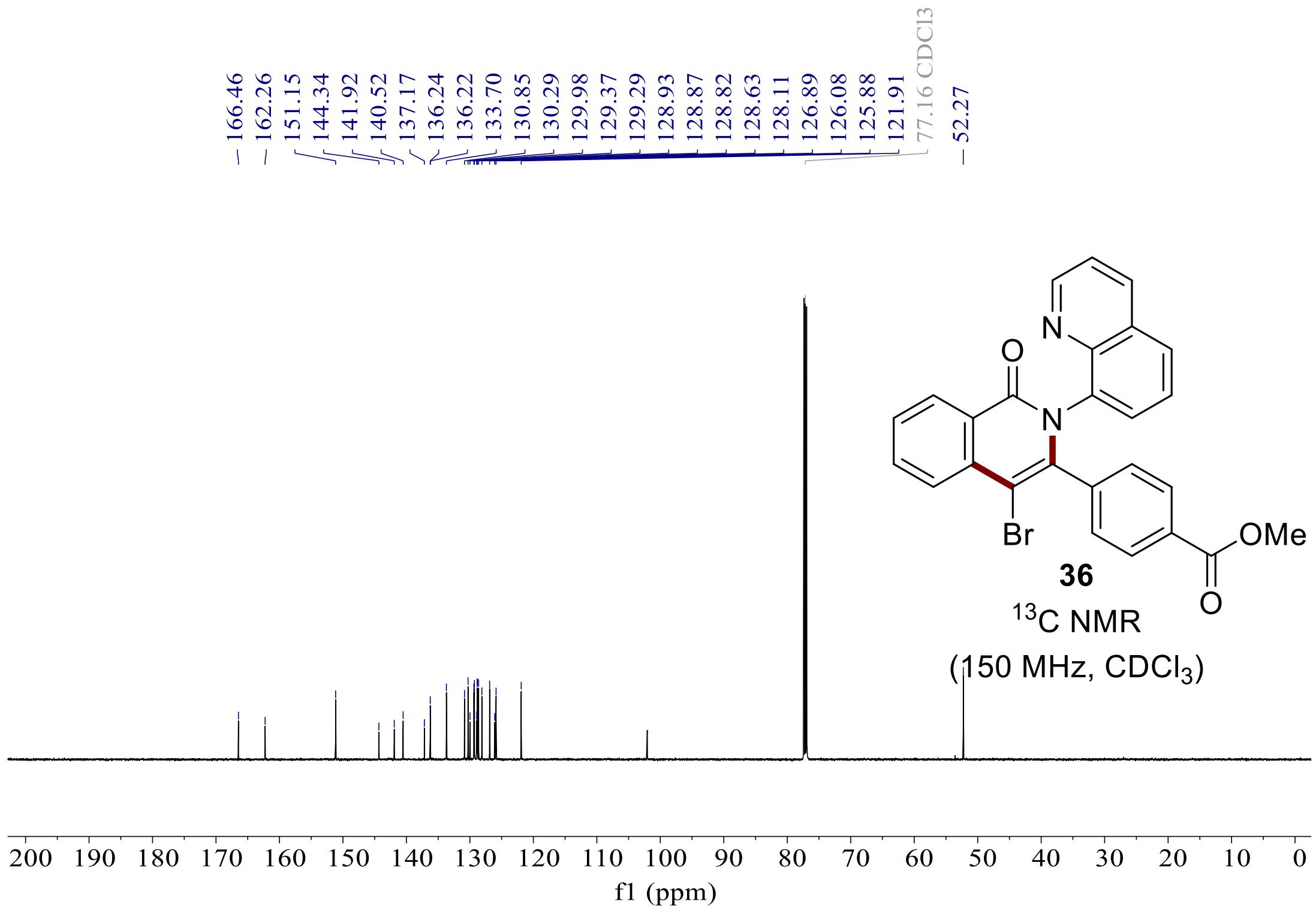

166.46
162.26
151.15
144.34
141.92
140.52
137.17
136.24
136.22
133.70
130.85
130.29
129.98
129.37
129.29
128.93
128.87
128.82
128.63
128.11
126.89
126.08
125.88
121.91
77.16 CDCl3
52.27
N
O
N
Br
OMe
O
36
^{13}C NMR
(150 MHz, CDCl$_3$)
200 190 180 170 160 150 140 130 120 110 100 90 80 70 60 50 40 30 20 10 0
f1 (ppm)

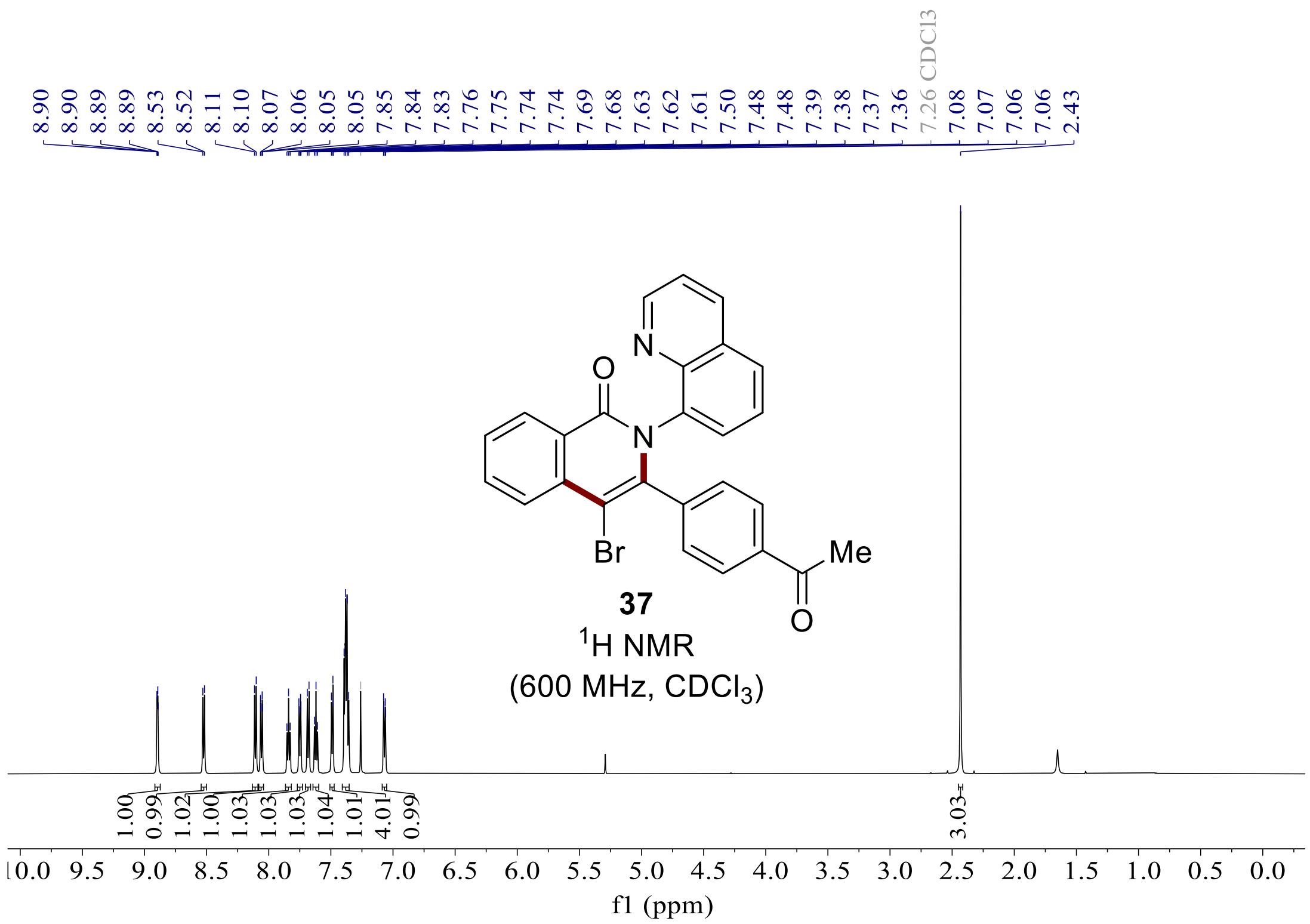

8.90
8.90
8.89
8.89
8.53
8.52
8.11
8.10
8.07
8.06
8.05
8.05
7.85
7.84
7.83
7.76
7.75
7.74
7.74
7.69
7.68
7.63
7.62
7.61
7.50
7.48
7.48
7.39
7.38
7.37
7.36
7.26 CDCl3
7.08
7.07
7.06
7.06
2.43
N
O
N
Br
Me
O
37
^{1}H NMR
(600 MHz, CDCl$_3$)
1.00
0.99
1.02
1.00
1.03
1.03
1.03
1.04
1.01
4.01
0.99
3.03
10.0 9.5 9.0 8.5 8.0 7.5 7.0 6.5 6.0 5.5 5.0 4.5 4.0 3.5 3.0 2.5 2.0 1.5 1.0 0.5 0.0
f1 (ppm)

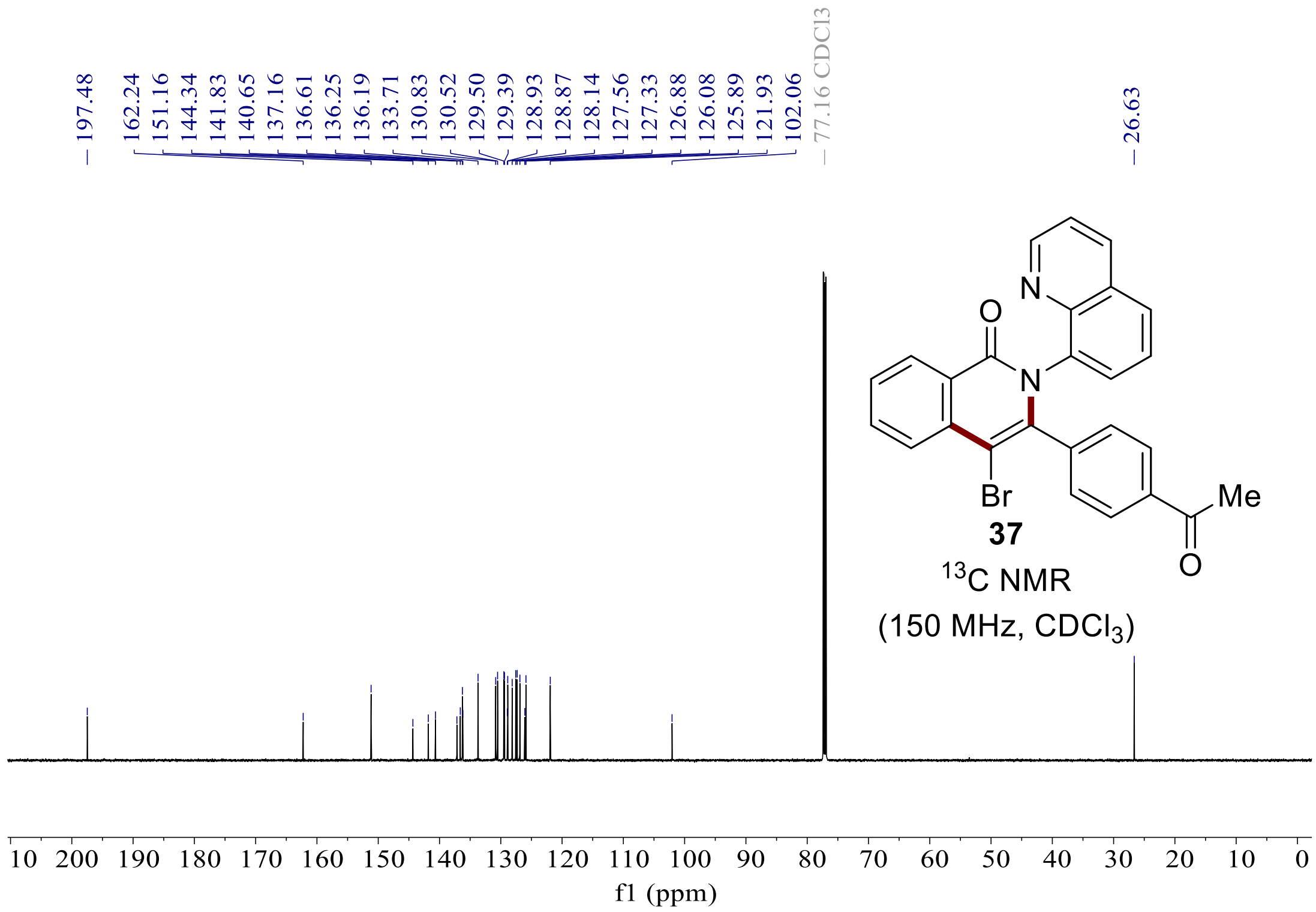
197.48
162.24
151.16
144.34
141.83
140.65
137.16
136.61
136.25
136.19
133.71
130.83
130.52
129.50
129.39
128.93
128.87
128.14
127.56
127.33
126.88
126.08
125.89
121.93
102.06
77.16 CDCl3
26.63
O
N
N
Br
Me
O
37
^{13}C NMR
(150 MHz, $CDCl_3$)
f1 (ppm)

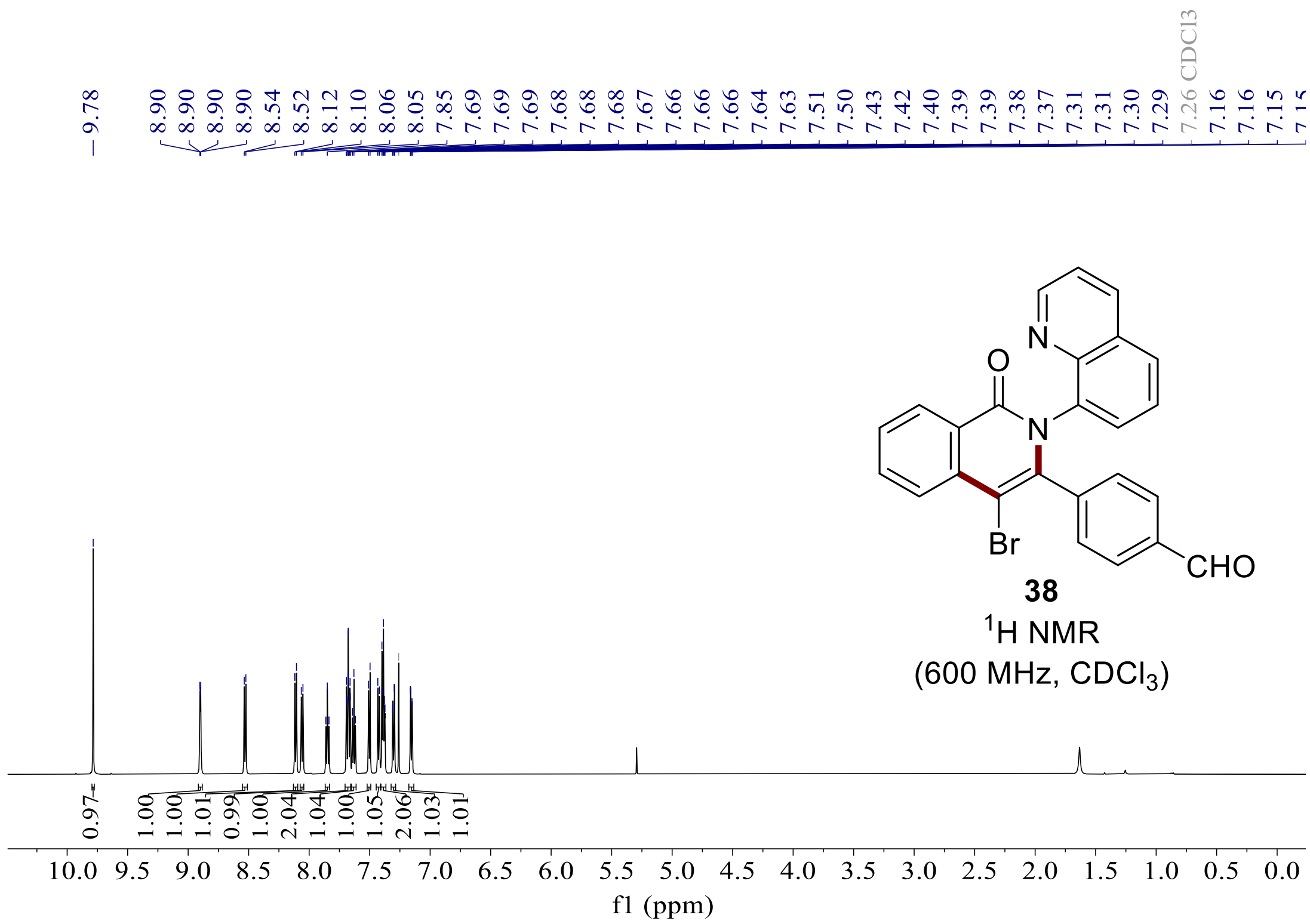
9.78
8.90
8.90
8.90
8.90
8.54
8.52
8.12
8.10
8.06
8.05
7.85
7.69
7.69
7.69
7.68
7.68
7.68
7.67
7.66
7.66
7.66
7.64
7.63
7.51
7.50
7.43
7.42
7.40
7.39
7.39
7.38
7.37
7.31
7.31
7.30
7.29
7.26 CDCl3
7.16
7.16
7.15
O
N
N
Br
CHO
38
^{1}H NMR
(600 MHz, $CDCl_3$)
0.97
1.00
1.00
1.01
0.99
1.00
2.04
1.04
1.00
1.05
2.06
1.03
1.01
f1 (ppm)

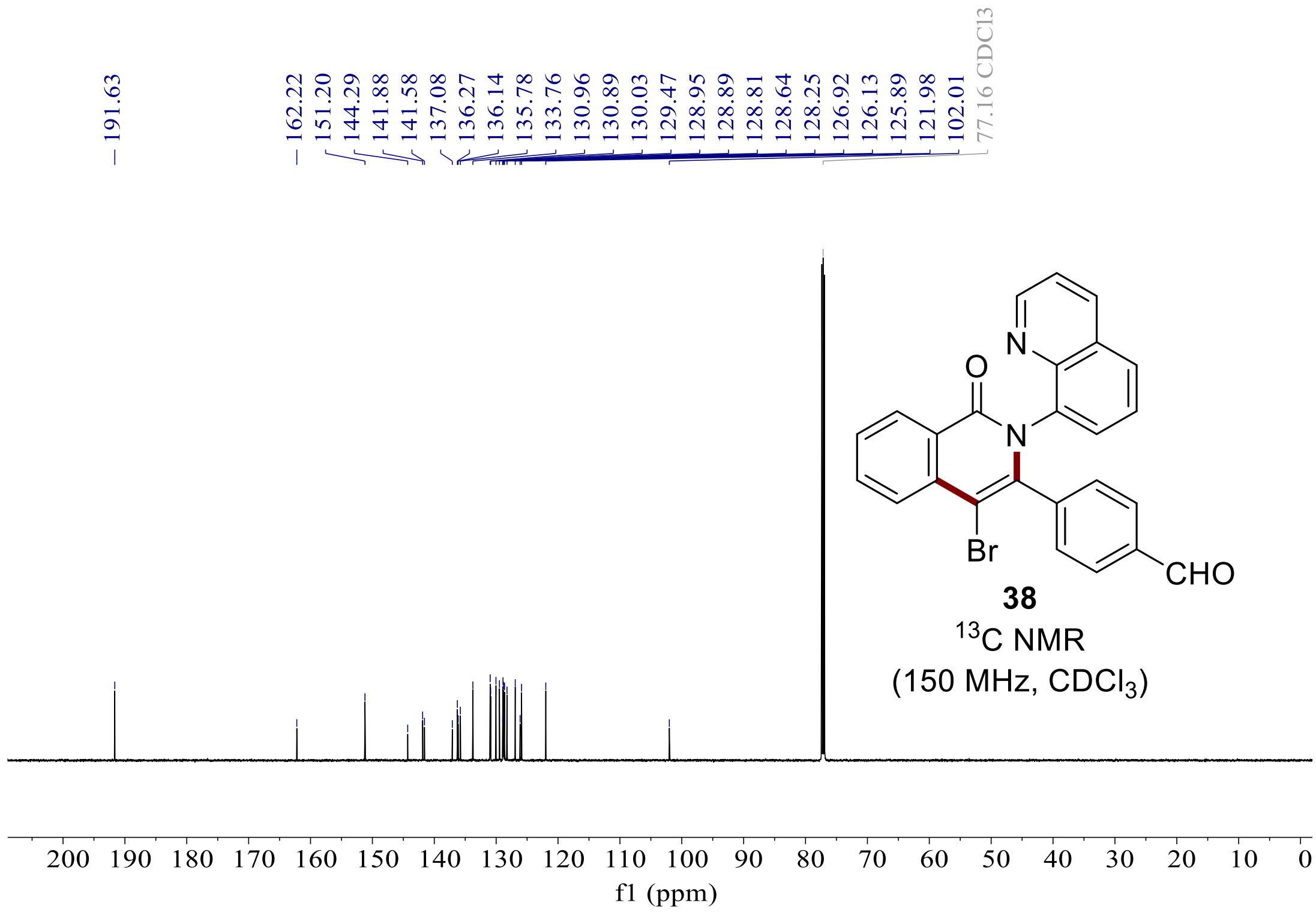
191.63
162.22
151.20
144.29
141.88
141.58
137.08
136.27
136.14
135.78
133.76
130.96
130.89
130.03
129.47
128.95
128.89
128.81
128.64
128.25
126.92
126.13
125.89
121.98
102.01
77.16 CDCl3
O
N
N
Br
CHO
38
13C NMR
(150 MHz, CDCl3)
200 190 180 170 160 150 140 130 120 110 100 90 80 70 60 50 40 30 20 10 0
f1 (ppm)

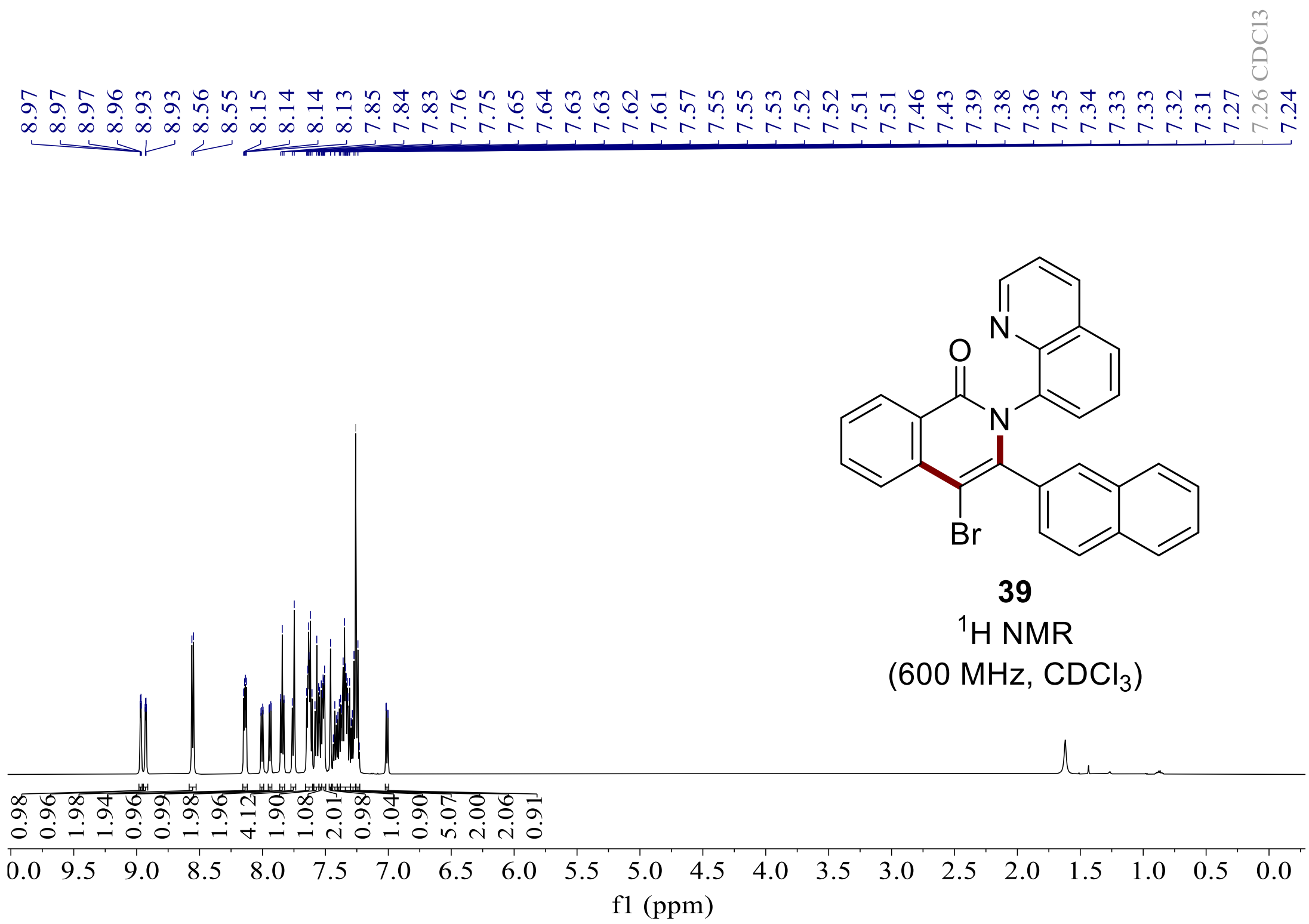
8.97
8.97
8.97
8.96
8.93
8.93
8.56
8.55
8.15
8.14
8.14
8.13
7.85
7.84
7.83
7.76
7.75
7.65
7.64
7.63
7.63
7.62
7.61
7.57
7.55
7.55
7.53
7.52
7.52
7.51
7.51
7.46
7.43
7.39
7.38
7.36
7.35
7.34
7.33
7.33
7.32
7.31
7.27
7.26 CDCl3
7.24
O
N
N
Br
39
1H NMR
(600 MHz, CDCl3)
0.98
0.96
1.98
1.94
0.96
0.99
1.98
1.96
4.12
1.90
1.08
2.01
0.98
1.04
0.90
5.07
2.00
2.06
0.91
0.0 9.5 9.0 8.5 8.0 7.5 7.0 6.5 6.0 5.5 5.0 4.5 4.0 3.5 3.0 2.5 2.0 1.5 1.0 0.5 0.0
f1 (ppm)

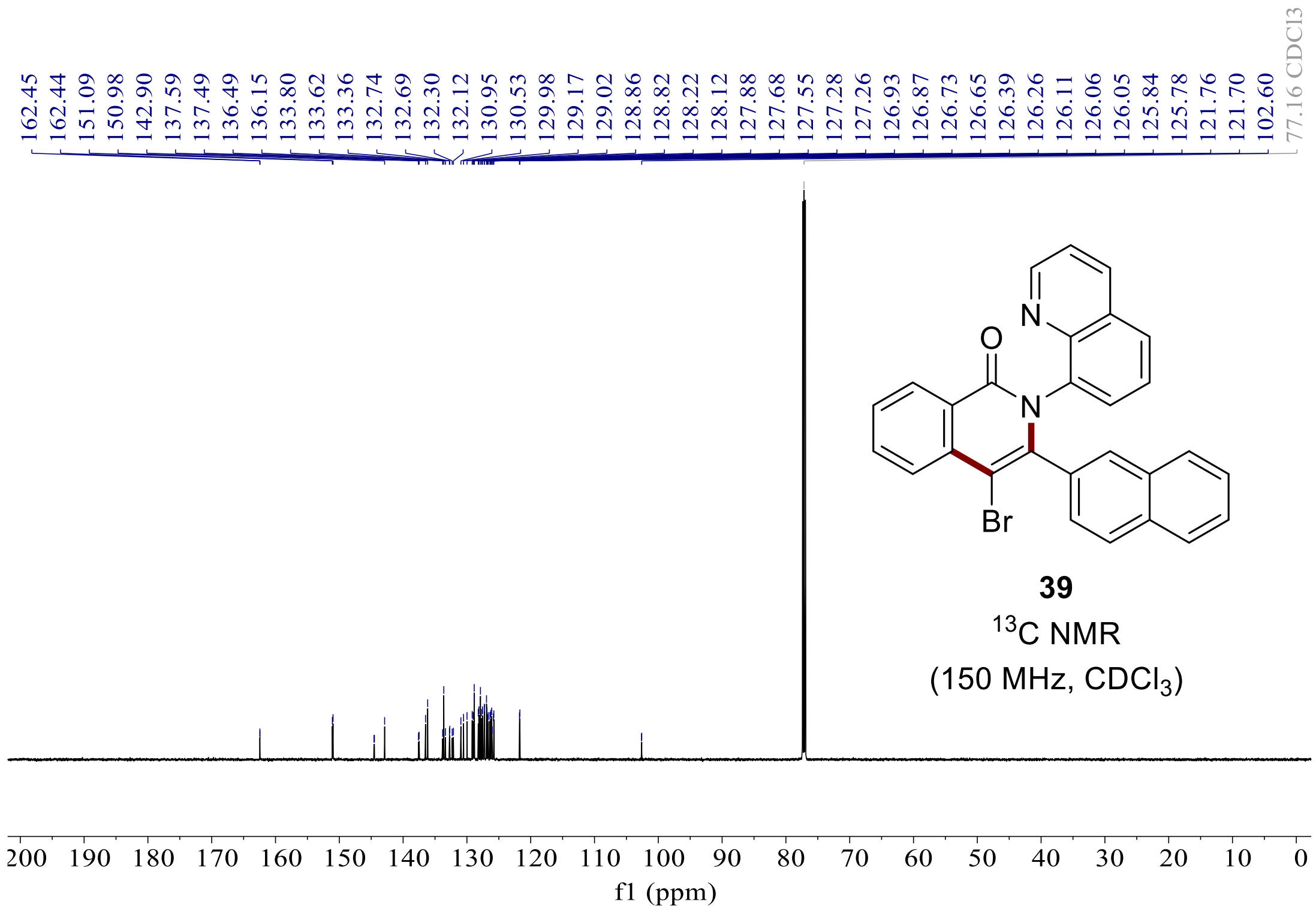
162.45
162.44
151.09
150.98
142.90
137.59
137.49
136.49
136.15
133.80
133.62
133.36
132.74
132.69
132.30
132.12
130.95
130.53
129.98
129.17
129.02
128.86
128.82
128.22
128.12
127.88
127.68
127.55
127.28
127.26
126.93
126.87
126.73
126.65
126.39
126.26
126.11
126.06
126.05
125.84
125.78
121.76
121.70
102.60
77.16 CDCl3
39
^{13}C NMR
(150 MHz, CDCl$_3$)
f1 (ppm)

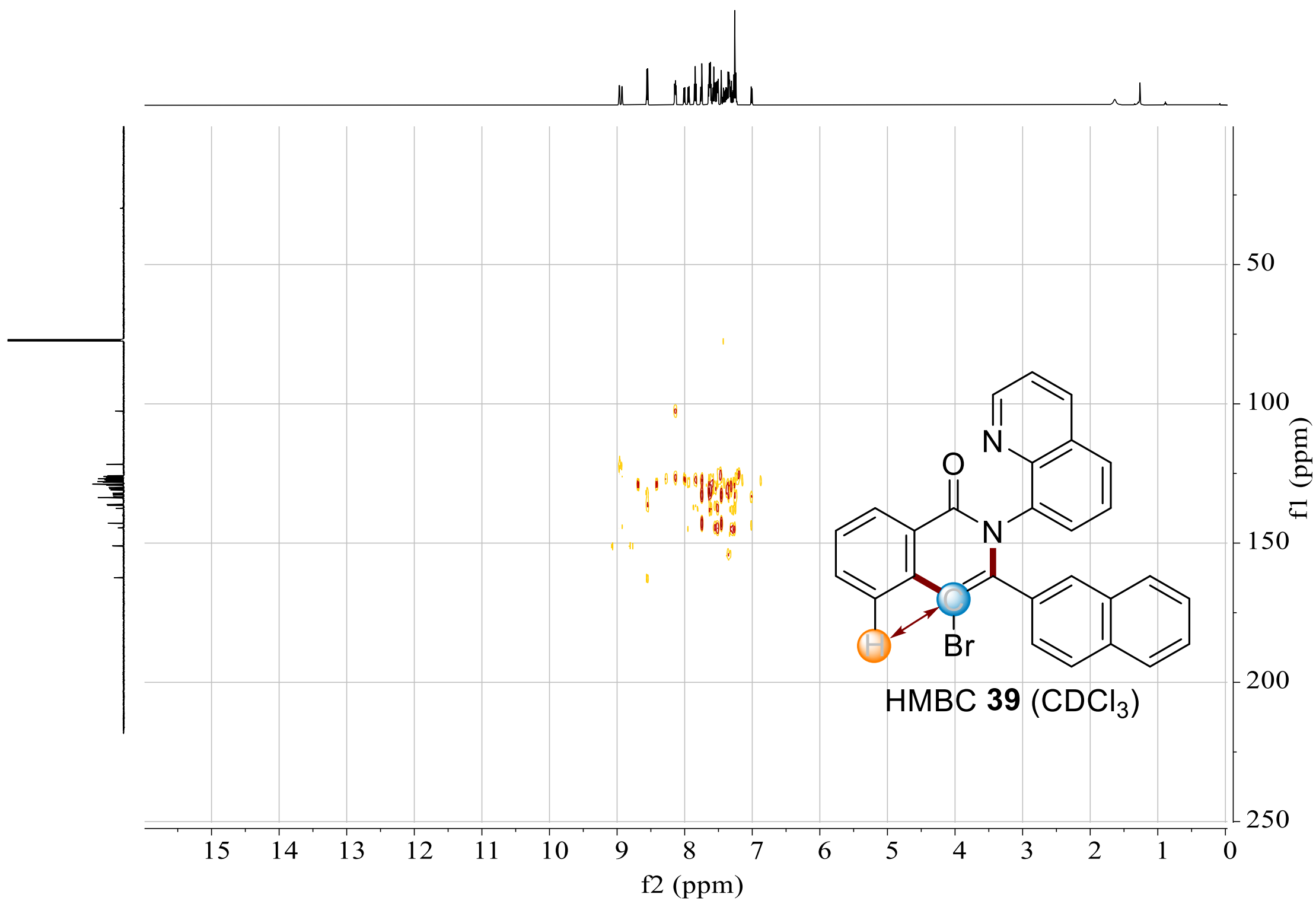
HMBC 39 (CDCl$_3$)
f2 (ppm)
f1 (ppm)

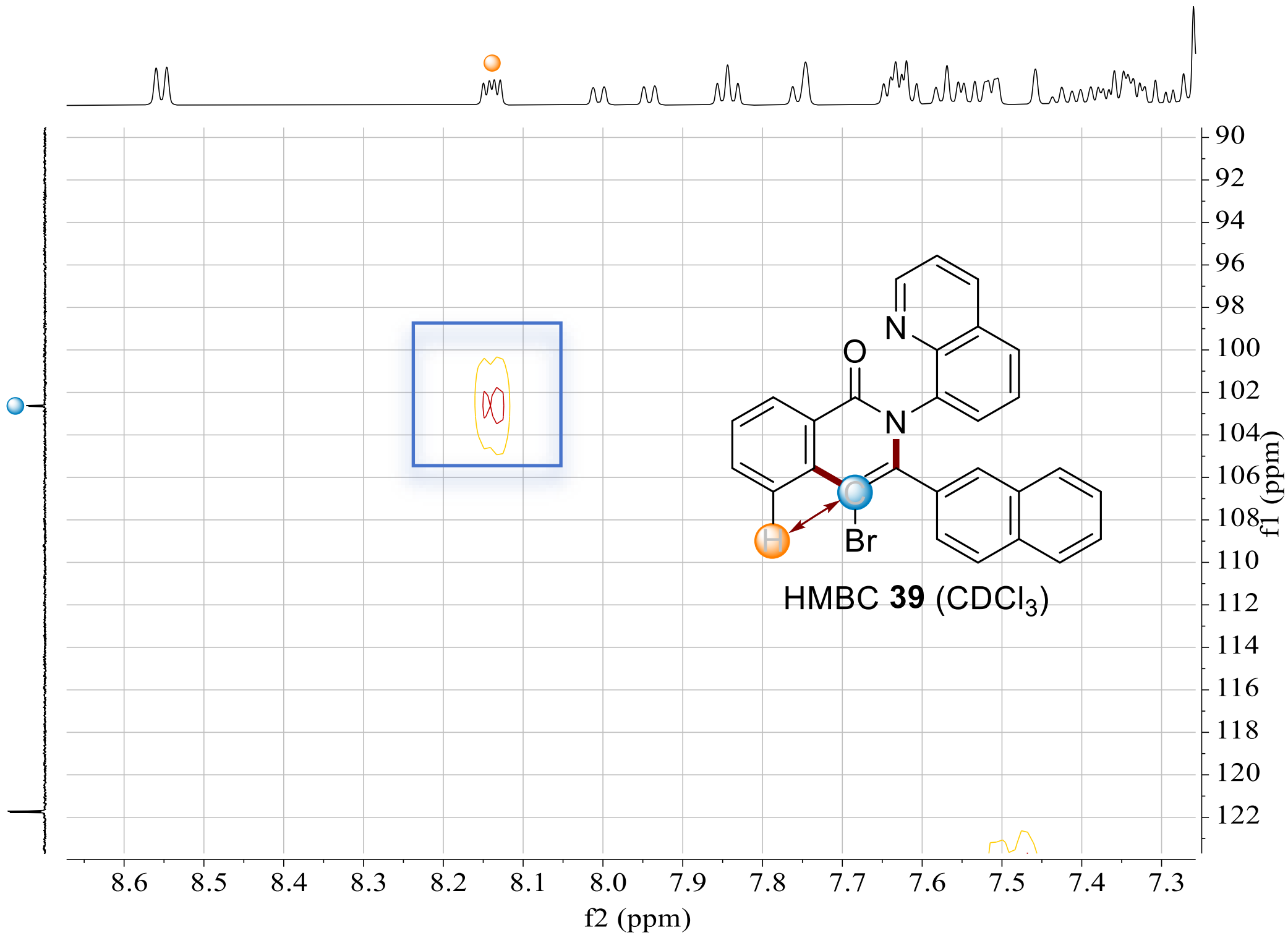
HMBC 39 (CDCl3)
f2 (ppm)
f1 (ppm)
Br
O
N
N

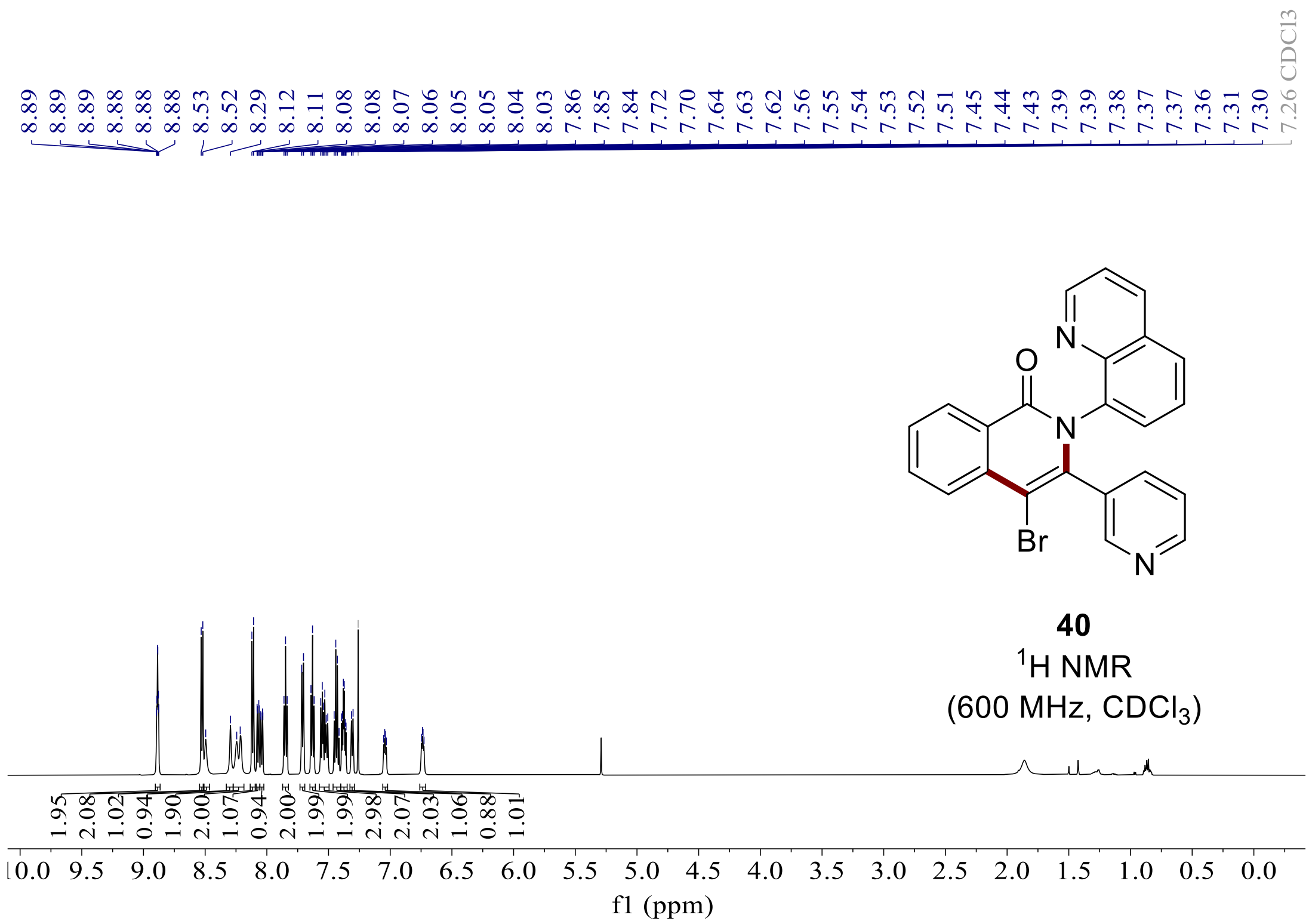
8.89 8.89 8.89 8.88 8.88 8.88 8.53 8.52 8.29 8.12 8.11 8.08 8.08 8.07 8.06 8.05 8.05 8.04 8.03 7.86 7.85 7.84 7.72 7.70 7.64 7.63 7.62 7.56 7.55 7.54 7.53 7.52 7.51 7.45 7.44 7.43 7.39 7.39 7.38 7.37 7.37 7.36 7.31 7.30 7.26 CDCl3
40
1H NMR
(600 MHz, CDCl3)
1.95 2.08 1.02 0.94 1.90 2.00 1.07 0.94 2.00 1.99 1.99 2.98 2.07 2.03 1.06 0.88 1.01
f1 (ppm)

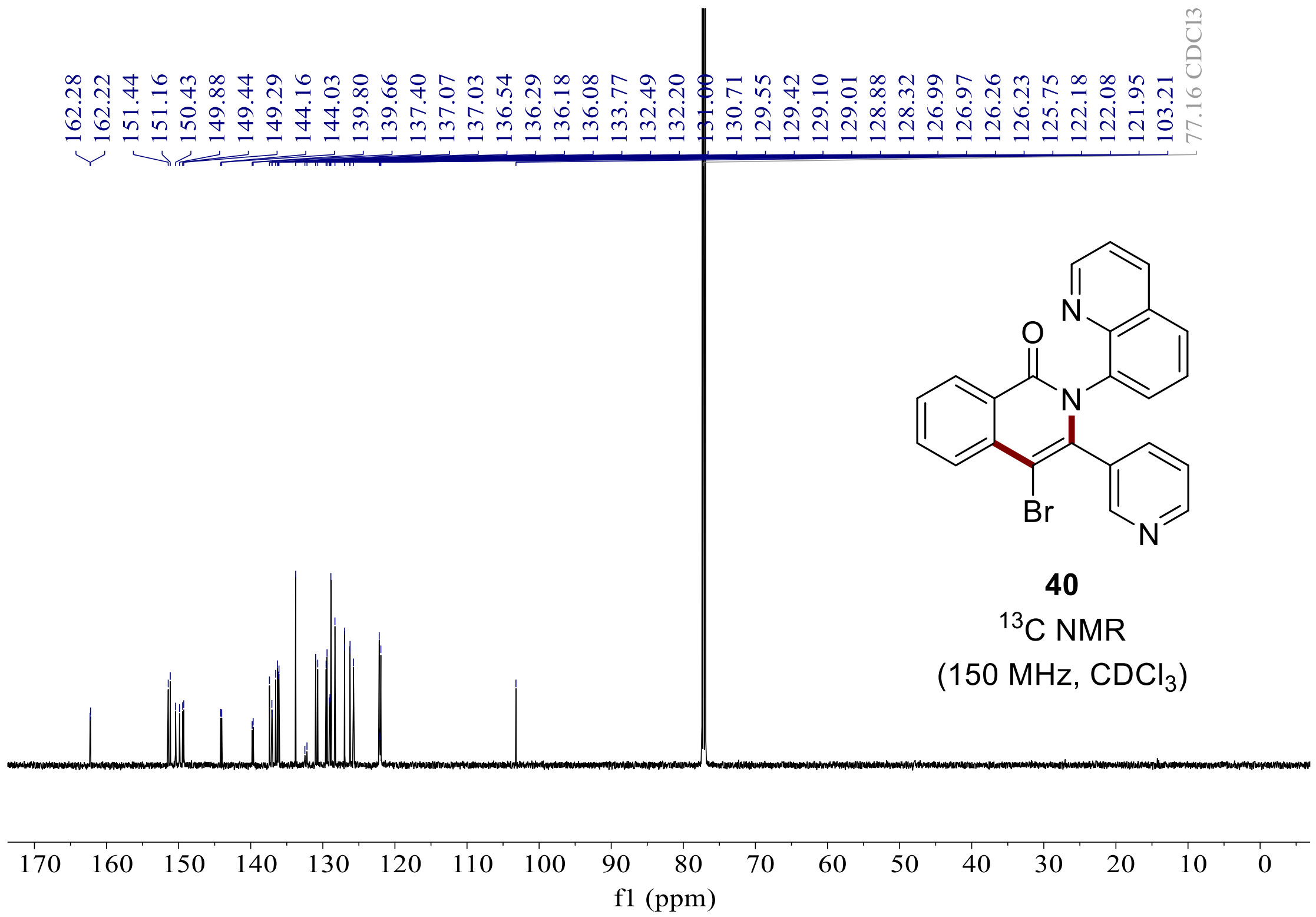
162.28
162.22
151.44
151.16
150.43
149.88
149.44
149.29
144.16
144.03
139.80
139.66
137.40
137.07
137.03
136.54
136.29
136.18
136.08
133.77
132.49
132.20
131.00
130.71
129.55
129.42
129.10
129.01
128.88
128.32
126.99
126.97
126.26
126.23
125.75
122.18
122.08
121.95
103.21
77.16 CDCl3
O
N
N
Br
N
40
^{13}C NMR
(150 MHz, $CDCl_3$)
f1 (ppm)

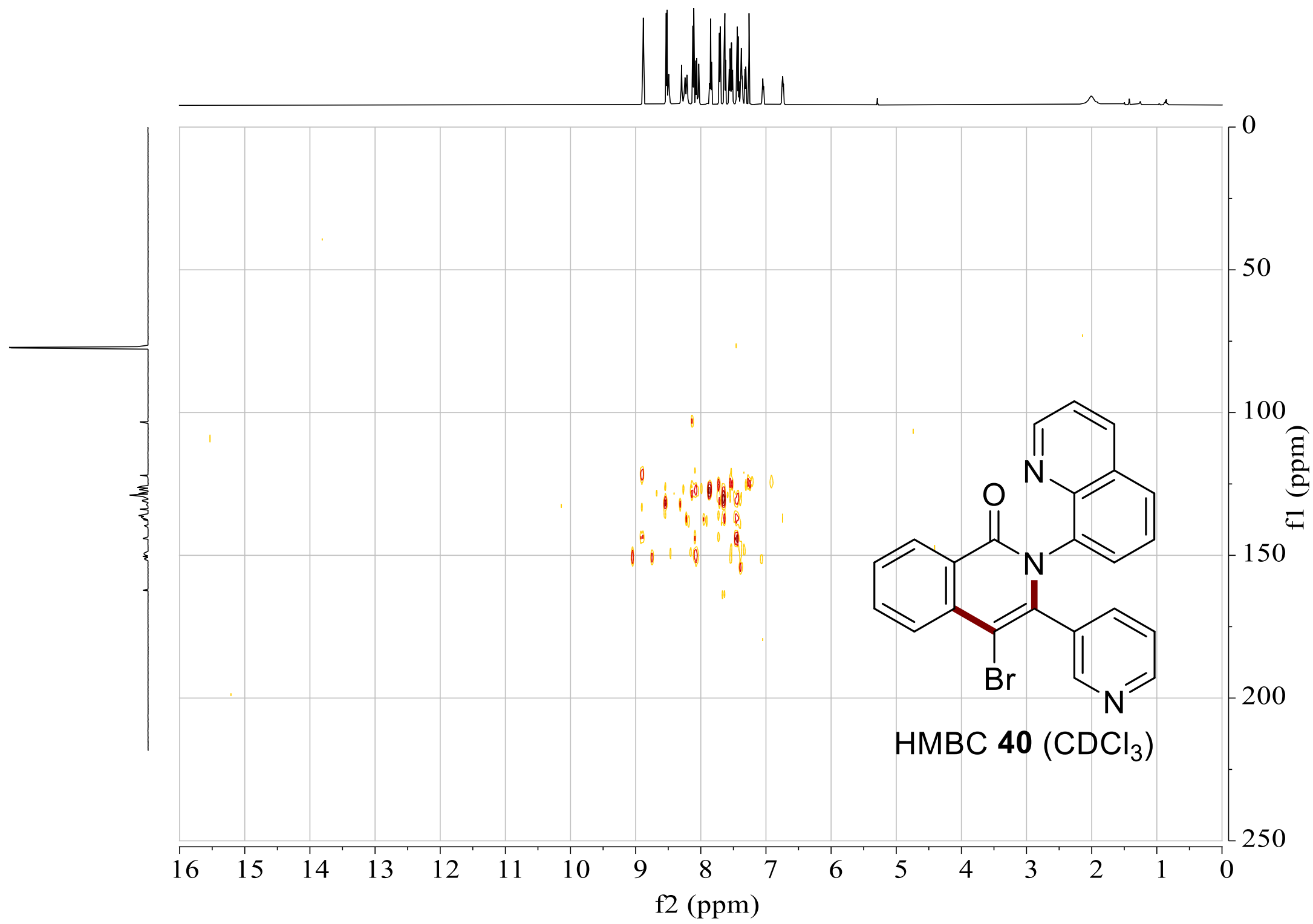
O
N
N
Br
N
HMBC **40** ($CDCl_3$)
f2 (ppm)
f1 (ppm)

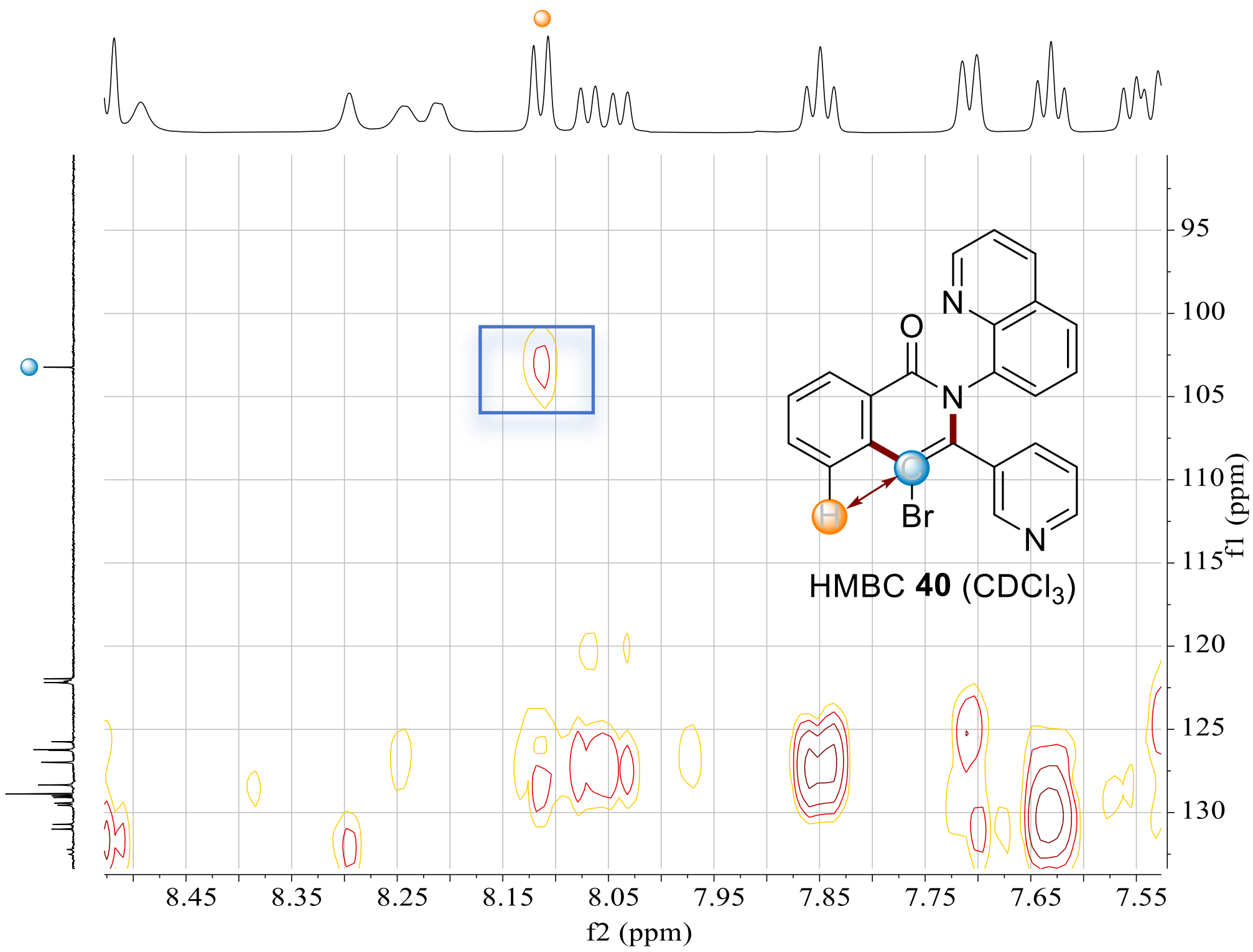

HMBC 40 (CDCl3)
f2 (ppm)
f1 (ppm)

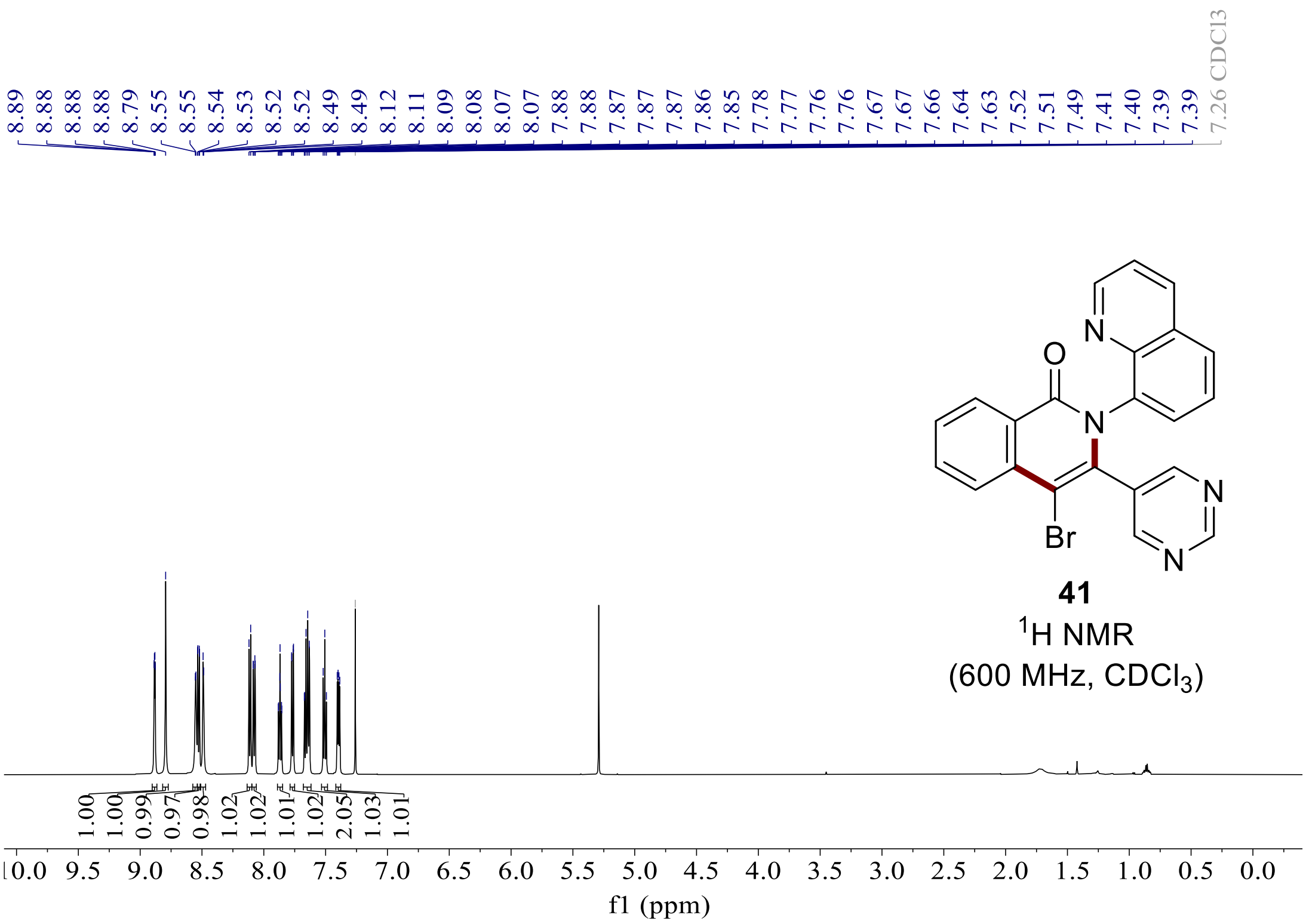

41
1H NMR
(600 MHz, CDCl3)
f1 (ppm)

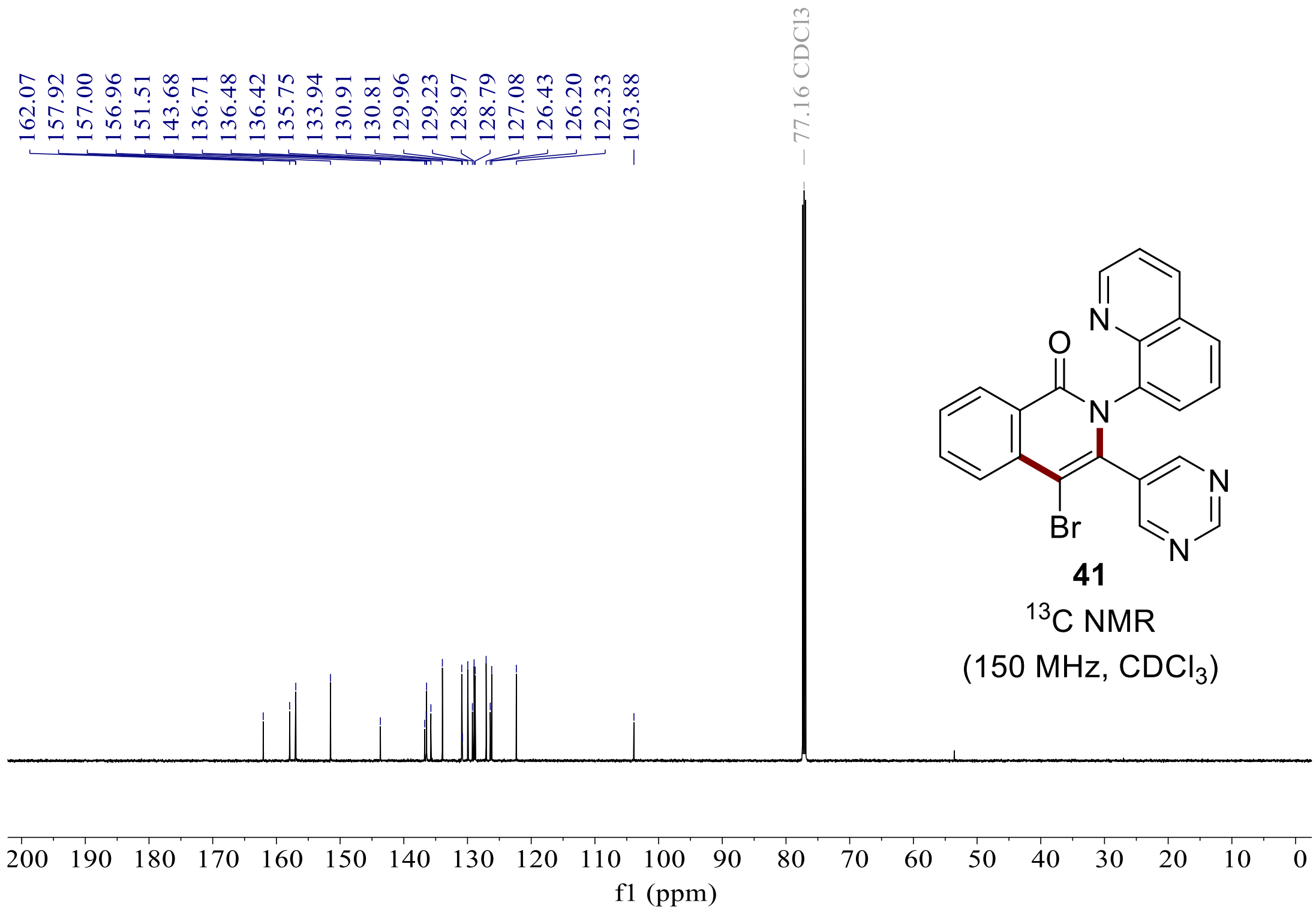

41
^{13}C NMR
(150 MHz, $CDCl_3$)

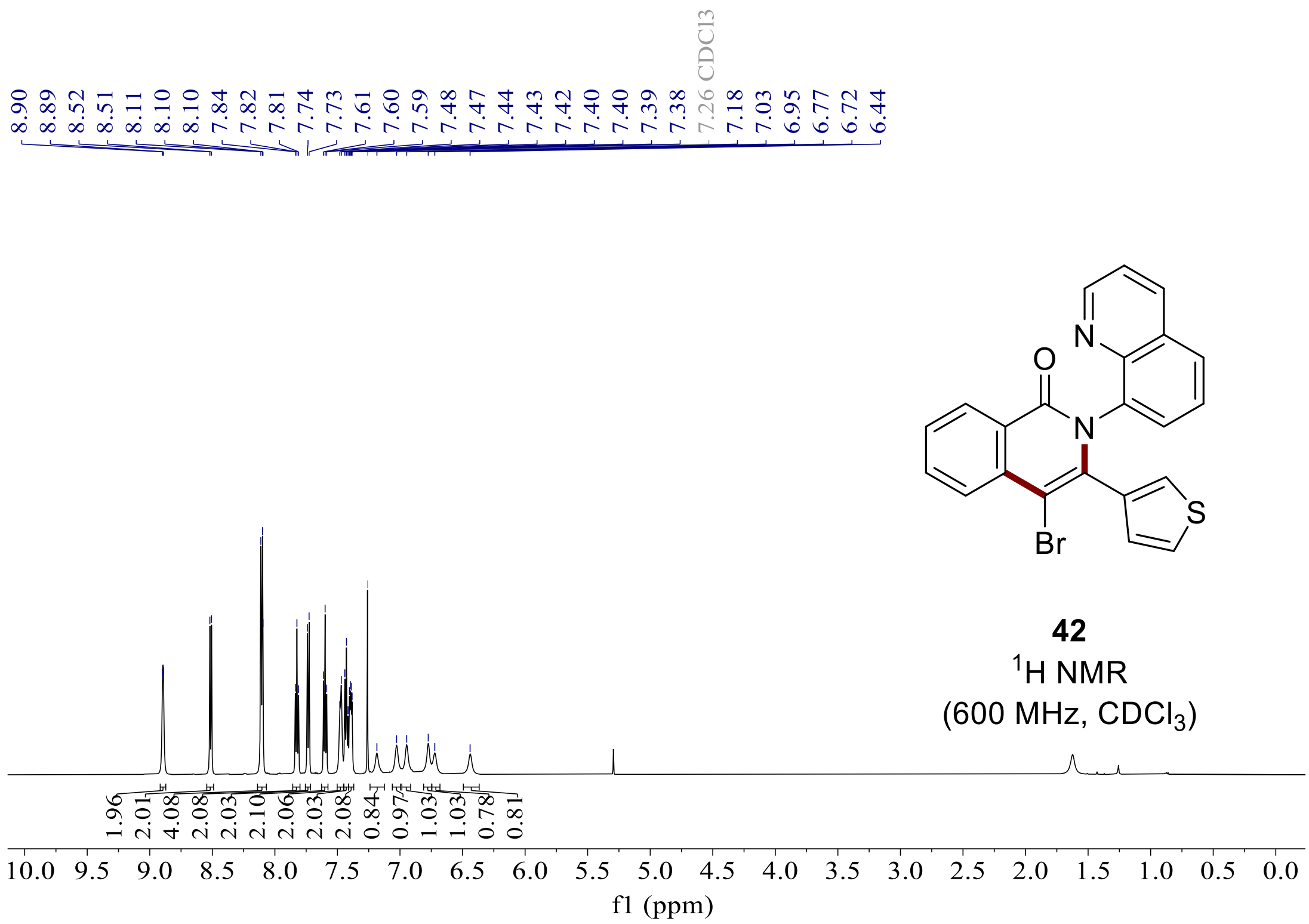

42
^{1}H NMR
(600 MHz, $CDCl_3$)

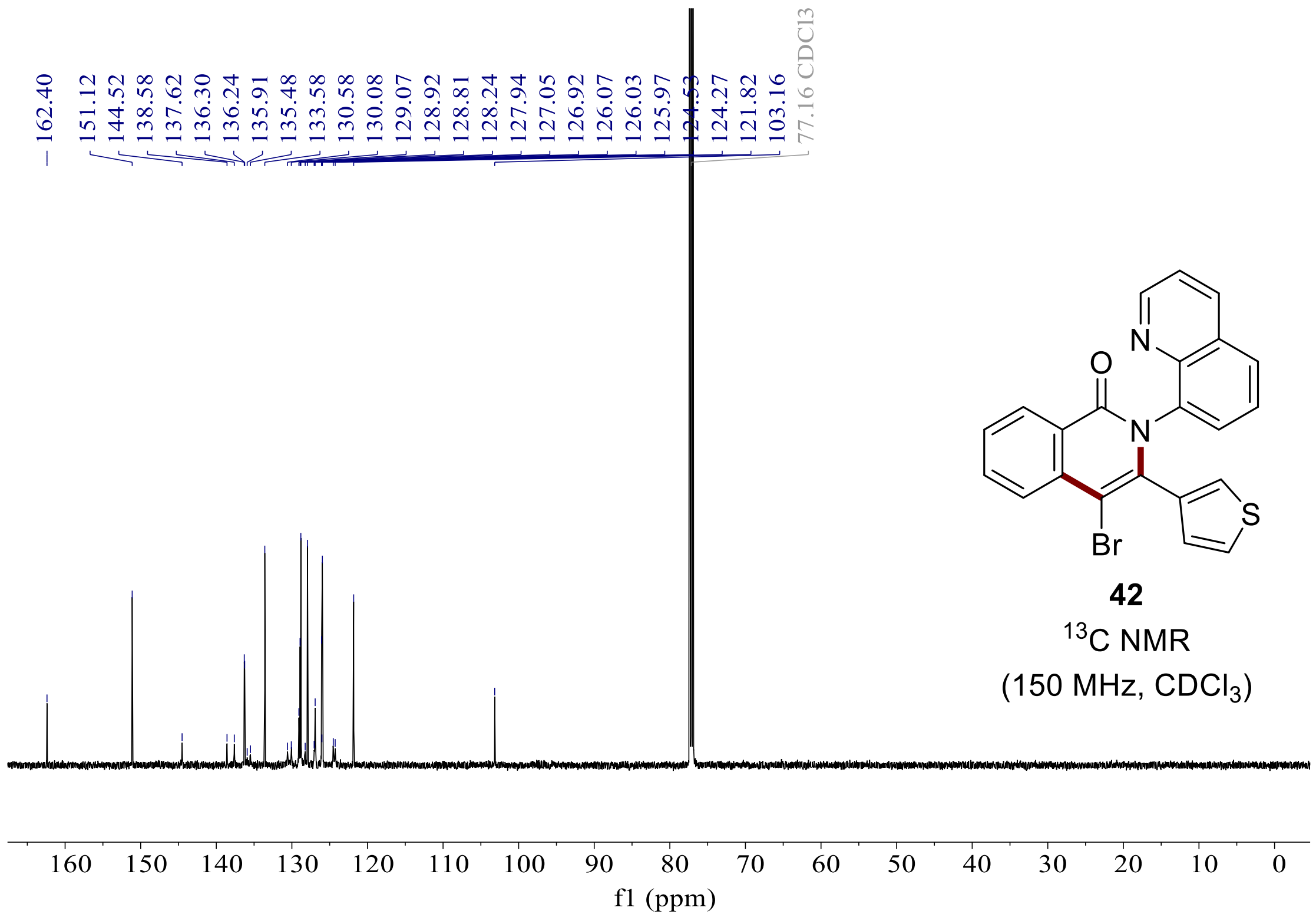

162.40
151.12
144.52
138.58
137.62
136.30
136.24
135.91
135.48
133.58
130.58
130.08
129.07
128.92
128.81
128.24
127.94
127.05
126.92
126.07
126.03
125.97
124.53
124.27
121.82
103.16
77.16 CDCl3
O
N
N
S
Br
42
13C NMR
(150 MHz, CDCl3)
160 150 140 130 120 110 100 90 80 70 60 50 40 30 20 10 0
f1 (ppm)

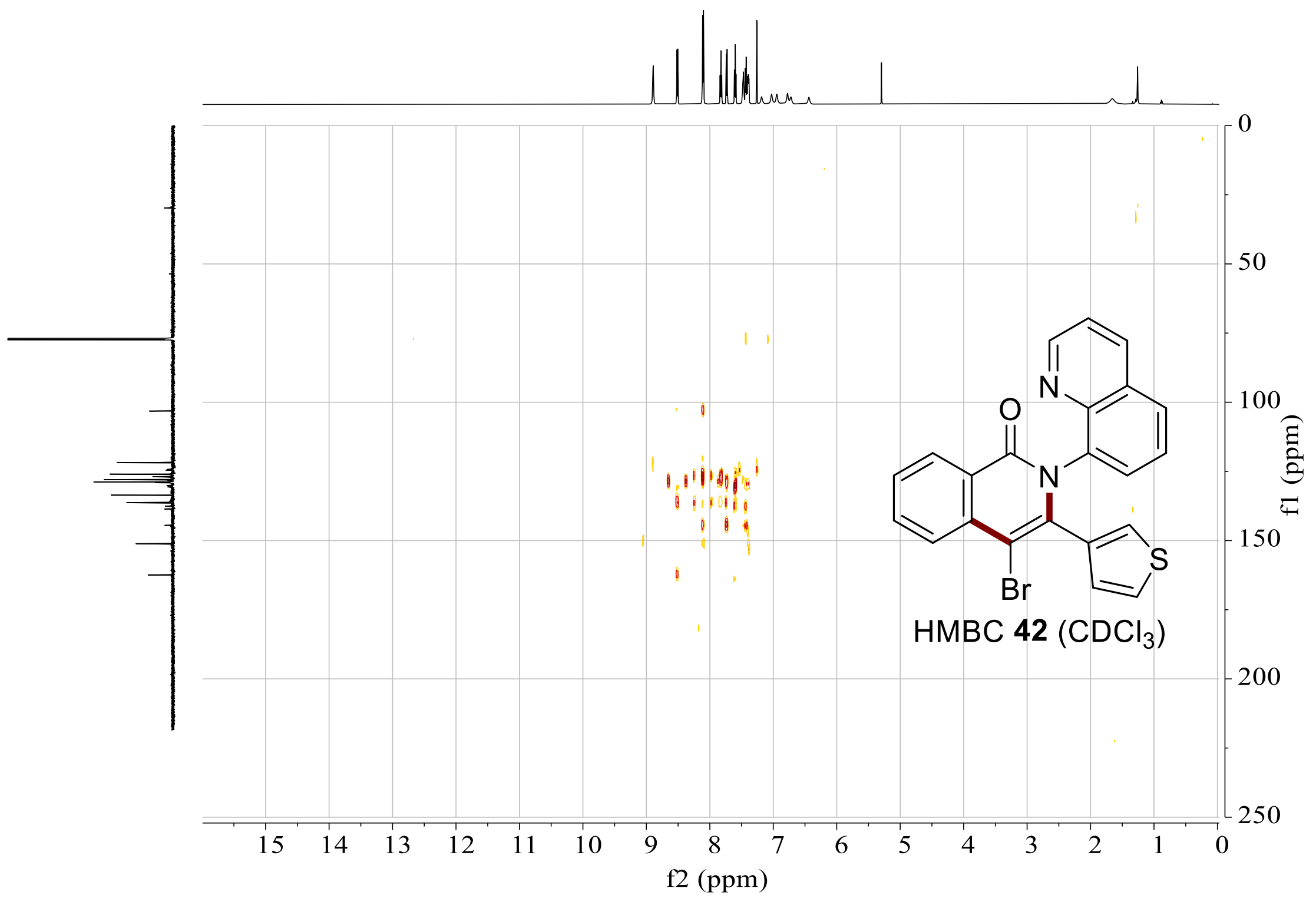

O
N
N
S
Br
HMBC 42 (CDCl3)
0 50 100 150 200 250
f1 (ppm)
15 14 13 12 11 10 9 8 7 6 5 4 3 2 1 0
f2 (ppm)

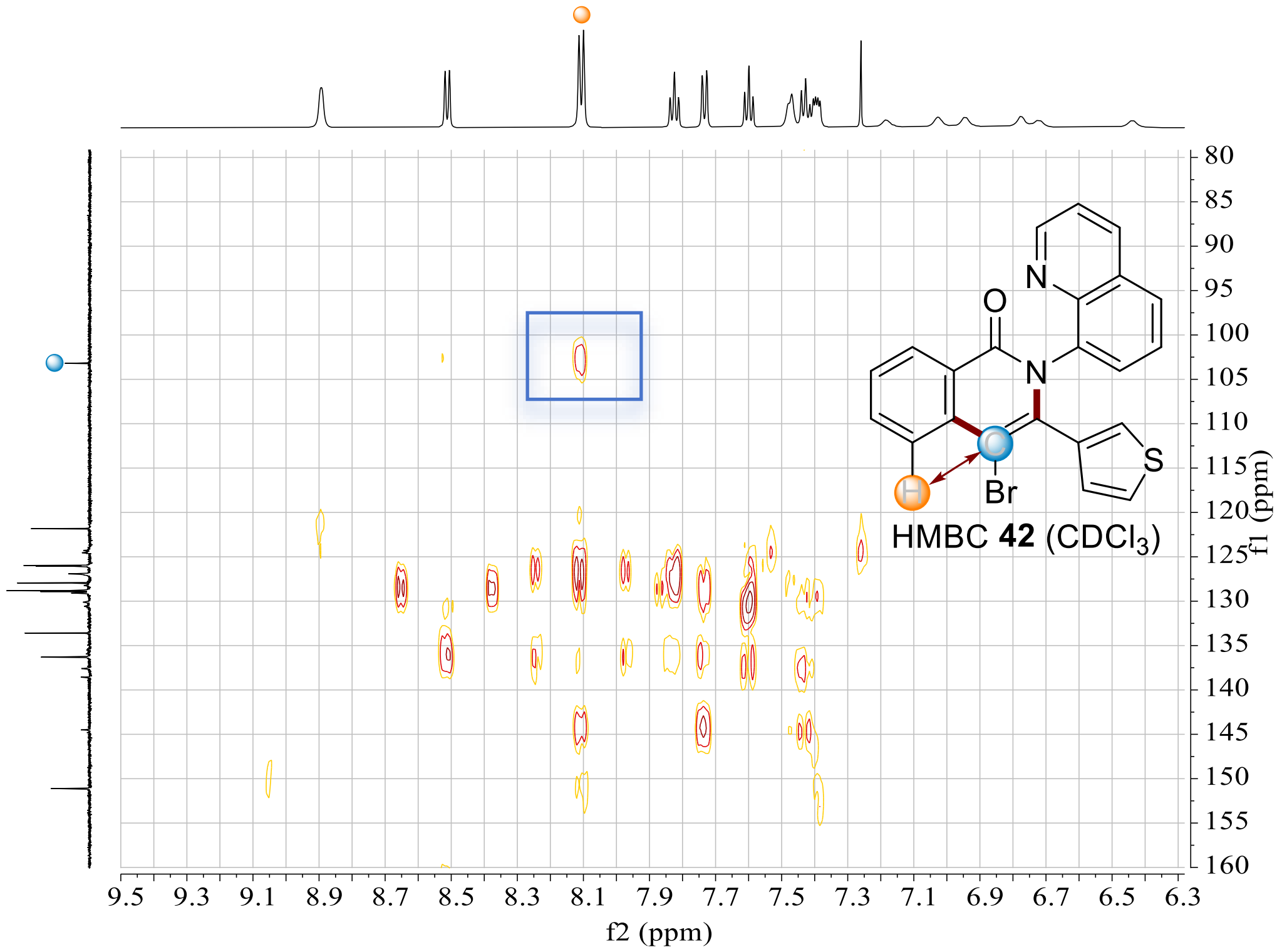

HMBC 42 (CDCl3)
f2 (ppm)
f1 (ppm)

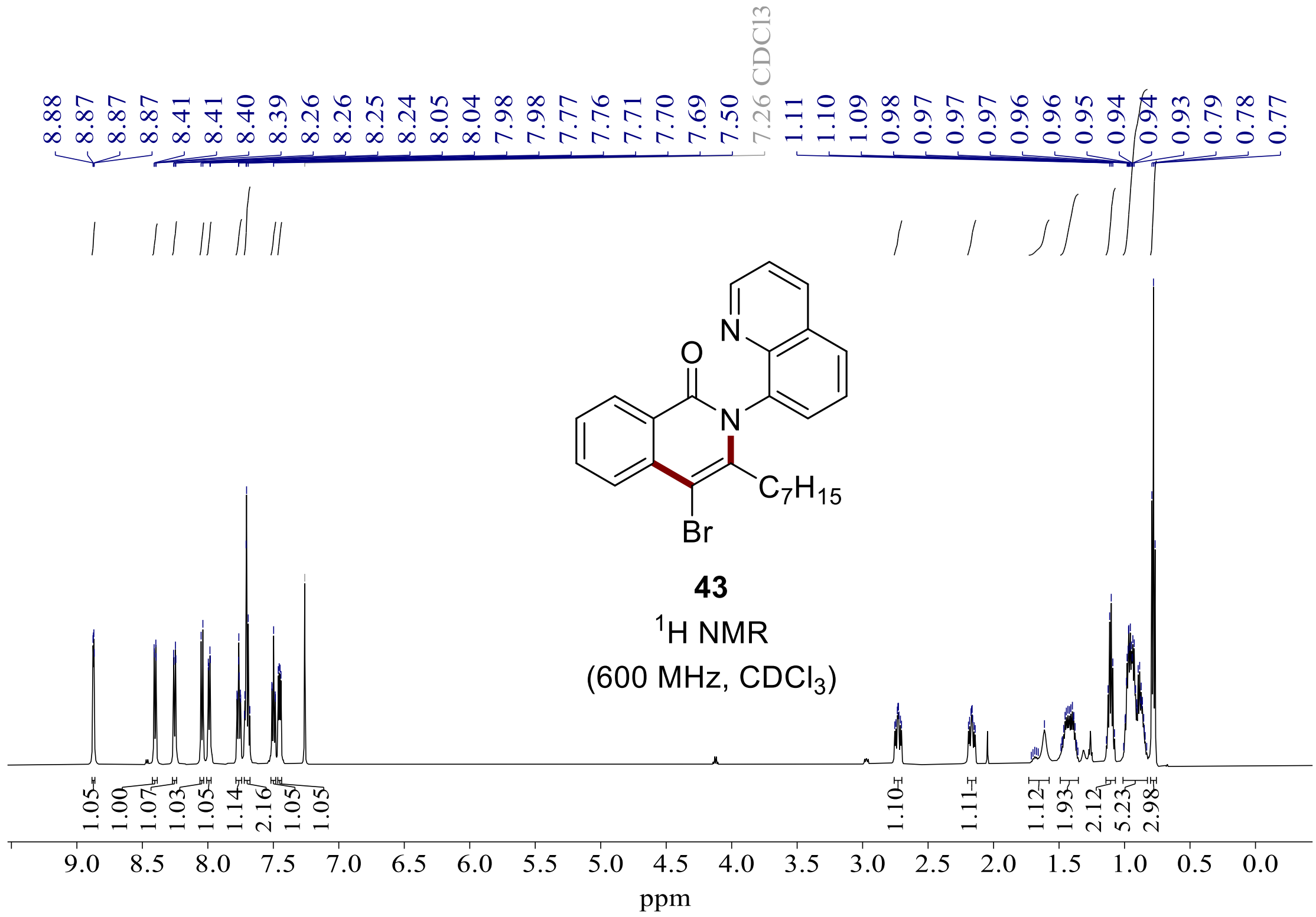

43
1H NMR
(600 MHz, CDCl3)
ppm
7.26 CDCl3

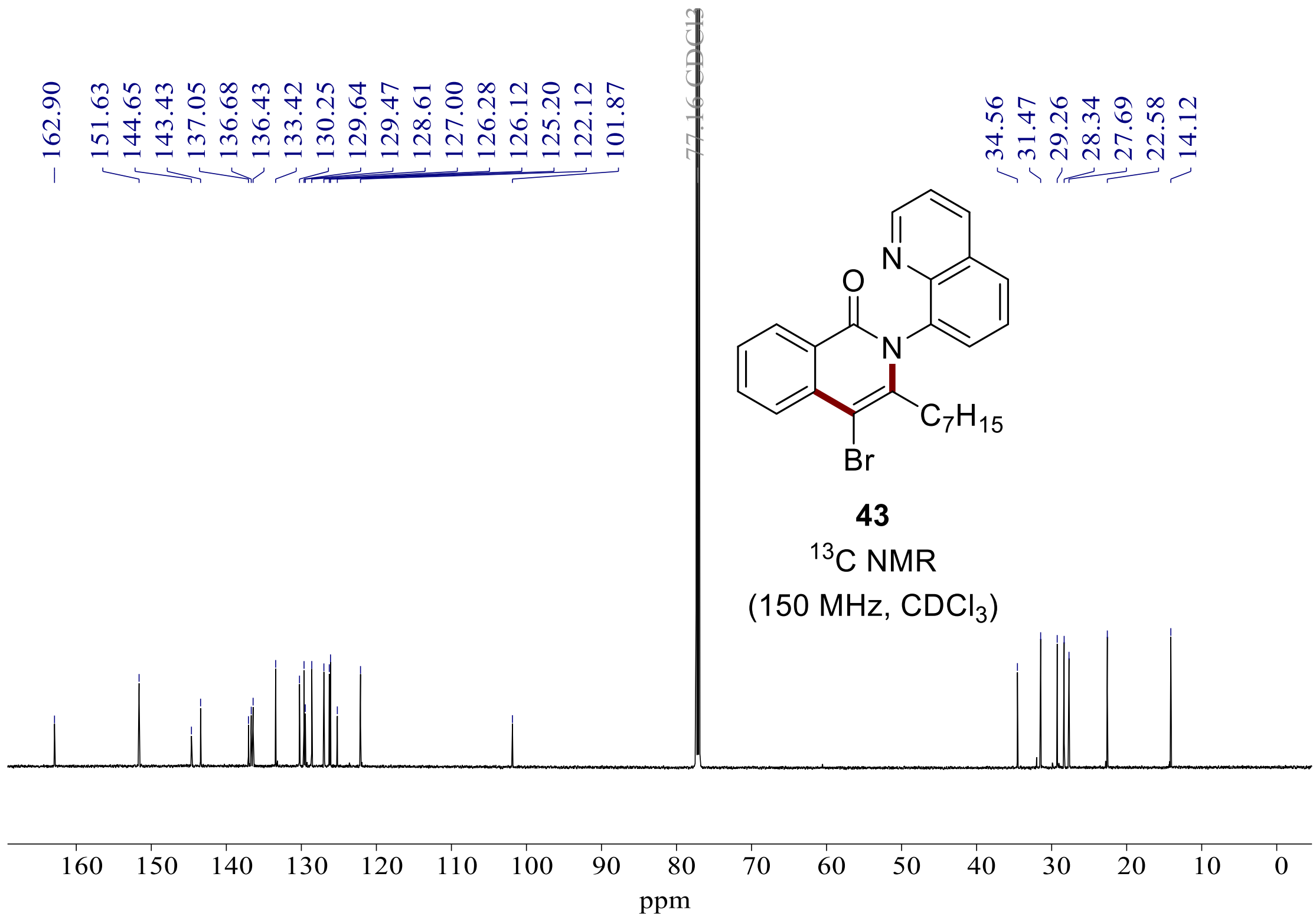

162.90
151.63
144.65
143.43
137.05
136.68
136.43
133.42
130.25
129.64
129.47
128.61
127.00
126.28
126.12
125.20
122.12
101.87
77.16 CDCl3
34.56
31.47
29.26
28.34
27.69
22.58
14.12
N
O
N
C_7H_{15}
Br
43
^{13}C NMR
(150 MHz, $CDCl_3$)
160 150 140 130 120 110 100 90 80 70 60 50 40 30 20 10 0
ppm

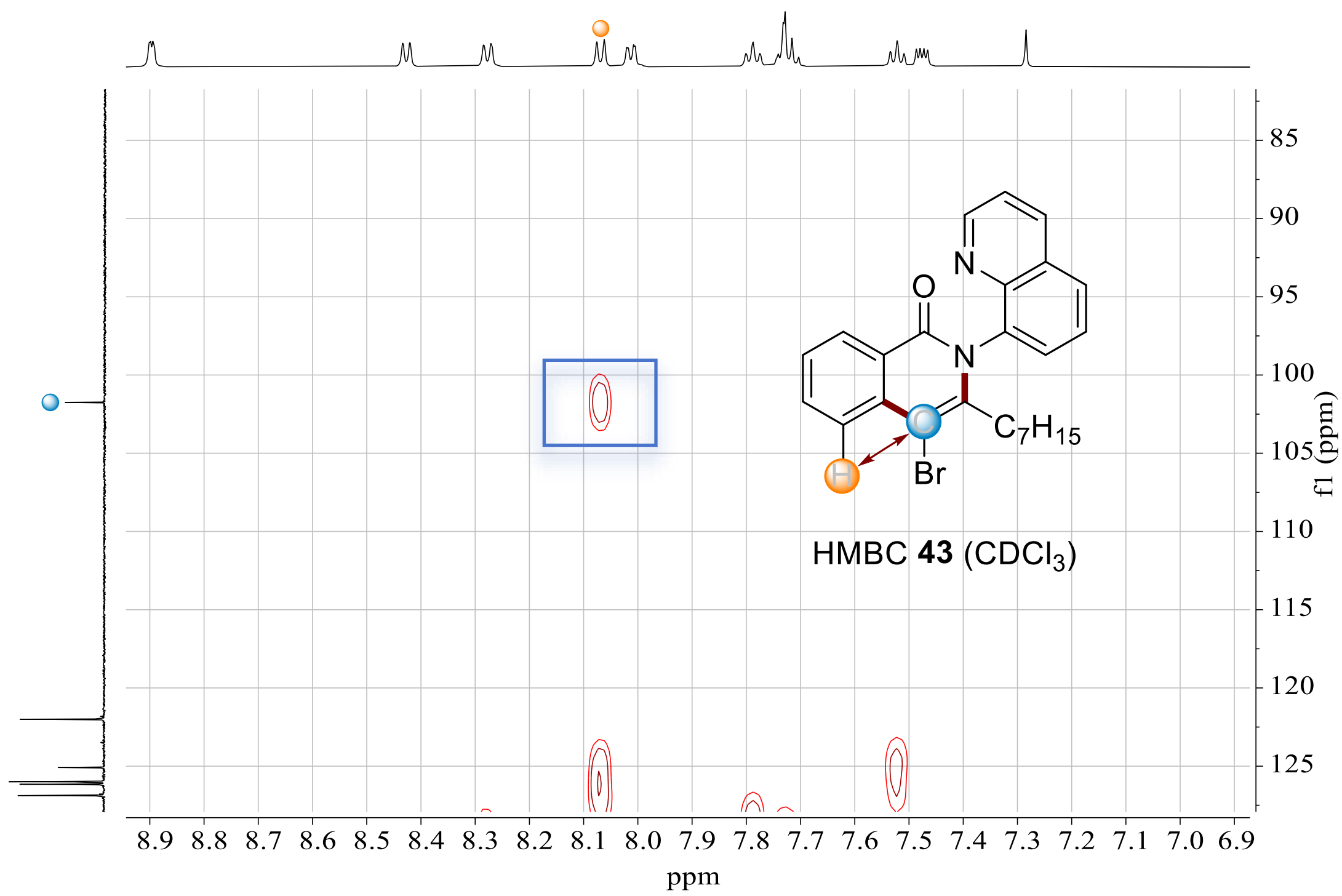

N
O
N
C_7H_{15}
H
Br
HMBC **43** ($CDCl_3$)
8.9 8.8 8.7 8.6 8.5 8.4 8.3 8.2 8.1 8.0 7.9 7.8 7.7 7.6 7.5 7.4 7.3 7.2 7.1 7.0 6.9
ppm
85 90 95 100 105 110 115 120 125
f1 (ppm)

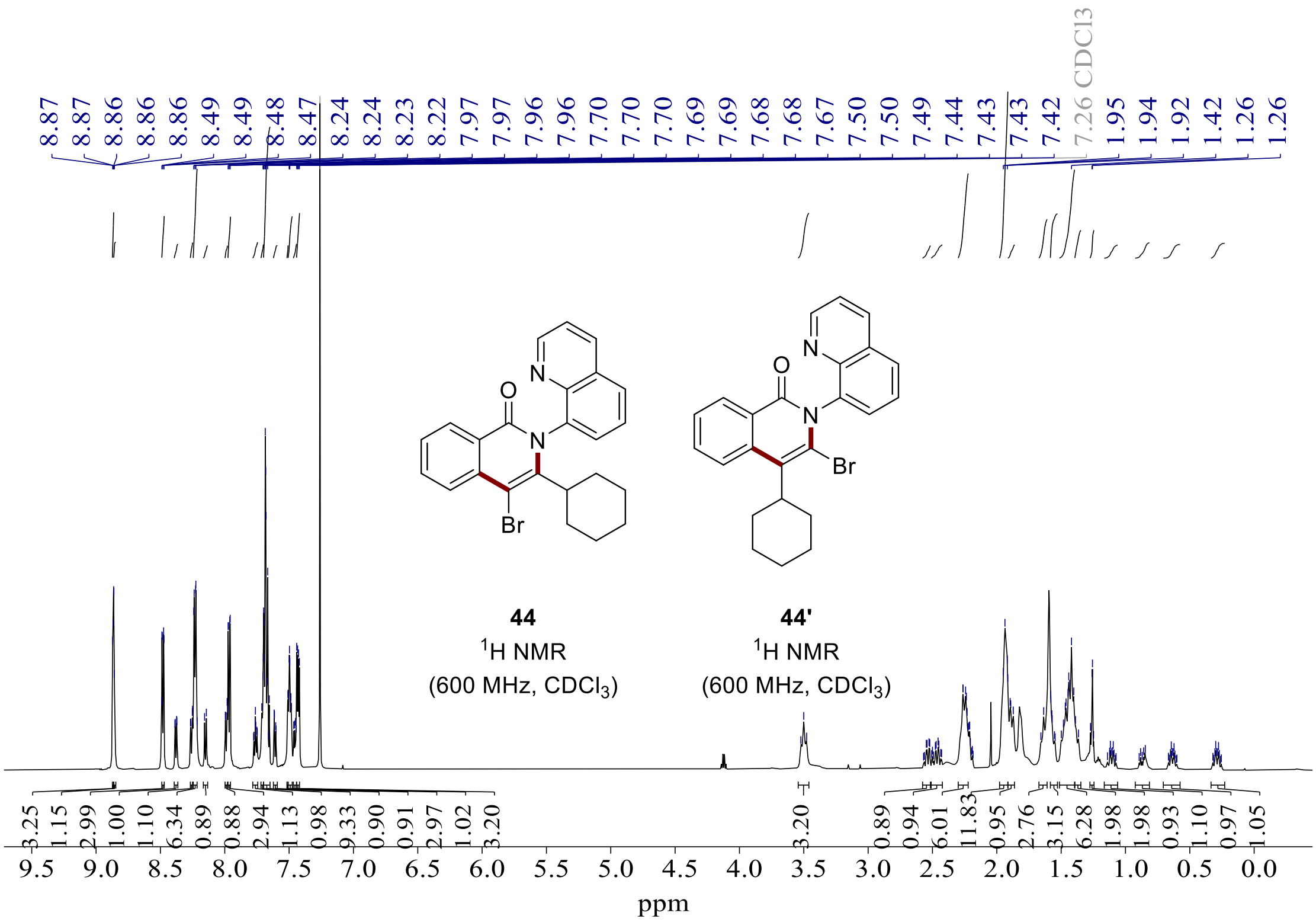
8.87 8.87 8.86 8.86 8.86 8.49 8.49 8.48 8.47 8.24 8.24 8.23 8.22 7.97 7.97 7.96 7.96 7.70 7.70 7.70 7.69 7.69 7.68 7.68 7.67 7.50 7.50 7.49 7.44 7.43 7.43 7.42 7.26 CDCl3 1.95 1.94 1.92 1.42 1.26 1.26
44
^{1}H NMR
(600 MHz, CDCl$_3$)
44'
^{1}H NMR
(600 MHz, CDCl$_3$)
3.25 1.15 2.99 1.00 1.10 6.34 0.89 0.88 2.94 1.13 0.98 9.33 0.90 0.91 2.97 1.02 3.20 3.20 0.89 0.94 6.01 11.83 0.95 2.76 3.15 6.28 1.98 1.98 0.93 1.10 0.97 1.05
9.5 9.0 8.5 8.0 7.5 7.0 6.5 6.0 5.5 5.0 4.5 4.0 3.5 3.0 2.5 2.0 1.5 1.0 0.5 0.0
ppm

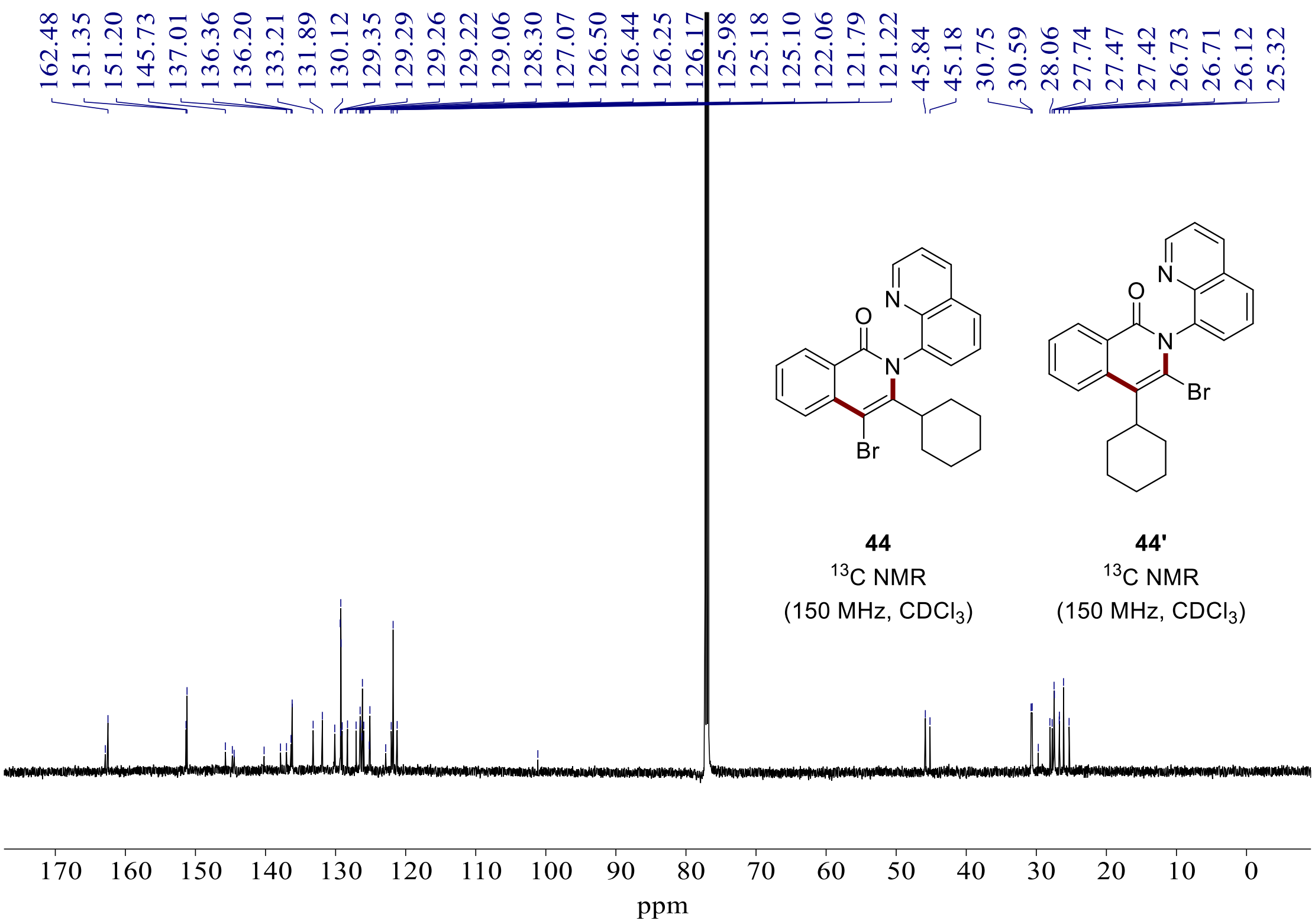
162.48 151.35 151.20 145.73 137.01 136.36 136.20 133.21 131.89 130.12 129.35 129.29 129.26 129.22 129.06 128.30 127.07 126.50 126.44 126.25 126.17 125.98 125.18 125.10 122.06 121.79 121.22 45.84 45.18 30.75 30.59 28.06 27.74 27.47 27.42 26.73 26.71 26.12 25.32
44
^{13}C NMR
(150 MHz, CDCl$_3$)
44'
^{13}C NMR
(150 MHz, CDCl$_3$)
170 160 150 140 130 120 110 100 90 80 70 60 50 40 30 20 10 0
ppm

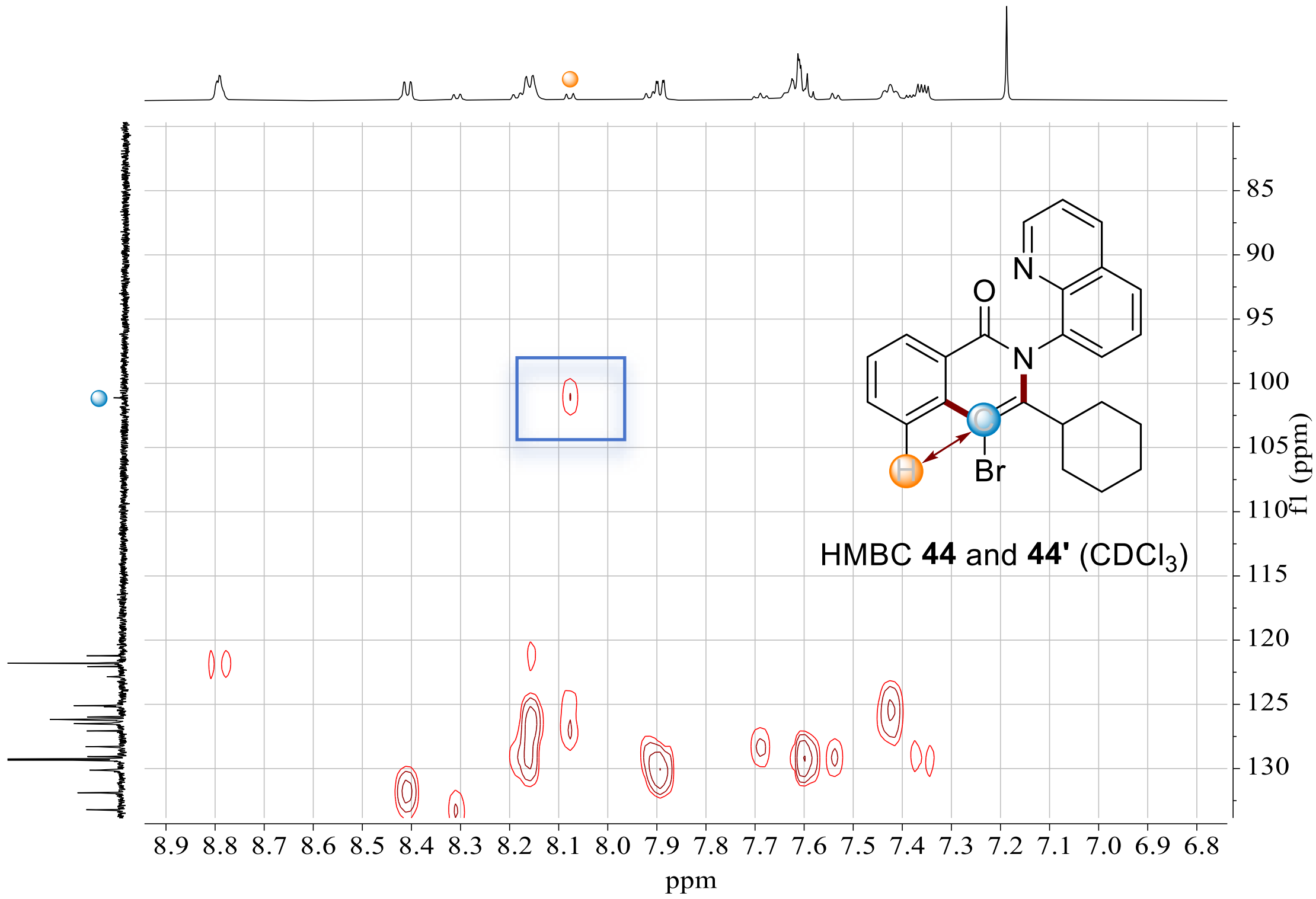
HMBC 44 and 44' (CDCl3)
ppm
f1 (ppm)

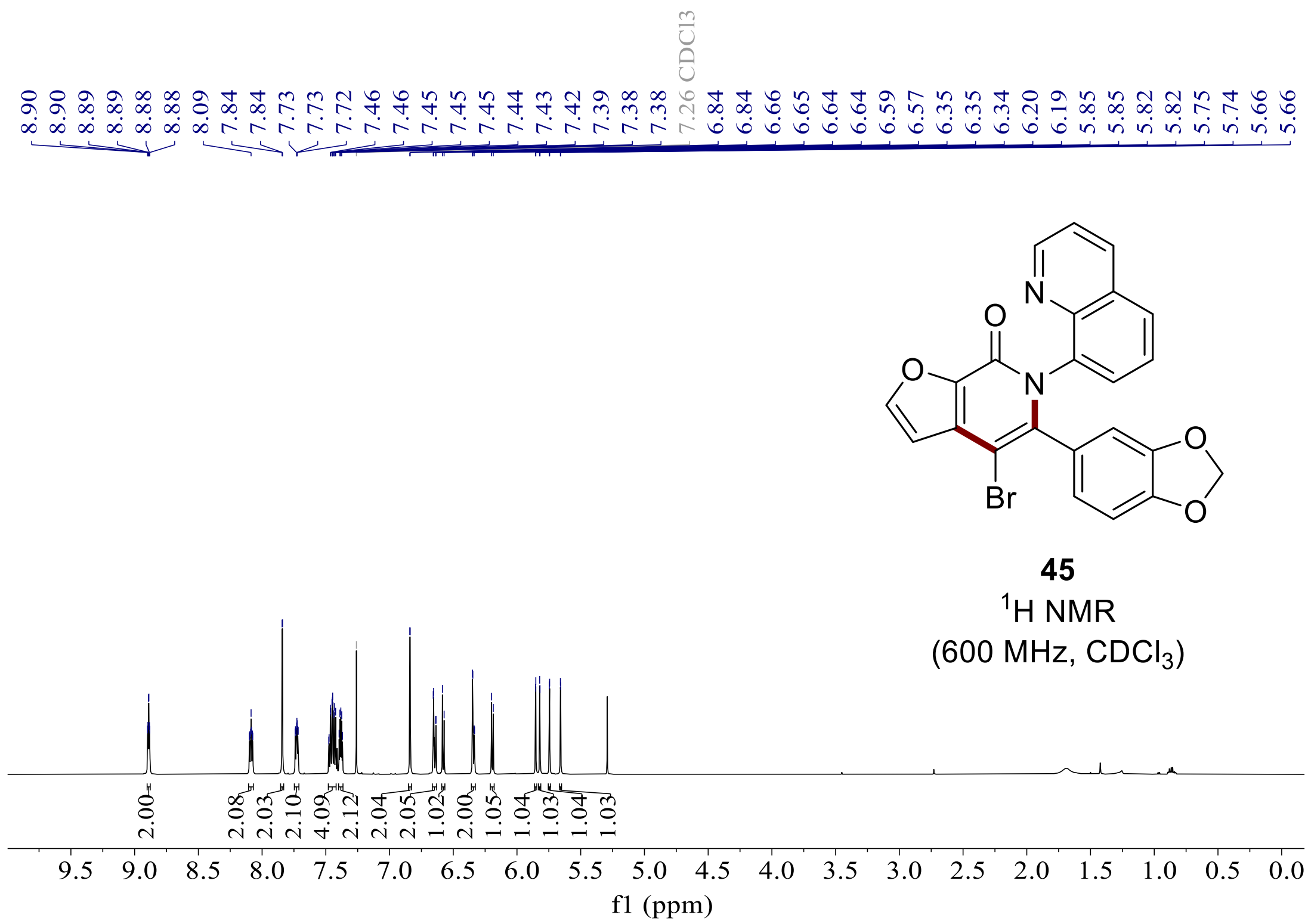
45
1H NMR
(600 MHz, CDCl3)
f1 (ppm)

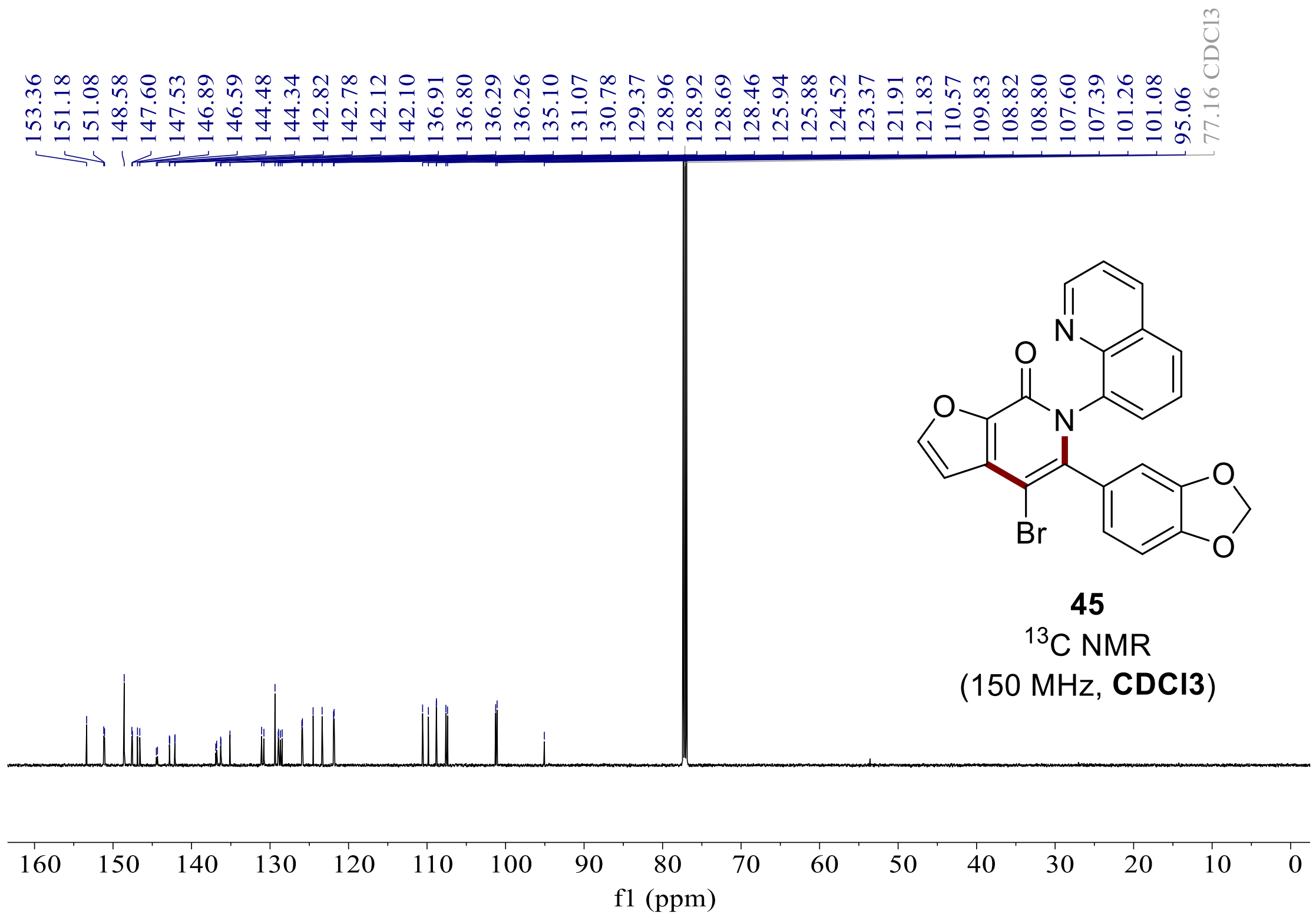
153.36
151.18
151.08
148.58
147.60
147.53
146.89
146.59
144.48
144.34
142.82
142.78
142.12
142.10
136.91
136.80
136.29
136.26
135.10
131.07
130.78
129.37
128.96
128.92
128.69
128.46
125.94
125.88
124.52
123.37
121.91
121.83
110.57
109.83
108.82
108.80
107.60
107.39
101.26
101.08
95.06
77.16 CDCl3
N
O
O
N
O
O
Br
45
^{13}C NMR
(150 MHz, CDCl3)
f1 (ppm)

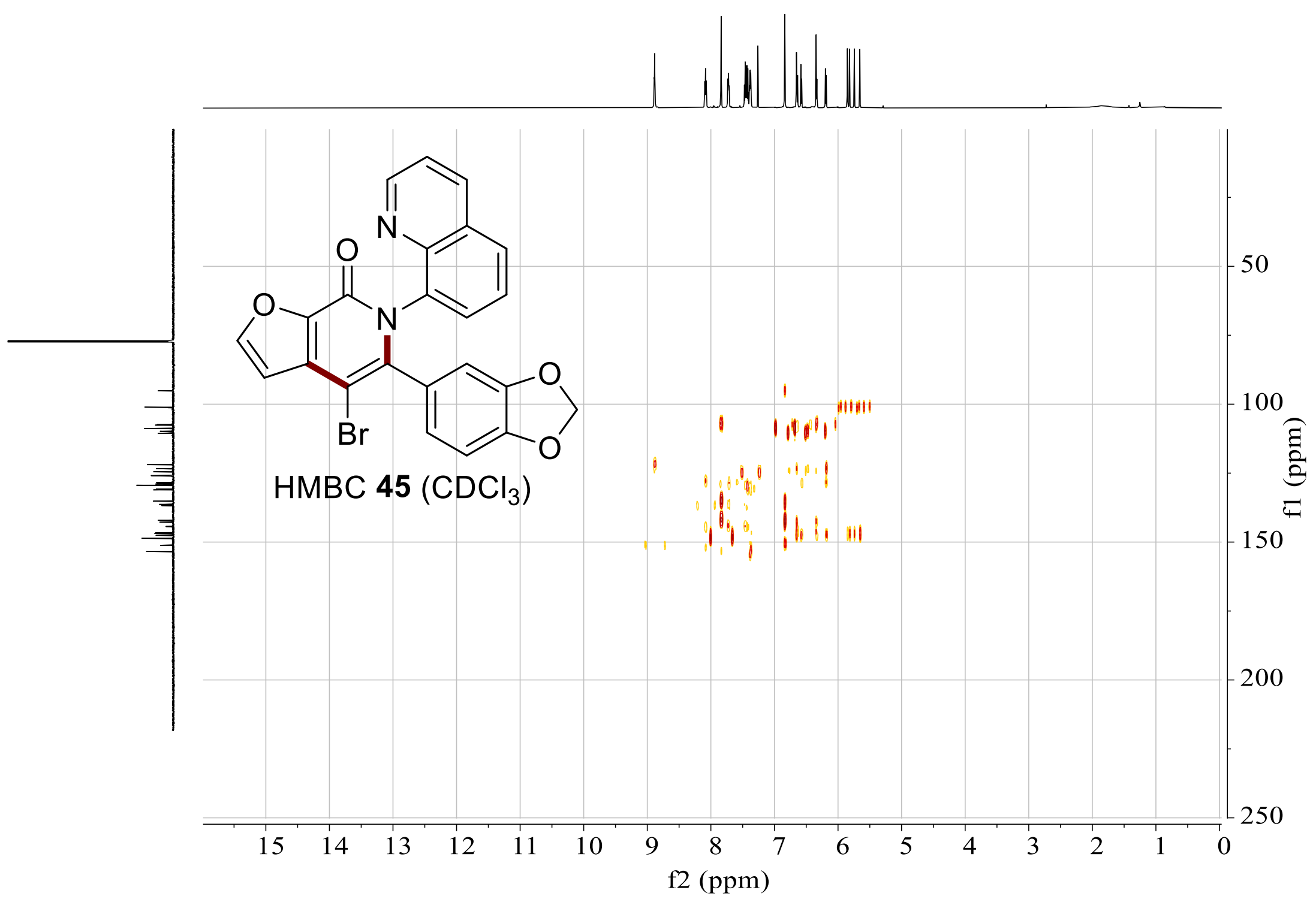
N
O
O
N
O
O
Br
HMBC 45 (CDCl$_3$)
f2 (ppm)
f1 (ppm)

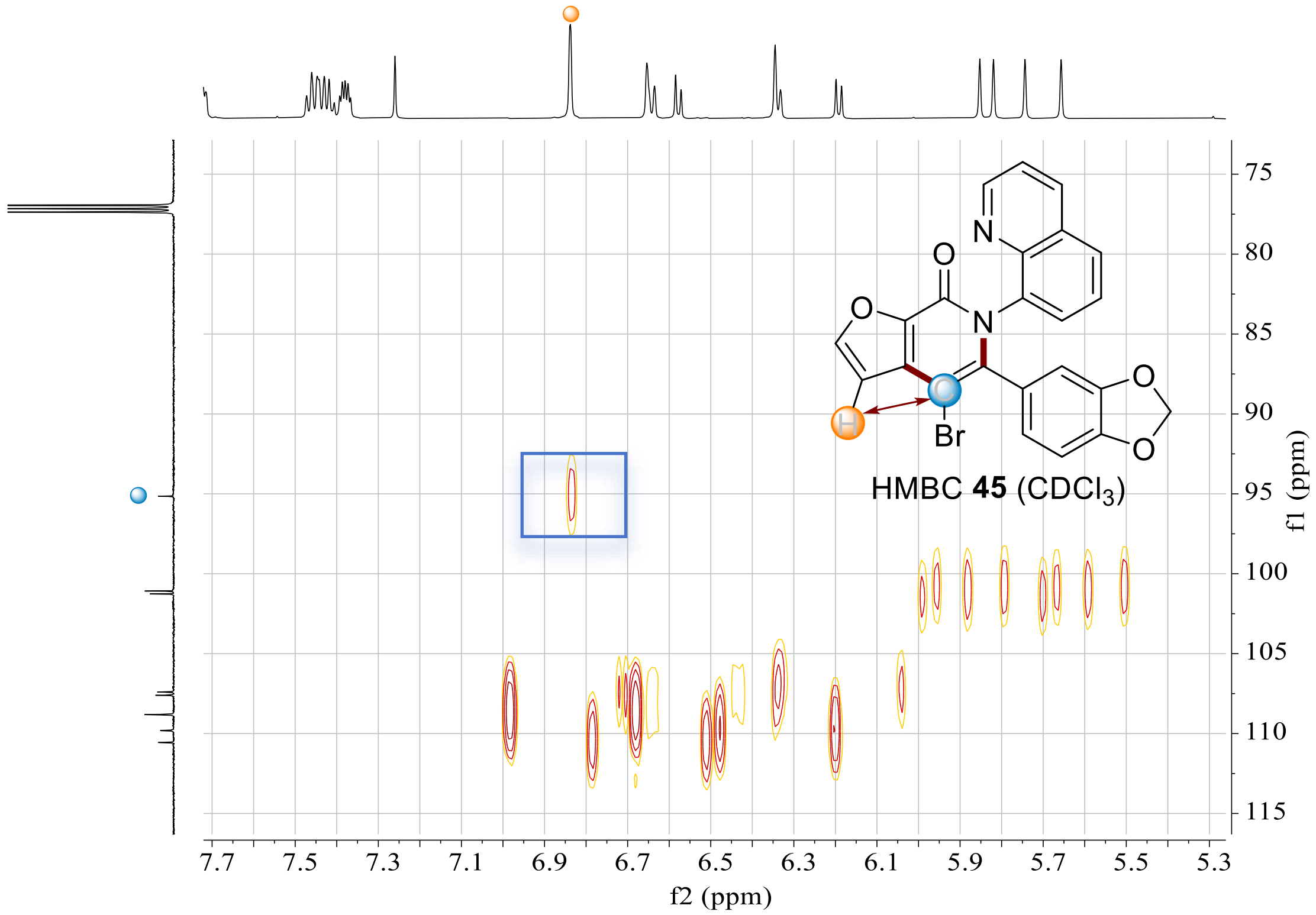

HMBC 45 (CDCl3)
f2 (ppm)
f1 (ppm)

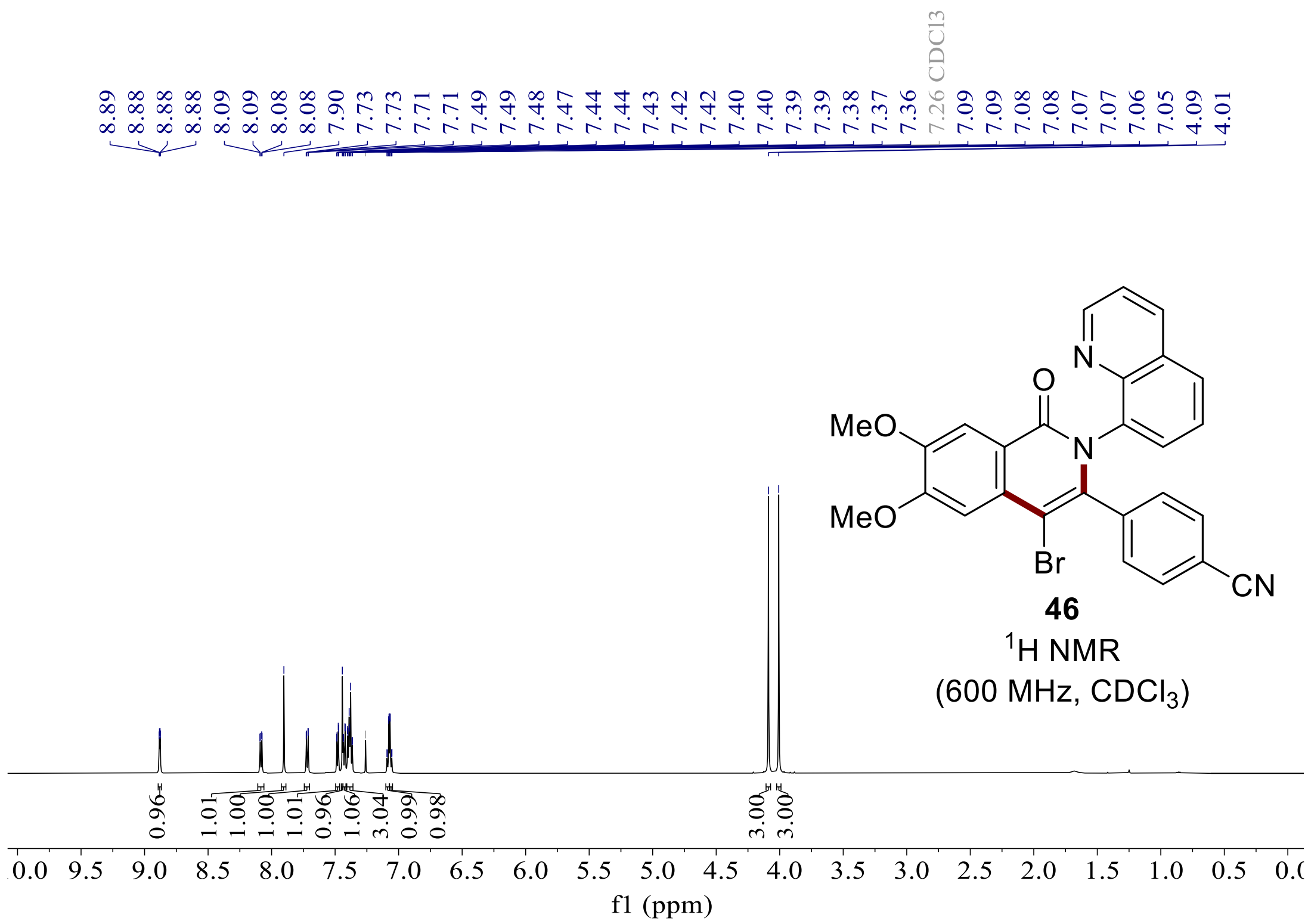

8.89 8.88 8.88 8.88 8.09 8.09 8.08 8.08 7.90 7.73 7.73 7.71 7.71 7.49 7.49 7.48 7.47 7.44 7.44 7.43 7.42 7.42 7.40 7.40 7.39 7.39 7.38 7.37 7.36 7.26 CDCl3 7.09 7.09 7.08 7.08 7.07 7.07 7.06 7.05 4.09 4.01
46
1H NMR
(600 MHz, CDCl3)
0.96 1.01 1.00 1.00 1.01 0.96 1.06 3.04 0.99 0.98 3.00 3.00
f1 (ppm)

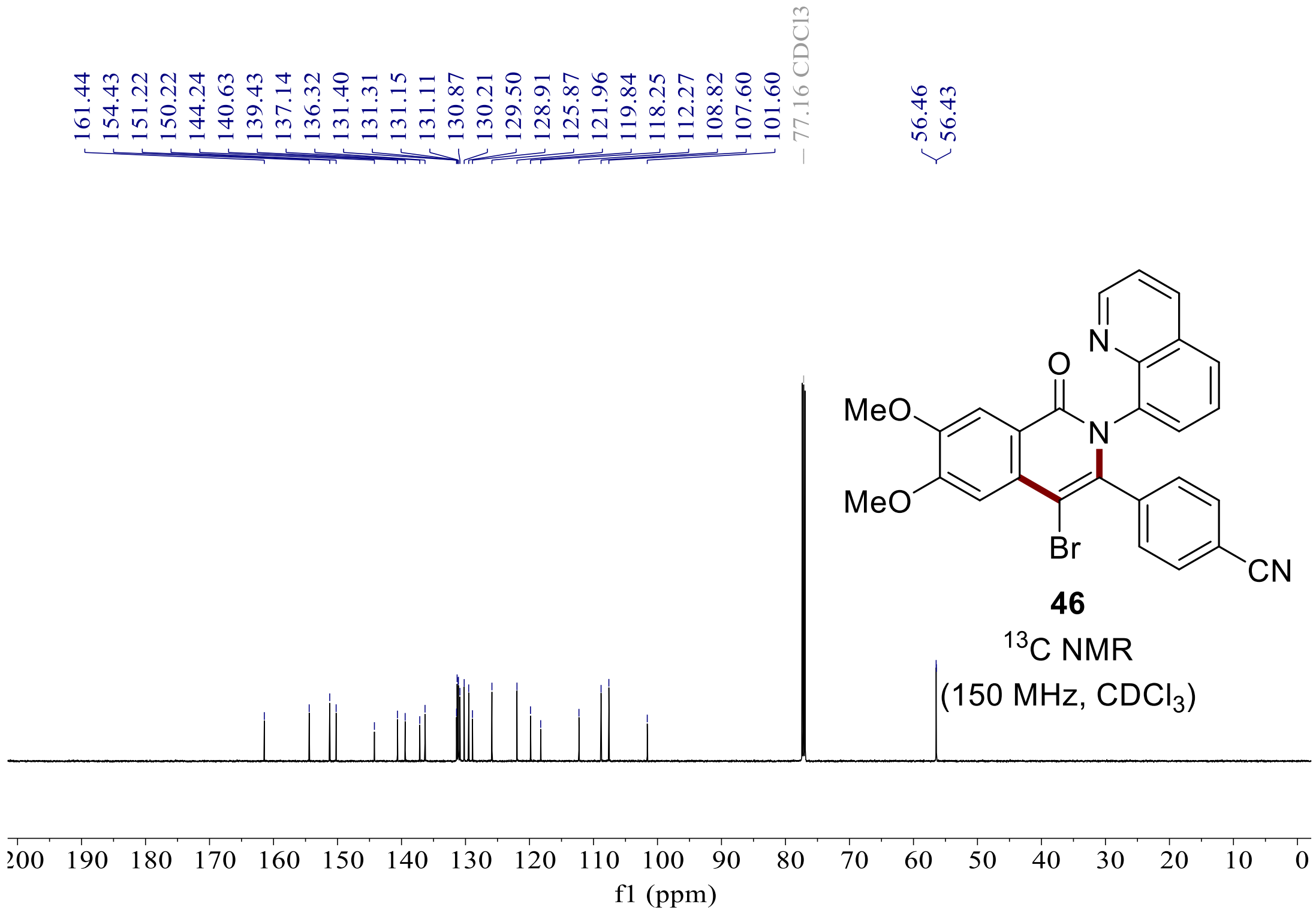
161.44
154.43
151.22
150.22
144.24
140.63
139.43
137.14
136.32
131.40
131.31
131.15
131.11
130.87
130.21
129.50
128.91
125.87
121.96
119.84
118.25
112.27
108.82
107.60
101.60
77.16 CDCl3
56.46
56.43
MeO
MeO
O
N
N
Br
CN
46
13C NMR
(150 MHz, CDCl3)
f1 (ppm)

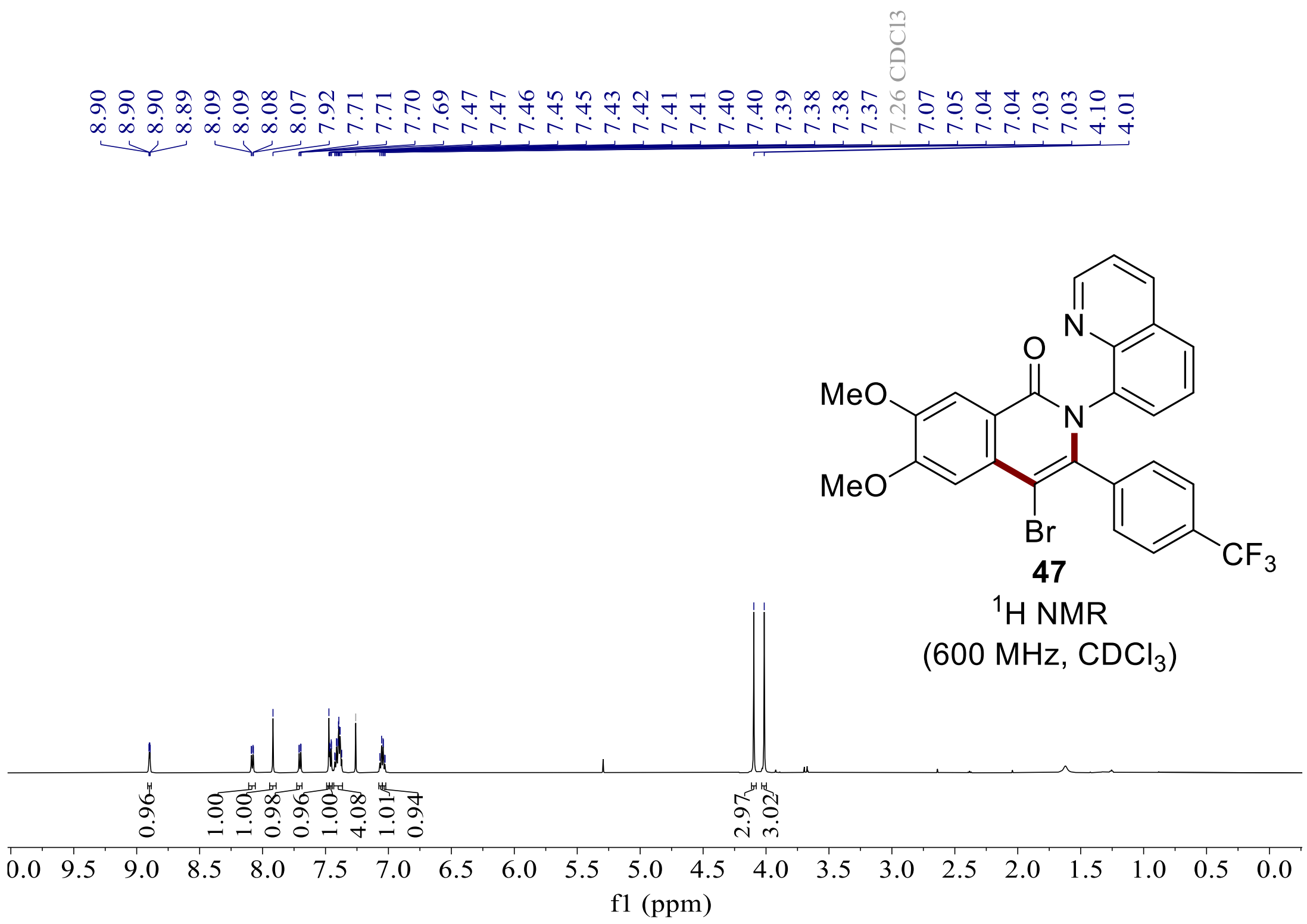
8.90
8.90
8.90
8.89
8.09
8.09
8.08
8.07
7.92
7.71
7.71
7.70
7.69
7.47
7.47
7.46
7.45
7.45
7.43
7.42
7.41
7.41
7.40
7.40
7.39
7.38
7.38
7.37
7.26 CDCl3
7.07
7.05
7.04
7.04
7.03
7.03
4.10
4.01
MeO
MeO
O
N
N
Br
CF3
47
1H NMR
(600 MHz, CDCl3)
0.96
1.00
1.00
0.98
0.96
1.00
4.08
1.01
0.94
2.97
3.02
f1 (ppm)

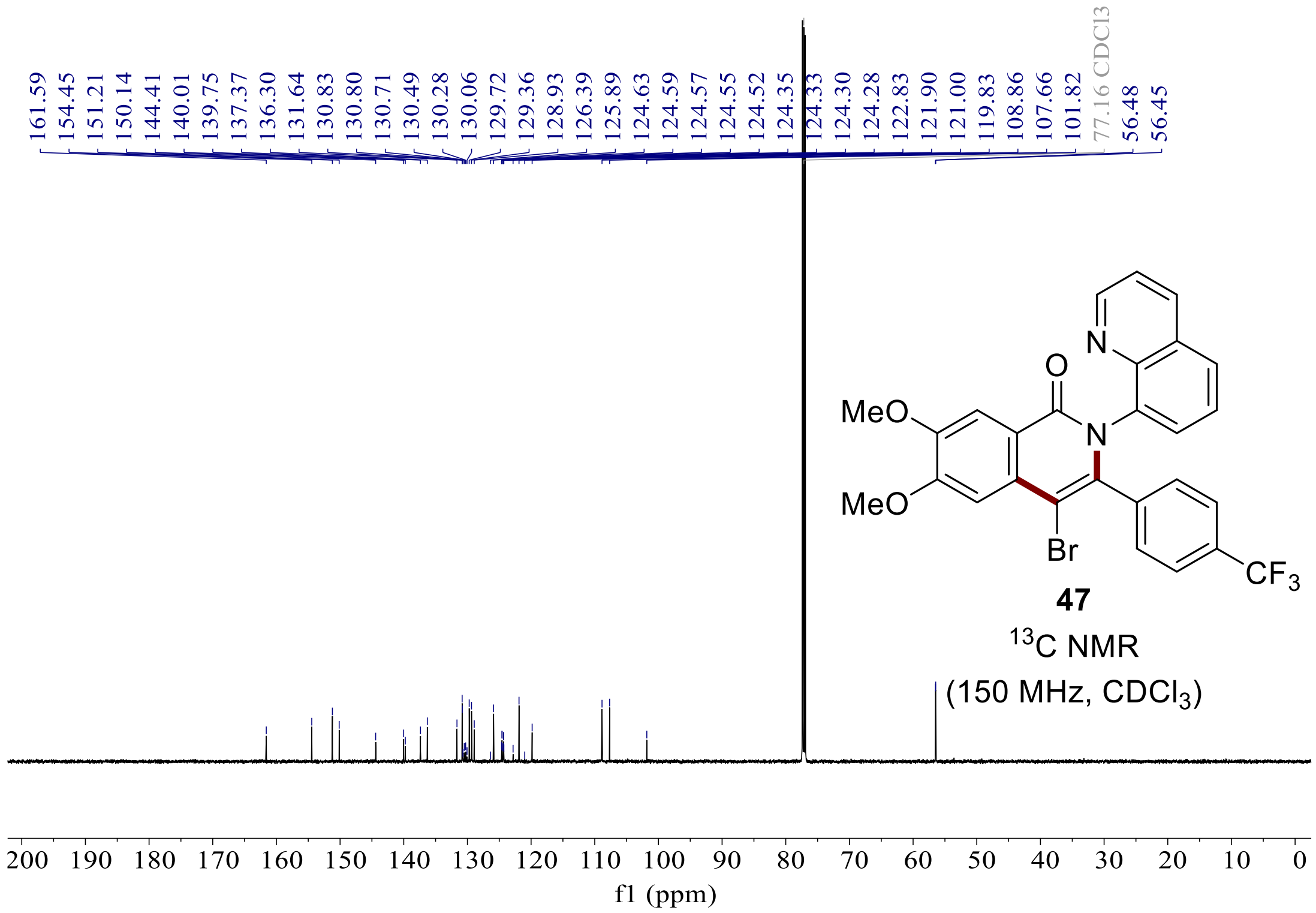

47

^{13}C NMR

(150 MHz, $CDCl_3$)

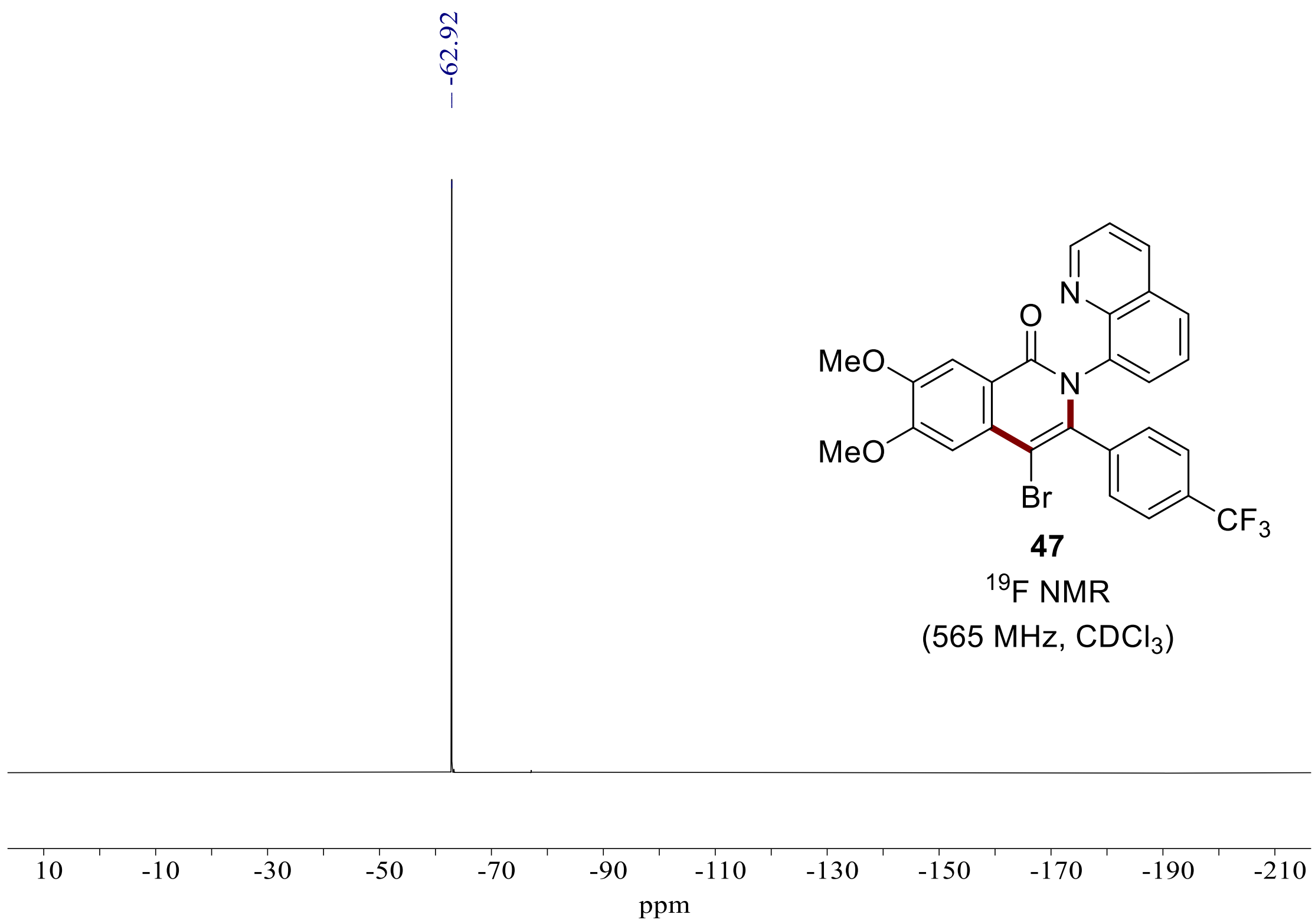

47

^{19}F NMR

(565 MHz, $CDCl_3$)

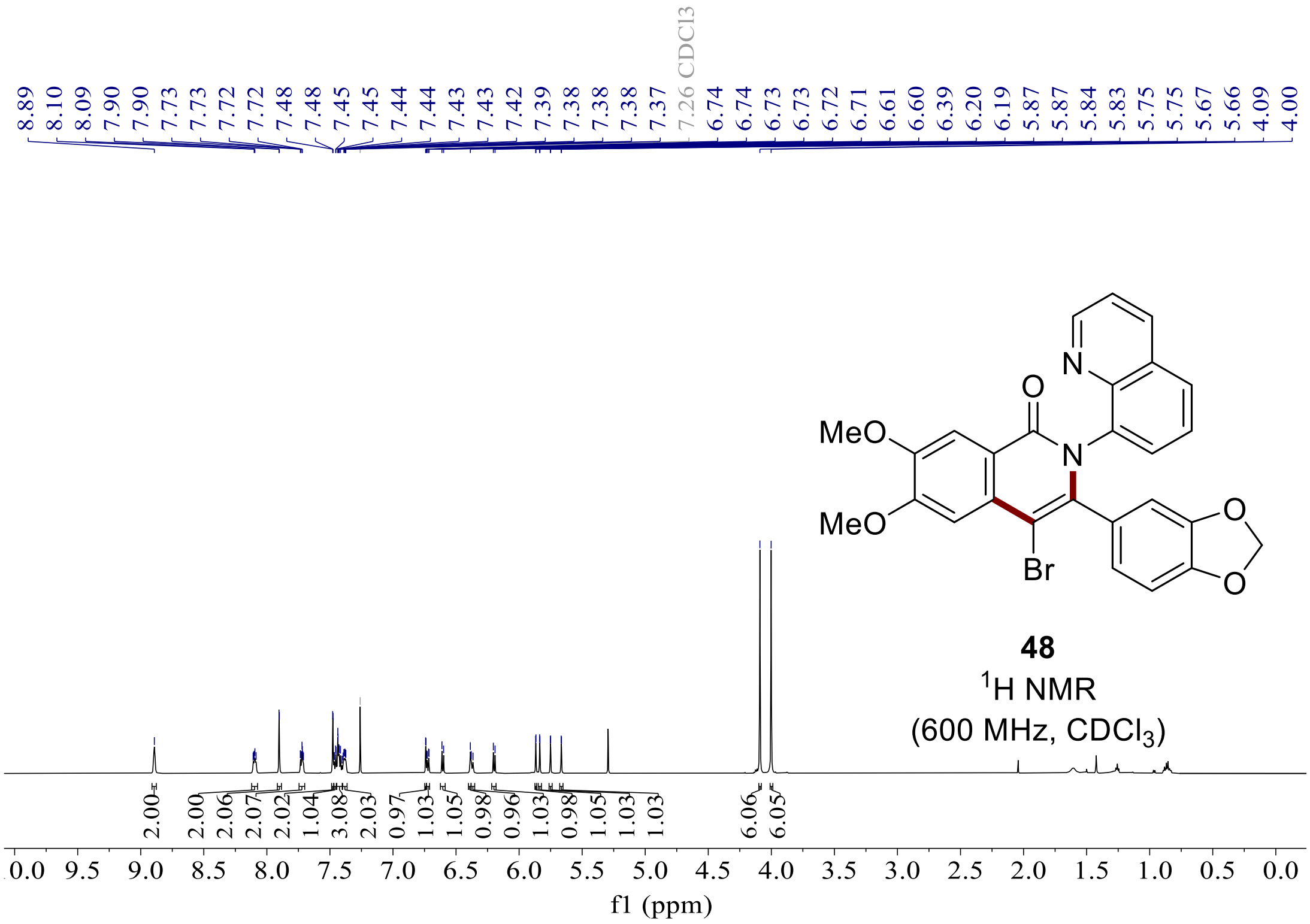
8.89 8.10 8.09 7.90 7.90 7.73 7.73 7.72 7.72 7.48 7.48 7.45 7.45 7.44 7.44 7.43 7.43 7.42 7.39 7.38 7.38 7.38 7.37 7.26 CDCl3 6.74 6.74 6.73 6.73 6.72 6.71 6.61 6.60 6.39 6.20 6.19 5.87 5.87 5.84 5.83 5.75 5.75 5.67 5.66 4.09 4.00
MeO
MeO
O
N
N
O
O
Br
48
^{1}H NMR
(600 MHz, $CDCl_3$)
2.00 2.00 2.06 2.07 2.02 1.04 3.08 2.03 0.97 1.03 1.05 0.98 0.96 1.03 0.98 1.05 1.03 1.03 6.06 6.05
0.0 9.5 9.0 8.5 8.0 7.5 7.0 6.5 6.0 5.5 5.0 4.5 4.0 3.5 3.0 2.5 2.0 1.5 1.0 0.5 0.0
f1 (ppm)

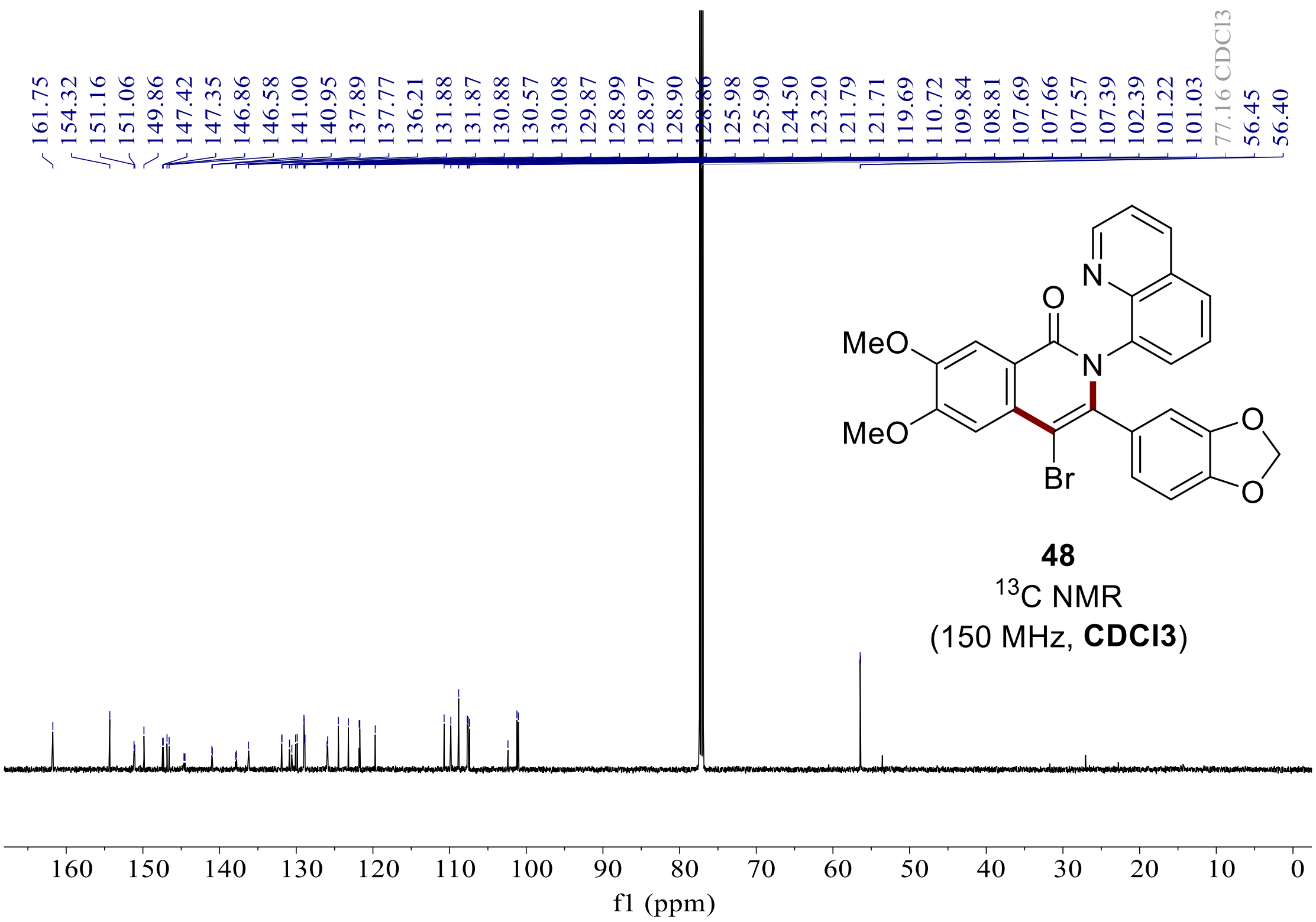
161.75 154.32 151.16 151.06 149.86 147.42 147.35 146.86 146.58 141.00 140.95 137.89 137.77 136.21 131.88 131.87 130.88 130.57 130.08 129.87 128.99 128.97 128.90 128.86 125.98 125.90 124.50 123.20 121.79 121.71 119.69 110.72 109.84 108.81 107.69 107.66 107.57 107.39 102.39 101.22 101.03 77.16 CDCl3 56.45 56.40
MeO
MeO
O
N
N
O
O
Br
48
^{13}C NMR
(150 MHz, **CDCl3**)
160 150 140 130 120 110 100 90 80 70 60 50 40 30 20 10 0
f1 (ppm)

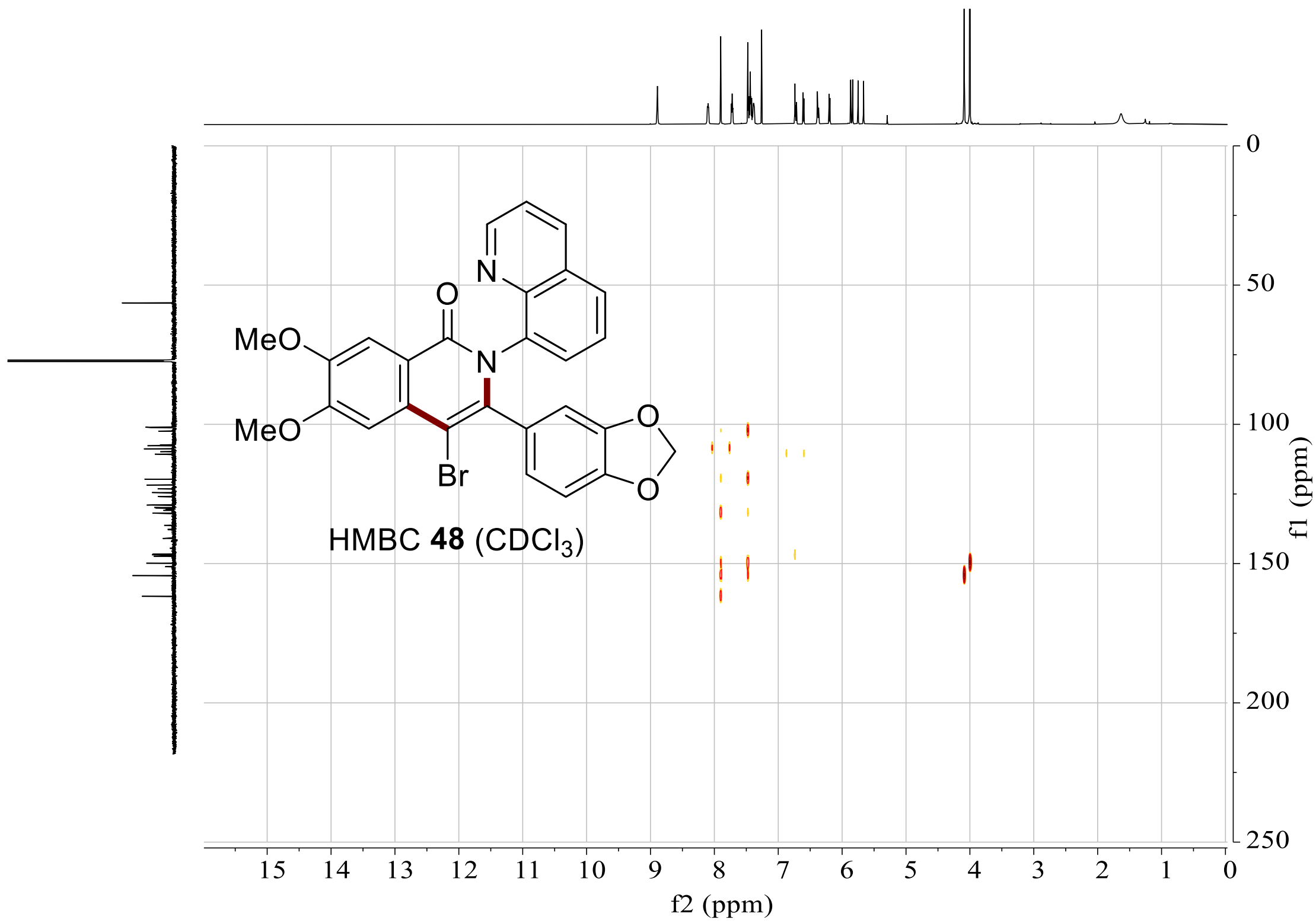
MeO
MeO
O
N
N
Br
O
O
HMBC 48 (CDCl3)
f1 (ppm)
f2 (ppm)

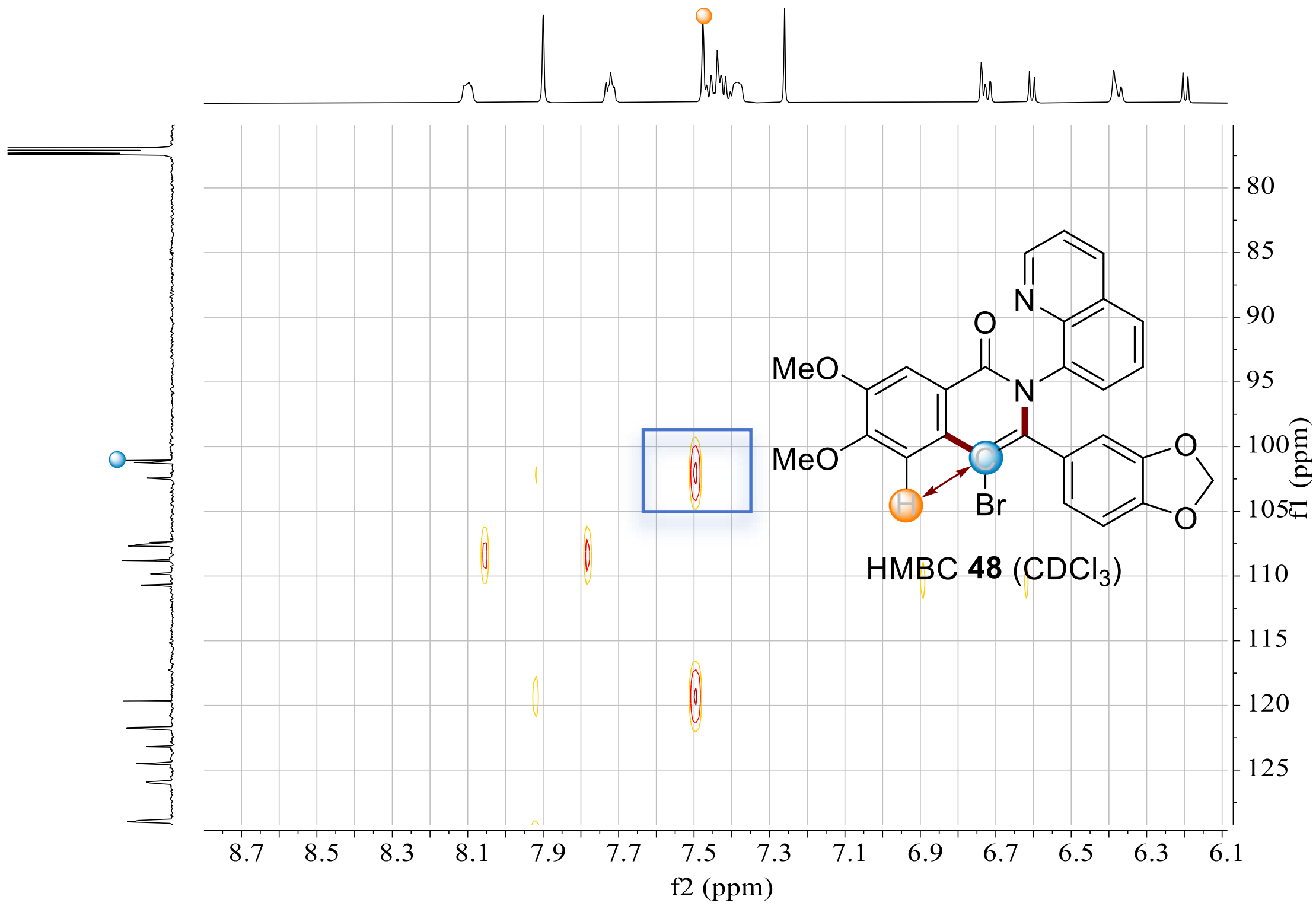
MeO
MeO
O
N
N
Br
O
O
H
C
HMBC 48 (CDCl3)
f1 (ppm)
f2 (ppm)

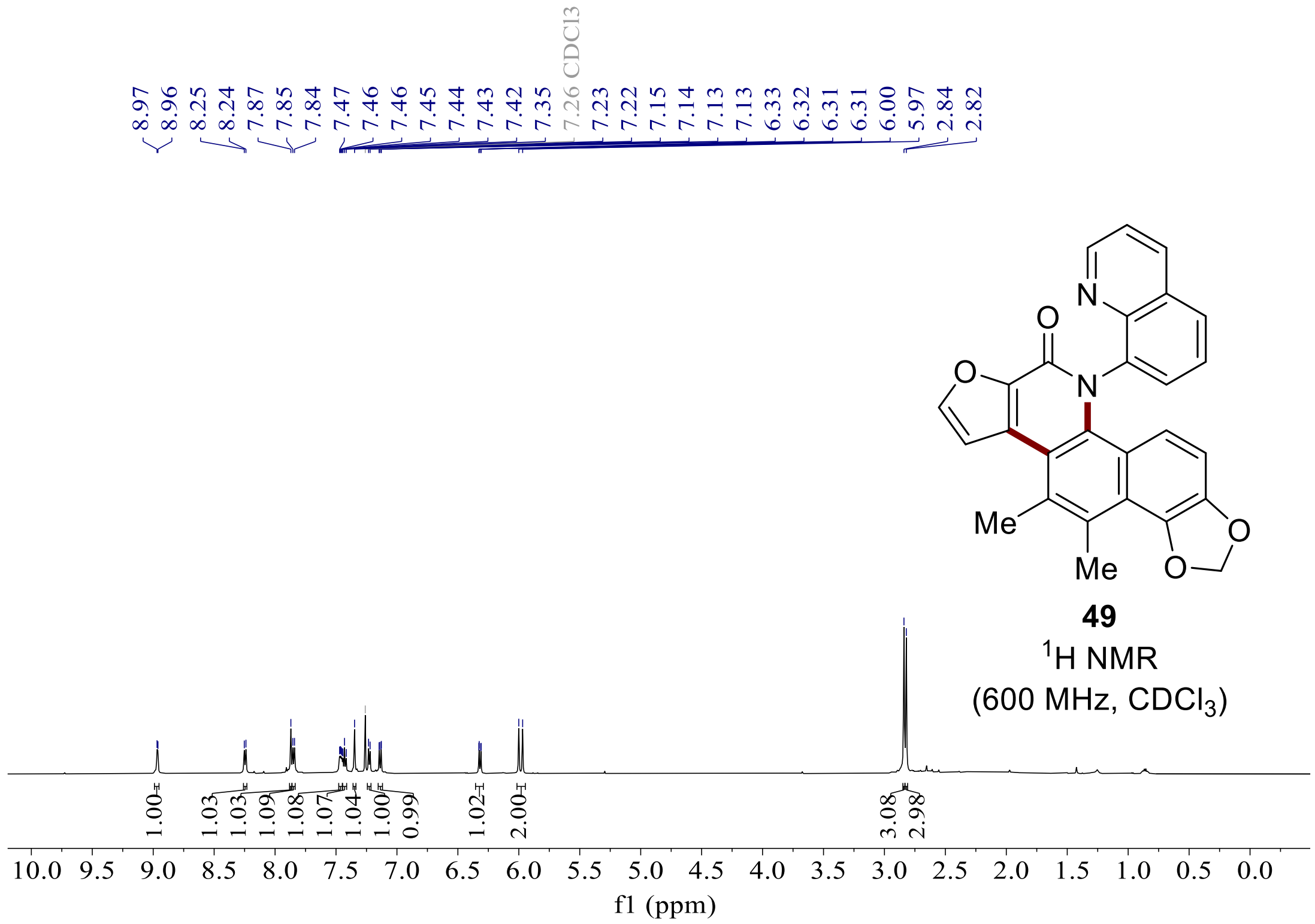
8.97
8.96
8.25
8.24
7.87
7.85
7.84
7.47
7.46
7.45
7.44
7.43
7.42
7.35
7.26 CDCl3
7.23
7.22
7.15
7.14
7.13
7.13
6.33
6.32
6.31
6.00
5.97
2.84
2.82
O
N
O
N
Me
Me
O
O
49
1H NMR
(600 MHz, CDCl3)
1.00
1.03
1.03
1.09
1.08
1.07
1.04
1.00
0.99
1.02
2.00
3.08
2.98
10.0 9.5 9.0 8.5 8.0 7.5 7.0 6.5 6.0 5.5 5.0 4.5 4.0 3.5 3.0 2.5 2.0 1.5 1.0 0.5 0.0
f1 (ppm)

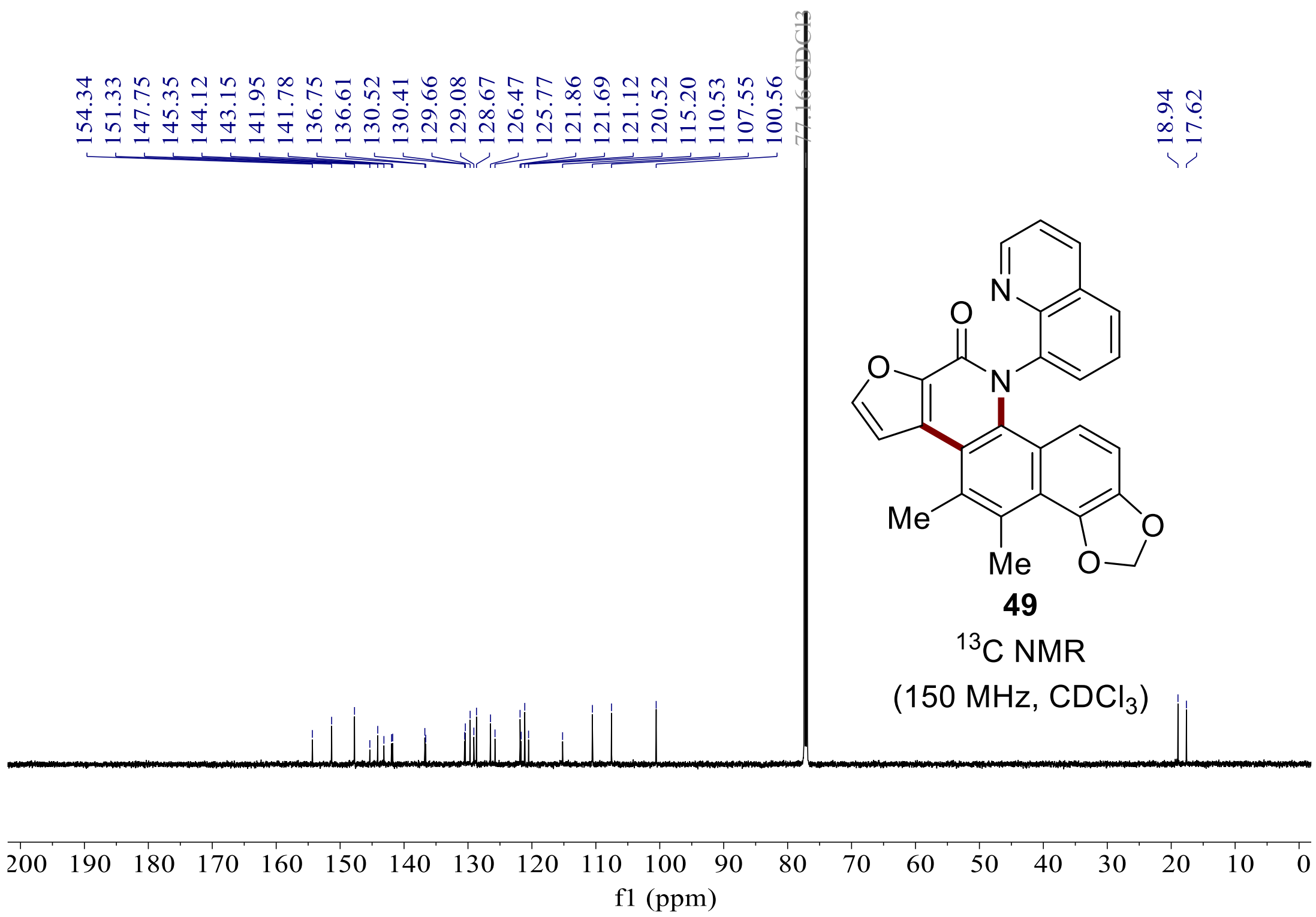
154.34
151.33
147.75
145.35
144.12
143.15
141.95
141.78
136.75
136.61
130.52
130.41
129.66
129.08
128.67
126.47
125.77
121.86
121.69
121.12
120.52
115.20
110.53
107.55
100.56
77.16 CDCl3
18.94
17.62
O
N
O
N
Me
Me
O
O
49
13C NMR
(150 MHz, CDCl3)
200 190 180 170 160 150 140 130 120 110 100 90 80 70 60 50 40 30 20 10 0
f1 (ppm)

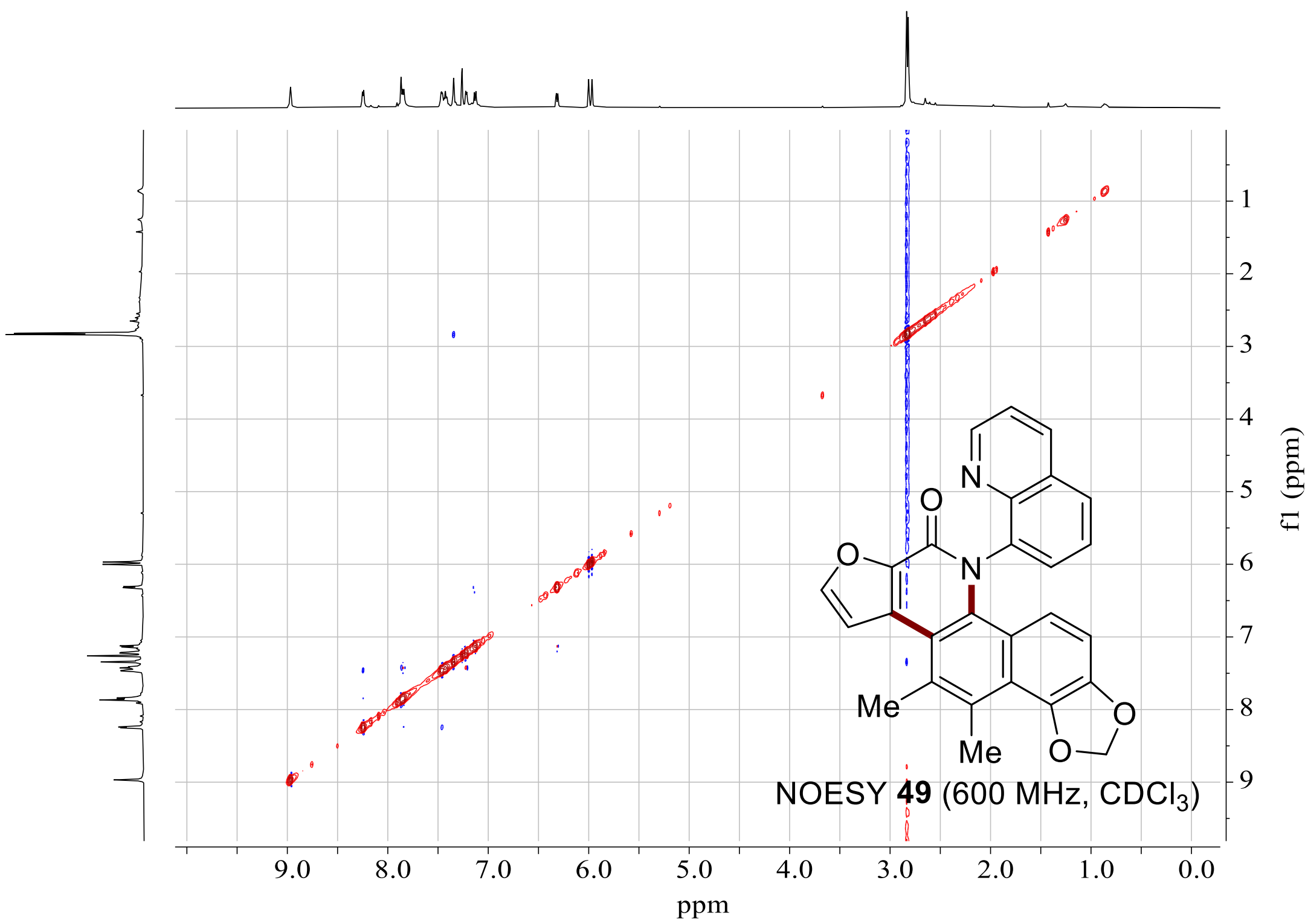

NOESY 49 (600 MHz, CDCl3)
ppm
f1 (ppm)

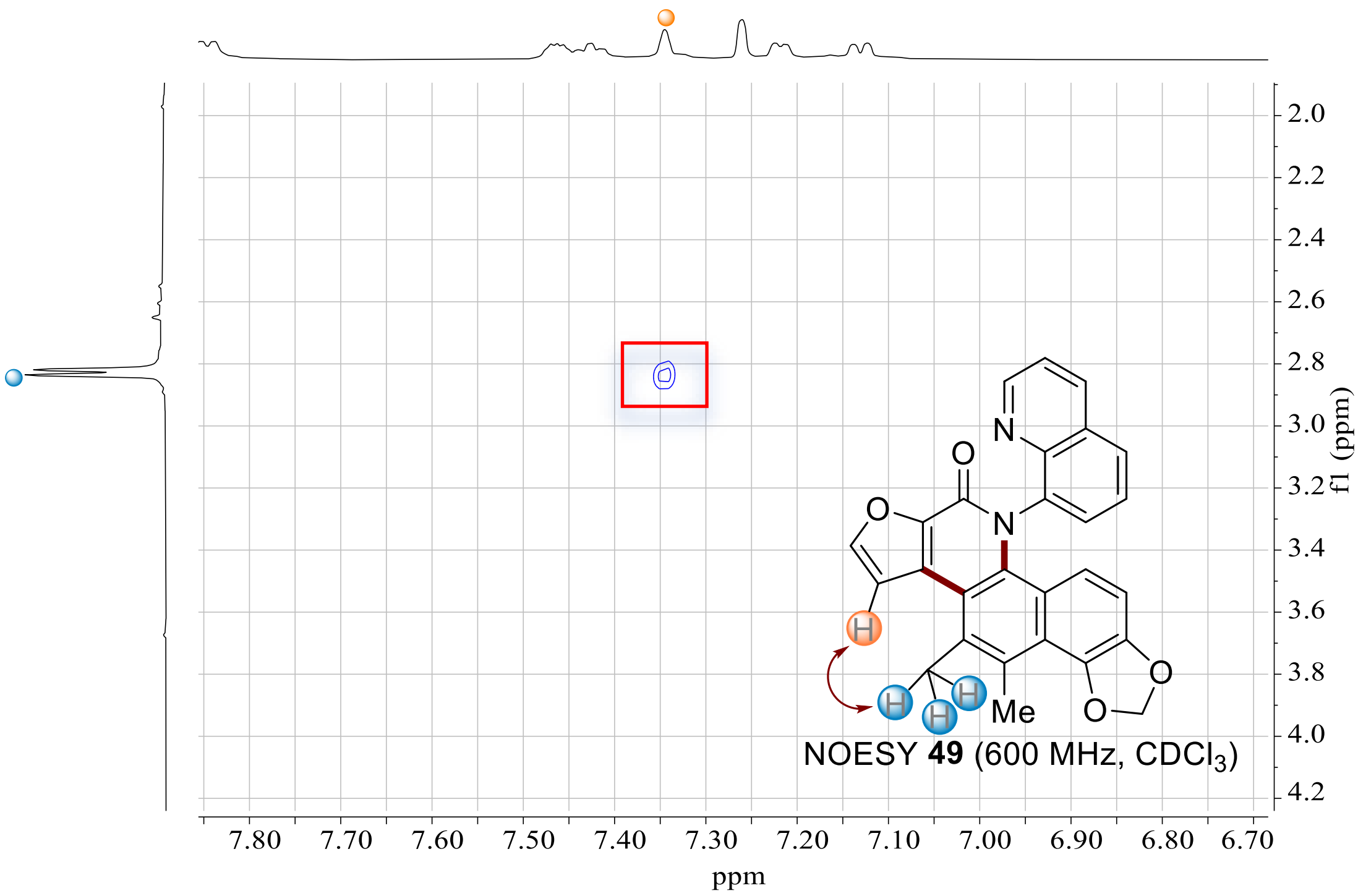

NOESY 49 (600 MHz, CDCl3)
ppm
f1 (ppm)

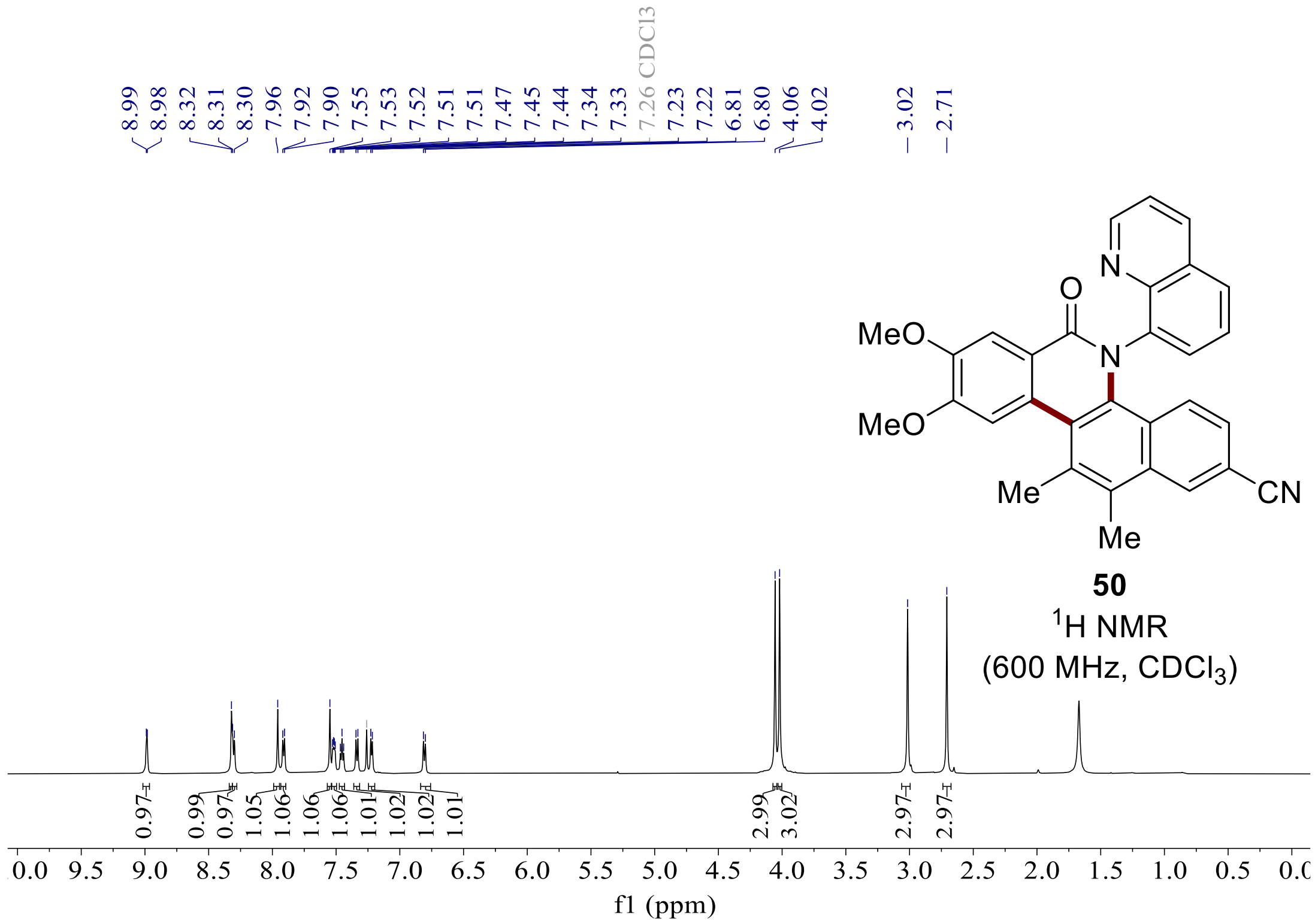
8.99
8.98
8.32
8.31
8.30
7.96
7.92
7.90
7.55
7.53
7.52
7.51
7.51
7.47
7.45
7.44
7.34
7.33
7.26 CDCl3
7.23
7.22
6.81
6.80
4.06
4.02
3.02
2.71
MeO
MeO
O
N
N
Me
Me
CN
50
1H NMR
(600 MHz, CDCl3)
0.97
0.99
0.97
1.05
1.06
1.06
1.06
1.01
1.02
1.02
1.01
2.99
3.02
2.97
2.97
f1 (ppm)

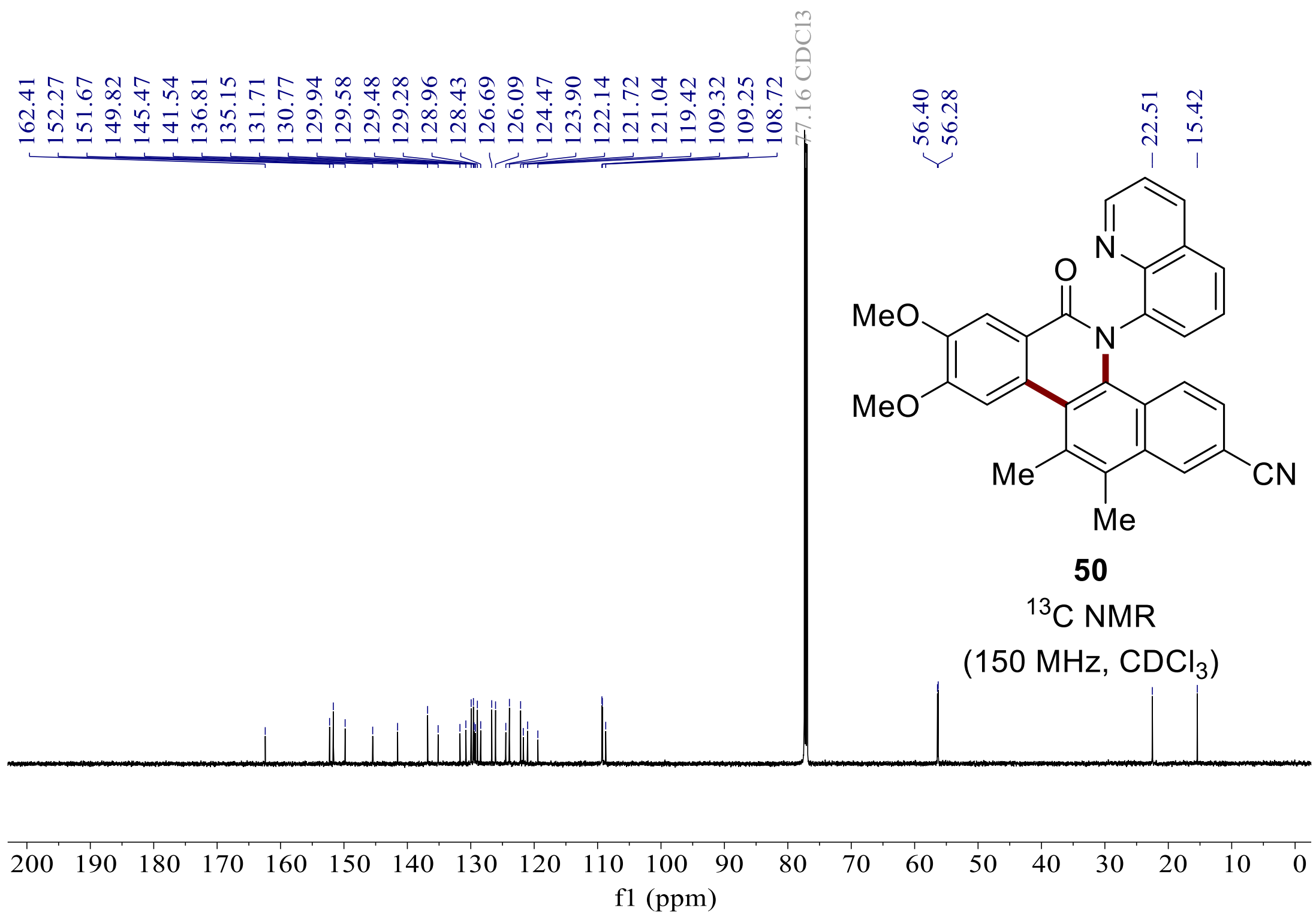
162.41
152.27
151.67
149.82
145.47
141.54
136.81
135.15
131.71
130.77
129.94
129.58
129.48
129.28
128.96
128.43
126.69
126.09
124.47
123.90
122.14
121.72
121.04
119.42
109.32
109.25
108.72
77.16 CDCl3
56.40
56.28
22.51
15.42
MeO
MeO
O
N
N
Me
Me
CN
50
13C NMR
(150 MHz, CDCl3)
f1 (ppm)

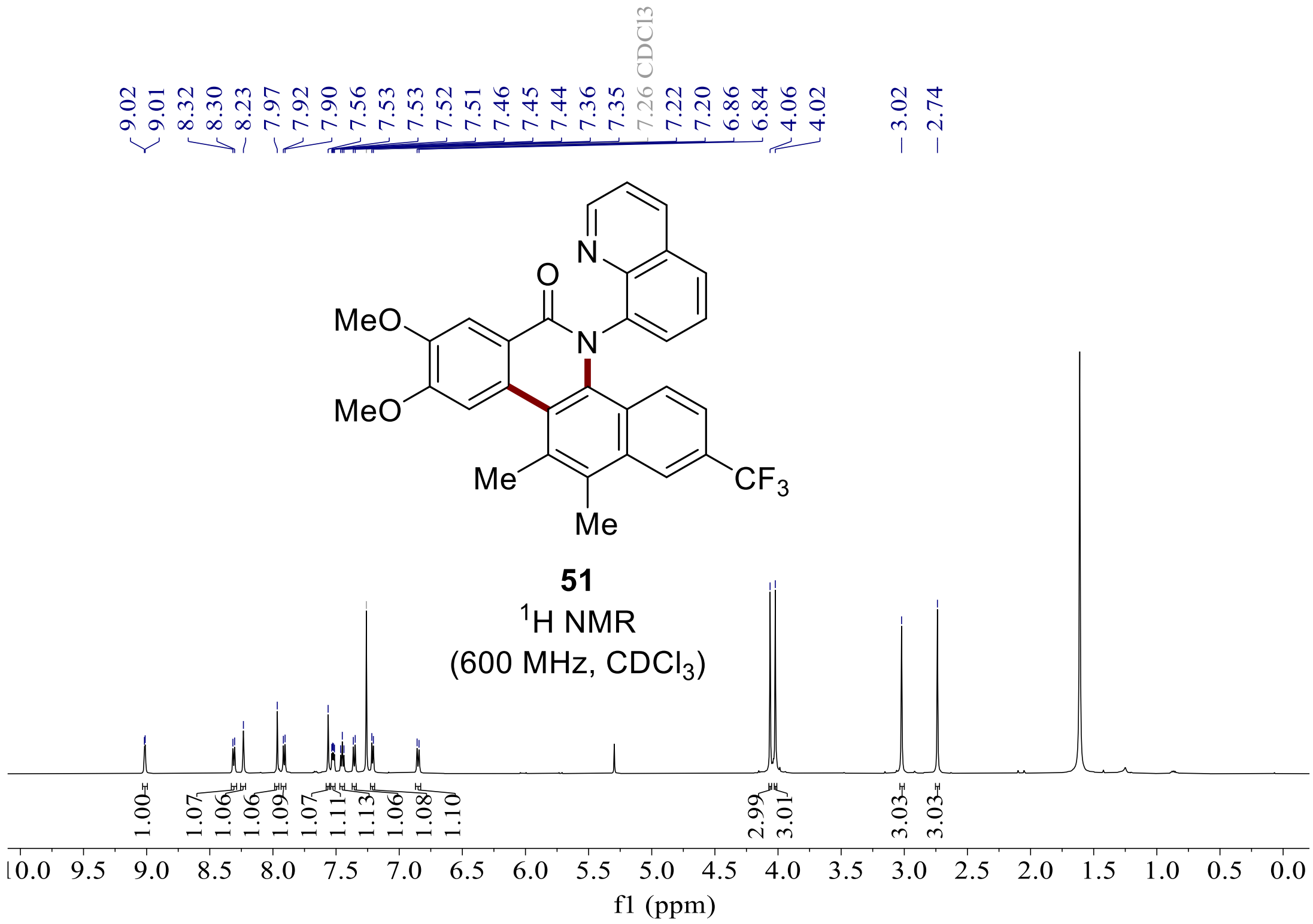
9.02
9.01
8.32
8.30
8.23
7.97
7.92
7.90
7.56
7.53
7.53
7.52
7.51
7.46
7.45
7.44
7.36
7.35
7.26 CDCl3
7.22
7.20
6.86
6.84
4.06
4.02
3.02
2.74
MeO
MeO
O
N
N
Me
Me
CF3
51
1H NMR
(600 MHz, CDCl3)
1.00
1.07
1.06
1.06
1.09
1.07
1.11
1.13
1.06
1.08
1.10
2.99
3.01
3.03
3.03
10.0 9.5 9.0 8.5 8.0 7.5 7.0 6.5 6.0 5.5 5.0 4.5 4.0 3.5 3.0 2.5 2.0 1.5 1.0 0.5 0.0
f1 (ppm)

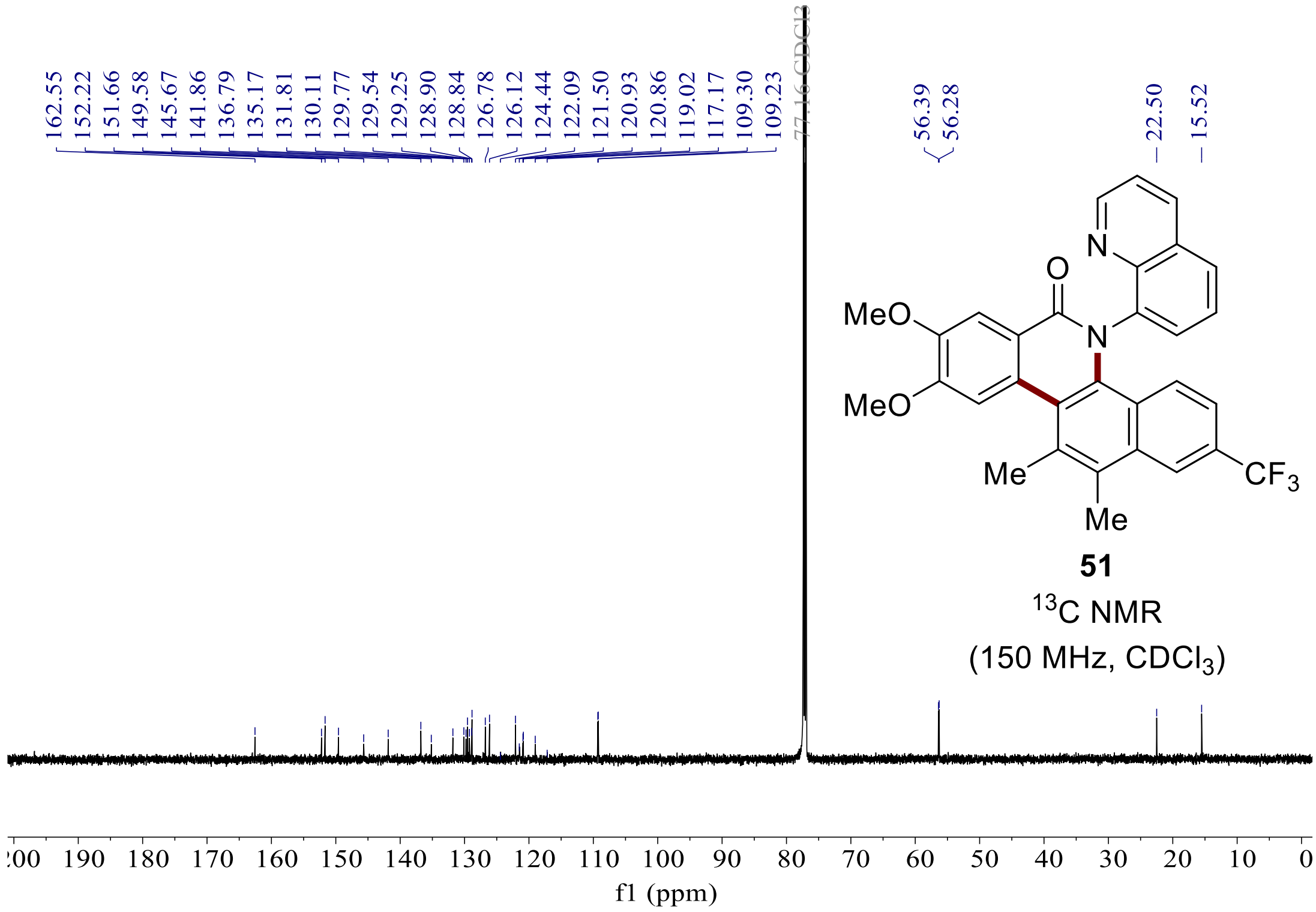
162.55
152.22
151.66
149.58
145.67
141.86
136.79
135.17
131.81
130.11
129.77
129.54
129.25
128.90
128.84
126.78
126.12
124.44
122.09
121.50
120.93
120.86
119.02
117.17
109.30
109.23
77.16 CDCl3
56.39
56.28
22.50
15.52
MeO
MeO
O
N
N
Me
Me
CF3
51
13C NMR
(150 MHz, CDCl3)
200 190 180 170 160 150 140 130 120 110 100 90 80 70 60 50 40 30 20 10 0
f1 (ppm)

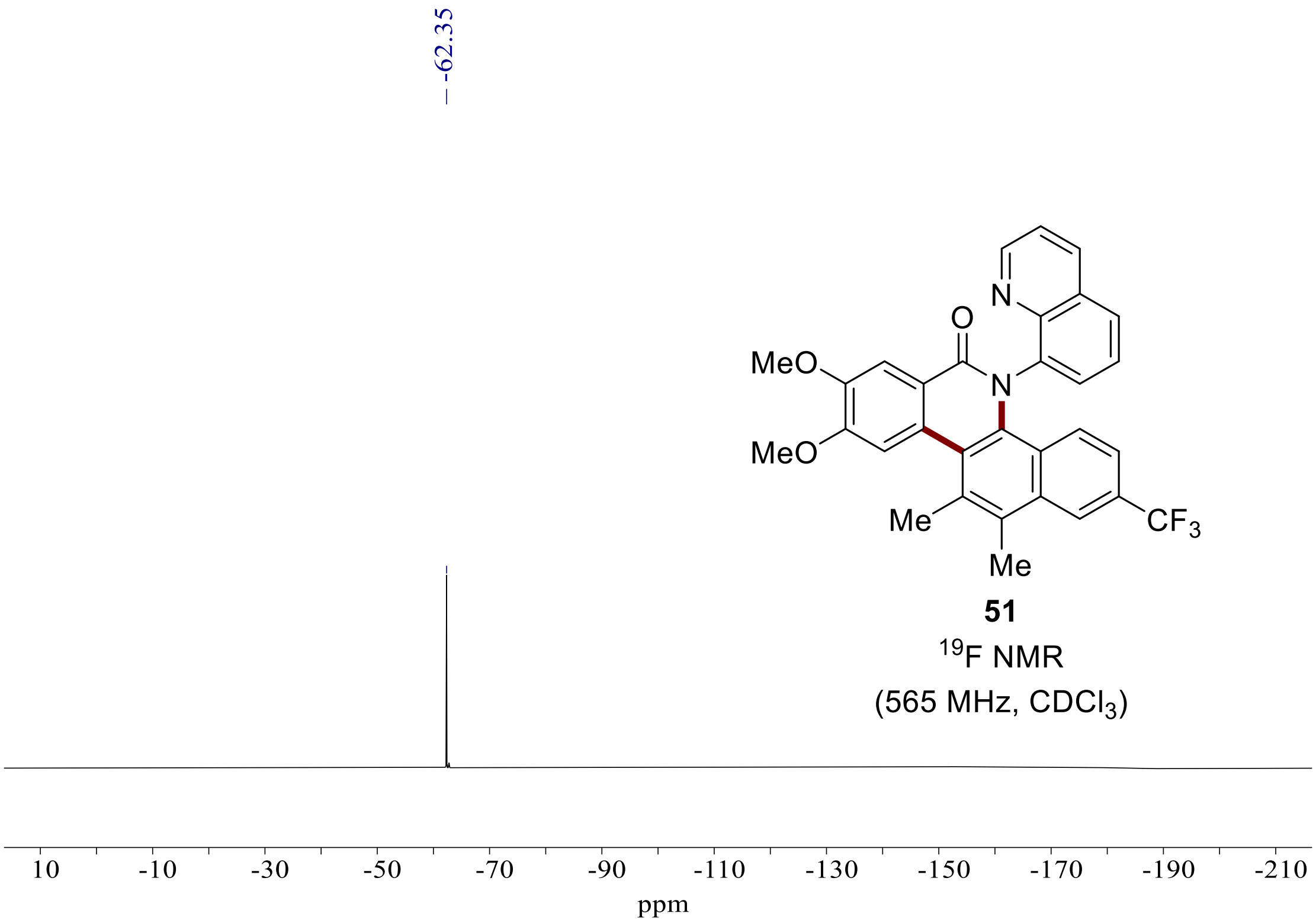
-62.35
MeO
MeO
O
N
N
Me
Me
CF3
51
19F NMR
(565 MHz, CDCl3)
10
-10
-30
-50
-70
-90
-110
-130
-150
-170
-190
-210
ppm

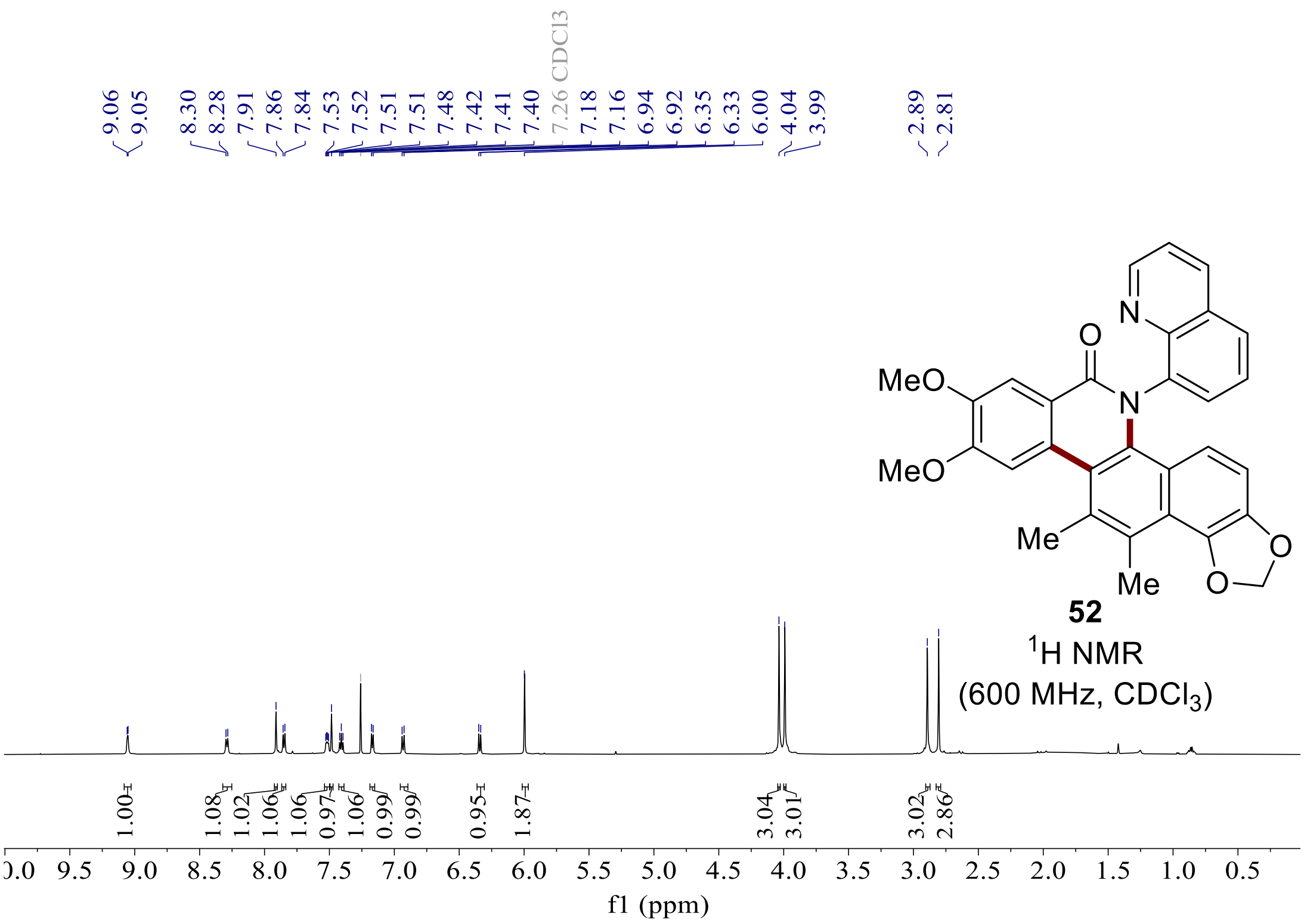
9.06
9.05
8.30
8.28
7.91
7.86
7.84
7.53
7.52
7.51
7.51
7.48
7.42
7.41
7.40
7.26 CDCl3
7.18
7.16
6.94
6.92
6.35
6.33
6.00
4.04
3.99
2.89
2.81
MeO
MeO
O
N
N
Me
Me
O
O
52
1H NMR
(600 MHz, CDCl3)
1.00
1.08
1.02
1.06
1.06
0.97
1.06
0.99
0.99
0.95
1.87
3.04
3.01
3.02
2.86
9.5
9.0
8.5
8.0
7.5
7.0
6.5
6.0
5.5
5.0
4.5
4.0
3.5
3.0
2.5
2.0
1.5
1.0
0.5
f1 (ppm)

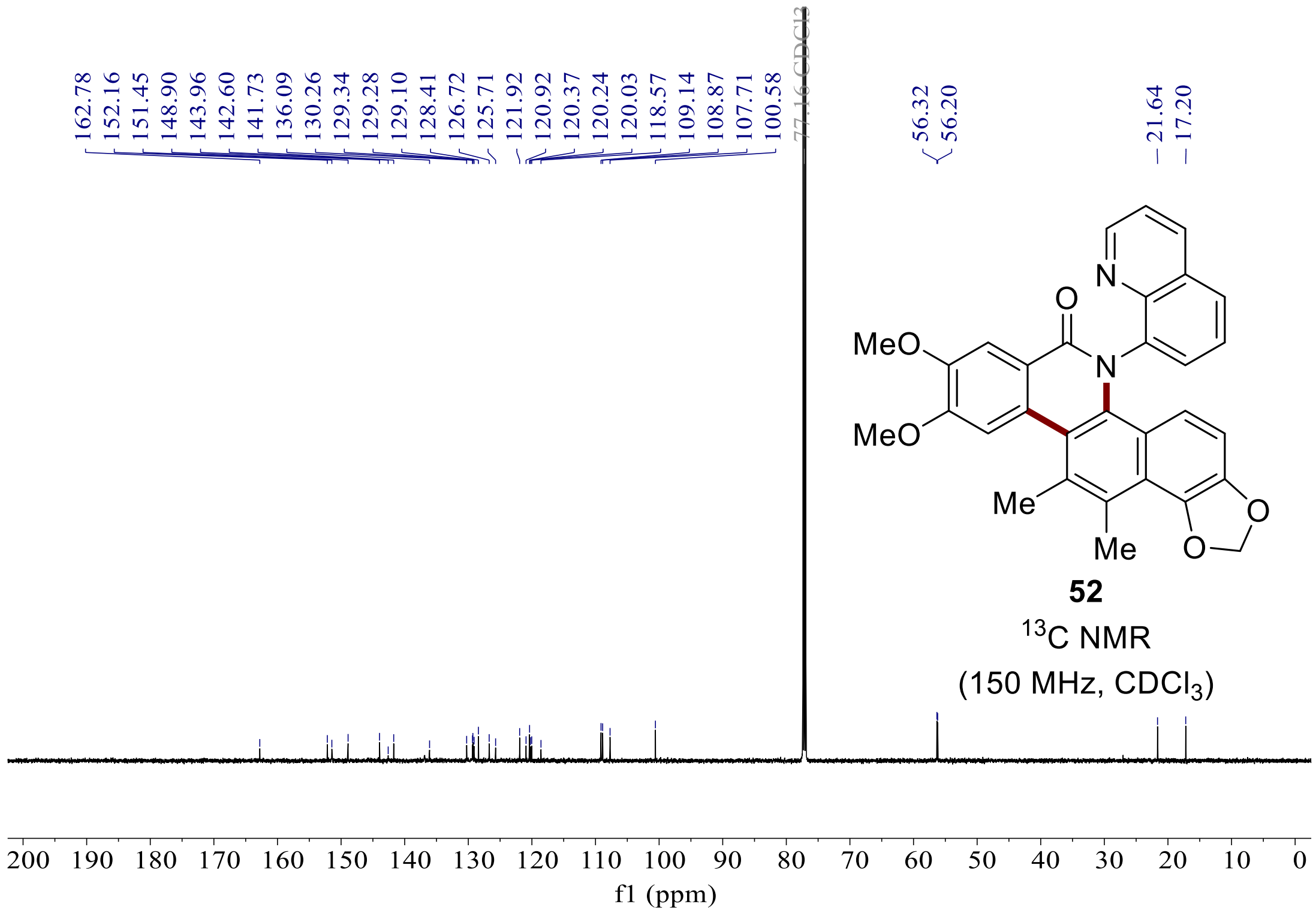
162.78
152.16
151.45
148.90
143.96
142.60
141.73
136.09
130.26
129.34
129.28
129.10
128.41
126.72
125.71
121.92
120.92
120.37
120.24
120.03
118.57
109.14
108.87
107.71
100.58
77.16 CDCl3
56.32
56.20
21.64
17.20
MeO
MeO
O
N
N
Me
Me
O
O
52
^{13}C NMR
(150 MHz, $CDCl_3$)
f1 (ppm)

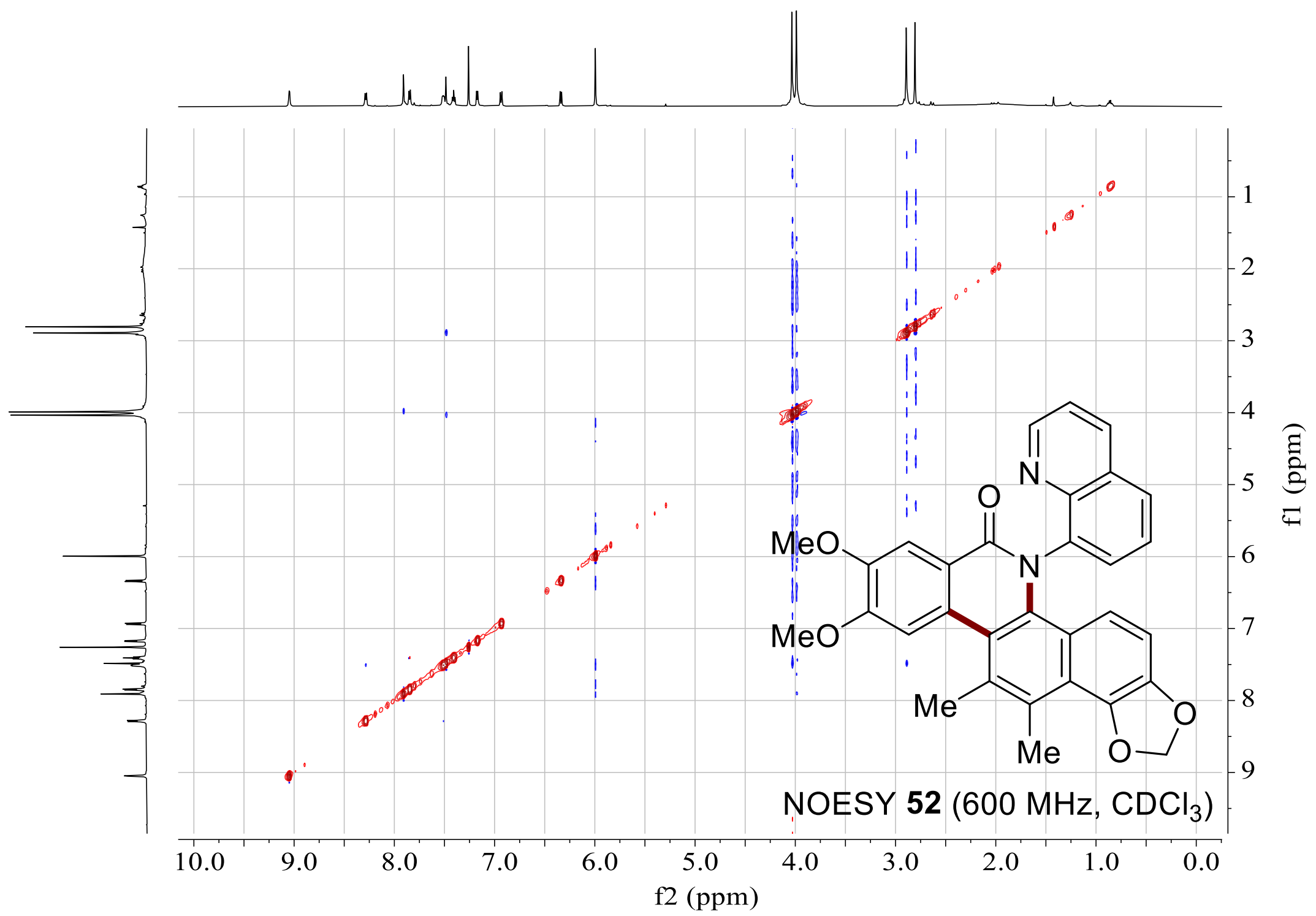
MeO
MeO
O
N
N
Me
Me
O
O
NOESY 52 (600 MHz, $CDCl_3$)
f2 (ppm)
f1 (ppm)

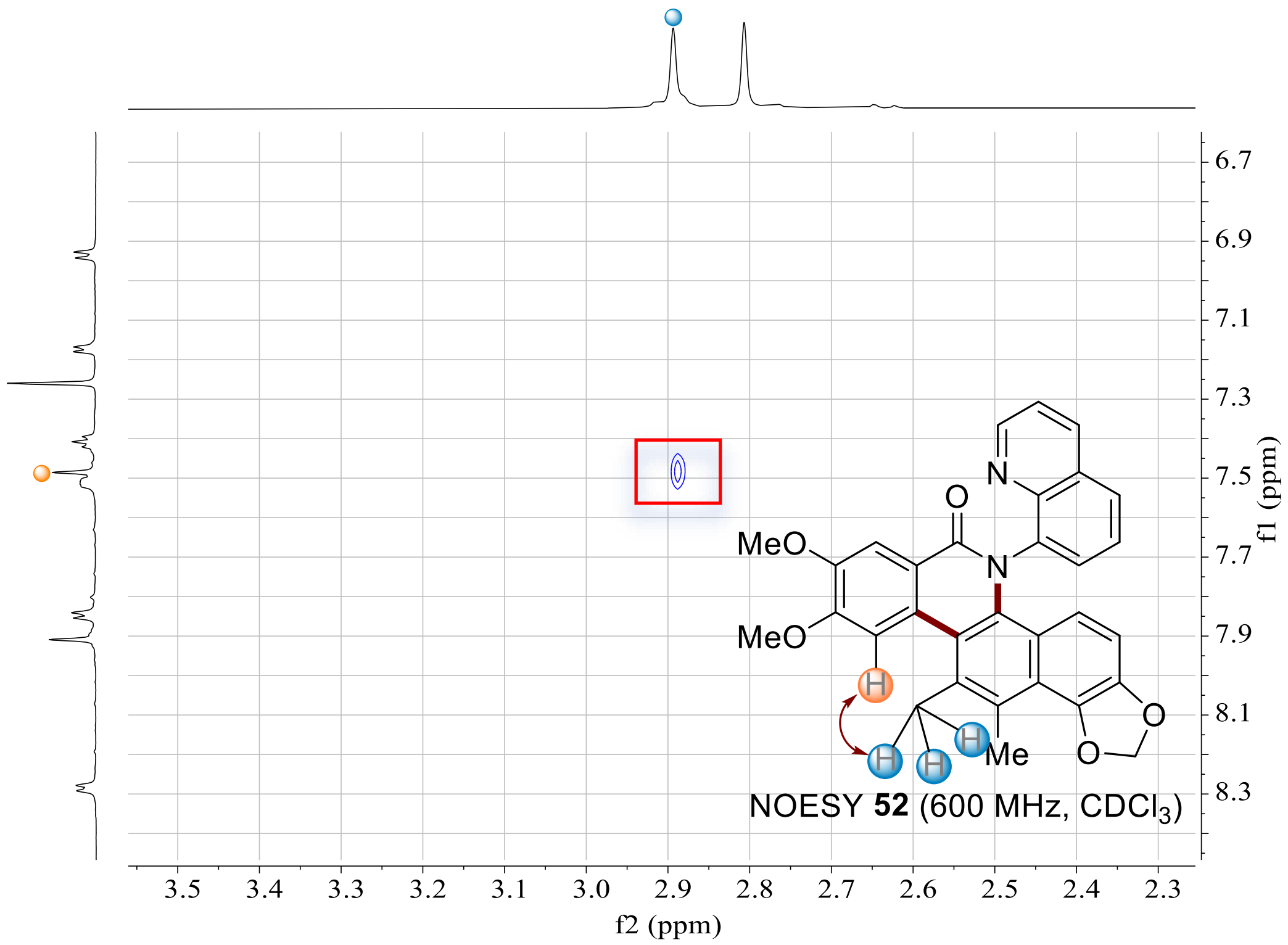
MeO
MeO
O
N
N
H
H
H
H
Me
O
O
NOESY 52 (600 MHz, CDCl3)
f2 (ppm)
f1 (ppm)

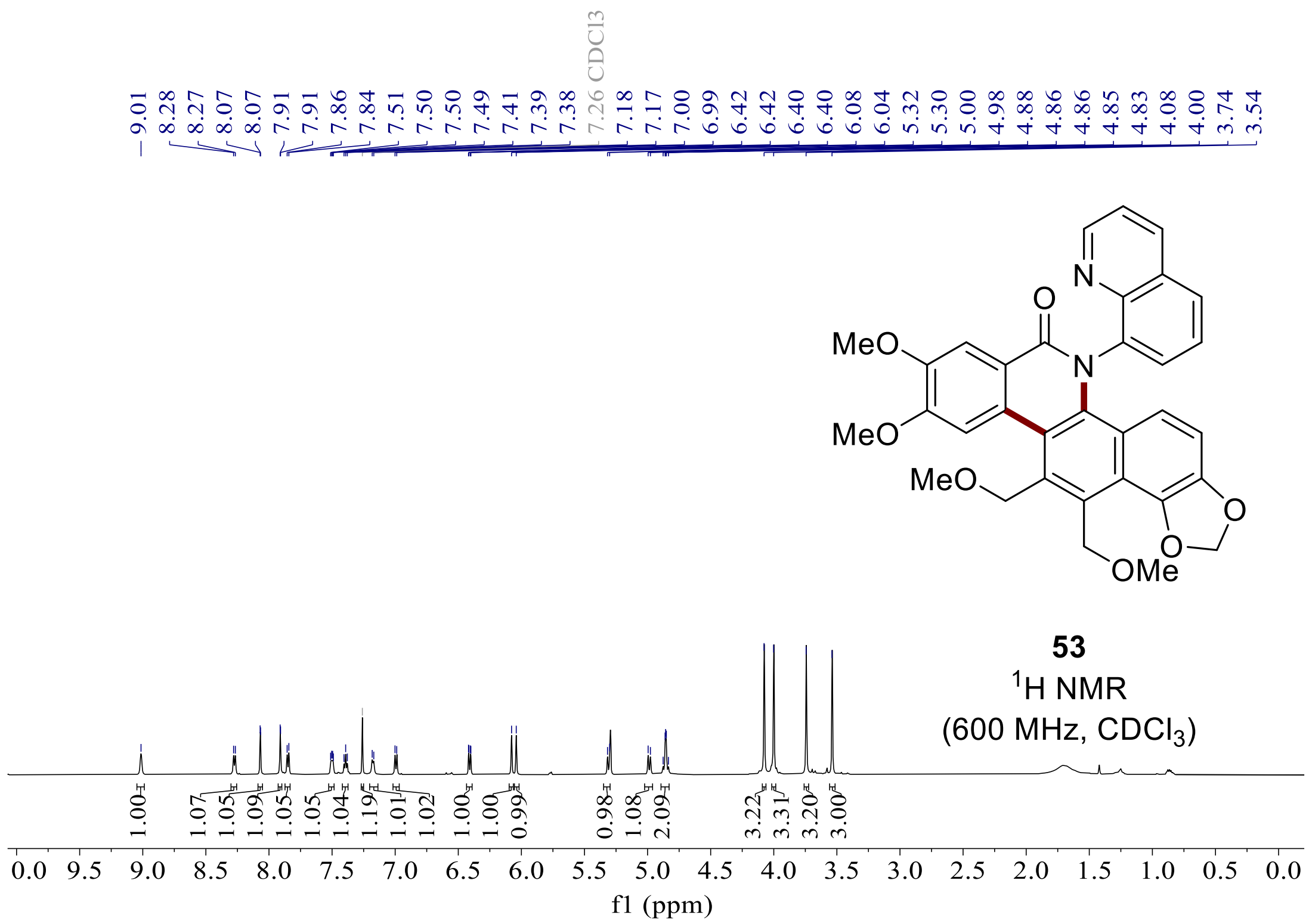
9.01
8.28
8.27
8.07
8.07
7.91
7.91
7.86
7.84
7.51
7.50
7.50
7.49
7.41
7.39
7.38
7.26 CDCl3
7.18
7.17
7.00
6.99
6.42
6.42
6.40
6.40
6.08
6.04
5.32
5.30
5.00
4.98
4.88
4.86
4.86
4.85
4.83
4.08
4.00
3.74
3.54
MeO
MeO
MeO
OMe
O
N
N
O
O
53
1H NMR
(600 MHz, CDCl3)
1.00
1.07
1.05
1.09
1.05
1.05
1.04
1.19
1.01
1.02
1.00
1.00
0.99
0.98
1.08
2.09
3.22
3.31
3.20
3.00
f1 (ppm)

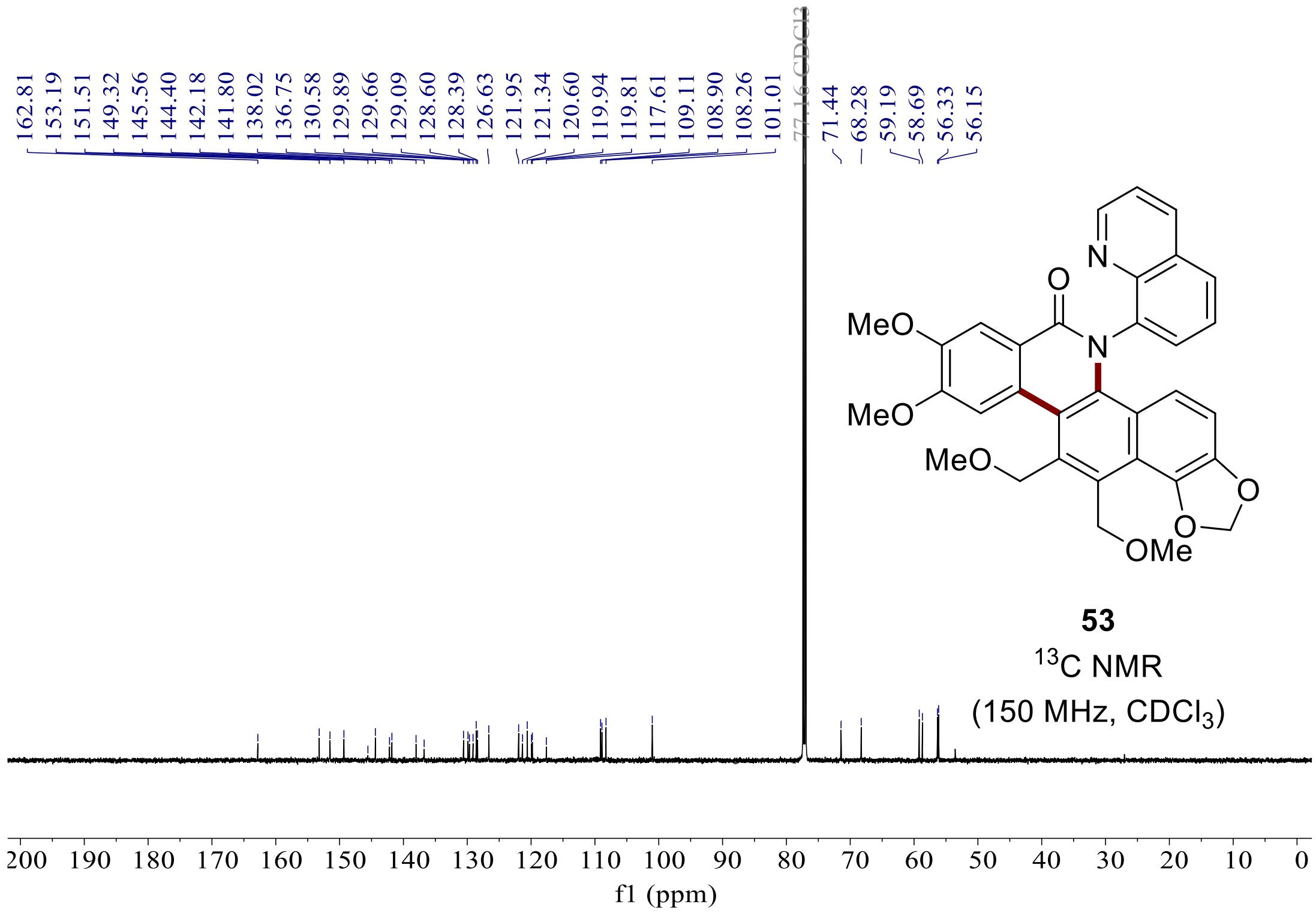
162.81
153.19
151.51
149.32
145.56
144.40
142.18
141.80
138.02
136.75
130.58
129.89
129.66
129.09
128.60
128.39
126.63
121.95
121.34
120.60
119.94
119.81
117.61
109.11
108.90
108.26
101.01
77.16 CDCl3
71.44
68.28
59.19
58.69
56.33
56.15
MeO
MeO
MeO
OMe
O
N
N
O
O
53
^{13}C NMR
(150 MHz, $CDCl_3$)
f1 (ppm)

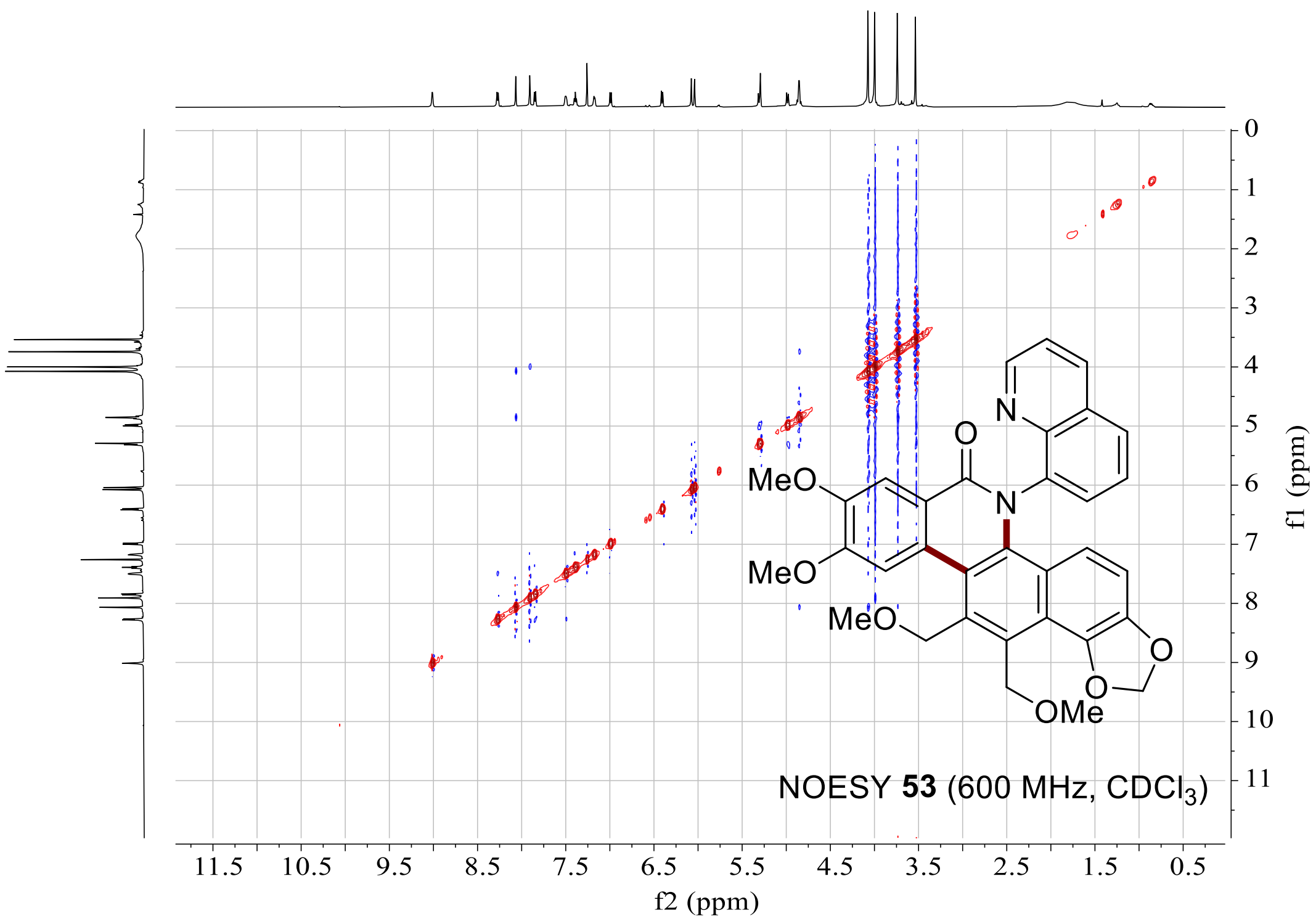
NOESY 53 (600 MHz, $CDCl_3$)
MeO
MeO
MeO
OMe
O
N
N
O
O
f2 (ppm)
f1 (ppm)

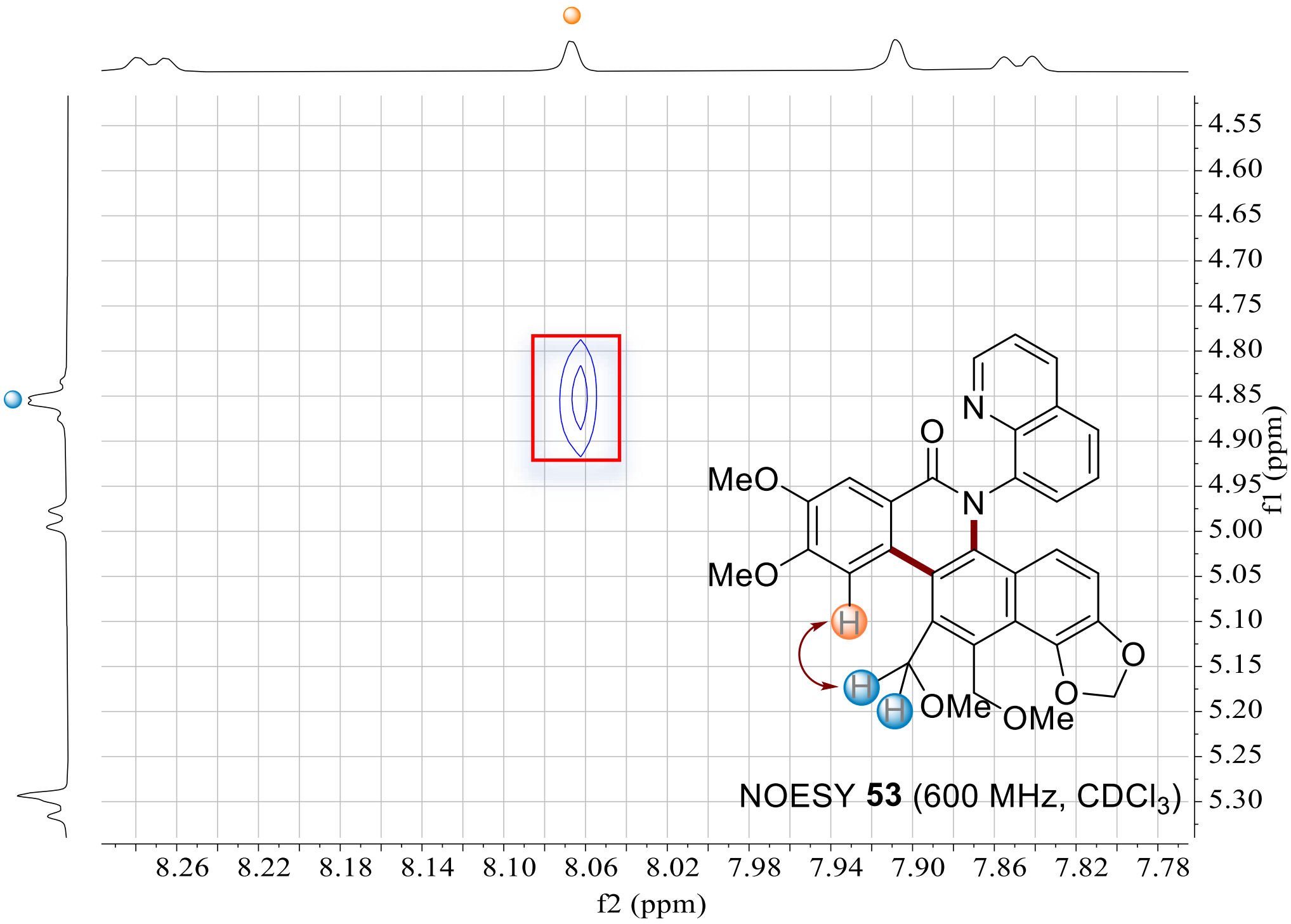
MeO
MeO
O
N
N
H
H
H
OMe
OMe
O
O
NOESY 53 (600 MHz, CDCl3)
8.26 8.22 8.18 8.14 8.10 8.06 8.02 7.98 7.94 7.90 7.86 7.82 7.78
f2 (ppm)
4.55 4.60 4.65 4.70 4.75 4.80 4.85 4.90 4.95 5.00 5.05 5.10 5.15 5.20 5.25 5.30
f1 (ppm)

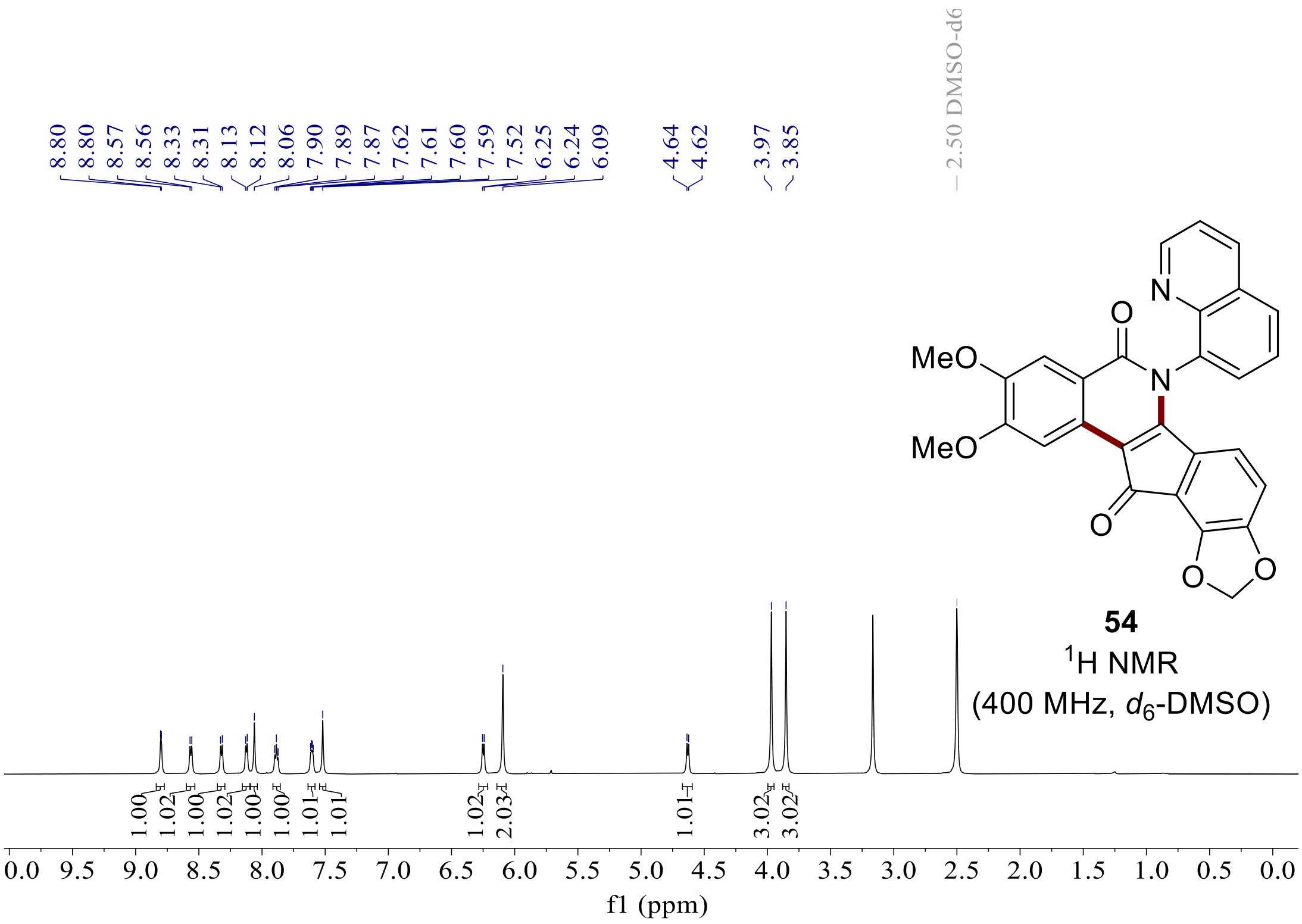
8.80 8.80 8.57 8.56 8.33 8.31 8.13 8.12 8.06 7.90 7.89 7.87 7.62 7.61 7.60 7.59 7.52 6.25 6.24 6.09
4.64 4.62
3.97 3.85
2.50 DMSO-d6
MeO
MeO
O
N
N
O
O
O
54
1H NMR
(400 MHz, d6-DMSO)
1.00 1.02 1.00 1.02 1.00 1.00 1.01 1.01
1.02 2.03
1.01
3.02 3.02
0.0 9.5 9.0 8.5 8.0 7.5 7.0 6.5 6.0 5.5 5.0 4.5 4.0 3.5 3.0 2.5 2.0 1.5 1.0 0.5 0.0
f1 (ppm)

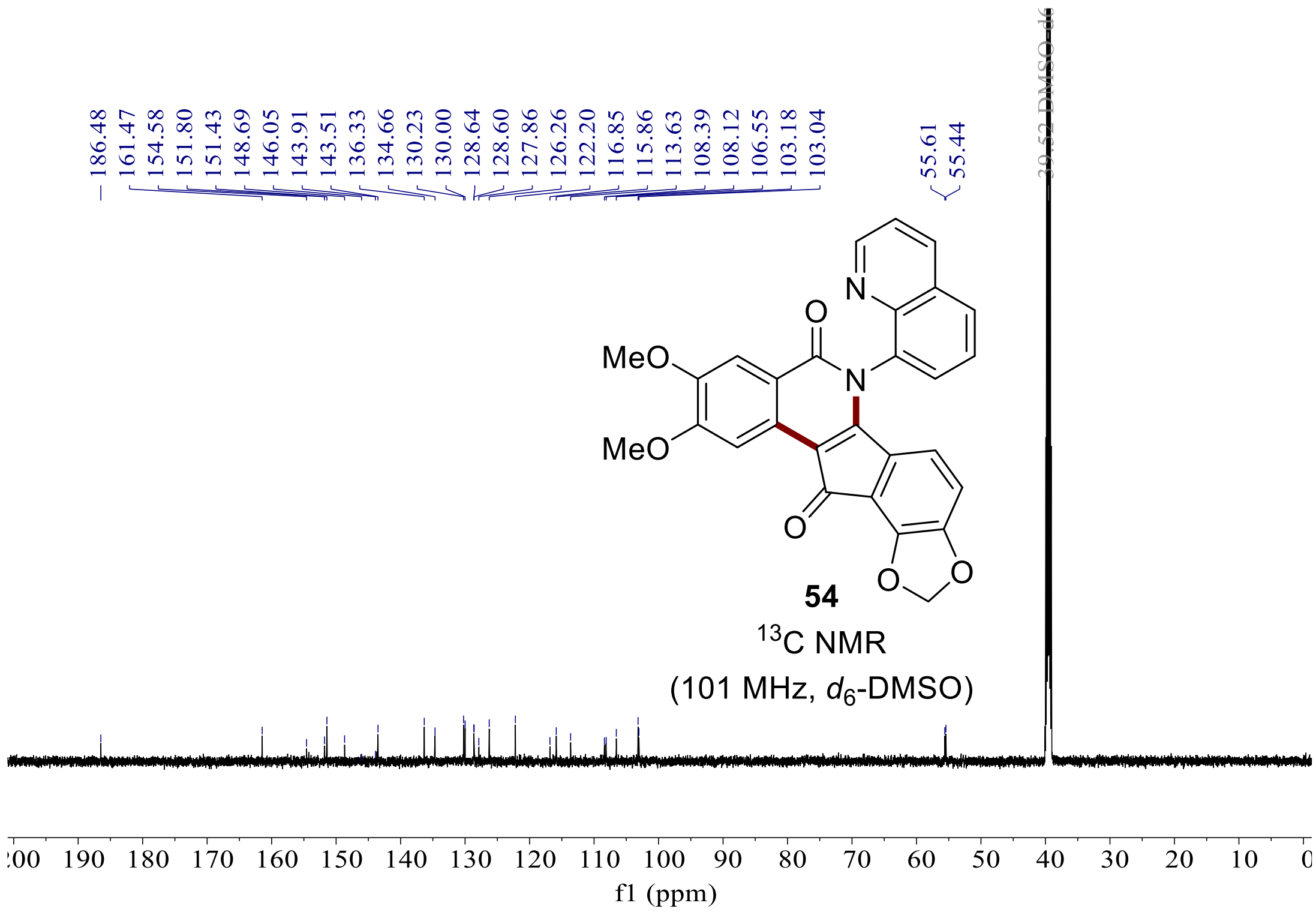

186.48
161.47
154.58
151.80
151.43
148.69
146.05
143.91
143.51
136.33
134.66
130.23
130.00
128.64
128.60
127.86
126.26
122.20
116.85
115.86
113.63
108.39
108.12
106.55
103.18
103.04
55.61
55.44
39.52 DMSO-d6
MeO
MeO
O
N
N
O
O
O
54
13C NMR
(101 MHz, d6-DMSO)
200 190 180 170 160 150 140 130 120 110 100 90 80 70 60 50 40 30 20 10 0
f1 (ppm)
